# Neural network training method for materials science based on multi-source databases

**DOI:** 10.1038/s41598-022-19426-8

**Published:** 2022-09-12

**Authors:** Jialong Guo, Ziyi Chen, Zhiwei Liu, Xianwei Li, Zhiyuan Xie, Zongguo Wang, Yangang Wang

**Affiliations:** 1 Computer Network Information Center, Chinese Academy of Sciences, Beijing, 100083 China; 2 University of Chinese Academy of Sciences, Beijing, 100049 China; 3 China Petroleum Pipeline Engineering Co., Ltd., International, Langfang, 065000 Hebei China; 4 Department of Physics, Renmin University of China, Beijing, 100872 China

**Keywords:** Scientific data, Engineering, Materials science

## Abstract

The fourth paradigm of science has achieved great success in material discovery and it highlights the sharing and interoperability of data. However, most material data are scattered among various research institutions, and a big data transmission will consume significant bandwidth and tremendous time. At the meanwhile, some data owners prefer to protect the data and keep their initiative in the cooperation. This dilemma gradually leads to the “data island” problem, especially in material science. To attack the problem and make full use of the material data, we propose a new strategy of neural network training based on multi-source databases. In the whole training process, only model parameters are exchanged and no any external access or connection to the local databases. We demonstrate its validity by training a model characterizing material structure and its corresponding formation energy, based on two and four local databases, respectively. The results show that the obtained model accuracy trained by this method is almost the same to that obtained from a single database combining all the local ones. Moreover, different communication frequencies between the client and server are also studied to improve the model training efficiency, and an optimal frequency is recommended.

## Introduction

Materials are closely related to technological innovation and economic development. Government have always paid more attention to the competition in the field of materials. In order to promote the development of materials, the United States announced the “Materials Genome Initiative” in 2011, to shorten the research cycle of new materials and decrease the cost of material research through the collaborative cooperation of advanced experimental, computation and database technologies. China, Japan and European Union have also launched similar research plans to seize the opportunity in a new round of competition in materials science^1^. During this period, high-throughput experiment, high-throughput computing and material database technologies have achieved rapid development and extensive application, and some representative computational material databases, such as Materials Project^2^, the Open Quantum Materials Database (OQMD)^3^ have been established. In addition, the development of artificial intelligence technology has further accelerated the transformation of scientific paradigm with “Data-Driven” mode in material field. Among them, support vector machine, random forest and neural network algorithms have become common data processing and analysis tools in the field of materials, and they have achieved remarkable results in the prediction of properties^4^, phase diagram^5^ and structures^6,7^. Data is an important factor of the data-driven scientific paradigm^8^, and its quality determines the upper limit of the predictive power of the model, while the improvement of model is only to constantly approach this upper limit. In recent years, many material databases have been established with a large number of data with the help of high-throughput technologies. However, the “data island” problem in materials science is very serious, due to lacking of security sharing strategy of material data. Firstly, a number of high-quality material data which are difficult to obtain are still stored locally in different institutions, and collecting these data needs to consume significant network bandwidth and tremendous time. Secondly, the data opening and sharing strategy is still imperfect. Researchsers in special material area who own the raw data have the absolutely initiative in the cooperation, and they usually refuse to share the raw data to maintain their advantageous. To best of our knowledge, there is no study on how to use the data located in different institutions without getting the raw data, although data protection in material science has been paid more and more attention. The suggestion, providing the access permission of database for their trust users^9^, has given a data sharing way but failed to protect the local data of the client from disclosure. Therefore, to explore the value-added potential of material data and improve the innovation ecosystem in material science, it is urgent to develop a new neural network training method based on multiple databases without remote raw data access for material researchers.

The successful application of federated learning strategy on medical^10–13^, finance, IoT^14–17^ and other fields^18^ provides a new important enlightenment to realize the secure sharing of material data. Federated learning^19^ is an emerging technology in the field of artificial intelligence. This technology was proposed to solve the problem of protecting users’ private data^20,21^, and it has developed a “Federated Transfer Learning” solution in the financial field combined with transfer learning^22^. Federated learning shows obvious advantages in effectively solving “data island” problem. It realizes the data sharing of each user while ensuring the user’s data security and personal data privacy, and carries out efficient machine learning among multiple participants or multiple computing nodes. As a machine learning framework, federated learning can be applied to neural networks and other algorithms. It is expected to become the basis of the next generation of artificial intelligence cooperative algorithms and networks.

Horizontal federated learning scheme is suitable for the situation that the features of material data in different datasets have a lot of overlap but the types of materials have no overlap^23,24^. According to the characteristics of target material dataset we investigate, horizontal federated learning scheme is adopted to realize the material data sharing and model training. Under this strategy, each user can get and use the global model without accessing others’ local datasets, so as to reduce the risk of user information leakage and achieve the purpose of data sharing.

**Figure 1:**
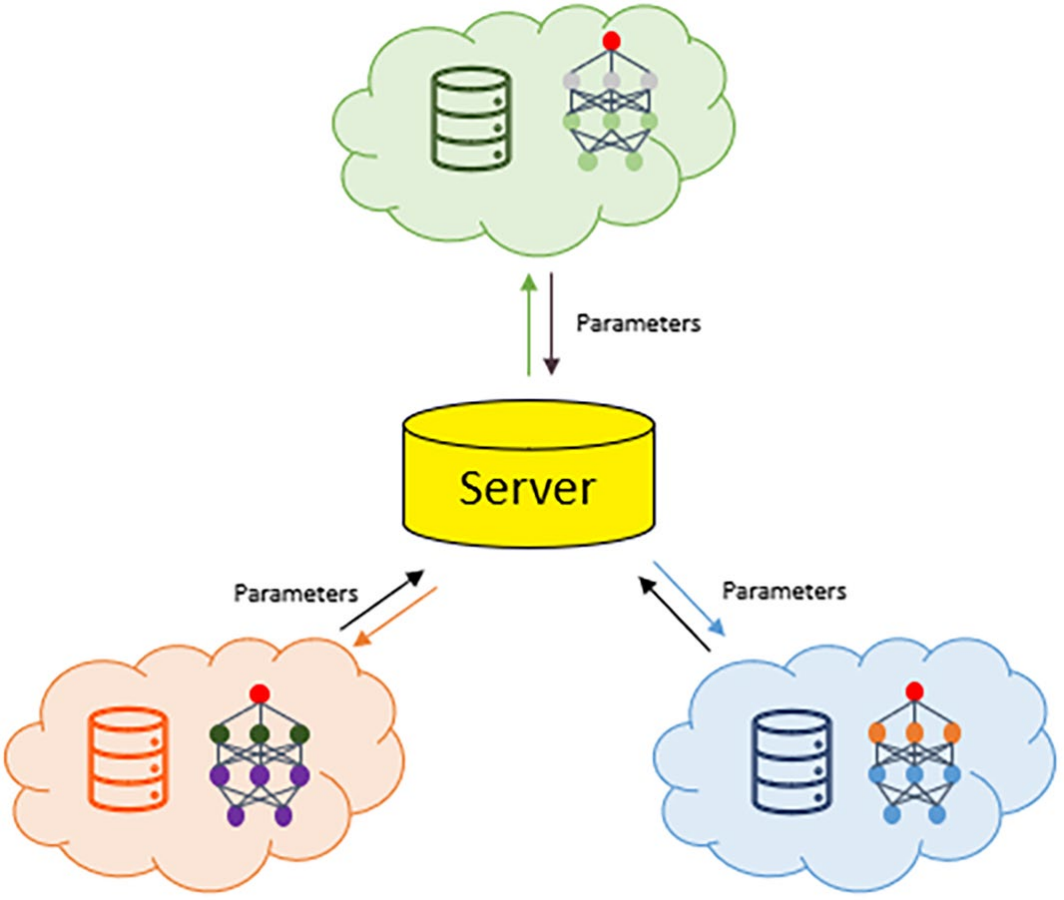
Schematic diagram of model training framework with multi-source databases. This framework contains three clients and one “Sever”. Each client has one local dataset in this illustration, and the “Server” is used to receive and send the model parameters.

In this paper, we have proposed a new neural network training method based on multi-source database which combines the federated learning schema and characteristics of material data. The illustration of model training framework with multi-database used in this paper is shown in Fig. 1. We use this method to predict the relationship between structure and its formation energy with two clients, the accuracy of predicted model is similar to that trained from all the training data directly. To investigate the influence of client numbers and data distribution on the model accuracy, four clients with different data distribution are used to train a neural network model. Different communication frequencies are studied and a suitable communication frequency that balances time and model performance is provided. This work supplies an important way and universal workflow to promote data sharing and improve the accuracy of material property prediction in the filed of materials science.

## Results

### Formation energy prediction from two databases

Neural network is a mathematical model that imitates the behavior characteristics of animal neural network. It is based on artificial neurons, and each neuron has an associated weight and threshold which are considered as the parameters of network used in materials science. Our aim is to train a shared model based on multi-source databases by sharing the parameters. The material dataset used in this work is randomly divided into training set and test set, training set has 10,000 terms of data and test set has 2,897 terms of data. The training set is equally divided into two subsets, each subset contains 5000 terms of data, and the two datasets are respectively placed on two clients located in two institutions. Using these two clients to train the relationship between structures and their formation energies.

**Figure 2:**
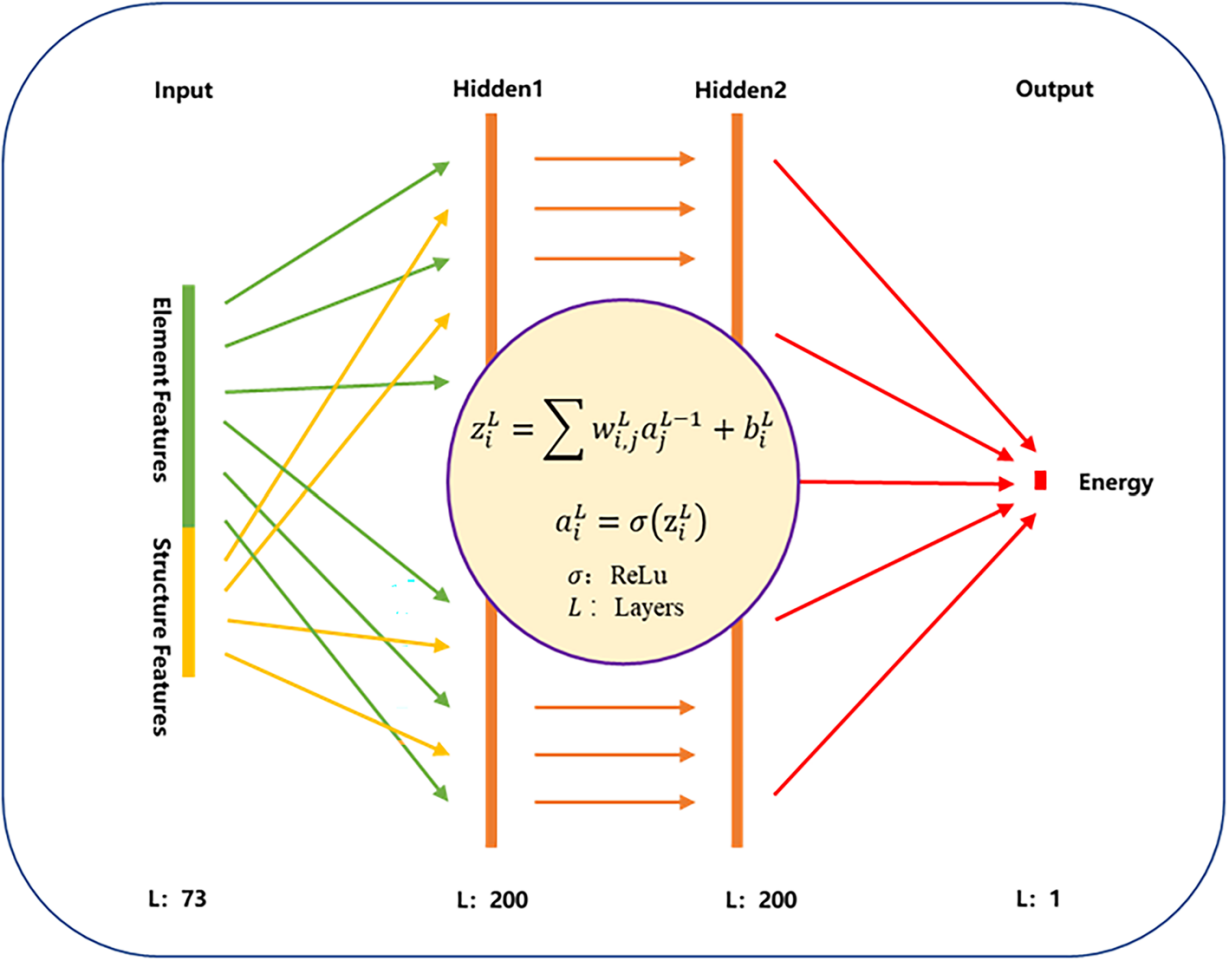
Neural network structure diagram, the neural network is a four layer fully connected network, the activation function is ReLu, the input includes element and structure features, and the output is formation energy of structures.

Both structure and element features are extracted and recalculated to as the input of the training network. The structure feature includes lattice constant, unit cell volume and space group number. The element feature includes row, column and block in periodic table, atomic mass, atomic radius, Mendeleev number, resistivity, speed of sound, thermal conductivity, melting point, Young’s modulus, and coefficient of linear thermal expansion. The recalculated method includes five types of statistical information: minimum, maximum, range, mean and variance. The formation energies are extracted to as the output of the network. The schematic diagram of the neural network used in this work is shown in Fig. 2. The dimension of input layer is 73, the dimension of the two hidden layers is 200, the learning rate is set to 0.001, the batch size is set to 200, the loss function is set to MSELoss function, and the total number of iteration steps is set to 100. The structure features and their formation energies that can be directly used in the training process for neural network are provided in Supplementary information.

**Figure 3:**
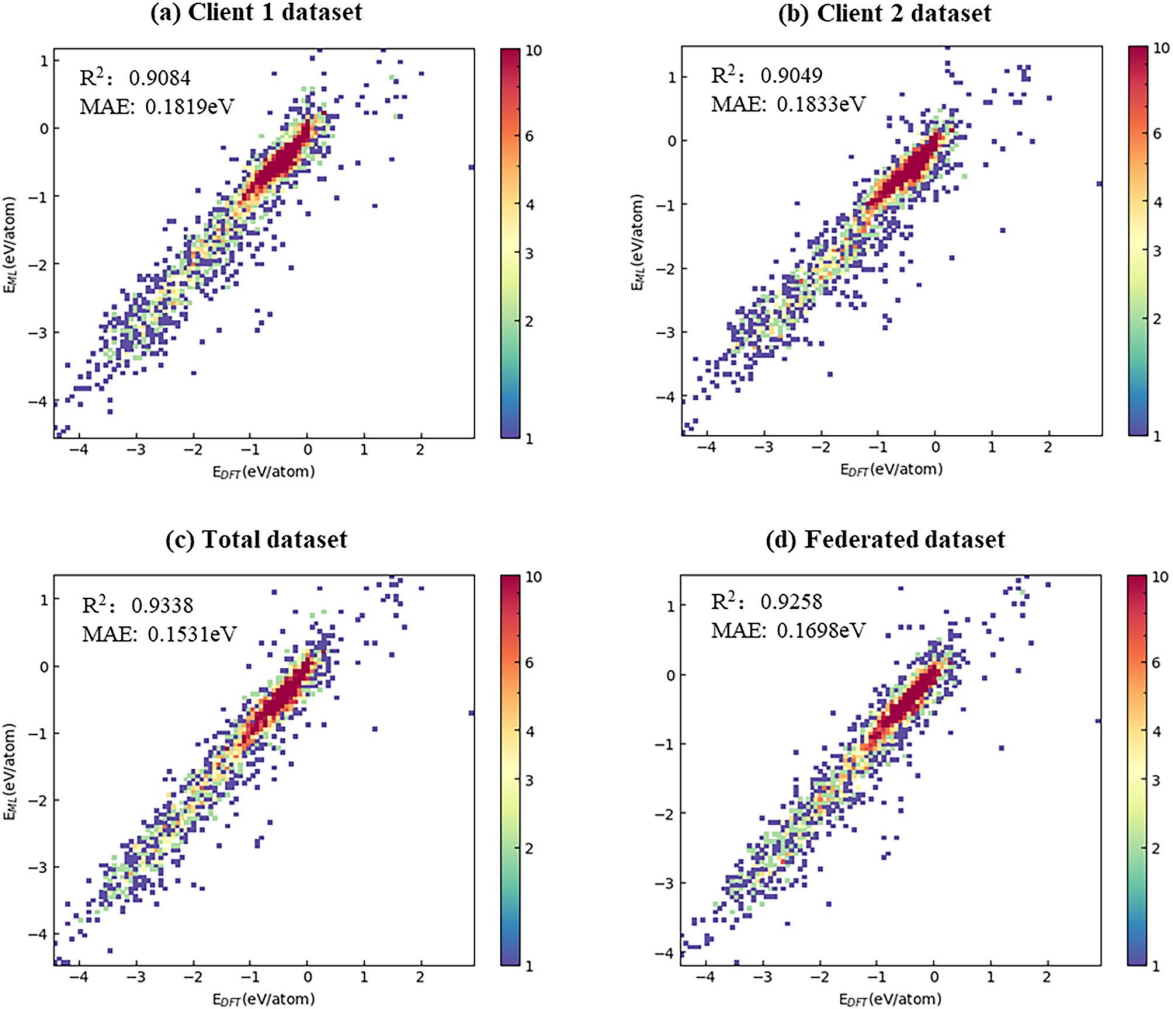
Prediction power of models trained on different datasets and method. (**a**) is for the model trained on the local dataset of client 1, (**b**) is for the model trained on the local dataset of client 2, (**c**) is for the model trained on the total training set, and (**d**) is for the shared model based on federated dataset (client 1 and 2) by using the method we proposed. Among them, different colors indicate aggregation degrees of points, red indicates that the point distribution is clustered, and blue indicates that the point distribution is sparse.

Using the same neural network, four models are trained based on different datasets. Two models are respectively trained based on the dataset of each client, the third one is trained based on the total training set (which merges the two datasets in client 1 and 2 together), and the fourth one is also trained based on the total training set but the two datasets are still respectively localted in the different clients, and this type of dataset is called as federated dataset and the fourth model is named as shared model in the following sections. The four models are verified on the same test set, and the coefficient of determination ($R^{2}$) and the Mean Absolute Error (MAE) of all models are shown in Fig. 3.

It can be seen that the prediction power of shared model is significantly improved than that of model trained from client 1 or client 2. In the training process, only the network parameters information is transmitted, and it is also able to encrypt data transmissions to improve the safety of data. Therefore, using the new neural network training method we proposed to train a shared model on multiple datasets can prevent the local data of the client from being directly obtained by others. At the same time, we should see the model performance is a little bit worse than that directly trained from total training set, but this can be further improved by optimizing the algorithms.

### Training model based on four databases with different data distribution

**Figure 4:**
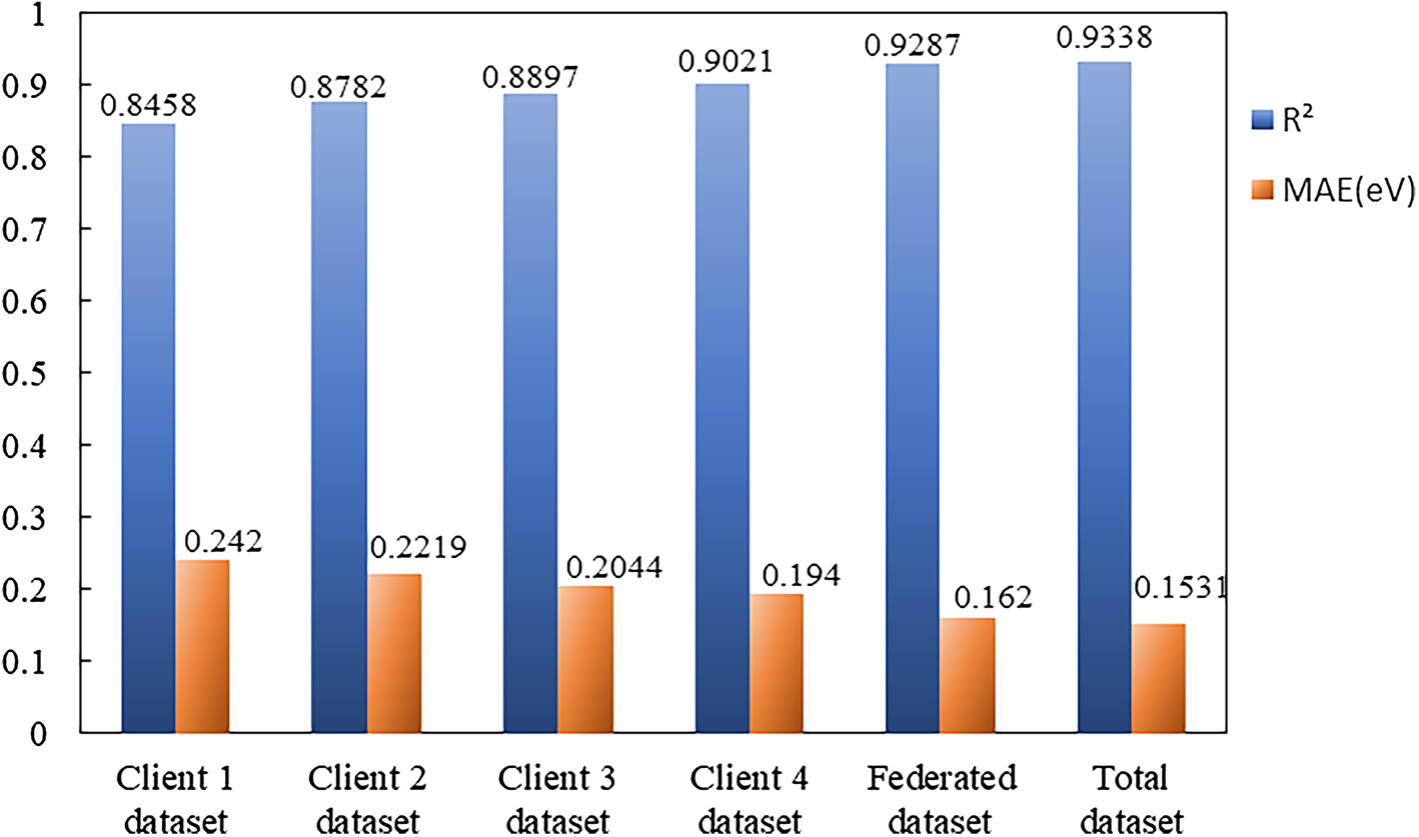
Column chart of shared model and individual trained model on each local dataset.

In the previous section, two clients with the same size of training sets are used to construct the model training framework and train a shared model. However, the situation in materials science is more complex, which usually has multiple databases from different research groups or institutions in the same field. It is necessary to consider the applications of multiple clients with different sizes of datasets. The shared model trained from four clients with different sizes of datasets is further investigated by the training framework. The sizes of total training set and test set are still 10,000 and 2,897, respectively, but the training data set is scattered into four subsets and stored into four different clients (labeled 1, 2, 3, and 4). The local dataset sizes are respectively set to 1000, 2000, 3000, and 4000, forming a total training set containing 10,000 terms of data. The same network structure and parameters shown in Fig. 2 are used. The shared model is trained based on four datasets located in four clients, the $R^{2}$ and MAE are shown in Fig. 4.

It can be seen that when each client uses its local dataset to train a model, the model trained by more data generally has a better performance on the test set. The shared model trained from multi-source datasets still performs more better than that from individual dataset. And compared with the case of two clients with same size of dataset, the prediction power of model does not decrease. This shows that the model training framework we used still has a stable performance in the case of more clients and uneven data distribution.

It is noticed that the choice of batch size for the case based on the total dataset is different with that based on multi-source datasets. When the model is training based on total training set, each batch size is randomly selected from the dataset, while each batch is a proportional combination of those extracted from each client when the model is training based on multi-source datasets located in different clients. Comparing these two methods, the former is more flexible, but this cannot be used as the absolute advantage for model training. Depending on the characteristics of dataset, the model training based on multi-source datasets may have a better performance than that based on the total training set.

### Communication frequency optimization

During the training process, a communication is that network parameters between server and client have one exchange. Training a shared model from two clients and four clients in the above two experiments, there will be a communication between the server and the client once the network parameters of the local model are updated. When the size of client’s local dataset is 5000, if both the batch size and iteration steps are set as 100, the number of communications will reach to 5000 in a local model training process and more time is need to finished a training process. In order to reduce the communication cost and improve the training efficiency, an in-depth study is carried out on the variation of prediction power of the training model with different communication frequencies.

**Figure 5:**
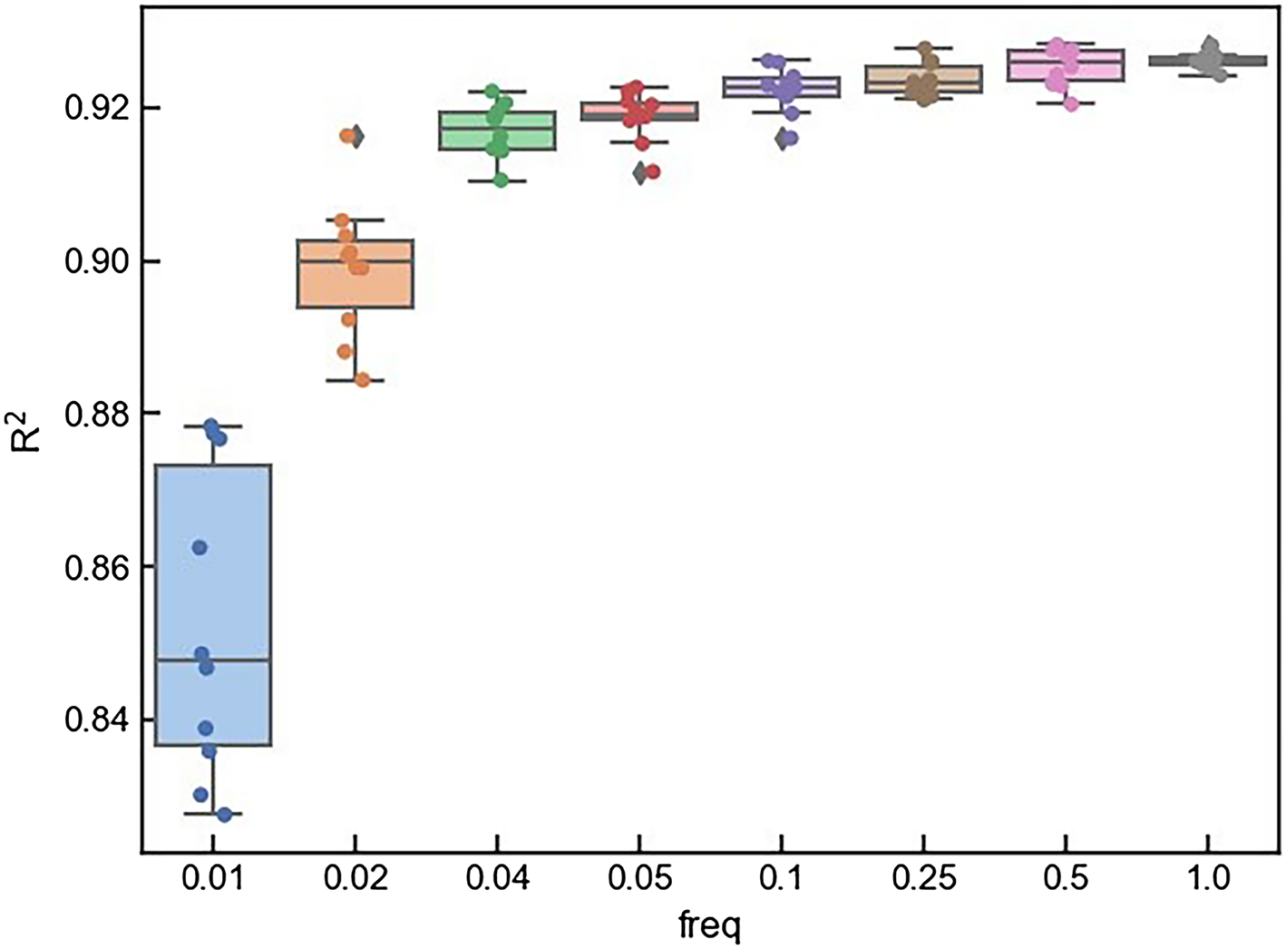
Prediction power of shared model based on four datasets with different communication frequencies from 10 times of experiments. Colors present different communication frequencies. The two ends of the rectangular box are respectively the upper quartile and lower quartile of $R^{2}$ according to current communication frequency, the horizontal line in the middle of the rectangle is the median, and the short horizontal lines outside of the rectangular box are the largest and smallest values of $R^{2}$ of ten data.

In this experiment, shared model is trained based on four clients and eight cases are considered, i.e., the communication frequency is respectively set to 0.01, 0.02, 0.04, 0.05, 0.1, 0.25, 0.5 and 1.0. When the total number of iteration steps is 100, the corresponding times of model training communication are 1, 2, 4, 5, 10, 25, 50 and 100, respectively. Take 10 times as example, the client will update its network parameters to the server when ten iteration steps are completed in the client. We have repeated the experiments ten times for all these eight cases. The performances of the trained model on the test set for each communication are shown in Fig. 5.

It can be seen that the prediction power of the model on the test set gradually increases with the increase of communication frequency. When the communication frequency is low, the shared model is obviously unstable, and it has a terrible performance than that from a single client dataset. With the increase of communication frequency, the prediction power of shared model rapidly increases, and the trend of increase will be slow when it reaches a desired level. Meanwhile, the fluctuation range of the $R^{2}$ in ten times of training processes generally decreases, and the shared model tends to be stable. It also can be seen that the model training framework can choose an appropriate communication frequency to balance the model performance and communication cost. In this experiment, when the model frequency is 0.25, it can both greatly reduce the communication cost and ensure the high prediction accuracy of model. In addition, the fewer communication times decreases the risk of data privacy leakage and increases the privacy-preserving capability in the model training process.

**Figure 6:**
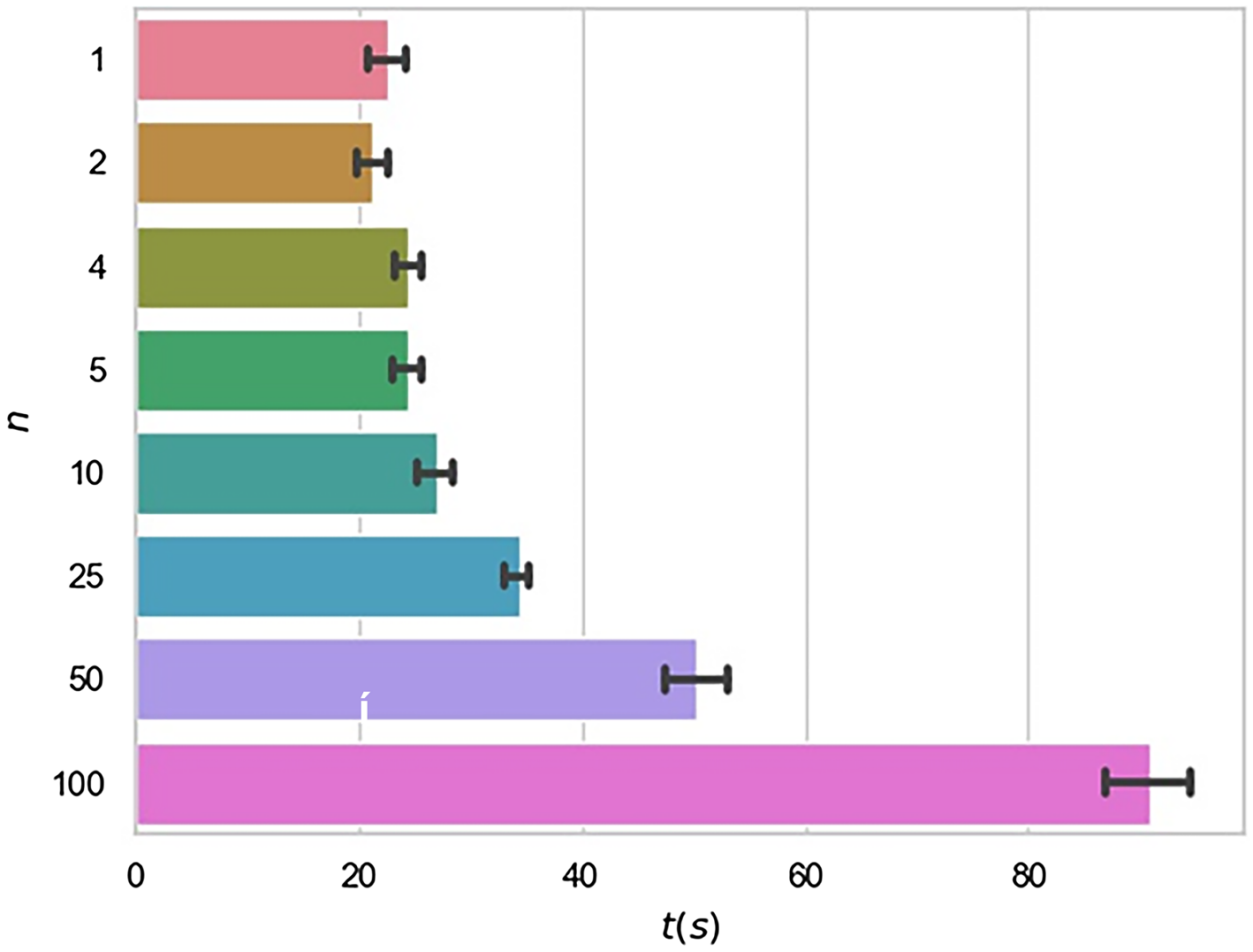
Bar chart of training time consuming with different communication times for model trained based on four clients. *t* is the time spent on model training, *n* is the communication times.

The training time of model with different communications has been also analyzed and the results are shown in Fig. 6. Among them, the training time have been mainly spent on communication between the client and the server and model gradient update calculation in the local client. It can be seen from the Fig. 6 that when the communication times is less than 10, the time of model gradient update calculation is dominant and the total training time keeps generally stable. With increasing the times of communications, the time spent on communications will be significantly longer than that on the model gradient update calculation. It is worth noting that the time up to 2710.45s (not shown) for the case that a communication will occur once the local model is updated in each client, which is longer than that in any case shown in Fig. 6, however, the prediction power has not been significantly improved. From Fig. 6, when the communication frequency is 0.25, both the accuracy and training efficiency of model have higher performance. In this way, the network bandwidth consumption has been reduced effectively.

## Discussion

In this paper, a new neural network training method for multi-source databases is proposed, and shared models between structures and their formation energies have been respectively trained based on two and four clients by using this method. Both of uniform and uneven data distributions in each client are investigated, and the results show that, the coefficient of determination $R^{2}$ of model trained on two clients with uniform data is closer to that trained on four clients with uneven data, and the model biases from both is controlled below 1$\%$ comparing to the model trained based on total training set. Through the study of different communication frequencies, a reasonable communication frequency has been recommended, and the prediction power is kept excellent as the same time as communication costs are decreased.

One of important goals of this method we proposed is to provide a way to protect the data privacy, and make researchers take the initiative to sharing their valuable data for model training in materials science. In the future work, technologies such as blockchain and differential privacy will be combined to obtain stronger privacy protection effects. Neural network training framework we used can effectively ensure the security and traceability of data by storing model parameters as transaction records in the blockchain. In addition, to get a more precise model, some technologies such as sample alignment, stack pollution intelligent detection^25^, and gradient screening and shielding^26^ methods will also be used to preprocess material data and reduce the interference of “noisy” data in the process of training universal model^27^ for different material properties.

The neural network training method this work proposed performs a success in the structure-formation prediction of materials. The accuracy and prediction power of models are improved with the participation of multi-source databases while safeguarding the data privacy. This work provides a new data application method and cooperation strategy for researchers to voluntarily share scientific data. It also supplies an important pathway to construct big data ecosystem and accelerate the transformation of scientific research paradigms in materials science.

## Methods

The neural network training framework includes the server and the client (shown in Fig. 1). The server collects, processes and shares the model parameters to all clients, the client uses the local dataset to train a model and updates parameters of the model. The client re-uploads the model parameters to the server when new model parameters are obtained from local dataset. And then, the server receives the model parameter data, calculates the weighted average of the model parameters, and distributes them to the client to start a new round of model training. The network parameters returned to each client by the server is calculated by Eq. (1):

1 $$\begin{aligned} w = \sum _{i} \alpha _{i} w_{i} \end{aligned}$$

where $w_{i}$ is the network parameter uploaded by client *i*, $\alpha _{i}$ is the weight of the client’s local dataset in the total dataset.

### Server

The server is used to realize the communication between different datasets. At the beginning, the server will connect with the clients of each institution or group who owns the dataset, and unify the initial model parameters trained on each client, such as iteration step, batch size, learning rate, optimizer, loss function and network structure. Then, the server will initialize the network model parameters and distribute the parameters to each client. After that, the server will wait for all clients to upload the updated network parameters. After all client network parameters are uploaded to the server, the server will perform a weighted average operation on all network parameters, and redistribute the operated network parameters to each client. Iterate continuously until the global model converges. The algorithm for sever used in this method is shown in Algorithm 1.
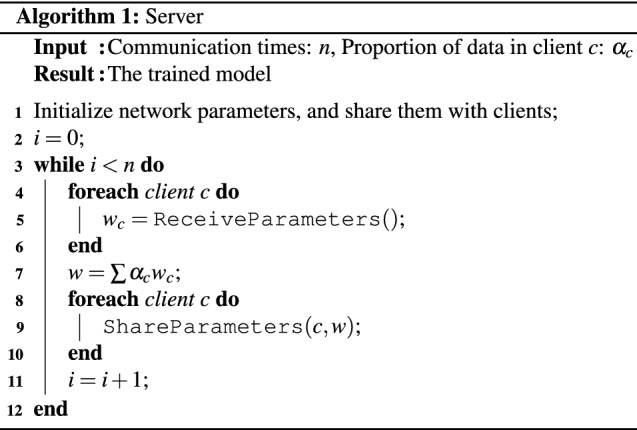


### Client

The client is used to implement the training process of the neural network model on the local dataset. First, the client will wait for the initial model parameters distributed by the server. Every client receives the model parameters and load them to the local network model. The local network model will read data from the local dataset, perform feedforward and backpropagation operations of the neural network, and update the weight parameters of the network. When the local model parameters are updated, the client will upload the local model parameters to the server and wait for the next round of local network model parameters’ update. The algorithm for client is shown in Algorithm 2.
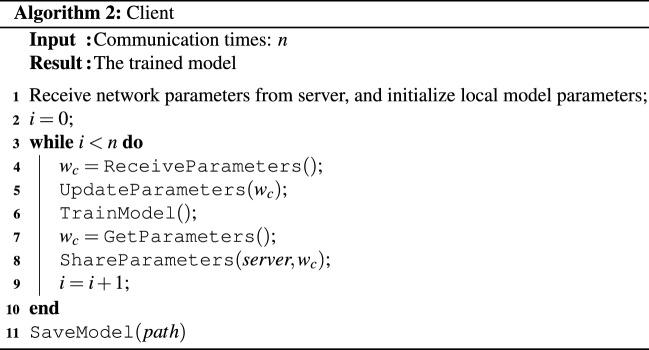


### Parameters transfer

**Figure 7:**
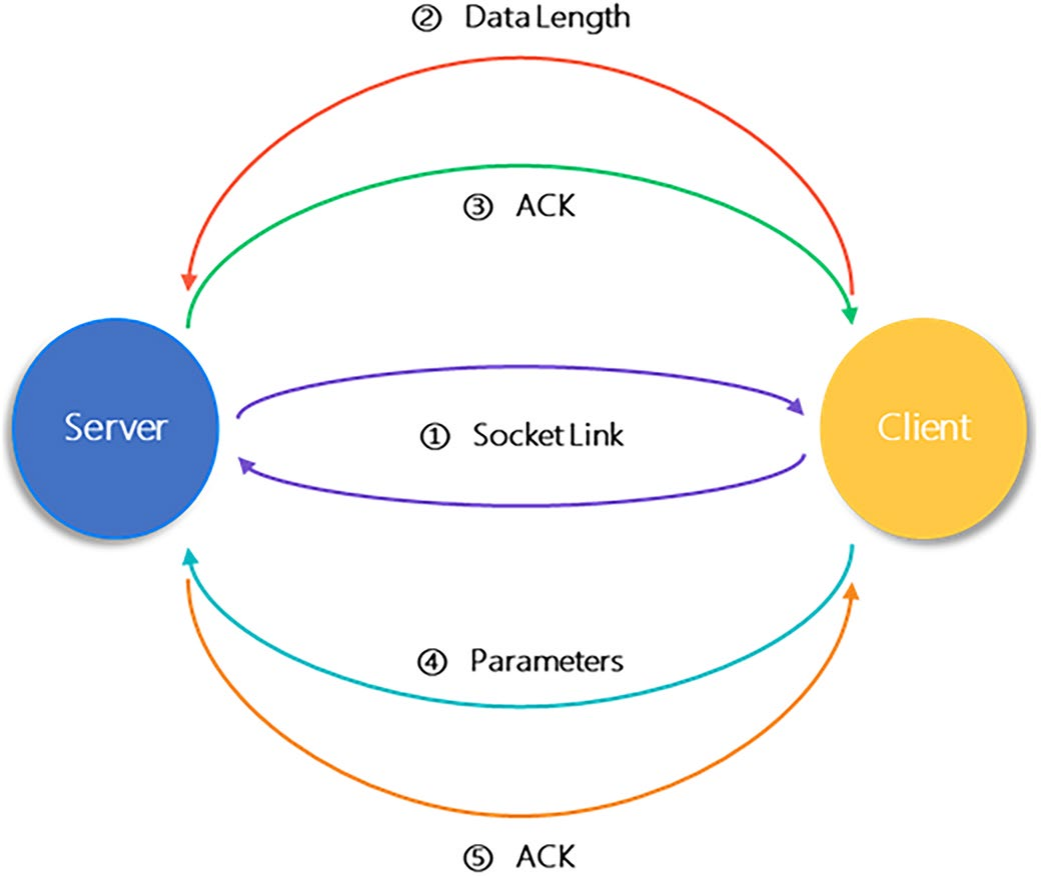
Diagram of the communication strategy between the server and the client.

In this method, socket is used to realize the communication between the server and the client. The transmission process of the network parameters includes the model parameters serialization and deserialization. In addition, in order to avoid missing the received parameters data, the length of communication data will be firstly sent, and the model parameters will be sent when the acknowledge character (ACK) returned by receivers is received. The model parameters are received according to the data length. After the data is completely received, the acknowledge character is returned again, and the sender completes the transmission process after receiving the acknowledge character. The communication process is shown in Fig. 7.

### Dataset

The data used in this work is from The Open Quantum Materials Database (OQMD), and 12,897 terms effective material data containing composition, unit cell, sites and formation energy are collected. Each data and its expanding information are extracted and stored in the database for model training. All data information can be seen from the supplementary information.

## Supplementary Information

Supplementary Information.

Supplementary Information.

## Data Availability

The datasets used and/or analysed during the current study available from the corresponding author on reasonable request.
