# Supplementary material for: Neural network training method for materials science based on multi-source databases
Source: Sci Rep. 2022 Sep 12;12:15326. doi: 10.1038/s41598-022-19426-8 (PMC9468338; doi:10.1038/s41598-022-19426-8)
Supplement: Supplementary file 2 — Supplementary Information. [file 41598_2022_19426_MOESM2_ESM.pdf]

Neural Network Training Method for Materials Science based on Multi-source Databases

Jialong Guo[1,2], Ziyi Chen[1,2], Zhiwei Liu[1,2], Xianwei Li[3], Zhiyuan Xie[4], Zongguo Wang[1,2,*], and Yangang Wang[1,2]

[1]Computer Network Information Center, Chinese Academy of Sciences, Beijing, 100083, China

[2]University of Chinese Academy of Sciences, Beijing, 100049, China

[3]China Petroleum Pipeline Engineering Co., Ltd., International, Langfang Hebei, 065000, China

[4]Department of Physics, Renmin University of China, Beijing, 100872, China

*Corresponding author: Zongguo Wang, wangzg@cnic.cn

Supplementary information

The structure features and their formation energies that can be directly used in the training process for neural network.

1	2	3	4	5	6	7	8	9	10	11	12	13	14	15	16	17	18	19	20	21	22	23	24	25	26	27	28	29	30	31	32	33	34	35	36	37	38	39	40	41	42	43	44	45	46	47	48	49	50	51	52	53	54	55	56	57	58	59	60	61	62	63	64	65	66	67	68	69	70	71	72	73	energy
0.79	2.58	1.79	2.03429	0.89127	3	8	5	4.14286	2.40832	1	16	15	13.1429	6.49615	1	3	2	2	0.68313	28.0855	164.93	136.845	64.883	68.8212	1	2.6	1.6	1.35	0.72858	8	94	86	70.2857	44.7474	2.10E-07	1.00E+23	1.00E+23	5.71E+22	6.32E+22	0	0	0	0	0	0.205	150	149.795	28.9743	65.1228	301.59	1734	1432.41	753.719	774.432	1.6	100	98.4	24.6286	42.0937	0	0	0	0	0	760.627	19	6.36095	6.70465	17.835	1.5708	1.5708	1.5708	-1.6089
1.25	1.25	0	1.25	0	8	8	0	8	0	15	15	0	15	0	3	3	0	3	0	168.934	168.934	0	168.934	0	1.75	1.75	0	1.75	0	21	21	0	21	0	6.76E-07	6.76E-07	0	6.76E-07	0	0	0	0	0	0	17	17	0	17	0	1818	1818	0	1818	0	45	45	0	45	0	1.33E-05	1.33E-05	0	1.33E-05	0	88.6111	166	3.49522	3.49522	8.61514	1.36653	1.77507	2.0944	0.00639
1.1	1.1	0	1.1	0	8	8	0	8	0	3	3	0	3	0	3	3	0	3	0	138.905	138.905	0	138.905	0	1.95	1.95	0	1.95	0	33	33	0	33	0	6.15E-07	6.15E-07	0	6.15E-07	0	2475	2475	0	2475	0	13	13	0	13	0	1193	1193	0	1193	0	28	28	0	28	0	1.21E-05	1.21E-05	0	1.21E-05	0	37.7862	229	3.66144	3.66144	3.66144	1.91063	1.91063	1.91063	0.13123
3	3	0	3	0	4	4	0	4	0	18	18	0	18	0	2	2	0	2	0	83.798	83.798	0	83.798	0	0	0	0	0	0	4	4	0	4	0	0	0	0	0	0	1120	1120	0	1120	0	0.00943	0.00943	0	0.00943	0	115.79	115.79	0	115.79	0	0	0	0	0	0	0	0	0	0	0	42.8308	225	3.92727	3.92727	3.92727	1.5708	2.0944	2.0944	0.00274
0.82	0.82	0	0.82	0	5	5	0	5	0	1	1	0	1	0	1	1	0	1	0	85.4678	85.4678	0	85.4678	0	2.35	2.35	0	2.35	0	9	9	0	9	0	1.33E-07	1.33E-07	0	1.33E-07	0	1300	1300	0	1300	0	58	58	0	58	0	312.46	312.46	0	312.46	0	2.5	2.5	0	2.5	0	0	0	0	0	0	89.5987	229	4.88249	4.88249	4.88249	1.91063	1.91063	1.91063	0.00072
1.5	1.5	0	1.5	0	6	6	0	6	0	5	5	0	5	0	3	3	0	3	0	180.948	180.948	0	180.948	0	1.45	1.45	0	1.45	0	52	52	0	52	0	1.35E-07	1.35E-07	0	1.35E-07	0	3400	3400	0	3400	0	57	57	0	57	0	3290	3290	0	3290	0	200	200	0	200	0	6.30E-06	6.30E-06	0	6.30E-06	0	18.7879	225	2.98399	2.98399	2.98399	1.5708	2.0944	2.0944	0.24226
1.63	1.63	0	1.63	0	4	4	0	4	0	5	5	0	5	0	3	3	0	3	0	50.9415	50.9415	0	50.9415	0	1.35	1.35	0	1.35	0	54	54	0	54	0	2.00E-07	2.00E-07	0	2.00E-07	0	4560	4560	0	4560	0	31	31	0	31	0	2183	2183	0	2183	0	160	160	0	160	0	8.40E-06	8.40E-06	0	8.40E-06	0	13.1686	229	2.57664	2.57664	2.57664	1.91063	1.91063	1.91063	0
0	0	0	0	0	2	2	0	2	0	18	18	0	18	0	2	2	0	2	0	20.1797	20.1797	0	20.1797	0	0	0	0	0	0	2	2	0	2	0	0	0	0	0	0	936	936	0	936	0	0.0491	0.0491	0	0.0491	0	24.56	24.56	0	24.56	0	0	0	0	0	0	0	0	0	0	0	22.212	225	3.15525	3.15525	3.15525	1.5708	2.0944	2.0944	0
1.61	1.61	0	1.61	0	3	3	0	3	0	13	13	0	13	0	2	2	0	2	0	26.9815	26.9815	0	26.9815	0	1.25	1.25	0	1.25	0	80	80	0	80	0	2.70E-08	2.70E-08	0	2.70E-08	0	5100	5100	0	5100	0	235	235	0	235	0	933.47	933.47	0	933.47	0	76	76	0	76	0	2.31E-05	2.31E-05	0	2.31E-05	0	16.4826	225	2.85658	2.85658	2.85658	1.5708	2.0944	2.0944	0.00079
0.82	0.82	0	0.82	0	4	4	0	4	0	1	1	0	1	0	1	1	0	1	0	39.0983	39.0983	0	39.0983	0	2.2	2.2	0	2.2	0	10	10	0	10	0	7.50E-08	7.50E-08	0	7.50E-08	0	2000	2000	0	2000	0	100	100	0	100	0	336.53	336.53	0	336.53	0	3.1	3.1	0	3.1	0	0	0	0	0	0	71.876	229	4.53665	4.53665	4.53665	1.91063	1.91063	1.91063	0.00031
0	0	0	0	0	2	2	0	2	0	18	18	0	18	0	2	2	0	2	0	20.1797	20.1797	0	20.1797	0	0	0	0	0	0	2	2	0	2	0	0	0	0	0	0	936	936	0	936	0	0.0491	0.0491	0	0.0491	0	24.56	24.56	0	24.56	0	0	0	0	0	0	0	0	0	0	0	10.2255	225	2.43631	2.43631	2.43631	1.5708	2.0944	2.0944	0.30914
1.2	1.2	0	1.2	0	8	8	0	8	0	10	10	0	10	0	3	3	0	3	0	157.25	157.25	0	157.25	0	1.8	1.8	0	1.8	0	27	27	0	27	0	1.31E-06	1.31E-06	0	1.31E-06	0	2680	2680	0	2680	0	11	11	0	11	0	1585	1585	0	1585	0	38	38	0	38	0	9.40E-06	9.40E-06	0	9.40E-06	0	32.5397	229	3.48347	3.48347	3.48347	1.91063	1.91063	1.91063	0.14608
1.91	1.91	0	1.91	0	4	4	0	4	0	10	10	0	10	0	3	3	0	3	0	58.6934	58.6934	0	58.6934	0	1.35	1.35	0	1.35	0	67	67	0	67	0	7.20E-08	7.20E-08	0	7.20E-08	0	4970	4970	0	4970	0	91	91	0	91	0	1728	1728	0	1728	0	180	180	0	180	0	1.34E-05	1.34E-05	0	1.34E-05	0	10.8604	229	2.41633	2.41633	2.41633	1.91063	1.91063	1.91063	0.0908
1.83	1.83	0	1.83	0	4	4	0	4	0	8	8	0	8	0	3	3	0	3	0	55.845	55.845	0	55.845	0	1.4	1.4	0	1.4	0	61	61	0	61	0	1.00E-07	1.00E-07	0	1.00E-07	0	4910	4910	0	4910	0	80	80	0	80	0	1811	1811	0	1811	0	170	170	0	170	0	1.18E-05	1.18E-05	0	1.18E-05	0	10.198	225	2.43413	2.43413	2.43413	1.5708	2.0944	2.0944	0.16145
1.9	1.9	0	1.9	0	5	5	0	5	0	7	7	0	7	0	3	3	0	3	0	98	98	0	98	0	1.35	1.35	0	1.35	0	59	59	0	59	0	2.20E-07	2.20E-07	0	2.20E-07	0	0	0	0	0	0	51	51	0	51	0	2430	2430	0	2430	0	0	0	0	0	0	0	0	0	0	0	14.5632	225	2.74109	2.74109	2.74109	1.5708	2.0944	2.0944	0.06853
1.9	1.9	0	1.9	0	6	6	0	6	0	7	7	0	7	0	3	3	0	3	0	186.207	186.207	0	186.207	0	1.35	1.35	0	1.35	0	58	58	0	58	0	1.80E-07	1.80E-07	0	1.80E-07	0	4700	4700	0	4700	0	48	48	0	48	0	3459	3459	0	3459	0	370	370	0	370	0	6.20E-06	6.20E-06	0	6.20E-06	0	14.9461	225	2.76491	2.76491	2.76491	1.5708	2.0944	2.0944	0.06205
2.28	2.28	0	2.28	0	6	6	0	6	0	10	10	0	10	0	3	3	0	3	0	195.084	195.084	0	195.084	0	1.35	1.35	0	1.35	0	68	68	0	68	0	1.06E-07	1.06E-07	0	1.06E-07	0	2680	2680	0	2680	0	72	72	0	72	0	2041.4	2041.4	0	2041.4	0	230	230	0	230	0	8.80E-06	8.80E-06	0	8.80E-06	0	15.5403	225	2.80107	2.80107	2.80107	1.5708	2.0944	2.0944	0
1.1	2.55	1.45	1.825	1.0253	4	8	4	6	2.82843	16	16	0	16	0	2	3	1	2.5	0.70711	78.96	173.04	94.08	126	66.5246	1.15	1.75	0.6	1.45	0.42426	17	93	76	55	53.7401	1.00E-08	2.50E-07	2.40E-07	1.30E-07	1.70E-07	1590	3350	1760	2470	1244.51	0.52	39	38.48	19.76	27.2095	494	1097	603	795.5	426.385	8.3	31	22.7	19.65	16.0513	0	0	0	0	0	51.3345	225	4.17165	4.17165	4.17165	1.5708	2.0944	2.0944	-2.1062
1.9	2.19	0.29	2.045	0.20506	3	3	0	3	0	14	15	1	14.5	0.70711	2	2	0	2	0	28.0855	30.9738	2.88826	29.5296	2.04231	1	1.1	0.1	1.05	0.07071	85	90	5	87.5	3.53553	1.00E-07	0.001	0.001	0.0005	0.00071	0	0	0	0	0	0.236	150	149.764	75.118	105.899	317.3	1687	1369.7	1002.15	968.524	11	100	89	55.5	62.9325	0	0	0	0	0	37.4149	216	3.75422	3.75422	3.75422	1.5708	2.0944	2.0944	0.10658
1.17	1.65	0.48	1.41	0.33941	4	8	4	6	2.82843	8	12	4	10	2.82843	3	3	0	3	0	65.409	150.36	84.951	107.885	60.0694	1.35	1.85	0.5	1.6	0.35355	28	76	48	52	33.9411	6.00E-08	9.40E-07	8.80E-07	5.00E-07	6.22E-07	2130	3700	1570	2915	1110.16	13	120	107	66.5	75.6604	692.68	1345	652.32	1018.84	461.26	38	70	32	54	22.6274	1.27E-05	3.02E-05	1.75E-05	2.15E-05	1.24E-05	47.8713	221	3.63099	3.63099	3.63099	1.5708	1.5708	1.5708	-0.3274
1.9	2.55	0.65	2.225	0.45962	2	3	1	2.5	0.70711	14	14	0	14	0	2	2	0	2	0	12.0107	28.0855	16.0748	20.0481	11.3666	0.7	1.1	0.4	0.9	0.28284	85	95	10	90	7.07107	1.00E-05	0.001	0.00099	0.00051	0.0007	2200	18350	16150	10275	11419.8	140	150	10	145	7.07107	1687	3800	2113	2743.5	1494.12	33	100	67	66.5	47.3762	2.60E-06	7.10E-06	4.50E-06	4.85E-06	3.18E-06	16.624	225	2.86473	2.86473	2.86473	1.5708	2.0944	2.0944	0.53432
1.33	1.33	0	1.33	0	5	5	0	5	0	4	4	0	4	0	3	3	0	3	0	91.224	91.224	0	91.224	0	1.55	1.55	0	1.55	0	49	49	0	49	0	4.33E-07	4.33E-07	0	4.33E-07	0	3800	3800	0	3800	0	23	23	0	23	0	2128	2128	0	2128	0	0	0	0	0	0	5.70E-06	5.70E-06	0	5.70E-06	0	23.1853	225	3.20068	3.20068	3.20068	1.5708	2.0944	2.0944	0.03969
2.2	2.2	0	2.2	0	6	6	0	6	0	8	8	0	8	0	3	3	0	3	0	190.23	190.23	0	190.23	0	1.3	1.3	0	1.3	0	63	63	0	63	0	8.10E-08	8.10E-08	0	8.10E-08	0	4940	4940	0	4940	0	88	88	0	88	0	3306	3306	0	3306	0	0	0	0	0	0	5.10E-06	5.10E-06	0	5.10E-06	0	14.3403	225	2.72704	2.72704	2.72704	1.5708	2.0944	2.0944	0.13649
1.65	1.91	0.26	1.78	0.18385	4	4	0	4	0	10	12	2	11	1.41421	3	3	0	3	0	58.6934	65.409	6.7156	62.0512	4.74865	1.35	1.35	0	1.35	0	67	76	9	71.5	6.36396	6.00E-08	7.20E-08	1.20E-08	6.60E-08	8.49E-09	3700	4970	1270	4335	898.026	91	120	29	105.5	20.5061	692.68	1728	1035.32	1210.34	732.082	70	180	110	125	77.7817	1.34E-05	3.02E-05	1.68E-05	2.18E-05	1.19E-05	23.9616	123	2.73529	2.73529	3.20266	1.5708	1.5708	1.5708	-0.2621
1.54	1.93	0.39	1.735	0.27577	4	5	1	4.5	0.70711	4	11	7	7.5	4.94975	3	3	0	3	0	47.867	107.868	60.0012	77.8676	42.4273	1.4	1.6	0.2	1.5	0.14142	51	71	20	61	14.1421	1.63E-08	4.00E-07	3.84E-07	2.08E-07	2.71E-07	2600	4140	1540	3370	1088.94	22	430	408	226	288.5	1234.93	1941	706.07	1587.97	499.267	100	110	10	105	7.07107	8.60E-06	1.89E-05	1.03E-05	1.37E-05	7.28E-06	33.9741	123	2.85812	2.85812	4.15899	1.5708	1.5708	1.5708	0.06953
1.65	1.91	0.26	1.78	0.18385	4	4	0	4	0	10	12	2	11	1.41421	3	3	0	3	0	58.6934	65.409	6.7156	62.0512	4.74865	1.35	1.35	0	1.35	0	67	76	9	71.5	6.36396	6.00E-08	7.20E-08	1.20E-08	6.60E-08	8.49E-09	3700	4970	1270	4335	898.026	91	120	29	105.5	20.5061	692.68	1728	1035.32	1210.34	732.082	70	180	110	125	77.7817	1.34E-05	3.02E-05	1.68E-05	2.18E-05	1.19E-05	24.0255	221	2.88552	2.88552	2.88552	1.5708	1.5708	1.5708	-0.2497
1.1	2.1	1	1.6	0.70711	5	8	3	6.5	2.12132	16	16	0	16	0	2	3	1	2.5	0.70711	127.6	173.04	45.44	150.32	32.1309	1.4	1.75	0.35	1.58	0.24749	17	92	75	54.5	53.033	2.50E-07	0.0001	9.98E-05	5.01E-05	7.05E-05	1590	2610	1020	2100	721.249	3	39	36	21	25.4558	722.66	1097	374.34	909.83	264.698	31	65	34	48	24.0416	0	0	0	0	0	63.9672	225	4.48908	4.48908	4.48908	1.5708	2.0944	2.0944	-1.6798
1.38	2.1	0.72	1.74	0.50912	5	9	4	7	2.82843	6	16	10	11	7.07107	2	3	1	2.5	0.70711	127.6	238.029	110.429	182.814	78.085	1.4	1.75	0.35	1.58	0.24749	45	92	47	68.5	33.234	2.80E-07	0.0001	9.97E-05	5.01E-05	7.05E-05	2610	3155	545	2882.5	385.373	3	27	24	15	16.9706	722.66	1405.3	682.64	1063.98	482.699	65	100	35	82.5	24.7487	0	0	0	0	0	52.5098	221	3.74467	3.74467	3.74467	1.5708	1.5708	1.5708	-0.4393
0.98	1.62	0.64	1.3	0.45255	2	6	4	4	2.82843	1	13	12	7	8.48528	1	2	1	1.5	0.70711	6.941	204.383	197.442	105.662	139.613	1.45	1.9	0.45	1.67	0.3182	12	78	66	45	46.669	9.50E-08	1.50E-07	5.50E-08	1.23E-07	3.89E-08	818	6000	5182	3409	3664.23	46	85	39	65.5	27.5772	453.69	577	123.31	515.345	87.1933	11	43	32	27	22.6274	2.99E-05	4.60E-05	1.61E-05	3.80E-05	1.14E-05	41.1753	221	3.45313	3.45313	3.45313	1.5708	1.5708	1.5708	-0.2332
1.9	2.55	0.65	2.225	0.45962	2	3	1	2.5	0.70711	14	14	0	14	0	2	2	0	2	0	12.0107	28.0855	16.0748	20.0481	11.3666	0.7	1.1	0.4	0.9	0.28284	85	95	10	90	7.07107	1.00E-05	0.001	0.00099	0.00051	0.0007	2200	18350	16150	10275	11419.8	140	150	10	145	7.07107	1687	3800	2113	2743.5	1494.12	33	100	67	66.5	47.3762	2.60E-06	7.10E-06	4.50E-06	4.85E-06	3.18E-06	20.8806	216	3.09091	3.09091	3.09091	1.5708	2.0944	2.0944	-0.2082
1.2	1.2	0	1.2	0	8	8	0	8	0	9	9	0	9	0	3	3	0	3	0	151.964	151.964	0	151.964	0	1.85	1.85	0	1.85	0	18	18	0	18	0	9.00E-07	9.00E-07	0	9.00E-07	0	0	0	0	0	0	14	14	0	14	0	1099	1099	0	1099	0	8.3	8.3	0	8.3	0	3.50E-05	3.50E-05	0	3.50E-05	0	40.3709	229	3.74309	3.74309	3.74309	1.91063	1.91063	1.91063	0
2.05	2.05	0	2.05	0	5	5	0	5	0	15	15	0	15	0	2	2	0	2	0	121.76	121.76	0	121.76	0	1.45	1.45	0	1.45	0	88	88	0	88	0	4.00E-07	4.00E-07	0	4.00E-07	0	3420	3420	0	3420	0	24	24	0	24	0	903.78	903.78	0	903.78	0	42	42	0	42	0	1.10E-05	1.10E-05	0	1.10E-05	0	30.0215	221	3.10797	3.10797	3.10797	1.5708	1.5708	1.5708	0.04331
1.1	1.1	0	1.1	0	8	8	0	8	0	16	16	0	16	0	3	3	0	3	0	173.04	173.04	0	173.04	0	1.75	1.75	0	1.75	0	17	17	0	17	0	2.50E-07	2.50E-07	0	2.50E-07	0	1590	1590	0	1590	0	39	39	0	39	0	1097	1097	0	1097	0	31	31	0	31	0	2.63E-05	2.63E-05	0	2.63E-05	0	36.8399	225	3.73489	3.73489	3.73489	1.5708	2.0944	2.0944	0
1.2	1.2	0	1.2	0	8	8	0	8	0	10	10	0	10	0	3	3	0	3	0	157.25	157.25	0	157.25	0	1.8	1.8	0	1.8	0	27	27	0	27	0	1.31E-06	1.31E-06	0	1.31E-06	0	2680	2680	0	2680	0	11	11	0	11	0	1585	1585	0	1585	0	38	38	0	38	0	9.40E-06	9.40E-06	0	9.40E-06	0	32.4096	225	3.57874	3.57874	3.57874	1.5708	2.0944	2.0944	0.01129
1.5	3.04	1.54	2.27	1.08894	2	6	4	4	2.82843	5	15	10	10	7.07107	2	3	1	2.5	0.70711	14.0067	180.948	166.941	97.4773	118.045	0.65	1.45	0.8	1.05	0.56569	52	100	48	76	33.9411	0	0	0	0	0	333.6	3400	3066.4	1866.8	2168.27	0.02583	57	56.9742	28.5129	40.2868	63.05	3290	3226.95	1676.53	2281.8	0	0	0	0	0	0	0	0	0	0	21.7021	187	2.94299	2.943	2.89329	1.5708	1.5708	2.09439	-1.2539
1.24	2.2	0.96	1.72	0.67882	6	8	2	7	1.41421	9	14	5	11.5	3.53553	3	3	0	3	0	167.259	192.217	24.958	179.738	17.648	1.35	1.75	0.4	1.55	0.28284	22	66	44	44	31.1127	4.70E-08	8.60E-07	8.13E-07	4.54E-07	5.75E-07	2830	4825	1995	3827.5	1410.68	15	150	135	82.5	95.4594	1802	2739	937	2270.5	662.559	44	320	276	182	195.161	6.40E-06	1.22E-05	5.80E-06	9.30E-06	4.10E-06	38.8199	221	3.38598	3.38598	3.38598	1.5708	1.5708	1.5708	-0.8872
1.25	1.25	0	1.25	0	8	8	0	8	0	15	15	0	15	0	3	3	0	3	0	168.934	168.934	0	168.934	0	1.75	1.75	0	1.75	0	21	21	0	21	0	6.76E-07	6.76E-07	0	6.76E-07	0	0	0	0	0	0	17	17	0	17	0	1818	1818	0	1818	0	45	45	0	45	0	1.33E-05	1.33E-05	0	1.33E-05	0	59.4921	194	3.53328	3.5332	5.50283	1.5708	1.5708	2.09441	0.00105
1	2.55	1.55	1.775	1.09602	4	4	0	4	0	2	16	14	9	9.89949	1	2	1	1.5	0.70711	40.078	78.96	38.882	59.519	27.4937	1.15	1.8	0.65	1.48	0.45962	16	93	77	54.5	54.4472	1.00E-08	3.40E-08	2.40E-08	2.20E-08	1.70E-08	3350	3810	460	3580	325.269	0.52	200	199.48	100.26	141.054	494	1115	621	804.5	439.113	8.3	17	8.7	12.65	6.15183	0	0	0	0	0	52.3408	225	4.19873	4.19873	4.19873	1.5708	2.0944	2.0944	-2.0436
1	2.1	1.1	1.55	0.77782	4	5	1	4.5	0.70711	2	16	14	9	9.89949	1	2	1	1.5	0.70711	40.078	127.6	87.522	83.839	61.8874	1.4	1.8	0.4	1.6	0.28284	16	92	76	54	53.7401	3.40E-08	0.0001	1.00E-04	5.00E-05	7.07E-05	2610	3810	1200	3210	848.528	3	200	197	101.5	139.3	722.66	1115	392.34	918.83	277.426	17	65	48	41	33.9411	0	0	0	0	0	65.2324	225	4.51848	4.51848	4.51848	1.5708	2.0944	2.0944	-1.6607
1.54	1.54	0	1.54	0	4	4	0	4	0	4	4	0	4	0	3	3	0	3	0	47.867	47.867	0	47.867	0	1.4	1.4	0	1.4	0	51	51	0	51	0	4.00E-07	4.00E-07	0	4.00E-07	0	4140	4140	0	4140	0	22	22	0	22	0	1941	1941	0	1941	0	110	110	0	110	0	8.60E-06	8.60E-06	0	8.60E-06	0	16.8486	229	2.79723	2.79723	2.79723	1.91063	1.91063	1.91063	0.11433
0	0	0	0	0	2	2	0	2	0	18	18	0	18	0	2	2	0	2	0	20.1797	20.1797	0	20.1797	0	0	0	0	0	0	2	2	0	2	0	0	0	0	0	0	936	936	0	936	0	0.0491	0.0491	0	0.0491	0	24.56	24.56	0	24.56	0	0	0	0	0	0	0	0	0	0	0	10.1371	225	2.42928	2.42928	2.42928	1.5708	2.0944	2.0944	0.32008
1.24	1.31	0.07	1.275	0.0495	3	8	5	5.5	3.53553	2	14	12	8	8.48528	1	3	2	2	1.41421	24.305	167.259	142.954	95.782	101.084	1.5	1.75	0.25	1.63	0.17678	22	73	51	47.5	36.0624	4.40E-08	8.60E-07	8.16E-07	4.52E-07	5.77E-07	2830	4602	1772	3716	1252.99	15	160	145	87.5	102.53	923	1802	879	1362.5	621.547	44	45	1	44.5	0.70711	8.20E-06	1.22E-05	4.00E-06	1.02E-05	2.83E-06	53.2562	221	3.76233	3.76233	3.76233	1.5708	1.5708	1.5708	-0.0666
0.98	0.98	0	0.98	0	2	2	0	2	0	1	1	0	1	0	1	1	0	1	0	6.941	6.941	0	6.941	0	1.45	1.45	0	1.45	0	12	12	0	12	0	9.50E-08	9.50E-08	0	9.50E-08	0	6000	6000	0	6000	0	85	85	0	85	0	453.69	453.69	0	453.69	0	11	11	0	11	0	4.60E-05	4.60E-05	0	4.60E-05	0	18.0942	225	2.94681	2.94681	2.94681	1.5708	2.0944	2.0944	0.00102
2.19	2.19	0	2.19	0	3	3	0	3	0	15	15	0	15	0	2	2	0	2	0	30.9738	30.9738	0	30.9738	0	1	1	0	1	0	90	90	0	90	0	1.00E-07	1.00E-07	0	1.00E-07	0	0	0	0	0	0	0.236	0.236	0	0.236	0	317.3	317.3	0	317.3	0	11	11	0	11	0	0	0	0	0	0	14.5944	65	2.44382	2.44382	2.44382	1.5708	1.56132	1.5708	0.13254
0.93	0.93	0	0.93	0	3	3	0	3	0	1	1	0	1	0	1	1	0	1	0	22.9898	22.9898	0	22.9898	0	1.8	1.8	0	1.8	0	11	11	0	11	0	4.90E-08	4.90E-08	0	4.90E-08	0	3200	3200	0	3200	0	140	140	0	140	0	370.87	370.87	0	370.87	0	6.3	6.3	0	6.3	0	7.10E-05	7.10E-05	0	7.10E-05	0	33.1271	229	3.5043	3.5043	3.5043	1.91063	1.91063	1.91063	0.00305
1.83	1.83	0	1.83	0	4	4	0	4	0	8	8	0	8	0	3	3	0	3	0	55.845	55.845	0	55.845	0	1.4	1.4	0	1.4	0	61	61	0	61	0	1.00E-07	1.00E-07	0	1.00E-07	0	4910	4910	0	4910	0	80	80	0	80	0	1811	1811	0	1811	0	170	170	0	170	0	1.18E-05	1.18E-05	0	1.18E-05	0	11.1914	229	2.44063	2.44063	2.44063	1.91063	1.91063	1.91063	0
1.36	1.36	0	1.36	0	9	9	0	9	0	7	7	0	7	0	3	3	0	3	0	237	237	0	237	0	1.75	1.75	0	1.75	0	44	44	0	44	0	1.20E-06	1.20E-06	0	1.20E-06	0	0	0	0	0	0	6	6	0	6	0	910	910	0	910	0	0	0	0	0	0	0	0	0	0	0	17.567	229	2.83644	2.83644	2.83644	1.91063	1.91063	1.91063	0.44372
1.31	1.31	0	1.31	0	3	3	0	3	0	2	2	0	2	0	1	1	0	1	0	24.305	24.305	0	24.305	0	1.5	1.5	0	1.5	0	73	73	0	73	0	4.40E-08	4.40E-08	0	4.40E-08	0	4602	4602	0	4602	0	160	160	0	160	0	923	923	0	923	0	45	45	0	45	0	8.20E-06	8.20E-06	0	8.20E-06	0	22.8362	229	3.09563	3.09563	3.09563	1.91063	1.91063	1.91063	0.02883
1.55	2.54	0.99	2.045	0.70004	4	6	2	5	1.41421	7	11	4	9	2.82843	3	3	0	3	0	54.938	196.967	142.029	125.952	100.429	1.35	1.4	0.05	1.38	0.03536	60	70	10	65	7.07107	2.20E-08	1.44E-06	1.42E-06	7.31E-07	1.00E-06	1740	5150	3410	3445	2411.23	7.8	320	312.2	163.9	220.759	1337.33	1519	181.67	1428.17	128.46	120	220	100	170	70.7107	1.42E-05	2.17E-05	7.50E-06	1.80E-05	5.30E-06	33.1826	123	3.24611	3.24611	3.14909	1.5708	1.5708	1.5708	-0.0129
1.63	1.83	0.2	1.73	0.14142	4	4	0	4	0	5	8	3	6.5	2.12132	3	3	0	3	0	50.9415	55.845	4.9035	53.3932	3.4673	1.35	1.4	0.05	1.38	0.03536	54	61	7	57.5	4.94975	1.00E-07	2.00E-07	1.00E-07	1.50E-07	7.07E-08	4560	4910	350	4735	247.487	31	80	49	55.5	34.6482	1811	2183	372	1997	263.044	160	170	10	165	7.07107	8.40E-06	1.18E-05	3.40E-06	1.01E-05	2.40E-06	23.5689	221	2.86712	2.86712	2.86712	1.5708	1.5708	1.5708	-0.1242
1.5	2.2	0.7	1.85	0.49497	5	6	1	5.5	0.70711	5	8	3	6.5	2.12132	3	3	0	3	0	101.07	180.948	79.8779	141.009	56.4822	1.3	1.45	0.15	1.38	0.10607	52	62	10	57	7.07107	7.10E-08	1.35E-07	6.40E-08	1.03E-07	4.53E-08	3400	5970	2570	4685	1817.26	57	120	63	88.5	44.5477	2607	3290	683	2948.5	482.954	200	220	20	210	14.1421	6.30E-06	6.40E-06	1.00E-07	6.35E-06	7.07E-08	31.9855	221	3.17432	3.17432	3.17432	1.5708	1.5708	1.5708	-0.283
2.01	2.01	0	2.01	0	4	4	0	4	0	14	14	0	14	0	2	2	0	2	0	72.64	72.64	0	72.64	0	1.25	1.25	0	1.25	0	84	84	0	84	0	0.0005	0.0005	0	0.0005	0	5400	5400	0	5400	0	60	60	0	60	0	1211.4	1211.4	0	1211.4	0	0	0	0	0	0	6.00E-06	6.00E-06	0	6.00E-06	0	19.093	229	2.9163	2.9163	2.9163	1.91063	1.91063	1.91063	0.34355
2.05	2.05	0	2.05	0	5	5	0	5	0	15	15	0	15	0	2	2	0	2	0	121.76	121.76	0	121.76	0	1.45	1.45	0	1.45	0	88	88	0	88	0	4.00E-07	4.00E-07	0	4.00E-07	0	3420	3420	0	3420	0	24	24	0	24	0	903.78	903.78	0	903.78	0	42	42	0	42	0	1.10E-05	1.10E-05	0	1.10E-05	0	27.1573	229	3.27971	3.27971	3.27971	1.91063	1.91063	1.91063	0.2276
1.25	1.25	0	1.25	0	8	8	0	8	0	15	15	0	15	0	3	3	0	3	0	168.934	168.934	0	168.934	0	1.75	1.75	0	1.75	0	21	21	0	21	0	6.76E-07	6.76E-07	0	6.76E-07	0	0	0	0	0	0	17	17	0	17	0	1818	1818	0	1818	0	45	45	0	45	0	1.33E-05	1.33E-05	0	1.33E-05	0	29.6251	225	3.47316	3.47316	3.47316	1.5708	2.0944	2.0944	0.03178
2.2	2.2	0	2.2	0	1	1	0	1	0	1	1	0	1	0	1	1	0	1	0	1.00794	1.00794	0	1.00794	0	0.25	0.25	0	0.25	0	103	103	0	103	0	0	0	0	0	0	1270	1270	0	1270	0	0.1805	0.1805	0	0.1805	0	14.01	14.01	0	14.01	0	0	0	0	0	0	0	0	0	0	0	2.72968	225	1.56871	1.56871	1.56871	1.5708	2.0944	2.0944	1.18766
2.2	2.2	0	2.2	0	1	1	0	1	0	1	1	0	1	0	1	1	0	1	0	1.00794	1.00794	0	1.00794	0	0.25	0.25	0	0.25	0	103	103	0	103	0	0	0	0	0	0	1270	1270	0	1270	0	0.1805	0.1805	0	0.1805	0	14.01	14.01	0	14.01	0	0	0	0	0	0	0	0	0	0	0	2.73279	229	1.52549	1.52549	1.52549	1.91063	1.91063	1.91063	1.19774
1.9	2.04	0.14	1.97	0.09899	2	5	3	3.5	2.12132	7	13	6	10	4.24264	2	3	1	2.5	0.70711	10.811	98	87.189	54.4055	61.6519	0.85	1.35	0.5	1.1	0.35355	59	86	27	72.5	19.0919	2.20E-07	1E+12	1E+12	5E+11	7.1E+11	0	0	0	0	0	27	51	24	39	16.9706	2349	2430	81	2389.5	57.2756	0	0	0	0	0	0	0	0	0	0	21.0997	187	2.88178	2.88183	2.93369	1.5708	1.5708	2.09439	-0.2825
1.23	2.18	0.95	1.705	0.67175	4	8	4	6	2.82843	13	15	2	14	1.41421	2	3	1	2.5	0.70711	74.9216	164.93	90.0087	119.926	63.6458	1.15	1.75	0.6	1.45	0.42426	23	89	66	56	46.669	3.30E-07	8.14E-07	4.84E-07	5.72E-07	3.42E-07	0	0	0	0	0	16	50	34	33	24.0416	1090	1734	644	1412	455.377	22	40	18	31	12.7279	0	0	0	0	0	48.5046	225	4.09354	4.09354	4.09354	1.5708	2.0944	2.0944	-1.5281
1.9	2.04	0.14	1.97	0.09899	2	5	3	3.5	2.12132	7	13	6	10	4.24264	2	3	1	2.5	0.70711	10.811	98	87.189	54.4055	61.6519	0.85	1.35	0.5	1.1	0.35355	59	86	27	72.5	19.0919	2.20E-07	1E+12	1E+12	5E+11	7.1E+11	0	0	0	0	0	27	51	24	39	16.9706	2349	2430	81	2389.5	57.2756	0	0	0	0	0	0	0	0	0	0	26.3891	216	3.3418	3.3418	3.3418	1.5708	2.0944	2.0944	0.71886
1.23	2.55	1.32	1.89	0.93338	4	8	4	6	2.82843	13	16	3	14.5	2.12132	2	3	1	2.5	0.70711	78.96	164.93	85.9703	121.945	60.7902	1.15	1.75	0.6	1.45	0.42426	23	93	70	58	49.4975	1.00E-08	8.14E-07	8.04E-07	4.12E-07	5.69E-07	2760	3350	590	3055	417.193	0.52	16	15.48	8.26	10.946	494	1734	1240	1114	876.812	8.3	40	31.7	24.15	22.4153	0	0	0	0	0	46.7289	225	4.04297	4.04297	4.04297	1.5708	2.0944	2.0944	-1.7219
1.27	2.2	0.93	1.735	0.65761	5	8	3	6.5	2.12132	10	17	7	13.5	4.94975	3	3	0	3	0	106.42	174.967	68.547	140.694	48.47	1.4	1.75	0.35	1.58	0.24749	20	69	49	44.5	34.6482	1.08E-07	5.80E-07	4.72E-07	3.44E-07	3.34E-07	0	0	0	0	0	16	72	56	44	39.598	1828.05	1925	96.95	1876.53	68.554	48	180	132	114	93.3381	9.90E-06	1.18E-05	1.90E-06	1.09E-05	1.34E-06	40.1355	221	3.42381	3.42381	3.42381	1.5708	1.5708	1.5708	-0.9718
1.27	2.28	1.01	1.775	0.71418	5	8	3	6.5	2.12132	9	17	8	13	5.65685	3	3	0	3	0	102.906	174.967	72.0615	138.936	50.9552	1.35	1.75	0.4	1.55	0.28284	20	65	45	42.5	31.8198	4.30E-08	5.80E-07	5.37E-07	3.12E-07	3.80E-07	0	0	0	0	0	16	150	134	83	94.7523	1925	2237	312	2081	220.617	48	380	332	214	234.759	8.20E-06	9.90E-06	1.70E-06	9.05E-06	1.20E-06	37.3813	221	3.34363	3.34363	3.34363	1.5708	1.5708	1.5708	-1.0133
1.27	2.2	0.93	1.735	0.65761	5	8	3	6.5	2.12132	8	17	9	12.5	6.36396	3	3	0	3	0	101.07	174.967	73.897	138.019	52.2531	1.3	1.75	0.45	1.53	0.3182	20	62	42	41	29.6985	7.10E-08	5.80E-07	5.09E-07	3.26E-07	3.60E-07	0	0	0	0	0	16	120	104	68	73.5391	1925	2607	682	2266	482.247	48	220	172	134	121.622	6.40E-06	9.90E-06	3.50E-06	8.15E-06	2.47E-06	36.7729	221	3.32539	3.32539	3.32539	1.5708	1.5708	1.5708	-0.4507
0.95	1.62	0.67	1.285	0.47376	5	6	1	5.5	0.70711	2	13	11	7.5	7.77817	1	2	1	1.5	0.70711	87.62	204.383	116.763	146.002	82.5641	1.9	2	0.1	1.95	0.07071	15	78	63	46.5	44.5477	1.35E-07	1.50E-07	1.50E-08	1.43E-07	1.06E-08	0	0	0	0	0	35	46	11	40.5	7.77817	577	1050	473	813.5	334.462	0	0	0	0	0	2.25E-05	2.99E-05	7.40E-06	2.62E-05	5.23E-06	66.9283	221	4.0601	4.0601	4.0601	1.5708	1.5708	1.5708	-0.428
1.2	1.2	0	1.2	0	8	8	0	8	0	10	10	0	10	0	3	3	0	3	0	157.25	157.25	0	157.25	0	1.8	1.8	0	1.8	0	27	27	0	27	0	1.31E-06	1.31E-06	0	1.31E-06	0	2680	2680	0	2680	0	11	11	0	11	0	1585	1585	0	1585	0	38	38	0	38	0	9.40E-06	9.40E-06	0	9.40E-06	0	65.0286	194	3.62027	3.62019	5.72934	1.5708	1.5708	2.09441	0.01706
1.27	1.65	0.38	1.46	0.2687	4	8	4	6	2.82843	12	17	5	14.5	3.53553	3	3	0	3	0	65.409	174.967	109.558	120.188	77.4692	1.35	1.75	0.4	1.55	0.28284	20	76	56	48	39.598	6.00E-08	5.80E-07	5.20E-07	3.20E-07	3.68E-07	0	0	0	0	0	16	120	104	68	73.5391	692.68	1925	1232.32	1308.84	871.382	48	70	22	59	15.5563	9.90E-06	3.02E-05	2.03E-05	2.01E-05	1.44E-05	42.2691	221	3.48344	3.48344	3.48344	1.5708	1.5708	1.5708	-0.3552
1.14	1.31	0.17	1.225	0.12021	3	8	5	5.5	3.53553	2	6	4	4	2.82843	1	3	2	2	1.41421	24.305	144.242	119.937	84.2735	84.8083	1.5	1.85	0.35	1.68	0.24749	30	73	43	51.5	30.4056	4.40E-08	6.43E-07	5.99E-07	3.44E-07	4.24E-07	2330	4602	2272	3466	1606.55	17	160	143	88.5	101.116	923	1297	374	1110	264.458	32	45	13	38.5	9.19239	8.20E-06	9.60E-06	1.40E-06	8.90E-06	9.90E-07	58.4562	221	3.881	3.881	3.881	1.5708	1.5708	1.5708	-0.1149
1.23	1.23	0	1.23	0	8	8	0	8	0	13	13	0	13	0	3	3	0	3	0	164.93	164.93	0	164.93	0	1.75	1.75	0	1.75	0	23	23	0	23	0	8.14E-07	8.14E-07	0	8.14E-07	0	2760	2760	0	2760	0	16	16	0	16	0	1734	1734	0	1734	0	40	40	0	40	0	1.12E-05	1.12E-05	0	1.12E-05	0	62.0836	194	3.58581	3.5857	5.57557	1.5708	1.5708	2.09441	0.00295
1.54	1.83	0.29	1.685	0.20506	4	4	0	4	0	4	8	4	6	2.82843	3	3	0	3	0	47.867	55.845	7.978	51.856	5.6413	1.4	1.4	0	1.4	0	51	61	10	56	7.07107	1.00E-07	4.00E-07	3.00E-07	2.50E-07	2.12E-07	4140	4910	770	4525	544.472	22	80	58	51	41.0122	1811	1941	130	1876	91.9239	110	170	60	140	42.4264	8.60E-06	1.18E-05	3.20E-06	1.02E-05	2.26E-06	25.5024	221	2.94348	2.94348	2.94348	1.5708	1.5708	1.5708	-0.4492
1.36	3.04	1.68	2.2	1.18794	2	4	2	3	1.41421	3	15	12	9	8.48528	2	3	1	2.5	0.70711	14.0067	44.9559	30.9492	29.4813	21.8844	0.65	1.6	0.95	1.13	0.67175	19	100	81	59.5	57.2756	0	0	0	0	0	0	0	0	0	0	0.02583	16	15.9742	8.01292	11.2954	63.05	1814	1750.95	938.525	1238.11	0	0	0	0	0	0	0	0	0	0	21.3903	221	2.77591	2.77591	2.77591	1.5708	1.5708	1.5708	-0.9885
1.1	1.1	0	1.1	0	8	8	0	8	0	11	11	0	11	0	3	3	0	3	0	158.925	158.925	0	158.925	0	1.75	1.75	0	1.75	0	26	26	0	26	0	1.15E-06	1.15E-06	0	1.15E-06	0	2620	2620	0	2620	0	11	11	0	11	0	1629	1629	0	1629	0	38.7	38.7	0	38.7	0	1.03E-05	1.03E-05	0	1.03E-05	0	64.4323	194	3.61792	3.61781	5.68424	1.5708	1.5708	2.09441	0.01226
1.1	1.96	0.86	1.53	0.60811	5	8	3	6.5	2.12132	14	16	2	15	1.41421	2	3	1	2.5	0.70711	118.71	173.04	54.33	145.875	38.4171	1.45	1.75	0.3	1.6	0.21213	17	83	66	50	46.669	1.15E-07	2.50E-07	1.35E-07	1.83E-07	9.55E-08	1590	2500	910	2045	643.467	39	67	28	53	19.799	505.08	1097	591.92	801.04	418.551	31	58	27	44.5	19.0919	2.20E-05	2.63E-05	4.30E-06	2.41E-05	3.04E-06	53.8951	123	3.45234	3.45234	4.52192	1.5708	1.5708	1.5708	-0.7203
1.63	2.2	0.57	1.915	0.40305	4	6	2	5	1.41421	5	8	3	6.5	2.12132	3	3	0	3	0	50.9415	190.23	139.289	120.586	98.4918	1.3	1.35	0.05	1.33	0.03536	54	63	9	58.5	6.36396	8.10E-08	2.00E-07	1.19E-07	1.41E-07	8.41E-08	4560	4940	380	4750	268.701	31	88	57	59.5	40.3051	2183	3306	1123	2744.5	794.081	0	0	0	0	0	5.10E-06	8.40E-06	3.30E-06	6.75E-06	2.33E-06	27.2522	221	3.00931	3.00931	3.00931	1.5708	1.5708	1.5708	-0.2263
1.36	2.19	0.83	1.775	0.5869	3	4	1	3.5	0.70711	3	15	12	9	8.48528	2	3	1	2.5	0.70711	30.9738	44.9559	13.9821	37.9648	9.88687	1	1.6	0.6	1.3	0.42426	19	90	71	54.5	50.2046	1.00E-07	5.50E-07	4.50E-07	3.25E-07	3.18E-07	0	0	0	0	0	0.236	16	15.764	8.118	11.1468	317.3	1814	1496.7	1065.65	1058.33	11	57	46	34	32.5269	0	0	0	0	0	35.1253	221	3.27497	3.27497	3.27497	1.5708	1.5708	1.5708	-1.0565
1.69	2.54	0.85	2.115	0.60104	5	6	1	5.5	0.70711	11	12	1	11.5	0.70711	3	3	0	3	0	112.411	196.967	84.5556	154.689	59.7898	1.35	1.55	0.2	1.45	0.14142	70	75	5	72.5	3.53553	2.20E-08	7.00E-08	4.80E-08	4.60E-08	3.39E-08	1740	2310	570	2025	403.051	97	320	223	208.5	157.685	594.22	1337.33	743.11	965.775	525.458	42	220	178	131	125.865	1.42E-05	3.08E-05	1.66E-05	2.25E-05	1.17E-05	38.3287	221	3.37164	3.37164	3.37164	1.5708	1.5708	1.5708	-0.1799
1.5	1.9	0.4	1.7	0.28284	5	6	1	5.5	0.70711	5	7	2	6	1.41421	3	3	0	3	0	98	180.948	82.9479	139.474	58.653	1.35	1.45	0.1	1.4	0.07071	52	59	7	55.5	4.94975	1.35E-07	2.20E-07	8.50E-08	1.78E-07	6.01E-08	0	0	0	0	0	51	57	6	54	4.24264	2430	3290	860	2860	608.112	0	0	0	0	0	0	0	0	0	0	32.2914	221	3.18441	3.18441	3.18441	1.5708	1.5708	1.5708	-0.501
1.54	1.61	0.07	1.575	0.0495	3	4	1	3.5	0.70711	4	13	9	8.5	6.36396	2	3	1	2.5	0.70711	26.9815	47.867	20.8855	37.4243	14.7683	1.25	1.4	0.15	1.33	0.10607	51	80	29	65.5	20.5061	2.70E-08	4.00E-07	3.73E-07	2.14E-07	2.64E-07	4140	5100	960	4620	678.823	22	235	213	128.5	150.614	933.47	1941	1007.53	1437.24	712.431	76	110	34	93	24.0416	8.60E-06	2.31E-05	1.45E-05	1.59E-05	1.03E-05	32.2471	123	2.81862	2.81862	4.059	1.5708	1.5708	1.5708	-0.436
1.1	1.62	0.52	1.36	0.3677	6	8	2	7	1.41421	11	13	2	12	1.41421	2	3	1	2.5	0.70711	158.925	204.383	45.4579	181.654	32.1436	1.75	1.9	0.15	1.83	0.10607	26	78	52	52	36.7696	1.50E-07	1.15E-06	1.00E-06	6.50E-07	7.07E-07	818	2620	1802	1719	1274.21	11	46	35	28.5	24.7487	577	1629	1052	1103	743.876	38.7	43	4.3	40.85	3.04056	1.03E-05	2.99E-05	1.96E-05	2.01E-05	1.39E-05	54.1523	221	3.78331	3.78331	3.78331	1.5708	1.5708	1.5708	-0.0232
1.54	2	0.46	1.77	0.32527	4	6	2	5	1.41421	4	12	8	8	5.65685	3	3	0	3	0	47.867	200.59	152.723	124.229	107.991	1.4	1.5	0.1	1.45	0.07071	51	74	23	62.5	16.2635	4.00E-07	9.60E-07	5.60E-07	6.80E-07	3.96E-07	1407	4140	2733	2773.5	1932.52	8.3	22	13.7	15.15	9.68736	234.32	1941	1706.68	1087.66	1206.81	25	110	85	67.5	60.1041	0	0	0	0	0	37.2046	123	3.02653	3.02653	4.06169	1.5708	1.5708	1.5708	-0.0072
1.31	2.2	0.89	1.755	0.62933	3	5	2	4	1.41421	2	10	8	6	5.65685	1	3	2	2	1.41421	24.305	106.42	82.115	65.3625	58.0641	1.4	1.5	0.1	1.45	0.07071	69	73	4	71	2.82843	4.40E-08	1.08E-07	6.40E-08	7.60E-08	4.53E-08	3070	4602	1532	3836	1083.29	72	160	88	116	62.2254	923	1828.05	905.05	1375.53	639.967	45	180	135	112.5	95.4594	8.20E-06	1.18E-05	3.60E-06	1.00E-05	2.55E-06	31.5558	221	3.16005	3.16005	3.16005	1.5708	1.5708	1.5708	-0.7445
0.79	2.54	1.75	1.665	1.23744	6	6	0	6	0	1	11	10	6	7.07107	1	3	2	2	1.41421	132.905	196.967	64.0611	164.936	45.2981	1.35	2.6	1.25	1.98	0.88388	8	70	62	39	43.8406	2.20E-08	2.10E-07	1.88E-07	1.16E-07	1.33E-07	0	0	0	0	0	36	320	284	178	200.818	301.59	1337.33	1035.74	819.46	732.379	1.6	220	218.4	110.8	154.432	0	0	0	0	0	77.4177	221	4.262	4.262	4.262	1.5708	1.5708	1.5708	-0.3204
0.82	2.54	1.72	1.68	1.21622	5	6	1	5.5	0.70711	1	11	10	6	7.07107	1	3	2	2	1.41421	85.4678	196.967	111.499	141.217	78.8415	1.35	2.35	1	1.85	0.70711	9	70	61	39.5	43.1335	2.20E-08	1.33E-07	1.11E-07	7.75E-08	7.85E-08	1300	1740	440	1520	311.127	58	320	262	189	185.262	312.46	1337.33	1024.87	824.895	724.693	2.5	220	217.5	111.25	153.796	0	0	0	0	0	69.1706	221	4.10494	4.10494	4.10494	1.5708	1.5708	1.5708	-0.2769
1.13	1.31	0.18	1.22	0.12728	3	8	5	5.5	3.53553	2	5	3	3.5	2.12132	1	3	2	2	1.41421	24.305	140.908	116.603	82.6063	82.4505	1.5	1.85	0.35	1.68	0.24749	31	73	42	52	29.6985	4.40E-08	7.00E-07	6.56E-07	3.72E-07	4.64E-07	2280	4602	2322	3441	1641.9	13	160	147	86.5	103.945	923	1208	285	1065.5	201.525	29	45	16	37	11.3137	6.70E-06	8.20E-06	1.50E-06	7.45E-06	1.06E-06	60.037	221	3.91567	3.91567	3.91567	1.5708	1.5708	1.5708	-0.1194
1.1	1.65	0.55	1.375	0.38891	4	8	4	6	2.82843	11	12	1	11.5	0.70711	3	3	0	3	0	65.409	158.925	93.5164	112.167	66.126	1.35	1.75	0.4	1.55	0.28284	26	76	50	51	35.3553	6.00E-08	1.15E-06	1.09E-06	6.05E-07	7.71E-07	2620	3700	1080	3160	763.675	11	120	109	65.5	77.0746	692.68	1629	936.32	1160.84	662.078	38.7	70	31.3	54.35	22.1324	1.03E-05	3.02E-05	1.99E-05	2.02E-05	1.41E-05	45.5898	123	3.57684	3.57684	3.56345	1.5708	1.5708	1.5708	-0.012
1.22	2.54	1.32	1.88	0.93338	6	8	2	7	1.41421	11	12	1	11.5	0.70711	3	3	0	3	0	162.5	196.967	34.4666	179.733	24.3715	1.35	1.75	0.4	1.55	0.28284	24	70	46	47	32.5269	2.20E-08	9.26E-07	9.04E-07	4.74E-07	6.39E-07	1740	2710	970	2225	685.894	11	320	309	165.5	218.496	1337.33	1680	342.67	1508.67	242.304	41	220	179	130.5	126.572	9.90E-06	1.42E-05	4.30E-06	1.21E-05	3.04E-06	45.9144	221	3.58082	3.58082	3.58082	1.5708	1.5708	1.5708	-0.8646
1	1.69	0.69	1.345	0.4879	4	5	1	4.5	0.70711	2	12	10	7	7.07107	1	3	2	2	1.41421	40.078	112.411	72.333	76.2445	51.1472	1.55	1.8	0.25	1.68	0.17678	16	75	59	45.5	41.7193	3.40E-08	7.00E-08	3.60E-08	5.20E-08	2.55E-08	2310	3810	1500	3060	1060.66	97	200	103	148.5	72.832	594.22	1115	520.78	854.61	368.247	17	42	25	29.5	17.6777	2.23E-05	3.08E-05	8.50E-06	2.66E-05	6.01E-06	55.8254	221	3.82188	3.82188	3.82188	1.5708	1.5708	1.5708	-0.3736
1.83	2.2	0.37	2.015	0.26163	4	5	1	4.5	0.70711	8	10	2	9	1.41421	3	3	0	3	0	55.845	106.42	50.575	81.1325	35.7619	1.4	1.4	0	1.4	0	61	69	8	65	5.65685	1.00E-07	1.08E-07	8.00E-09	1.04E-07	5.66E-09	3070	4910	1840	3990	1301.08	72	80	8	76	5.65685	1811	1828.05	17.05	1819.53	12.0562	170	180	10	175	7.07107	1.18E-05	1.18E-05	0	1.18E-05	0	27.5263	123	2.70864	2.70864	3.75184	1.5708	1.5708	1.5708	-0.0658
1.3	2.2	0.9	1.75	0.6364	5	6	1	5.5	0.70711	4	8	4	6	2.82843	3	3	0	3	0	101.07	178.49	77.42	139.78	54.7442	1.3	1.55	0.25	1.43	0.17678	50	62	12	56	8.48528	7.10E-08	3.40E-07	2.69E-07	2.05E-07	1.90E-07	3010	5970	2960	4490	2093.04	23	120	97	71.5	68.5894	2506	2607	101	2556.5	71.4178	110	220	110	165	77.7817	5.90E-06	6.40E-06	5.00E-07	6.15E-06	3.54E-07	33.9267	221	3.23728	3.23728	3.23728	1.5708	1.5708	1.5708	-0.8125
1.1	2.05	0.95	1.575	0.67175	5	8	3	6.5	2.12132	11	15	4	13	2.82843	2	3	1	2.5	0.70711	121.76	158.925	37.1654	140.343	26.2799	1.45	1.75	0.3	1.6	0.21213	26	88	62	57	43.8406	4.00E-07	1.15E-06	7.50E-07	7.75E-07	5.30E-07	2620	3420	800	3020	565.685	11	24	13	17.5	9.19239	903.78	1629	725.22	1266.39	512.808	38.7	42	3.3	40.35	2.33345	1.03E-05	1.10E-05	7.00E-07	1.07E-05	4.95E-07	60.1284	225	4.39742	4.39742	4.39742	1.5708	2.0944	2.0944	-0.8222
1.12	1.31	0.19	1.215	0.13435	3	8	5	5.5	3.53553	2	4	2	3	1.41421	1	3	2	2	1.41421	24.305	140.116	115.811	82.2105	81.8907	1.5	1.85	0.35	1.68	0.24749	32	73	41	52.5	28.9914	4.40E-08	7.40E-07	6.96E-07	3.92E-07	4.92E-07	2100	4602	2502	3351	1769.18	11	160	149	85.5	105.359	923	1068	145	995.5	102.53	22	45	23	33.5	16.2635	6.30E-06	8.20E-06	1.90E-06	7.25E-06	1.34E-06	61.163	221	3.94	3.94	3.94	1.5708	1.5708	1.5708	-0.1228
1.22	1.9	0.68	1.56	0.48083	4	5	1	4.5	0.70711	3	11	8	7	5.65685	3	3	0	3	0	63.546	88.9059	25.3599	76.2259	17.9321	1.35	1.8	0.45	1.58	0.3182	25	72	47	48.5	33.234	1.72E-08	6.00E-07	5.83E-07	3.09E-07	4.12E-07	3300	3570	270	3435	190.919	17	400	383	208.5	270.822	1357.77	1799	441.23	1578.39	311.997	41	140	99	90.5	70.0036	1.06E-05	1.65E-05	5.90E-06	1.36E-05	4.17E-06	41.5965	221	3.46486	3.46486	3.46486	1.5708	1.5708	1.5708	-0.2439
1.3	1.9	0.6	1.6	0.42426	5	6	1	5.5	0.70711	4	7	3	5.5	2.12132	3	3	0	3	0	98	178.49	80.49	138.245	56.915	1.35	1.55	0.2	1.45	0.14142	50	59	9	54.5	6.36396	2.20E-07	3.40E-07	1.20E-07	2.80E-07	8.49E-08	0	0	0	0	0	23	51	28	37	19.799	2430	2506	76	2468	53.7401	0	0	0	0	0	0	0	0	0	0	34.6008	221	3.25858	3.25858	3.25858	1.5708	1.5708	1.5708	-0.4823
1.2	1.31	0.11	1.255	0.07778	3	8	5	5.5	3.53553	2	9	7	5.5	4.94975	1	3	2	2	1.41421	24.305	151.964	127.659	88.1345	90.2685	1.5	1.85	0.35	1.68	0.24749	18	73	55	45.5	38.8909	4.40E-08	9.00E-07	8.56E-07	4.72E-07	6.05E-07	0	0	0	0	0	14	160	146	87	103.238	923	1099	176	1011	124.451	8.3	45	36.7	26.65	25.9508	8.20E-06	3.50E-05	2.68E-05	2.16E-05	1.90E-05	66.8207	221	4.05792	4.05792	4.05792	1.5708	1.5708	1.5708	-0.0991
1.17	1.31	0.14	1.24	0.09899	3	8	5	5.5	3.53553	2	8	6	5	4.24264	1	3	2	2	1.41421	24.305	150.36	126.055	87.3325	89.1343	1.5	1.85	0.35	1.68	0.24749	28	73	45	50.5	31.8198	4.40E-08	9.40E-07	8.96E-07	4.92E-07	6.34E-07	2130	4602	2472	3366	1747.97	13	160	147	86.5	103.945	923	1345	422	1134	298.399	38	45	7	41.5	4.94975	8.20E-06	1.27E-05	4.50E-06	1.05E-05	3.18E-06	56.7043	221	3.84183	3.84183	3.84183	1.5708	1.5708	1.5708	-0.1044
0	0	0	0	0	2	2	0	2	0	18	18	0	18	0	2	2	0	2	0	20.1797	20.1797	0	20.1797	0	0	0	0	0	0	2	2	0	2	0	0	0	0	0	0	936	936	0	936	0	0.0491	0.0491	0	0.0491	0	24.56	24.56	0	24.56	0	0	0	0	0	0	0	0	0	0	0	10.0614	225	2.42321	2.42321	2.42321	1.5708	2.0944	2.0944	0.32978
2.01	2.55	0.54	2.28	0.38184	2	4	2	3	1.41421	14	14	0	14	0	2	2	0	2	0	12.0107	72.64	60.6293	42.3254	42.8714	0.7	1.25	0.55	0.98	0.38891	84	95	11	89.5	7.77817	1.00E-05	0.0005	0.00049	0.00026	0.00035	5400	18350	12950	11875	9157.03	60	140	80	100	56.5685	1211.4	3800	2588.6	2505.7	1830.42	0	0	0	0	0	6.00E-06	7.10E-06	1.10E-06	6.55E-06	7.78E-07	24.0721	216	3.24098	3.24098	3.24098	1.5708	2.0944	2.0944	0.43361
1.36	2.55	1.19	1.955	0.84146	2	4	2	3	1.41421	3	14	11	8.5	7.77817	2	3	1	2.5	0.70711	12.0107	44.9559	32.9452	28.4833	23.2958	0.7	1.6	0.9	1.15	0.6364	19	95	76	57	53.7401	5.50E-07	1.00E-05	9.45E-06	5.28E-06	6.68E-06	0	0	0	0	0	16	140	124	78	87.6812	1814	3800	1986	2807	1404.31	33	57	24	45	16.9706	7.10E-06	1.02E-05	3.10E-06	8.65E-06	2.19E-06	23.7885	221	2.876	2.876	2.876	1.5708	1.5708	1.5708	0.38917
1.24	2.28	1.04	1.76	0.73539	5	8	3	6.5	2.12132	9	14	5	11.5	3.53553	3	3	0	3	0	102.906	167.259	64.3535	135.082	45.5048	1.35	1.75	0.4	1.55	0.28284	22	65	43	43.5	30.4056	4.30E-08	8.60E-07	8.17E-07	4.52E-07	5.78E-07	2830	4700	1870	3765	1322.29	15	150	135	82.5	95.4594	1802	2237	435	2019.5	307.591	44	380	336	212	237.588	8.20E-06	1.22E-05	4.00E-06	1.02E-05	2.83E-06	38.833	221	3.38636	3.38636	3.38636	1.5708	1.5708	1.5708	-0.9357
1.22	2.55	1.33	1.885	0.94045	2	5	3	3.5	2.12132	3	14	11	8.5	7.77817	2	3	1	2.5	0.70711	12.0107	88.9059	76.8952	50.4583	54.3731	0.7	1.8	1.1	1.25	0.77782	25	95	70	60	49.4975	6.00E-07	1.00E-05	9.40E-06	5.30E-06	6.65E-06	3300	18350	15050	10825	10642	17	140	123	78.5	86.9741	1799	3800	2001	2799.5	1414.92	33	41	8	37	5.65685	7.10E-06	1.06E-05	3.50E-06	8.85E-06	2.47E-06	29.8509	221	3.10208	3.10208	3.10208	1.5708	1.5708	1.5708	0.56832
0	0	0	0	0	2	2	0	2	0	18	18	0	18	0	2	2	0	2	0	20.1797	20.1797	0	20.1797	0	0	0	0	0	0	2	2	0	2	0	0	0	0	0	0	936	936	0	936	0	0.0491	0.0491	0	0.0491	0	24.56	24.56	0	24.56	0	0	0	0	0	0	0	0	0	0	0	10.1864	225	2.4332	2.4332	2.4332	1.5708	2.0944	2.0944	0.31393
2.55	2.55	0	2.55	0	4	4	0	4	0	16	16	0	16	0	2	2	0	2	0	78.96	78.96	0	78.96	0	1.15	1.15	0	1.15	0	93	93	0	93	0	1.00E-08	1.00E-08	0	1.00E-08	0	3350	3350	0	3350	0	0.52	0.52	0	0.52	0	494	494	0	494	0	8.3	8.3	0	8.3	0	0	0	0	0	0	22.3286	166	2.8177	2.8177	2.8177	1.6059	1.6059	1.6059	0.17154
1.23	1.23	0	1.23	0	8	8	0	8	0	13	13	0	13	0	3	3	0	3	0	164.93	164.93	0	164.93	0	1.75	1.75	0	1.75	0	23	23	0	23	0	8.14E-07	8.14E-07	0	8.14E-07	0	2760	2760	0	2760	0	16	16	0	16	0	1734	1734	0	1734	0	40	40	0	40	0	1.12E-05	1.12E-05	0	1.12E-05	0	30.6384	225	3.51232	3.51232	3.51232	1.5708	2.0944	2.0944	0.01948
1.96	2.19	0.23	2.075	0.16263	3	5	2	4	1.41421	14	15	1	14.5	0.70711	2	2	0	2	0	30.9738	118.71	87.7362	74.8419	62.0389	1	1.45	0.45	1.23	0.3182	83	90	7	86.5	4.94975	1.00E-07	1.15E-07	1.50E-08	1.08E-07	1.06E-08	0	0	0	0	0	0.236	67	66.764	33.618	47.2093	317.3	505.08	187.78	411.19	132.781	11	58	47	34.5	33.234	0	0	0	0	0	43.5789	225	3.95	3.95	3.95	1.5708	2.0944	2.0944	-0.1691
1.23	2.1	0.87	1.665	0.61518	5	8	3	6.5	2.12132	13	16	3	14.5	2.12132	2	3	1	2.5	0.70711	127.6	164.93	37.3303	146.265	26.3965	1.4	1.75	0.35	1.58	0.24749	23	92	69	57.5	48.7904	8.14E-07	0.0001	9.92E-05	5.04E-05	7.01E-05	2610	2760	150	2685	106.066	3	16	13	9.5	9.19239	722.66	1734	1011.34	1228.33	715.125	40	65	25	52.5	17.6777	0	0	0	0	0	57.2701	225	4.32661	4.32661	4.32661	1.5708	2.0944	2.0944	-1.2438
2	2.55	0.55	2.275	0.38891	4	6	2	5	1.41421	12	16	4	14	2.82843	2	3	1	2.5	0.70711	78.96	200.59	121.63	139.775	86.0054	1.15	1.5	0.35	1.33	0.24749	74	93	19	83.5	13.435	1.00E-08	9.60E-07	9.50E-07	4.85E-07	6.72E-07	1407	3350	1943	2378.5	1373.91	0.52	8.3	7.78	4.41	5.50129	234.32	494	259.68	364.16	183.621	8.3	25	16.7	16.65	11.8087	0	0	0	0	0	60.1431	216	4.39778	4.39778	4.39778	1.5708	2.0944	2.0944	-0.183
1.1	1.69	0.59	1.395	0.41719	5	8	3	6.5	2.12132	3	12	9	7.5	6.36396	3	3	0	3	0	112.411	138.905	26.4945	125.658	18.7344	1.55	1.95	0.4	1.75	0.28284	33	75	42	54	29.6985	7.00E-08	6.15E-07	5.45E-07	3.43E-07	3.85E-07	2310	2475	165	2392.5	116.673	13	97	84	55	59.397	594.22	1193	598.78	893.61	423.401	28	42	14	35	9.89949	1.21E-05	3.08E-05	1.87E-05	2.15E-05	1.32E-05	59.9057	221	3.91282	3.91282	3.91282	1.5708	1.5708	1.5708	-0.3331
2.2	2.2	0	2.2	0	1	1	0	1	0	1	1	0	1	0	1	1	0	1	0	1.00794	1.00794	0	1.00794	0	0.25	0.25	0	0.25	0	103	103	0	103	0	0	0	0	0	0	1270	1270	0	1270	0	0.1805	0.1805	0	0.1805	0	14.01	14.01	0	14.01	0	0	0	0	0	0	0	0	0	0	0	2.72938	225	1.56865	1.56865	1.56865	1.5708	2.0944	2.0944	1.18768
2	2.58	0.58	2.29	0.41012	3	6	3	4.5	2.12132	12	16	4	14	2.82843	2	3	1	2.5	0.70711	32.065	200.59	168.525	116.328	119.165	1	1.5	0.5	1.25	0.35355	74	94	20	84	14.1421	9.60E-07	1.00E+23	1.00E+23	5.00E+22	7.07E+22	0	0	0	0	0	0.205	8.3	8.095	4.2525	5.72403	234.32	388.36	154.04	311.34	108.923	7.7	25	17.3	16.35	12.2329	0	0	0	0	0	53.7178	216	4.23524	4.23524	4.23524	1.5708	2.0944	2.0944	-0.2487
1.96	2.55	0.59	2.255	0.41719	2	5	3	3.5	2.12132	14	14	0	14	0	2	2	0	2	0	12.0107	118.71	106.699	65.3604	75.4478	0.7	1.45	0.75	1.08	0.53033	83	95	12	89	8.48528	1.15E-07	1.00E-05	9.89E-06	5.06E-06	6.99E-06	2500	18350	15850	10425	11207.6	67	140	73	103.5	51.6188	505.08	3800	3294.92	2152.54	2329.86	33	58	25	45.5	17.6777	7.10E-06	2.20E-05	1.49E-05	1.46E-05	1.05E-05	32.794	216	3.59283	3.59283	3.59283	1.5708	2.0944	2.0944	0.76514
1.36	2.19	0.83	1.775	0.5869	3	4	1	3.5	0.70711	3	15	12	9	8.48528	2	3	1	2.5	0.70711	30.9738	44.9559	13.9821	37.9648	9.88687	1	1.6	0.6	1.3	0.42426	19	90	71	54.5	50.2046	1.00E-07	5.50E-07	4.50E-07	3.25E-07	3.18E-07	0	0	0	0	0	0.236	16	15.764	8.118	11.1468	317.3	1814	1496.7	1065.65	1058.33	11	57	46	34	32.5269	0	0	0	0	0	37.4726	225	3.75615	3.75615	3.75615	1.5708	2.0944	2.0944	-1.6191
1.69	2.2	0.51	1.945	0.36062	5	5	0	5	0	10	12	2	11	1.41421	3	3	0	3	0	106.42	112.411	5.991	109.416	4.23628	1.4	1.55	0.15	1.48	0.10607	69	75	6	72	4.24264	7.00E-08	1.08E-07	3.80E-08	8.90E-08	2.69E-08	2310	3070	760	2690	537.401	72	97	25	84.5	17.6777	594.22	1828.05	1233.83	1211.14	872.45	42	180	138	111	97.5807	1.18E-05	3.08E-05	1.90E-05	2.13E-05	1.34E-05	34.5296	123	3.06351	3.06351	3.6792	1.5708	1.5708	1.5708	-0.4189
2.05	2.05	0	2.05	0	5	5	0	5	0	15	15	0	15	0	2	2	0	2	0	121.76	121.76	0	121.76	0	1.45	1.45	0	1.45	0	88	88	0	88	0	4.00E-07	4.00E-07	0	4.00E-07	0	3420	3420	0	3420	0	24	24	0	24	0	903.78	903.78	0	903.78	0	42	42	0	42	0	1.10E-05	1.10E-05	0	1.10E-05	0	63.3559	166	4.57709	4.57709	4.57709	0.9975	0.99751	0.9975	0
1.22	1.61	0.39	1.415	0.27577	3	8	5	5.5	3.53553	12	13	1	12.5	0.70711	2	3	1	2.5	0.70711	26.9815	162.5	135.518	94.7408	95.826	1.25	1.75	0.5	1.5	0.35355	24	80	56	52	39.598	2.70E-08	9.26E-07	8.99E-07	4.77E-07	6.36E-07	2710	5100	2390	3905	1689.99	11	235	224	123	158.392	933.47	1680	746.53	1306.74	527.876	41	76	35	58.5	24.7487	9.90E-06	2.31E-05	1.32E-05	1.65E-05	9.33E-06	46.5798	221	3.59804	3.59804	3.59804	1.5708	1.5708	1.5708	-0.3955
2	2.2	0.2	2.1	0.14142	5	6	1	5.5	0.70711	10	12	2	11	1.41421	3	3	0	3	0	106.42	200.59	94.17	153.505	66.5882	1.4	1.5	0.1	1.45	0.07071	69	74	5	71.5	3.53553	1.08E-07	9.60E-07	8.52E-07	5.34E-07	6.02E-07	1407	3070	1663	2238.5	1175.92	8.3	72	63.7	40.15	45.0427	234.32	1828.05	1593.73	1031.19	1126.94	25	180	155	102.5	109.602	0	0	0	0	0	35.957	123	3.08091	3.08091	3.78814	1.5708	1.5708	1.5708	-0.1427
1.2	2.18	0.98	1.69	0.69296	4	8	4	6	2.82843	10	15	5	12.5	3.53553	2	3	1	2.5	0.70711	74.9216	157.25	82.3284	116.086	58.215	1.15	1.8	0.65	1.48	0.45962	27	89	62	58	43.8406	3.30E-07	1.31E-06	9.80E-07	8.20E-07	6.93E-07	0	0	0	0	0	11	50	39	30.5	27.5772	1090	1585	495	1337.5	350.018	22	38	16	30	11.3137	0	0	0	0	0	50.7359	225	4.15538	4.15538	4.15538	1.5708	2.0944	2.0944	-1.5424
1.91	2.55	0.64	2.23	0.45255	2	4	2	3	1.41421	10	14	4	12	2.82843	2	3	1	2.5	0.70711	12.0107	58.6934	46.6827	35.3521	33.0097	0.7	1.35	0.65	1.03	0.45962	67	95	28	81	19.799	7.20E-08	1.00E-05	9.93E-06	5.04E-06	7.02E-06	4970	18350	13380	11660	9461.09	91	140	49	115.5	34.6482	1728	3800	2072	2764	1465.13	33	180	147	106.5	103.945	7.10E-06	1.34E-05	6.30E-06	1.03E-05	4.45E-06	16.9606	225	2.88393	2.88393	2.88393	1.5708	2.0944	2.0944	1.10668
1.81	1.81	0	1.81	0	4	4	0	4	0	13	13	0	13	0	2	2	0	2	0	69.723	69.723	0	69.723	0	1.3	1.3	0	1.3	0	81	81	0	81	0	1.40E-07	1.40E-07	0	1.40E-07	0	2740	2740	0	2740	0	29	29	0	29	0	302.91	302.91	0	302.91	0	0	0	0	0	0	0.00012	0.00012	0	0.00012	0	36.9491	63	2.79219	3.25394	4.29973	1.24011	1.5708	1.5708	0.02375
1.33	2	0.67	1.665	0.47376	5	6	1	5.5	0.70711	4	12	8	8	5.65685	3	3	0	3	0	91.224	200.59	109.366	145.907	77.3334	1.5	1.55	0.05	1.53	0.03536	49	74	25	61.5	17.6777	4.33E-07	9.60E-07	5.27E-07	6.97E-07	3.73E-07	1407	3800	2393	2603.5	1692.11	8.3	23	14.7	15.65	10.3945	234.32	2128	1893.68	1181.16	1339.03	0	0	0	0	0	0	0	0	0	0	42.5813	123	3.17549	3.17549	4.22277	1.5708	1.5708	1.5708	-0.1402
1.13	2.1	0.97	1.615	0.68589	5	8	3	6.5	2.12132	5	16	11	10.5	7.77817	2	3	1	2.5	0.70711	127.6	140.908	13.3076	134.254	9.40993	1.4	1.85	0.45	1.63	0.3182	31	92	61	61.5	43.1335	7.00E-07	0.0001	9.93E-05	5.04E-05	7.02E-05	2280	2610	330	2445	233.345	3	13	10	8	7.07107	722.66	1208	485.34	965.33	343.187	29	65	36	47	25.4558	0	0	0	0	0	59.239	221	3.89825	3.89825	3.89825	1.5708	1.5708	1.5708	-1.3483
1.9	2.04	0.14	1.97	0.09899	2	5	3	3.5	2.12132	7	13	6	10	4.24264	2	3	1	2.5	0.70711	10.811	98	87.189	54.4055	61.6519	0.85	1.35	0.5	1.1	0.35355	59	86	27	72.5	19.0919	2.20E-07	1E+12	1E+12	5E+11	7.1E+11	0	0	0	0	0	27	51	24	39	16.9706	2349	2430	81	2389.5	57.2756	0	0	0	0	0	0	0	0	0	0	21.2836	225	3.11067	3.11067	3.11067	1.5708	2.0944	2.0944	-0.0076
1.6	2.58	0.98	2.09	0.69296	3	5	2	4	1.41421	5	16	11	10.5	7.77817	2	3	1	2.5	0.70711	32.065	92.9064	60.8414	62.4857	43.0214	1	1.45	0.45	1.23	0.3182	53	94	41	73.5	28.9914	1.52E-07	1.00E+23	1.00E+23	5.00E+22	7.07E+22	0	0	0	0	0	0.205	54	53.795	27.1025	38.0388	388.36	2750	2361.64	1569.18	1669.93	7.7	170	162.3	88.85	114.763	0	0	0	0	0	31.4653	187	3.30608	3.30616	3.32399	1.5708	1.5708	2.09438	-1.0367
1.91	3.04	1.13	2.475	0.79903	2	4	2	3	1.41421	10	15	5	12.5	3.53553	2	3	1	2.5	0.70711	14.0067	58.6934	44.6867	36.35	31.5983	0.65	1.35	0.7	1	0.49497	67	100	33	83.5	23.3345	0	0	0	0	0	333.6	4970	4636.4	2651.8	3278.43	0.02583	91	90.9742	45.5129	64.3285	63.05	1728	1664.95	895.525	1177.3	0	0	0	0	0	0	0	0	0	0	19.9326	216	3.04341	3.04341	3.04341	1.5708	2.0944	2.0944	0.34759
1.2	2.54	1.34	1.87	0.94752	6	8	2	7	1.41421	10	11	1	10.5	0.70711	3	3	0	3	0	157.25	196.967	39.7166	177.108	28.0839	1.35	1.8	0.45	1.58	0.3182	27	70	43	48.5	30.4056	2.20E-08	1.31E-06	1.29E-06	6.66E-07	9.11E-07	1740	2680	940	2210	664.68	11	320	309	165.5	218.496	1337.33	1585	247.67	1461.17	175.129	38	220	182	129	128.693	9.40E-06	1.42E-05	4.80E-06	1.18E-05	3.39E-06	47.3209	221	3.61702	3.61702	3.61702	1.5708	1.5708	1.5708	-0.84
1.1	2	0.9	1.55	0.6364	6	8	2	7	1.41421	3	12	9	7.5	6.36396	3	3	0	3	0	138.905	200.59	61.6845	169.748	43.6175	1.5	1.95	0.45	1.73	0.3182	33	74	41	53.5	28.9914	6.15E-07	9.60E-07	3.45E-07	7.88E-07	2.44E-07	1407	2475	1068	1941	755.19	8.3	13	4.7	10.65	3.3234	234.32	1193	958.68	713.66	677.889	25	28	3	26.5	2.12132	0	0	0	0	0	58.4591	221	3.88106	3.88106	3.88106	1.5708	1.5708	1.5708	-0.4666
0	0	0	0	0	2	2	0	2	0	18	18	0	18	0	2	2	0	2	0	20.1797	20.1797	0	20.1797	0	0	0	0	0	0	2	2	0	2	0	0	0	0	0	0	936	936	0	936	0	0.0491	0.0491	0	0.0491	0	24.56	24.56	0	24.56	0	0	0	0	0	0	0	0	0	0	0	10.0184	225	2.41975	2.41975	2.41975	1.5708	2.0944	2.0944	0.33543
1.55	2.55	1	2.05	0.70711	4	4	0	4	0	7	16	9	11.5	6.36396	2	3	1	2.5	0.70711	54.938	78.96	24.022	66.949	16.9861	1.15	1.4	0.25	1.28	0.17678	60	93	33	76.5	23.3345	1.00E-08	1.44E-06	1.43E-06	7.25E-07	1.01E-06	3350	5150	1800	4250	1272.79	0.52	7.8	7.28	4.16	5.14774	494	1519	1025	1006.5	724.784	8.3	120	111.7	64.15	78.9838	0	0	0	0	0	51.0937	216	4.16512	4.16512	4.16512	1.5708	2.0944	2.0944	-0.3584
1.31	1.62	0.31	1.465	0.2192	3	6	3	4.5	2.12132	2	13	11	7.5	7.77817	1	2	1	1.5	0.70711	24.305	204.383	180.078	114.344	127.335	1.5	1.9	0.4	1.7	0.28284	73	78	5	75.5	3.53553	4.40E-08	1.50E-07	1.06E-07	9.70E-08	7.50E-08	818	4602	3784	2710	2675.69	46	160	114	103	80.6102	577	923	346	750	244.659	43	45	2	44	1.41421	8.20E-06	2.99E-05	2.17E-05	1.91E-05	1.53E-05	48.4111	221	3.64459	3.64459	3.64459	1.5708	1.5708	1.5708	-0.0342
0.98	0.98	0	0.98	0	2	2	0	2	0	1	1	0	1	0	1	1	0	1	0	6.941	6.941	0	6.941	0	1.45	1.45	0	1.45	0	12	12	0	12	0	9.50E-08	9.50E-08	0	9.50E-08	0	6000	6000	0	6000	0	85	85	0	85	0	453.69	453.69	0	453.69	0	11	11	0	11	0	4.60E-05	4.60E-05	0	4.60E-05	0	16.7856	166	2.83773	2.83773	2.83773	2.06416	2.06416	1.07743	0.02432
0	0	0	0	0	2	2	0	2	0	18	18	0	18	0	2	2	0	2	0	20.1797	20.1797	0	20.1797	0	0	0	0	0	0	2	2	0	2	0	0	0	0	0	0	936	936	0	936	0	0.0491	0.0491	0	0.0491	0	24.56	24.56	0	24.56	0	0	0	0	0	0	0	0	0	0	0	10.2936	225	2.44171	2.44171	2.44171	1.5708	2.0944	2.0944	0.30099
0	0	0	0	0	2	2	0	2	0	18	18	0	18	0	2	2	0	2	0	20.1797	20.1797	0	20.1797	0	0	0	0	0	0	2	2	0	2	0	0	0	0	0	0	936	936	0	936	0	0.0491	0.0491	0	0.0491	0	24.56	24.56	0	24.56	0	0	0	0	0	0	0	0	0	0	0	10.0465	225	2.42202	2.42202	2.42202	1.5708	2.0944	2.0944	0.33172
1.25	1.31	0.06	1.28	0.04243	3	8	5	5.5	3.53553	2	15	13	8.5	9.19239	1	3	2	2	1.41421	24.305	168.934	144.629	96.6196	102.268	1.5	1.75	0.25	1.63	0.17678	21	73	52	47	36.7696	4.40E-08	6.76E-07	6.32E-07	3.60E-07	4.47E-07	0	0	0	0	0	17	160	143	88.5	101.116	923	1818	895	1370.5	632.861	45	45	0	45	0	8.20E-06	1.33E-05	5.10E-06	1.08E-05	3.61E-06	52.669	221	3.74845	3.74845	3.74845	1.5708	1.5708	1.5708	-0.0568
0.95	2.55	1.6	1.75	1.13137	4	5	1	4.5	0.70711	2	16	14	9	9.89949	1	2	1	1.5	0.70711	78.96	87.62	8.66	83.29	6.12354	1.15	2	0.85	1.58	0.60104	15	93	78	54	55.1543	1.00E-08	1.35E-07	1.25E-07	7.25E-08	8.84E-08	0	0	0	0	0	0.52	35	34.48	17.76	24.381	494	1050	556	772	393.151	0	0	0	0	0	0	0	0	0	0	62.0255	225	4.44319	4.44319	4.44319	1.5708	2.0944	2.0944	-2.0725
0	0	0	0	0	1	1	0	1	0	18	18	0	18	0	2	2	0	2	0	4.0026	4.0026	0	4.0026	0	0	0	0	0	0	1	1	0	1	0	0	0	0	0	0	970	970	0	970	0	0.1513	0.1513	0	0.1513	0	0.95	0.95	0	0.95	0	0	0	0	0	0	0	0	0	0	0	9.90896	225	2.41091	2.41091	2.41091	1.5708	2.0944	2.0944	0.03061
1.69	2.28	0.59	1.985	0.41719	5	6	1	5.5	0.70711	10	12	2	11	1.41421	3	3	0	3	0	112.411	195.084	82.673	153.748	58.4586	1.35	1.55	0.2	1.45	0.14142	68	75	7	71.5	4.94975	7.00E-08	1.06E-07	3.60E-08	8.80E-08	2.55E-08	2310	2680	370	2495	261.63	72	97	25	84.5	17.6777	594.22	2041.4	1447.18	1317.81	1023.31	42	230	188	136	132.936	8.80E-06	3.08E-05	2.20E-05	1.98E-05	1.56E-05	34.7228	123	2.99637	2.99637	3.86746	1.5708	1.5708	1.5708	-0.3238
1.66	3.04	1.38	2.35	0.97581	2	4	2	3	1.41421	6	15	9	10.5	6.36396	2	3	1	2.5	0.70711	14.0067	51.9961	37.9894	33.0014	26.8626	0.65	1.4	0.75	1.03	0.53033	57	100	43	78.5	30.4056	0	0	0	0	0	333.6	5940	5606.4	3136.8	3964.32	0.02583	94	93.9742	47.0129	66.4498	63.05	2180	2116.95	1121.53	1496.91	0	0	0	0	0	0	0	0	0	0	17.5176	225	2.91517	2.91517	2.91517	1.5708	2.0944	2.0944	-0.4379
2.33	2.55	0.22	2.44	0.15556	4	6	2	5	1.41421	14	16	2	15	1.41421	2	2	0	2	0	78.96	207.2	128.24	143.08	90.6794	1.15	1.8	0.65	1.48	0.45962	82	93	11	87.5	7.77817	1.00E-08	2.10E-07	2.00E-07	1.10E-07	1.41E-07	1260	3350	2090	2305	1477.85	0.52	35	34.48	17.76	24.381	494	600.61	106.61	547.305	75.3847	8.3	46	37.7	27.15	26.6579	0	0	0	0	0	53.0103	221	3.75653	3.75653	3.75653	1.5708	1.5708	1.5708	-0.3305
1.63	3.44	1.81	2.535	1.27986	2	4	2	3	1.41421	5	16	11	10.5	7.77817	2	3	1	2.5	0.70711	15.9994	50.9415	34.9421	33.4705	24.7078	0.6	1.35	0.75	0.98	0.53033	54	101	47	77.5	33.234	0	0	0	0	0	317.5	4560	4242.5	2438.75	2999.9	0.02658	31	30.9734	15.5133	21.9015	54.8	2183	2128.2	1118.9	1504.86	0	0	0	0	0	0	0	0	0	0	21.6233	225	3.12713	3.12713	3.12713	1.5708	2.0944	2.0944	-2.26
1.69	2.28	0.59	1.985	0.41719	5	6	1	5.5	0.70711	10	12	2	11	1.41421	3	3	0	3	0	112.411	195.084	82.673	153.748	58.4586	1.35	1.55	0.2	1.45	0.14142	68	75	7	71.5	4.94975	7.00E-08	1.06E-07	3.60E-08	8.80E-08	2.55E-08	2310	2680	370	2495	261.63	72	97	25	84.5	17.6777	594.22	2041.4	1447.18	1317.81	1023.31	42	230	188	136	132.936	8.80E-06	3.08E-05	2.20E-05	1.98E-05	1.56E-05	34.7213	123	2.99615	2.99615	3.86783	1.5708	1.5708	1.5708	-0.3238
1	1.78	0.78	1.39	0.55154	4	5	1	4.5	0.70711	2	13	11	7.5	7.77817	1	2	1	1.5	0.70711	40.078	114.818	74.74	77.448	52.8492	1.55	1.8	0.25	1.68	0.17678	16	79	63	47.5	44.5477	3.40E-08	8.00E-08	4.60E-08	5.70E-08	3.25E-08	1215	3810	2595	2512.5	1834.94	82	200	118	141	83.4386	429.75	1115	685.25	772.375	484.545	0	0	0	0	0	2.23E-05	3.21E-05	9.80E-06	2.72E-05	6.93E-06	56.4322	221	3.83566	3.83566	3.83566	1.5708	1.5708	1.5708	-0.4867
1.57	3.44	1.87	2.505	1.32229	2	2	0	2	0	2	16	14	9	9.89949	1	2	1	1.5	0.70711	9.01218	15.9994	6.98722	12.5058	4.94071	0.6	1.05	0.45	0.83	0.3182	77	101	24	89	16.9706	0	0	0	0	0	317.5	13000	12682.5	6658.75	8967.88	0.02658	190	189.973	95.0133	134.331	54.8	1560	1505.2	807.4	1064.34	0	0	0	0	0	0	0	0	0	0	13.7009	216	2.68589	2.68589	2.68589	1.5708	2.0944	2.0944	-2.9604
1.23	2.54	1.31	1.885	0.92631	6	8	2	7	1.41421	11	13	2	12	1.41421	3	3	0	3	0	164.93	196.967	32.0362	180.948	22.653	1.35	1.75	0.4	1.55	0.28284	23	70	47	46.5	33.234	2.20E-08	8.14E-07	7.92E-07	4.18E-07	5.60E-07	1740	2760	1020	2250	721.249	16	320	304	168	214.96	1337.33	1734	396.67	1535.67	280.488	40	220	180	130	127.279	1.12E-05	1.42E-05	3.00E-06	1.27E-05	2.12E-06	45.3111	221	3.56507	3.56507	3.56507	1.5708	1.5708	1.5708	-0.875
1.25	1.65	0.4	1.45	0.28284	4	8	4	6	2.82843	12	15	3	13.5	2.12132	3	3	0	3	0	65.409	168.934	103.525	117.172	73.2034	1.35	1.75	0.4	1.55	0.28284	21	76	55	48.5	38.8909	6.00E-08	6.76E-07	6.16E-07	3.68E-07	4.36E-07	0	0	0	0	0	17	120	103	68.5	72.832	692.68	1818	1125.32	1255.34	795.721	45	70	25	57.5	17.6777	1.33E-05	3.02E-05	1.69E-05	2.18E-05	1.20E-05	43.366	221	3.51331	3.51331	3.51331	1.5708	1.5708	1.5708	-0.3532
0	0	0	0	0	2	2	0	2	0	18	18	0	18	0	2	2	0	2	0	20.1797	20.1797	0	20.1797	0	0	0	0	0	0	2	2	0	2	0	0	0	0	0	0	936	936	0	936	0	0.0491	0.0491	0	0.0491	0	24.56	24.56	0	24.56	0	0	0	0	0	0	0	0	0	0	0	10.0034	225	2.41855	2.41855	2.41855	1.5708	2.0944	2.0944	0.33741
0.95	1.31	0.36	1.13	0.25456	3	5	2	4	1.41421	2	2	0	2	0	1	1	0	1	0	24.305	87.62	63.315	55.9625	44.7705	1.5	2	0.5	1.75	0.35355	15	73	58	44	41.0122	4.40E-08	1.35E-07	9.10E-08	8.95E-08	6.43E-08	0	0	0	0	0	35	160	125	97.5	88.3883	923	1050	127	986.5	89.8026	0	0	0	0	0	8.20E-06	2.25E-05	1.43E-05	1.54E-05	1.01E-05	72.8647	221	4.17676	4.17676	4.17676	1.5708	1.5708	1.5708	-0.0563
1.24	2.18	0.94	1.71	0.66468	4	8	4	6	2.82843	14	15	1	14.5	0.70711	2	3	1	2.5	0.70711	74.9216	167.259	92.3374	121.09	65.2924	1.15	1.75	0.6	1.45	0.42426	22	89	67	55.5	47.3762	3.30E-07	8.60E-07	5.30E-07	5.95E-07	3.75E-07	0	0	0	0	0	15	50	35	32.5	24.7487	1090	1802	712	1446	503.46	22	44	22	33	15.5563	0	0	0	0	0	47.8257	225	4.07435	4.07435	4.07435	1.5708	2.0944	2.0944	-1.5182
1.81	1.91	0.1	1.86	0.07071	4	4	0	4	0	10	13	3	11.5	2.12132	2	3	1	2.5	0.70711	58.6934	69.723	11.0296	64.2082	7.7991	1.3	1.35	0.05	1.33	0.03536	67	81	14	74	9.89949	7.20E-08	1.40E-07	6.80E-08	1.06E-07	4.81E-08	2740	4970	2230	3855	1576.85	29	91	62	60	43.8406	302.91	1728	1425.09	1015.46	1007.69	0	0	0	0	0	1.34E-05	0.00012	0.00011	6.67E-05	7.54E-05	24.617	221	2.90901	2.90901	2.90901	1.5708	1.5708	1.5708	-0.3757
1.33	2.2	0.87	1.765	0.61518	5	5	0	5	0	4	10	6	7	4.24264	3	3	0	3	0	91.224	106.42	15.196	98.822	10.7452	1.4	1.55	0.15	1.48	0.10607	49	69	20	59	14.1421	1.08E-07	4.33E-07	3.25E-07	2.71E-07	2.30E-07	3070	3800	730	3435	516.188	23	72	49	47.5	34.6482	1828.05	2128	299.95	1978.03	212.097	0	0	0	0	0	5.70E-06	1.18E-05	6.10E-06	8.75E-06	4.31E-06	37.665	221	3.35207	3.35207	3.35207	1.5708	1.5708	1.5708	-0.585
1.55	2	0.45	1.775	0.3182	4	6	2	5	1.41421	7	12	5	9.5	3.53553	3	3	0	3	0	54.938	200.59	145.652	127.764	102.991	1.4	1.5	0.1	1.45	0.07071	60	74	14	67	9.89949	9.60E-07	1.44E-06	4.80E-07	1.20E-06	3.39E-07	1407	5150	3743	3278.5	2646.7	7.8	8.3	0.5	8.05	0.35355	234.32	1519	1284.68	876.66	908.406	25	120	95	72.5	67.1751	0	0	0	0	0	36.3926	221	3.31389	3.31389	3.31389	1.5708	1.5708	1.5708	0.36427
0	0	0	0	0	2	2	0	2	0	18	18	0	18	0	2	2	0	2	0	20.1797	20.1797	0	20.1797	0	0	0	0	0	0	2	2	0	2	0	0	0	0	0	0	936	936	0	936	0	0.0491	0.0491	0	0.0491	0	24.56	24.56	0	24.56	0	0	0	0	0	0	0	0	0	0	0	10.2923	225	2.44161	2.44161	2.44161	1.5708	2.0944	2.0944	0.30114
0	0	0	0	0	2	2	0	2	0	18	18	0	18	0	2	2	0	2	0	20.1797	20.1797	0	20.1797	0	0	0	0	0	0	2	2	0	2	0	0	0	0	0	0	936	936	0	936	0	0.0491	0.0491	0	0.0491	0	24.56	24.56	0	24.56	0	0	0	0	0	0	0	0	0	0	0	10.2435	225	2.43774	2.43774	2.43774	1.5708	2.0944	2.0944	0.30696
1.5	3.04	1.54	2.27	1.08894	2	6	4	4	2.82843	5	15	10	10	7.07107	2	3	1	2.5	0.70711	14.0067	180.948	166.941	97.4773	118.045	0.65	1.45	0.8	1.05	0.56569	52	100	48	76	33.9411	0	0	0	0	0	333.6	3400	3066.4	1866.8	2168.27	0.02583	57	56.9742	28.5129	40.2868	63.05	3290	3226.95	1676.53	2281.8	0	0	0	0	0	0	0	0	0	0	21.3991	225	3.11628	3.11628	3.11628	1.5708	2.0944	2.0944	-0.9505
1.1	2.19	1.09	1.645	0.77075	3	8	5	5.5	3.53553	11	15	4	13	2.82843	2	3	1	2.5	0.70711	30.9738	158.925	127.952	94.9496	90.4754	1	1.75	0.75	1.38	0.53033	26	90	64	58	45.2548	1.00E-07	1.15E-06	1.05E-06	6.25E-07	7.42E-07	0	0	0	0	0	0.236	11	10.764	5.618	7.6113	317.3	1629	1311.7	973.15	927.512	11	38.7	27.7	24.85	19.5869	0	0	0	0	0	46.1459	225	4.02608	4.02608	4.02608	1.5708	2.0944	2.0944	-1.4093
2	2.1	0.1	2.05	0.07071	5	6	1	5.5	0.70711	12	16	4	14	2.82843	2	3	1	2.5	0.70711	127.6	200.59	72.99	164.095	51.6117	1.4	1.5	0.1	1.45	0.07071	74	92	18	83	12.7279	9.60E-07	0.0001	9.90E-05	5.05E-05	7.00E-05	1407	2610	1203	2008.5	850.649	3	8.3	5.3	5.65	3.74767	234.32	722.66	488.34	478.49	345.309	25	65	40	45	28.2843	0	0	0	0	0	57.3571	225	4.3288	4.3288	4.3288	1.5708	2.0944	2.0944	0.01382
1.55	2	0.45	1.775	0.3182	4	6	2	5	1.41421	7	12	5	9.5	3.53553	3	3	0	3	0	54.938	200.59	145.652	127.764	102.991	1.4	1.5	0.1	1.45	0.07071	60	74	14	67	9.89949	9.60E-07	1.44E-06	4.80E-07	1.20E-06	3.39E-07	1407	5150	3743	3278.5	2646.7	7.8	8.3	0.5	8.05	0.35355	234.32	1519	1284.68	876.66	908.406	25	120	95	72.5	67.1751	0	0	0	0	0	36.4513	123	3.30775	3.30775	3.33156	1.5708	1.5708	1.5708	0.36425
1.17	1.69	0.52	1.43	0.3677	5	8	3	6.5	2.12132	8	12	4	10	2.82843	3	3	0	3	0	112.411	150.36	37.949	131.386	26.834	1.55	1.85	0.3	1.7	0.21213	28	75	47	51.5	33.234	7.00E-08	9.40E-07	8.70E-07	5.05E-07	6.15E-07	2130	2310	180	2220	127.279	13	97	84	55	59.397	594.22	1345	750.78	969.61	530.882	38	42	4	40	2.82843	1.27E-05	3.08E-05	1.81E-05	2.18E-05	1.28E-05	54.5879	221	3.79343	3.79343	3.79343	1.5708	1.5708	1.5708	-0.3248
2.36	2.55	0.19	2.455	0.13435	2	6	4	4	2.82843	6	14	8	10	5.65685	2	3	1	2.5	0.70711	12.0107	183.84	171.829	97.9254	121.502	0.7	1.35	0.65	1.03	0.45962	55	95	40	75	28.2843	5.40E-08	1.00E-05	9.95E-06	5.03E-06	7.03E-06	5174	18350	13176	11762	9316.84	140	170	30	155	21.2132	3695	3800	105	3747.5	74.2462	33	310	277	171.5	195.869	4.50E-06	7.10E-06	2.60E-06	5.80E-06	1.84E-06	21.1131	187	2.92309	2.92312	2.8532	1.5708	1.5708	2.09439	-0.1257
0.79	3.16	2.37	1.975	1.67584	3	6	3	4.5	2.12132	1	17	16	9	11.3137	1	2	1	1.5	0.70711	35.453	132.905	97.4525	84.1792	68.9093	1	2.6	1.6	1.8	1.13137	8	99	91	53.5	64.3467	2.10E-07	1E+10	1E+10	5E+09	7.1E+09	0	0	0	0	0	0.0089	36	35.9911	18.0045	25.4496	171.6	301.59	129.99	236.595	91.9168	1.1	1.6	0.5	1.35	0.35355	0	0	0	0	0	71.862	221	4.15751	4.15751	4.15751	1.5708	1.5708	1.5708	-2.1052
1.22	1.22	0	1.22	0	5	5	0	5	0	3	3	0	3	0	3	3	0	3	0	88.9059	88.9059	0	88.9059	0	1.8	1.8	0	1.8	0	25	25	0	25	0	6.00E-07	6.00E-07	0	6.00E-07	0	3300	3300	0	3300	0	17	17	0	17	0	1799	1799	0	1799	0	41	41	0	41	0	1.06E-05	1.06E-05	0	1.06E-05	0	64.6224	194	3.62755	3.62748	5.67069	1.5708	1.5708	2.09441	0.00067
1.65	2.58	0.93	2.115	0.65761	3	4	1	3.5	0.70711	12	16	4	14	2.82843	2	3	1	2.5	0.70711	32.065	65.409	33.344	48.737	23.5778	1	1.35	0.35	1.18	0.24749	76	94	18	85	12.7279	6.00E-08	1.00E+23	1.00E+23	5.00E+22	7.07E+22	0	0	0	0	0	0.205	120	119.795	60.1025	84.7079	388.36	692.68	304.32	540.52	215.187	7.7	70	62.3	38.85	44.0528	0	0	0	0	0	39.9152	216	3.83605	3.83605	3.83605	1.5708	2.0944	2.0944	-0.956
1.24	2.05	0.81	1.645	0.57276	5	8	3	6.5	2.12132	14	15	1	14.5	0.70711	2	3	1	2.5	0.70711	121.76	167.259	45.499	144.51	32.1727	1.45	1.75	0.3	1.6	0.21213	22	88	66	55	46.669	4.00E-07	8.60E-07	4.60E-07	6.30E-07	3.25E-07	2830	3420	590	3125	417.193	15	24	9	19.5	6.36396	903.78	1802	898.22	1352.89	635.137	42	44	2	43	1.41421	1.10E-05	1.22E-05	1.20E-06	1.16E-05	8.49E-07	58.0175	225	4.34535	4.34535	4.34535	1.5708	2.0944	2.0944	-1.0963
1.31	1.93	0.62	1.62	0.43841	3	5	2	4	1.41421	2	11	9	6.5	6.36396	1	3	2	2	1.41421	24.305	107.868	83.5632	66.0866	59.0881	1.5	1.6	0.1	1.55	0.07071	71	73	2	72	1.41421	1.63E-08	4.40E-08	2.77E-08	3.02E-08	1.96E-08	2600	4602	2002	3601	1415.63	160	430	270	295	190.919	923	1234.93	311.93	1078.97	220.568	45	100	55	72.5	38.8909	8.20E-06	1.89E-05	1.07E-05	1.35E-05	7.57E-06	35.7837	221	3.2953	3.2953	3.2953	1.5708	1.5708	1.5708	-0.2541

1.33	2.55	1.22	1.94	0.86267	2	5	3	3.5	2.12132	4	14	10	9	7.07107	2	3	1	2.5	0.70711	12.0107
1.33	3.04	1.71	2.185	1.20915	2	5	3	3.5	2.12132	4	15	11	9.5	7.77817	2	3	1	2.5	0.70711	14.0067
1.25	1.69	0.44	1.47	0.31113	5	8	3	6.5	2.12132	12	15	3	13.5	2.12132	3	3	0	3	0	112.411
1	1.62	0.62	1.31	0.43841	4	6	2	5	1.41421	2	13	11	7.5	7.77817	1	2	1	1.5	0.70711	40.078
1.33	2.19	0.86	1.76	0.60811	3	5	2	4	1.41421	4	15	11	9.5	7.77817	2	3	1	2.5	0.70711	30.9738
0.89	2.55	1.66	1.72	1.1738	4	6	2	5	1.41421	2	16	14	9	9.89949	1	2	1	1.5	0.70711	78.96
1.31	2.58	1.27	1.945	0.89803	3	3	0	3	0	2	16	14	9	9.89949	1	2	1	1.5	0.70711	24.305
1.62	1.96	0.34	1.79	0.24042	5	6	1	5.5	0.70711	13	14	1	13.5	0.70711	2	2	0	2	0	118.71
1.22	1.69	0.47	1.455	0.33234	5	5	0	5	0	3	12	9	7.5	6.36396	3	3	0	3	0	88.9059
1.1	1.69	0.59	1.395	0.41719	5	8	3	6.5	2.12132	12	16	4	14	2.82843	3	3	0	3	0	112.411
2.16	3.04	0.88	2.6	0.62225	2	5	3	3.5	2.12132	6	15	9	10.5	6.36396	2	3	1	2.5	0.70711	14.0067
1.55	3.44	1.89	2.495	1.33643	2	4	2	3	1.41421	7	16	9	11.5	6.36396	2	3	1	2.5	0.70711	15.9994
1.23	2.19	0.96	1.71	0.67882	3	8	5	5.5	3.53553	13	15	2	14	1.41421	2	3	1	2.5	0.70711	30.9738
1.65	2.1	0.45	1.875	0.3182	4	5	1	4.5	0.70711	12	16	4	14	2.82843	2	3	1	2.5	0.70711	65.409
1.91	3.44	1.53	2.675	1.08187	2	4	2	3	1.41421	10	16	6	13	4.24264	2	3	1	2.5	0.70711	15.9994
1.9	2.55	0.65	2.225	0.45962	2	6	4	4	2.82843	7	14	7	10.5	4.94975	2	3	1	2.5	0.70711	12.0107
1.55	2.2	0.65	1.875	0.45962	4	5	1	4.5	0.70711	7	10	3	8.5	2.12132	3	3	0	3	0	54.938
1.13	2	0.87	1.565	0.61518	6	8	2	7	1.41421	5	12	7	8.5	4.94975	3	3	0	3	0	140.908
0.95	2	1.05	1.475	0.74246	5	6	1	5.5	0.70711	2	12	10	7	7.07107	1	3	2	2	1.41421	87.62
1.31	1.31	0	1.31	0	3	3	0	3	0	2	2	0	2	0	1	1	0	1	0	24.305
1.2	2.02	0.82	1.61	0.57983	6	8	2	7	1.41421	10	15	5	12.5	3.53553	2	3	1	2.5	0.70711	157.25
1.33	2.58	1.25	1.955	0.88388	3	5	2	4	1.41421	4	16	12	10	8.48528	2	3	1	2.5	0.70711	32.065
2.2	3.04	0.84	2.62	0.59397	2	5	3	3.5	2.12132	10	15	5	12.5	3.53553	2	3	1	2.5	0.70711	14.0067

[illegible]

1.36	3.04	1.68	2.2	1.18794	2	9	7	5.5	4.94975	7	15	8	11	5.65685	2	3
1.28	2.55	1.27	1.915	0.89803	2	9	7	5.5	4.94975	8	14	6	11	4.24264	2	3
1.36	1.96	0.6	1.66	0.42426	4	5	1	4.5	0.70711	3	14	11	8.5	7.77817	2	3
2.54	3.04	0.5	2.79	0.35355	2	6	4	4	2.82843	11	15	4	13	2.82843	2	3
1.81	2.2	0.39	2.005	0.27577	4	6	2	5	1.41421	9	13	4	11	2.82843	2	3
1.2	2.28	1.08	1.74	0.76368	5	8	3	6.5	2.12132	9	10	1	9.5	0.70711	3	3
1.36	1.91	0.55	1.635	0.38891	4	4	0	4	0	3	10	7	6.5	4.94975	3	3
2.36	3.04	0.68	2.7	0.48083	2	6	4	4	2.82843	6	15	9	10.5	6.36396	2	3
1.1	3.04	1.94	2.07	1.37179	2	8	6	5	4.24264	3	15	12	9	8.48528	2	3
2.2	3.04	0.84	2.62	0.59397	2	6	4	4	2.82843	8	15	7	11.5	4.94975	2	3
1.61	1.9	0.29	1.755	0.20506	3	6	3	4.5	2.12132	7	13	6	10	4.24264	2	3
2.2	2.55	0.35	2.375	0.24749	2	5	3	3.5	2.12132	10	14	4	12	2.82843	2	3
1.1	1.69	0.59	1.395	0.41719	5	8	3	6.5	2.12132	11	12	1	11.5	0.70711	3	3
1.2	3.44	2.24	2.32	1.58392	2	8	6	5	4.24264	10	16	6	13	4.24264	2	3
1.36	2.58	1.22	1.97	0.86267	3	9	6	6	4.24264	7	16	9	11.5	6.36396	2	3
1.38	2.05	0.67	1.715	0.47376	5	9	4	7	2.82843	6	15	9	10.5	6.36396	2	3
1.36	2.2	0.84	1.78	0.59397	4	6	2	5	1.41421	3	9	6	6	4.24264	3	3
2.66	2.66	0	2.66	0	5	5	0	5	0	17	17	0	17	0	2	2
1.2	1.9	0.7	1.55	0.49497	4	8	4	6	2.82843	10	11	1	10.5	0.70711	3	3
1.28	3.44	2.16	2.36	1.52735	2	9	7	5.5	4.94975	8	16	8	12	5.65685	2	3
0.89	1.69	0.8	1.29	0.56569	5	6	1	5.5	0.70711	2	12	10	7	7.07107	1	3
1.54	2.2	0.66	1.87	0.46669	4	6	2	5	1.41421	4	9	5	6.5	3.53553	3	3

0.93	2.18	1.25	1.555	0.88388	3	4	1	3.5	0.70711	1	15	14	8	9.89949	1	2	[illegible]
1.3	2.19	0.89	1.745	0.62933	3	9	6	6	4.24264	4	15	11	9.5	7.77817	2	3	[illegible]
1.24	2.58	1.34	1.91	0.94752	3	8	5	5.5	3.53553	14	16	2	15	1.41421	2	3	[illegible]
1.6	3.04	1.44	2.32	1.01823	2	5	3	3.5	2.12132	5	15	10	10	7.07107	2	3	[illegible]
1.93	3.04	1.11	2.50667	0.55627	2	5	3	3	1.73205	11	15	4	13.3333	2.08167	2	3	[illegible]
1.55	2.04	0.49	1.87667	0.34648	2	4	2	2.66667	1.41421	7	13	6	11	4.24264	2	3	[illegible]
1.66	2.04	0.38	1.91333	0.2687	2	4	2	2.66667	1.41421	6	13	7	10.6667	4.94975	2	3	[illegible]
1.31	2.04	0.73	1.79667	0.51619	2	3	1	2.33333	0.70711	2	13	11	9.33333	7.77817	1	2	[illegible]
1.3	2.04	0.74	1.79333	0.52326	2	6	4	3.33333	2.82843	4	13	9	10	6.36396	2	3	[illegible]
2.2	2.2	0	2.2	0	1	1	0	1	0	1	1	0	1	0	1	1	[illegible]
1.54	1.9	0.36	1.66	0.25456	4	6	2	4.66667	1.41421	4	7	3	5	2.12132	3	3	[illegible]
1.78	3.04	1.26	2.41	0.89095	2	5	3	3.5	2.12132	13	15	2	14	1.41421	2	2	[illegible]
1.38	2.05	0.67	1.715	0.47376	5	9	4	7	2.82843	6	15	9	10.5	6.36396	2	3	[illegible]
1.31	1.96	0.65	1.52667	0.45962	3	5	2	3.66667	1.41421	2	14	12	6	8.48528	1	2	[illegible]
1.54	3.98	2.44	3.16667	1.72534	2	4	2	2.66667	1.41421	4	17	13	12.6667	9.19239	2	3	[illegible]
1.9	2.58	0.68	2.12667	0.48083	3	4	1	3.66667	0.70711	11	16	5	12.6667	3.53553	2	3	[illegible]
2.55	3.04	0.49	2.75	0.2571	2	5	3	3	1.73205	14	17	3	15.3333	1.52753	2	2	[illegible]
1.25	2.58	1.33	1.915	0.94045	3	8	5	5.5	3.53553	15	16	1	15.5	0.70711	2	3	[illegible]
1.1	2.04	0.94	1.72667	0.66468	2	8	6	4	4.24264	13	16	3	14	2.12132	2	3	[illegible]
1.23	2.28	1.05	1.84333	0.54684	6	8	2	6.66667	1.1547	10	15	5	12.6667	2.51661	2	3	[illegible]
1.22	3.04	1.82	2.13	1.28693	2	5	3	3.5	2.12132	3	15	12	9	8.48528	2	3	[illegible]
1.55	3.16	1.61	2.62333	1.13844	3	4	1	3.33333	0.70711	7	17	10	13.6667	7.07107	2	3	[illegible]
1.25	2.04	0.79	1.77667	0.55861	2	8	6	4	4.24264	13	15	2	13.6667	1.41421	2	3	[illegible]
[illegible]	[illegible]	[illegible]	[illegible]	[illegible]	[illegible]	[illegible]	[illegible]	[illegible]	[illegible]	[illegible]	[illegible]	[illegible]	[illegible]	[illegible]	[illegible]	[illegible]	[illegible]

1.33	3.04	1.71	1.8275	0.89721	2	5	3	3.75	1.64317	4	15	11	9	6.38749	2	3	1	2.5	0.63246	14.0067	91.224
1.23	2.1	0.87	1.84	0.45405	5	8	3	5.75	1.64317	11	16	5	14	2.68328	2	3	1	2.5	0.63246	107.868	164.93
1.17	2.33	1.16	1.8	0.58643	4	8	4	6	2	8	14	6	11	3	2	3	1	2.67	0.57735	63.546	207.2
1.78	2.55	0.77	2.1925	0.44288	4	5	1	4.125	0.40825	8	16	8	13	4.14039	2	3	1	2.38	0.59761	55.845	114.818
1.33	1.83	0.5	1.455	0.35355	4	5	1	4.75	0.70711	4	8	4	5	2.82843	3	3	0	3	0	55.845	91.224
0.79	3.16	2.37	2.386	1.31903	3	6	3	3.8	1.55839	1	17	16	11.6	9.08688	1	3	2	2	0.84515	35.453	132.905
0.89	3.44	2.55	2.566	1.45691	2	6	4	3.5	2.43891	2	16	14	11.8	8.1537	1	3	2	1.9	0.70711	15.9994	208.98
1.2	3.16	1.96	2.376	1.38593	3	8	5	5	3.53553	10	17	7	14.2	4.94975	2	3	1	2.4	0.70711	35.453	157.25
2.01	3.16	1.15	2.354	0.54744	1	4	3	2	1.69031	1	17	16	6.8	9.57676	1	2	1	1.4	0.65465	1.00794	72.64
0.79	3.98	3.19	2.811	1.91612	2	6	4	3.2	2.1009	1	17	16	11.3	9.5059	1	3	2	1.8	0.78784	18.9984	132.905
0.89	3.44	2.55	2.572	1.46711	2	8	6	3.7	2.87977	2	16	14	11.1	8.14629	1	3	2	2	0.83045	15.9994	138.905
1.6	3.98	2.38	3.148	1.39466	2	6	4	3.5	2.52763	5	17	12	14.3	5.2015	2	3	1	2.4	0.66667	18.9984	200.59
1.38	2.2	0.82	1.916	0.37463	3	9	6	5.4	2.80624	6	14	8	10.4	3.9211	2	3	1	2.6	0.61237	28.0855	238.029
1	3.44	2.44	2.704	1.30695	2	6	4	3.2	2.13809	2	16	14	11.8	7.48331	1	3	2	2	0.84515	15.9994	192.217
1.78	3.44	1.66	2.828	1.00763	2	5	3	2.6	1.60357	13	16	3	14.8	1.96396	2	2	0	2	0	10.811	114.818
0.79	3.98	3.19	2.818	1.91013	2	6	4	3.2	2.1009	1	17	16	11.6	9.39919	1	3	2	1.8	0.78784	18.9984	132.905
1.81	2.55	0.74	2.254	0.52326	4	4	0	4	0	13	16	3	14.8	2.12132	2	2	0	2	0	69.723	78.96
1.12	2.18	1.06	1.86	0.48651	4	8	4	4.8	2	4	15	11	10.8	5.08675	2	3	1	2.6	0.61237	58.6934	140.116
1.1	2.04	0.94	1.8	0.44349	2	8	6	4	2.73861	10	13	3	11.4	1.69558	2	3	1	2.6	0.61237	10.811	158.925
1.38	2.04	0.66	1.88	0.34184	2	9	7	4.2	3.81725	6	13	7	10.4	4.27618	2	3	1	2.4	0.65465	10.811	238.029
2.1	2.2	0.1	2.16	0.07071	5	5	0	5	0	10	16	6	12.4	4.24264	2	3	1	2.6	0.70711	106.42	127.6
1.1	3.44	2.34	2.664	1.31425	2	8	6	3.6	3.11677	3	16	13	12.4	6.79285	2	3	1	2.4	0.65465	15.9994	138.905
1.12	1.9	0.78	1.47	0.44735	3	8	5	5	3.06186	2	14	12	7.6	6.59545	1	3	2	2.2	0.93541	24.305	140.116
0.95	3.44	2.49	2.562	1.45843	2	5	3	3	1.69031	2	16	14	10.8	8.55236	1	3	2	2	0.84515	15.9994	87.62
1.3	2.01	0.71	1.584	0.50205	4	9	5	7	3.53553	4	14	10	8	7.07107	2	3	1	2.6	0.70711	72.64	232.038
1.2	2.66	1.46	1.744	0.67215	4	8	4	5.8	2.29129	8	17	9	10.6	4.15331	2	3	1	2.8	0.5	55.845	157.25
0.89	3.04	2.15	1.728	0.94459	2	6	4	4.4	1.76383	2	15	13	9.2	6.96818	1	2	1	1.6	0.57735	14.0067	137.327
1.13	2.55	1.42	2.062	0.64853	2	8	6	3.2	3	5	14	9	11.8	4.28661	2	3	1	2.2	0.5	10.811	140.908

0.89	2.18	1.29	1.35	0.72021	2	6	4	4	2	1	15	14	6	7.81025	1	2
1.9	2.2	0.3	2	0.21213	3	5	2	3.66667	1.41421	8	14	6	12	4.24264	2	3
1.38	2.02	0.64	1.7	0.45255	6	9	3	7.5	2.12132	6	15	9	10.5	6.36396	2	3
1.66	2.2	0.54	2.02	0.38184	1	4	3	2	2.12132	1	6	5	2.66667	3.53553	1	3
1	2	1	1.66667	0.70711	4	6	2	5.33333	1.41421	2	12	10	8.66667	7.07107	1	3
1.57	2.2	0.63	1.99	0.44548	2	5	3	4	2.12132	2	10	8	7.33333	5.65685	1	3
0.98	2.2	1.22	1.79333	0.70437	1	5	4	2.66667	2.08167	1	10	9	4	5.19615	1	3
1.22	1.9	0.68	1.67333	0.48083	3	8	5	4.66667	3.53553	12	14	2	13.3333	1.41421	2	3
1.9	1.9	0	1.9	3.33E-16	3	6	3	4	2.12132	7	14	7	11.6667	4.94975	2	3
0.98	1.96	0.98	1.57333	0.52166	2	5	3	4	1.73205	1	14	13	9.33333	7.23418	1	2
1.36	2.55	1.19	1.955	0.84146	2	9	7	5.5	4.94975	7	14	7	10.5	4.94975	2	3
2	2.28	0.28	2.09333	0.19799	6	6	0	6	0	10	12	2	11.3333	1.41421	3	3
1.9	2.28	0.38	2.15333	0.2687	3	6	3	5	2.12132	10	14	4	11.3333	2.82843	2	3
1.25	2.54	1.29	2.11	0.91217	6	8	2	6.66667	1.41421	11	15	4	12.3333	2.82843	3	3
1.25	2.28	1.03	1.86	0.54065	5	8	3	6.33333	1.52753	10	15	5	13.3333	2.88675	2	3
0.93	0.93	0	0.93	0	3	3	0	3	0	1	1	0	1	0	1	1
0.95	2	1.05	1.65	0.74246	5	6	1	5.66667	0.70711	2	12	10	8.66667	7.07107	1	3
2.02	2.66	0.64	2.26	0.34871	5	6	1	5.33333	0.57735	15	17	2	16	1	2	2
[illegible]																

1.38	2.54	1.16	2.15333	0.82024	6	9	3	7	2.12132	6	11	5	9.33333	3.53553	3	3	0	3	0	196.967	238.029	41.0623	210.654
1.91	3.16	1.25	2.74333	0.88388	3	4	1	3.33333	0.70711	10	17	7	14.6667	4.94975	2	3	1	2.33	0.70711	35.453	58.6934	23.2404	43.1998
1.2	2.01	0.81	1.74	0.57276	4	8	4	5.33333	2.82843	9	14	5	12.3333	3.53553	2	3	1	2.33	0.70711	72.64	151.964	79.324	99.0813
1.1	2.05	0.95	1.68667	0.51287	4	8	4	5.66667	2.08167	10	16	6	13.6667	3.21455	2	3	1	2.67	0.57735	58.6934	173.04	114.347	117.831
0.89	2.55	1.66	1.78	0.83648	2	6	4	3.66667	2.08167	2	14	12	10	6.9282	1	2	1	1.67	0.57735	12.0107	137.327	125.316	59.1411
1.38	1.96	0.58	1.75	0.3214	4	9	5	6	2.64575	6	14	8	10	4	2	3	1	2.67	0.57735	58.6934	238.029	179.336	138.477
0.95	2.55	1.6	2.01667	1.13137	2	5	3	3	2.12132	2	14	12	10	8.48528	1	2	1	1.67	0.70711	12.0107	87.62	75.6093	37.2138
1.63	2.28	0.65	2.06333	0.45962	4	6	2	5.33333	1.41421	5	10	5	8.33333	3.53553	3	3	0	3	0	50.9415	195.084	144.143	147.037
2.33	3.44	1.11	3.07	0.78489	2	6	4	3.33333	2.82843	14	16	2	15.3333	1.41421	2	2	0	2	0	15.9994	207.2	191.201	79.7329
1.22	2.28	1.06	1.84	0.55245	6	8	2	6.66667	1.1547	10	15	5	12.3333	2.51661	2	3	1	2.67	0.57735	162.5	208.98	46.4804	188.855
1.25	2.01	0.76	1.63	0.5374	4	8	4	6	2.82843	14	15	1	14.5	0.70711	2	3	1	2.5	0.70711	72.64	168.934	96.2942	120.787
0.82	2.02	1.2	1.12	0.84853	4	6	2	4.5	1.41421	1	15	14	4.5	9.89949	1	2	1	1.25	0.70711	39.0983	208.98	169.882	81.5688
1.33	1.91	0.58	1.62	0.41012	4	5	1	4.5	0.70711	4	10	6	7	4.24264	3	3	0	3	0	58.6934	91.224	32.5306	74.9587
1.12	1.65	0.53	1.4325	0.28794	3	8	5	4.75	2.42899	2	12	10	7.5	5.76194	1	3	2	2.5	1.09545	24.305	140.116	115.811	73.8098
0.98	2.2	1.22	1.685	0.55868	2	6	4	4.5	1.89737	1	13	12	9	6.19677	1	3	2	2	0.89443	6.941	192.217	185.276	107.199
1.13	2.55	1.42	2.035	0.73895	2	8	6	4	3.09839	5	14	9	10.75	4.67974	2	3	1	2.5	0.63246	12.0107	140.908	128.897	55.9056
[illegible]																							

1.91	3.98	2.07	3.29	1.46371	2	4	2	2.66667	1.41421	10	17	7	14.6667	4.94975	2	3	1	2.33	0.70711	18.9984	58.6934	39.695	32.2301	28.0686	0.5	1.35
1.1	2.05	0.95	1.68333	0.51072	4	8	4	5.66667	2.08167	11	16	5	14	2.64575	2	3	1	2.67	0.57735	63.546	173.04	109.494	119.449	54.7836	1.35	1.75
1.65	3.98	2.33	3.20333	1.64756	2	4	2	2.66667	1.41421	12	17	5	15.3333	3.53553	2	3	1	2.33	0.70711	18.9984	65.409	46.4106	34.4686	32.8172	0.5	1.35
2.2	3.04	0.84	2.46667	0.53433	1	4	3	1.66667	1.56347	1	17	16	6	10.0333	1	2	1	1.33	0.66667	1.00794	79.904	78.8961	16.3237	40.7681	0.25	1.15
1.38	3.04	1.66	2.376	1.1738	2	9	7	4.8	4.94975	6	15	9	11.4	6.36396	2	3	1	2.4	0.70711	14.0067	238.029	224.022	103.616	158.408	0.65	1.75
1.13	1.91	0.78	1.75	0.38754	3	8	5	4.4	2.3184	5	14	9	10.6	4.15331	2	3	1	2.6	0.61237	28.0855	140.908	112.822	62.8931	51.6741	1.1	1.85
1.2	1.9	0.7	1.732	0.33479	3	8	5	4.4	2.3184	8	14	6	10.6	3.5	2	3	1	2.6	0.61237	28.0855	151.964	123.879	63.965	57.1467	1.1	1.85
1.62	2.55	0.93	2.104	0.4728	4	6	2	4.4	1	11	16	5	13.4	2.80624	2	3	1	2.4	0.61237	63.546	204.383	140.837	97.8791	67.1205	1.15	1.9
1.54	3.04	1.5	2.04	1.06066	2	4	2	3.33333	1.41421	4	15	11	7.66667	7.77817	2	3	1	2.67	0.70711	14.0067	47.867	33.8603	36.5802	23.9428	0.65	1.4
1.22	1.9	0.68	1.57667	0.34122	3	5	2	3.66667	1.1547	3	14	11	10	6.08276	2	3	1	2.33	0.57735	26.9815	88.9059	61.9243	47.991	35.4376	1.1	1.8
1.13	2.55	1.42	1.96667	0.50928	2	8	6	4.33333	2.32048	5	14	9	9.83333	4.91857	2	3	1	2.5	0.58835	12.0107	140.908	128.897	66.8282	55.1705	0.7	1.85
1.38	2.33	0.95	1.855	0.67175	6	9	3	7.5	2.12132	6	14	8	10	5.65685	2	3	1	2.5	0.70711	207.2	238.029	30.8289	222.614	21.7993	1.75	1.8
1.24	1.91	0.67	1.616	0.38831	4	8	4	5.8	2.29129	10	14	4	12.2	2.29129	2	3	1	2.8	0.5	58.6934	167.259	108.566	113.345	60.697	1.35	1.75
1.12	3.44	2.32	2.65	1.32541	2	8	6	3.6	3.11677	4	16	12	13	6.21059	2	3	1	2.2	0.53452	15.9994	140.116	124.117	51.5674	65.3718	0.6	1.85
1.24	2.01	0.77	1.672	0.37286	4	8	4	4.8	2	7	14	7	11.2	4.28661	2	3	1	2.6	0.61237	54.938	167.259	112.321	84.483	52.6729	1.25	1.75
0.79	2.58	1.79	1.7875	1.01907	3	8	5	5	2.68328	1	16	15	10.5	7.82304	1	3	2	2	0.89443	32.065	151.964	119.899	87.2499	70.3224	1	2.6
1.24	2.28	1.04	1.92	0.47513	3	8	5	5.2	2.42384	10	14	4	12.4	2.44949	2	3	1	2.6	0.61237	28.0855	195.084	166.999	122.72	97.4169	1.1	1.75
1.2	2.2	1	1.924	0.4648	4	8	4	5.6	1.87083	9	14	5	11	3.06186	2	3	1	2.6	0.61237	72.64	192.217	119.577	136.336	67.5555	1.25	1.85
1.66	2.19	0.53	2.01333	0.37477	3	4	1	3.33333	0.70711	6	15	9	12	6.36396	2	3	1	2.33	0.70711	30.9738	51.9961	21.0223	37.9812	14.865	1	1.4
2.02	3.98	1.96	3.14667	1.01239	2	6	4	3.33333	2.3094	15	17	2	16	1	2	2	0	2	0	15.9994	208.98	192.981	81.3261	110.562	0.5	1.6
1.55	3.98	2.43	3.17	1.71827	2	4	2	2.66667	1.41421	7	17	10	13.6667	7.07107	2	3	1	2.33	0.70711	18.9984	54.938	35.9396	30.9783	25.4132	0.5	1.4
1.14	2.54	1.4	2.004	0.64777	3	8	5	5.2	2.42384	6	14	8	11.2	3.65718	2	3	1	2.6	0.61237	28.0855	196.967	168.881	118.869	95.73	1.1	1.85
1.24	2.01	0.77	1.784	0.35458	4	8	4	4.8	2	8	14	6	11.6	3.67423	2	3	1	2.6	0.61237	55.845	167.259	111.414	84.8458	52.3569	1.25	1.75
1.55	2.36	0.81	1.855	0.42525	4	6	2	4.75	1.04881	6	14	8	8.5	4.04969	2	3	1	2.75	0.54772	54.938	183.84	128.902	103.107	67.533	1.35	1.45
1.28	2.01	0.73	1.4625	0.51619	4	9	5	7.75	3.53553	8	14	6	9.5	4.24264	2	3	1	2.75	0.70711	72.64	244	171.36	201.16	121.17	1.25	1.75
1.63	1.83	0.2	1.775	0.1064	4	4	0	4	0	5	13	8	8.5	3.63318	2	3	1	2.75	0.54772	50.9415	69.723	18.7815	58.0886	8.86584	1.3	1.4
1.2	1.9	0.7	1.695	0.36677	4	8	4	5.25	2.07364	10	13	3	11.25	1.3784	2	3	1	2.75	0.54772	63.546	157.25	93.704	99.79	49.6175	1.35	1.8
1.14	1.9	0.76	1.68	0.3992	4	8	4	5.25	2.07364	6	13	7	10.25	3.27109	2	3	1	2.75	0.54772	63.546	144.242	80.696	96.538	43.7574	1.35	1.85
1.91	2.1	0.19	2.01	0.11419	4	5	1	4.75	0.54772	10	16	6	13.25	3.50714	2	3	1	2.5	0.63246	58.6934	127.6	68.9066	105.44	35.6274	1.35	1.6
1.17	3.44	2.27	2.4875	1.24827	2	8	6	4	3.09839	8	16	8	12.75	4.32435	2	3	1	2.5	0.63246	15.9994	150.36	134.361	61.4762	69.4	0.6	1.85
1.2	3.44	2.24	2.495	1.23565	2	8	6	4	3.09839	9	16	7	13	3.89872	2	3	1	2.5	0.63246	15.9994	151.964	135.965	61.8772	70.2224	0.6	1.85
1.63	2.28	0.65	2.0375	0.3404	4	5	1	4.75	0.54772	5	14	9	9.25	4.03733	2	3	1	2.75	0.54772	50.9415	118.71	67.7685	93.8656	32.3924	1.35	1.45

1.17	2.2	1.03	1.838	0.471	4	8	4	5.2	1.83712	8	13	5	10.8	2.42384	2	3
1.14	2.2	1.06	1.984	0.52753	3	8	5	4.8	2.29129	6	15	9	10.4	4.78278	2	3
1.13	2.55	1.42	1.512	0.73845	2	8	6	6.4	3.11677	5	14	9	8.4	5.58058	2	3
1.3	2.01	0.71	1.828	0.3347	4	9	5	5	2.5	4	14	10	10.4	4.58258	2	3
1.13	2.2	1.07	1.982	0.53253	3	8	5	4.8	2.29129	5	15	10	10.2	5.08675	2	3
0.89	2.28	1.39	1.81	0.63662	5	6	1	5.4	0.65465	2	14	12	10.8	6.23355	1	3
1.12	3.44	2.32	2.512	1.64049	2	8	6	4.4	4.24264	4	16	12	11.2	8.48528	2	3
0.82	2.58	1.76	1.96	0.80495	3	4	1	3.6	0.61237	1	16	15	10.6	6.87386	1	3
1.1	2.28	1.18	1.936	0.54386	4	8	4	5.2	1.83712	3	14	11	9.8	5.08675	2	3
1.1	2.2	1.1	1.904	0.5136	4	8	4	5.2	1.83712	3	14	11	9.4	5.22015	2	3
1.83	3.44	1.61	2.834	0.99504	2	6	4	3.2	2.13809	8	16	8	14.2	4.17475	2	3
1	2.18	1.18	1.796	0.53878	4	4	0	4	0	2	15	13	11.6	6.10328	1	2
1.66	2.54	0.88	2.364	0.62225	4	6	2	5.6	1.41421	6	11	5	10	3.53553	3	3
1.2	2.04	0.84	1.788	0.38579	2	8	6	4	2.73861	8	13	5	10.4	2.80624	2	3
0.89	2.2	1.31	1.934	0.65252	3	6	3	4.4	1.5	2	15	13	9.6	6.15427	1	3
1.27	2.2	0.93	1.894	0.42453	3	8	5	5.2	2.42384	8	17	9	12.2	4.5	2	3

1.14	1.96	0.82	1.66667	0.45709	4	8	4	5.66667	2.08167	6	14	8	10.3333	4.04145	2	3	1	2.67	0.57735	63.546	144.242	80.696	108.833	41.2448	1.35	1.85	0.5	1.55	0.26458	30	83	53	61.6667	27.9702	1.72E-08	6.43E-07	6.26E-07	2.58E-07	3.37E-07	2330	3570
1.54	3.44	1.9	2.64667	0.98799	2	4	2	3.33333	1.1547	4	17	13	12.3333	7.23418	2	3	1	2.33	0.57735	15.9994	79.904	63.9046	47.9235	31.9523	0.6	1.4	0.8	1.05	0.40927	51	101	50	83.3333	28.0416	0	0	0	0	0	0	0
1.14	1.9	0.76	1.53	0.38039	3	8	5	5	2.64575	6	14	8	9	4.3589	2	3	1	2.67	0.57735	28.0855	144.242	116.157	75.7552	60.812	1.1	1.85	0.75	1.45	0.37749	30	85	55	58.3333	27.5379	6.43E-07	0.001	0.001	0.00033	0.00058	2200	5150
1.1	1.91	0.81	1.76833	0.42272	4	8	4	4.66667	2.10819	10	16	6	11.6667	2.78887	3	3	0	3	0	58.6934	173.04	114.347	80.9862	58.2738	1.35	1.75	0.4	1.42	0.21082	17	72	55	62	28.5774	1.72E-08	2.50E-07	2.33E-07	6.51E-08	1.20E-07	1590	4970
1.24	3.16	1.92	2.32667	0.98475	3	8	5	4.66667	2.88675	14	17	3	15.6667	1.52753	2	3	1	2.33	0.57735	32.065	167.259	135.194	78.259	77.0949	1	1.75	0.75	1.25	0.43301	22	99	77	71.6667	43.0852	8.60E-07	1.00E+23	1.00E+23	3.33E+22	5.77E+22	0	0
1.1	3.44	2.34	2.5	1.23596	2	9	7	5	3.60555	3	17	14	12	7.81025	2	3	1	2.33	0.57735	15.9994	227	211.001	107.634	108.199	0.6	1.95	1.35	1.23	0.67885	48	101	53	82.3333	29.7714	0	0	0	0	0	0	0
0.82	2.18	1.36	1.65333	0.73002	4	5	1	4.33333	0.57735	1	15	14	10	7.81025	1	2	1	1.67	0.57735	39.0983	118.71	79.6117	77.5766	39.8722	1.15	2.2	1.05	1.6	0.54083	10	89	79	60.6667	43.9811	7.50E-08	3.30E-07	2.55E-07	1.73E-07	1.37E-07	0	0
1.12	1.96	0.84	1.772	0.40846	4	8	4	5.2	1.83712	4	14	10	10.4	4.58258	2	3	1	2.6	0.61237	58.6934	140.116	81.4226	98.9846	42.2668	1.35	1.85	0.5	1.49	0.23184	32	83	51	66.4	23.2863	7.20E-08	7.40E-07	6.68E-07	2.23E-07	3.24E-07	2100	4970
2.02	2.55	0.53	2.158	0.24905	4	6	2	5.2	0.93541	15	16	1	15.6	0.61237	2	2	0	2	0	78.96	208.98	130.02	150.424	63.7541	1.15	1.6	0.45	1.43	0.20767	87	93	6	90.2	3.29773	1.00E-08	0.0001	1.00E-04	4.05E-05	6.07E-05	1790	3350
1.28	3.44	2.16	2.62667	1.17462	2	9	7	4.66667	3.78594	8	17	9	13.6667	4.93288	2	3	1	2.33	0.57735	15.9994	244	228.001	98.4841	126.395	0.6	1.75	1.15	1.12	0.58381	43	101	58	81	32.9242	0	0	0	0	0	206	2260
1.27	1.96	0.69	1.81167	0.34355	4	8	4	4.83333	2.06828	10	17	7	11.8333	3.8658	2	3	1	2.83	0.52705	58.6934	174.967	116.274	88.0751	63.0921	1.35	1.75	0.4	1.43	0.20683	20	83	63	61.8333	27.7178	7.20E-08	5.80E-07	5.08E-07	1.64E-07	2.64E-07	0	0
1.13	1.65	0.52	1.56333	0.3677	4	8	4	4.66667	2.82843	5	12	7	10.8333	4.94975	3	3	0	3	0	65.409	140.908	75.4986	77.9921	53.3856	1.35	1.85	0.5	1.43	0.35355	31	76	45	68.5	31.8198	6.00E-08	7.00E-07	6.40E-07	1.67E-07	4.53E-07	2280	3700
1.17	3.44	2.27	2.52333	1.19634	2	8	6	4.66667	3.05505	8	17	9	13.6667	4.93288	2	3	1	2.33	0.57735	15.9994	150.36	134.361	82.0878	67.2069	0.6	1.85	1.25	1.2	0.6265	28	101	73	75.6667	41.3078	0	0	0	0	0	0	0
0.89	2.66	1.77	1.84	0.88413	1	6	5	3.33333	3.01511	1	17	16	4	7.45898	1	2	1	1.17	0.47673	1.00794	137.327	136.319	67.4304	85.084	0.25	2.15	1.9	1.08	1.10273	14	103	89	72.3333	52.8342	0	0	0	0	0	0	0
1.28	3.44	2.16	2.56	1.1342	2	9	7	5	3.60555	8	17	9	13.6667	4.93288	2	3	1	2.33	0.57735	15.9994	244	228.001	113.301	117.612	0.6	1.75	1.15	1.17	0.57518	43	101	58	80.6667	32.6548	0	0	0	0	0	0	0
1.2	1.9	0.7	1.55	0.35	3	8	5	5	2.64575	7	14	7	10.3333	3.51188	2	3	1	2.67	0.57735	28.0855	157.25	129.165	80.0912	68.157	1.1	1.8	0.7	1.43	0.35119	27	85	58	57.3333	29.0918	1.31E-06	0.001	0.001	0.00033	0.00058	2200	5150
1.1	1.9	0.8	1.61	0.44306	3	8	5	5	2.64575	3	14	11	8.33333	5.50757	2	3	1	2.67	0.57735	28.0855	138.905	110.82	74.2787	57.6638	1.1	1.95	0.85	1.48	0.43108	33	85	52	59.6667	26.0256	1.00E-07	0.001	0.001	0.00033	0.00058	2200	4910
1.1	2.33	1.23	1.77667	0.62421	4	8	4	6	2	11	16	5	13.6667	2.51661	2	3	1	2.67	0.57735	63.546	207.2	143.654	147.929	75.047	1.35	1.8	0.45	1.63	0.24664	17	82	65	57	35	1.72E-08	2.50E-07	2.33E-07	1.59E-07	1.24E-07	1260	3570
1.14	2.2	1.06	1.78333	0.56518	4	8	4	5.66667	2.08167	6	14	8	9.33333	4.16333	2	3	1	2.67	0.57735	72.64	144.242	71.602	105.984	36.053	1.25	1.85	0.6	1.47	0.33292	30	84	54	58.6667	27.1539	7.10E-08	0.0005	0.0005	0.00017	0.00029	2330	5970
1.3	2.16	0.86	1.87333	0.60811	5	6	1	5.33333	0.70711	4	6	2	5.33333	1.41421	3	3	0	3	0	95.94	178.49	82.55	123.457	58.3717	1.45	1.55	0.1	1.48	0.07071	50	56	6	54	4.24264	5.50E-08	3.40E-07	2.85E-07	1.50E-07	2.02E-07	3010	6190
1.3	2.05	0.75	1.8	0.53033	5	9	4	6.33333	2.82843	4	15	11	11.3333	7.77817	2	3	1	2.33	0.70711	121.76	232.038	110.278	158.519	77.9784	1.45	1.8	0.35	1.57	0.24749	47	88	41	74.3333	28.9914	1.50E-07	4.00E-07	2.50E-07	3.17E-07	1.77E-07	2490	3420
1.22	3.44	2.22	2.60667	1.20902	2	5	3	3.33333	1.52753	3	17	14	12	7.81025	2	3	1	2.33	0.57735	15.9994	88.9059	72.9065	46.7861	37.7514	0.6	1.8	1.2	1.13	0.61101	25	101	76	75	43.3128	0	0	0	0	0	206	3300
1.55	2.01	0.46	1.72333	0.25007	3	4	1	3.66667	0.57735	7	14	7	11.3333	3.78594	2	3	1	2.33	0.57735	26.9815	72.64	45.6585	51.5199	23.0204	1.25	1.4	0.15	1.3	0.0866	60	84	24	74.6667	12.8582	2.70E-08	0.0005	0.0005	0.00017	0.00029	5100	5400
1.1	2.54	1.44	1.84667	0.72148	3	8	5	5.66667	2.51661	11	16	5	13.6667	2.51661	2	3	1	2.67	0.57735	28.0855	196.967	168.881	132.697	91.383	1.1	1.75	0.65	1.4	0.32787	17	85	68	57.3333	35.7258	2.20E-08	0.001	0.001	0.00033	0.00058	1590	2200
1.3	1.91	0.61	1.70667	0.43134	4	6	2	4.66667	1.41421	4	10	6	8	4.24264	3	3	0	3	0	58.6934	178.49	119.797	98.6256	84.709	1.35	1.55	0.2	1.42	0.14142	50	67	17	61.3333	12.0208	7.20E-08	3.40E-07	2.68E-07	1.61E-07	1.90E-07	3010	4970
1.1	1.96	0.86	1.768	0.41843	4	8	4	5.2	1.83712	3	14	11	10.2	5.02494	2	3	1	2.6	0.61237	58.6934	138.905	80.2121	98.7425	41.9014	1.35	1.95	0.6	1.51	0.28062	33	83	50	66.6	22.8254	7.20E-08	6.15E-07	5.43E-07	1.98E-07	2.62E-07	2475	4970
1.36	1.9	0.54	1.72	0.38184	3	9	6	5	4.24264	7	14	7	11.6667	4.94975	2	3	1	2.33	0.70711	28.0855	237	208.915	97.7237	147.725	1.1	1.75	0.65	1.32	0.45962	44	85	41	71.3333	28.9914	1.20E-06	0.001	0.001	0.00067	0.00071	0	0
1.2	2.33	1.13	1.84333	0.58106	6	8	2	6.66667	1.1547	9	14	5	11.6667	2.51661	2	3	1	2.67	0.57735	151.964	207.2	55.236	186.585	30.164	1.5	1.85	0.35	1.72	0.1893	18	82	64	58	34.8712	2.10E-07	9.60E-07	7.50E-07	6.90E-07	4.17E-07	0	0
1	3.16	2.16	2.12	1.08222	1	4	3	2.66667	1.52753	1	17	16	6.66667	8.96289	1	2	1	1.33	0.57735	1.00794	40.078	39.0701	25.513	21.3476	0.25	1.8	1.55	1.02	0.77513	16	103	87	72.6667	49.1155	0	0	0	0	0	206	3810
1.65	3.16	1.51	2.65667	1.06773	3	4	1	3.33333	0.70711	12	17	5	15.3333	3.53553	2	3	1	2.33	0.70711	35.453	65.409	29.956	45.4383	21.1821	1	1.35	0.35	1.12	0.24749	76	99	23	91.3333	16.2635	6.00E-08	1E+10	1E+10	6.7E+09	7.1E+09	206	3700
0.95	3.44	2.49	2.35333	1.44789	2	5	3	3.33333	1.7581	2	16	14	10.5	8.01419	1	3	2	1.83	0.87905	15.9994	87.62	71.6206	47.7974	41.9514	0.6	2	1.4	1.19	0.80601	15	101	86	67.5	49.266	0	0	0	0	0	0	0
1.54	3.04	1.5	2.04	1.06066	2	4	2	3.33333	1.41421	4	15	11	7.66667	7.77817	2	3	1	2.67	0.70711	14.0067	47.867	33.8603	36.5802	23.9428	0.65	1.4	0.75	1.15	0.53033	51	100	49	67.3333	34.6482	0	0	0	0	0	333.6	4140
1.9	2.18	0.28	1.99333	0.19799	4	4	0	4	0	11	15	4	12.3333	2.82843	2	3	1	2.67	0.70711	63.546	74.9216	11.3756	67.3379	8.04376	1.15	1.35	0.2	1.28	0.14142	72	89	17	77.6667	12.0208	1.72E-08	3.30E-07	3.13E-07	1.21E-07	2.21E-07	0	0
1	1.91	0.91	1.65833	0.519	3	4	1	3.83333	0.52705	2	10	8	7.33333	5.33333	1	3	2	2.33	1.33333	24.305	58.6934	34.3884	49.8594	18.8048	1.35	1.8	0.45	1.45	0.23452	16	73	57	59.5	27.6857	3.40E-08	7.20E-08	3.80E-08	6.10E-08	2.24E-08	3810	4970
1.23	2.28	1.05	2.025	0.47507	2	8	6	4.5	2.63715	9	13	4	11	2.55841	2	3	1	2.67	0.60302	10.811	164.93	154.119	82.5448	70.6898	0.85	1.75	0.9	1.25	0.40452	23	86	63	65	26.8633	4.30E-08	1E+12	1E+12	3.3E+11	6E+11	2760	16200
1.1	3.98	2.88	2.54333	1.44001	2	8	6	4.66667	3.05505	3	17	14	12	7.81025	2	3	1	2.33	0.57735	18.9984	138.905	119.907	78.9546	59.9535	0.5	1.95	1.45	1.2	0.72629	33	102	69	76	37.51	0	0	0	0	0	0	0
1.66	3.44	1.78	2.84667	1.25865	2	4	2	2.66667	1.41421	6	16	10	12.6667	7.07107	2	3	1	2.33	0.70711	15.9994	51.9961	35.9967	27.9983	25.4535	0.6	1.4	0.8	0.87	0.56569	57	101	44	86.3333	31.1127	0	0	0	0	0	317.5	5940
1.17	1.9	0.73	1.47667	0.37873	3	8	5	5	2.64575	3	14	11	8.33333	5.50757	2	3	1	2.67	0.57735	28.0855	150.36	122.275	74.4671	66.2642	1.1	1.85	0.75	1.52	0.38188	19	85	66	44	35.7911	5.50E-07	0.001	0.001	0.00033	0.00058	0	0
0.82	3.98	3.16	3.095	1.81739	2	5	3	2.83333	1.71594	1	17	16	12.8333	8.80972	1	3	2	2	0.8165	18.9984	85.4678	66.4694	36.2177	36.5003	0.5	2.35	1.85	0.96	0.99534	9	102	93	79.6667	49.4548	0	0	0	0	0	0	0
1.33	2.01	0.68	1.78333	0.48083	4	5	1	4.33333	0.70711	4	14	10	10.6667	7.07107	2	3	1	2.33	0.70711	72.64	91.224	18.584	78.8347	13.1409	1.25	1.55	0.3	1.35	0.21213	49	84	35	72.3333	24.7487	4.33E-07	0.0005	0.0005	0.00033	0.00035	3800	5400
1.3	2.01	0.71	1.77333	0.50205	4	6	2	4.66667	1.41421	4	14	10	10.6667	7.07107	2	3	1	2.33	0.70711	72.64	178.49	105.85	107.923	74.8473	1.25	1.55	0.3	1.35	0.21213	50	84	34	72.6667	24.0416	3.40E-07	0.0005	0.0005	0.00033	0.00035	3010	5400
1.83	2.1	0.27	2.01	0.19092	4	5	1	4.66667	0.70711	8	16	8	13.3333	5.65685	2	3	1	2.33	0.70711	55.845	127.6	71.755	103.682	50.7384	1.4	1.4	0	1.4	3.33E-16	61	92	31	81.6667	21.9203	1.00E-07	0.0001	9.99E-05	6.67E-05	7.06E-05	2610	4910
2.04	2.2	0.16	2.14667	0.11314	2	5	3	4	2.12132	10	13	3	11	2.12132	2	3	1	2.67	0.70711	10.811	106.42	95.609	74.5503	67.6058	0.85	1.4	0.55	1.22	0.38891	69	86	17	74.6667	12.0208	1.08E-07	1E+12	1E+12	3.3E+11	7.1E+11	3070	16200
1.83	3.44	1.61	2.81	0.86017	2	4	2	3	1	8	17	9	13.6667	4.93288	2	3	1	2.33	0.57735	15.9994	55.845	39.8456	35.7658	19.9246	0.6	1.4	0.8	1	0.4	61	101	40	87	22.5389	0	0	0	0	0	206	4910
1.38	3.16	1.78	2.52667	0.99485	2	9	7	4.66667	3.78594	6	17	11	12.6667	5.85947	2	3	1	2.33	0.57735	14.0067	238.029	224.022	95.8295	123.614	0.65	1.75	1.1	1.13	0.56199	45	100	55	81.3333	31.4696	0	0	0	0	0	206	3155
1.69	2.18	0.49	2.01667	0.34648	4	5	1	4.33333	0.70711	12	15	3	14	2.12132	2	3	1	2.33	0.57735	74.9216	112.411	37.4894	87.4181	26.509	1.15	1.55	0.4	1.28	0.28284	75	89	14	84.3333	9.89949	7.00E-08	3.30E-07	2.60E-07	2.43E-07	1.84E-07	0	0
1.1	2.01	0.91	1.644	0.42629	4	8	4	4.8	2	7	14	7	10.6	3.9211	2	3	1	2.6	0.61237	54.938	158.925	103.987	82.8163	48.5866	1.25	1.75	0.5	1.41	0.22845	26	84	58	62.8	26.6271	1.15E-06	0.0005	0.0005	0.0002	0.00031	2620	5400
1.14	2.58	1.44	2.1	1.01823	3	8	5	4.66667	3.53553	6	16	10	12.6667	7.07107	2	3	1	2.33	0.57735	32.065	144.242	112.177	69.4573	79.3211	1	1.85	0.85	1.28	0.60104	30	94	64	72.6667	45.2548	6.43E-07	1.00E+23	1.00E+23	6.67E+22	7.07E+22	0	0
2.04	2.16	0.12	2.12	0.08485	2	5	3	4	2.12132	6	13	7	8.33333	4.94975	2	3	1	2.67	0.70711	10.811	95.94	85.129	67.5637	60.1953	0.85	1.45	0.6	1.25	0.42426	56	86	30	66	21.2132	5.50E-08	1E+12	1E+12	3.3E+11	7.1E+11	6190	16200
2.04	2.36	0.32	2.25333	0.22627	2	6	4	4.66667	2.82843	6	13	7	8.33333	4.94975	2	3	1	2.67	0.70711	10.811	183.84	173.029	126.164	122.35	0.85	1.35	0.5	1.18	0.35355	56	86	31	65.3333	21.9203	5.40E-08	1E+12	1E+12	3.3E+11	7.1E+11	5174	16200
1.38	3.04	1.66	2.204	0.92809	2	9	7	5.2	3.98434	6	15	9	11.4	5.51135	2	3	1	2.4	0.61237	14.0067	238.029	224.022	115.799	127.812	0.65	1.75	1.1	1.19	0.61543	45	100	55	75.8	31.8336	0	0	0	0	0	0	0
1.63	3.44	1.81	2.74333	0.97429	2	4	2	3	1	5	17	12	12.6667	6.65833	2	3	1	2.33	0.57735	15.9994	50.9415	34.9421	34.1313	17.5085	0.6	1.35	0.75	0.98	0.37528	54	101	47	84.6667	26.5769	0	0	0	0	0	206	4560
1.6	2.58	0.98	2.12333	0.49339	3	5	2	3.66667	1.1547	5	16	11	12	6.08276	2	3	1	2.33	0.57735	30.9738	92.9064	61.9326	51.9817	35.446	1	1.45	0.45	1.15	0.25981	53	94	41	79	22.6053	1.00E-07	1.00E+23	1.00E+23	3.33E+22	5.77E+22	0	0
1	2.05	1.05	1.7	0.74246	4	5	1	4.66667	0.70711	2	15	13	10.6667	9.19239	1	2	1	1.67	0.57735	40.078	121.76	81.682	94.5327	57.7579	1.45	1.8	0.35	1.57	0.24749	16	88	72	64	50.9117	3.40E-08	4.00E-07	3.66E-07	2.78E-07	2.59E-07	3420	3810
1.6	2.55	0.95	2.11333	0.47962	3	5	2	4	1	5	16	11	12	6.08276	2	3	1	2.33	0.57735	30.9738	92.9064	61.9326	67.6134	32.488	1	1.45	0.45	1.2	0.22913	53	93	40	78.6667	22.2785	1.00E-08	1.52E-07	1.42E-07	8.73E-08	7.18E-08	0	0
2.2	3.98	1.78	3.38667	1.25865	2	5	3	3	2.12132	10	17	7	14.6667	4.94975	2	3	1	2.33	0.57735	18.9984	106.42	87.4216	48.1389	61.8164	0.5	1.4	0.9	0.8	0.6364	69	102	33	91	23.3345	0	0	0	0	0	0	0
1.69	2.96	1.27	2.53667	0.89803	4	5	1	4.33333	0.70711	12	17	5	15.3333	3.53553	2	3	1	2.33	0.70711	79.904	112.411	32.507	90.7397	22.9859	1.15	1.55	0.4	1.28	0.28284	75	98	23	90.3333	16.2635	7.00E-08	1.00E+18	1.00E+18	6.67E+17	7.07E+17	0	0
0.93	2.05	1.12	1.54333	0.56757	3	5	2	4	1	1	15	14	9.33333	7.37111	1	3	2	2	1	22.9898	121.76	98.7702	70.0529	49.5486	1.35	1.8	0.45	1.53	0.23629	11	88	77	58.3333	41.4287	4.90E-08	4.00E-07	3.51E-07	1.70E-07	2.00E-07	3200	3700
0.93	2.55	1.62	1.79333	0.81525	3	4	1	3.66667	0.57735	1	16	15	9.33333	7.63763	1	3	2	2	1	22.9898	78.96	55.9702	55.1653	28.911	1.15	1.8	0.65	1.43	0.33292	11	93	82	58.6667	42.595	1.00E-08	4.90E-08	3.90E-08	2.54E-08	2.08E-08	3200	3570
0.93	2.1	1.17	1.64333	0.62581	3	5	2	4	1	1	16	15	9.33333	7.63763	1	3	2	2	1	22.9898	127.6	104.61	71.3786	52.7431	1.35	1.8	0.45	1.52	0.24664	11	92	81	58.3333	42.194	1.72E-08	0.0001	1.00E-04	3.34E-05	5.77E-05	2610	3570
0.98	2.18	1.2	1.57667	0.60003	2	4	2	2.66667	1.1547	1	15	14	6	7.81025	1	2	1	1.33	0.57735	6.941	74.9216	67.9806	30.2916	38.6646	1.05	1.45	0.4	1.22	0.20817	12	89	77	59.3333	41.4287	3.80E-08	3.30E-07	2.92E-07	1.54E-07	1.55E-07	0	0
0.82	2.55	1.73	1.75667	0.87386	4	4	0	4	0	1	16	15	9.33333	7.63763	1	3	2	2	1	39.0983	78.96	39.8617	60.5348	20.1007	1.15	2.2	1.05	1.57	0.55752	10	93	83	58.3333	43.1548	1.00E-08	7.50E-08	6.50E-08	3.41E-08	3.56E-08	2000	3570
0.82	2.1	1.28	1.60667	0.68857	4	5	1	4.33333	0.57735	1	16	15	9.33333	7.63763	1	3	2	2	1	39.0983	127.6	88.5017	76.7481	45.704	1.35	2.2	0.85	1.65	0.47697	10	92	82	58	42.7551	1.72E-08	0.0001	1.00E-04	3.34E-05	5.77E-05	2000	3570
0.89	2.58	1.69	2.01667	1.19501	3	6	3	4	2.12132	2	16	14	11.3333	9.89949	1	2	1	1.67	0.70711	32.065	137.327	105.262	67.1523	74.4315	1	2.15	1.15	1.38	0.81317	14	94	80	67.3333	56.5685	3.40E-07	1.00E+23	1.00E+23	6.67E+22	7.07E+22	0	0

[illegible]

1.2	1.61	0.41	1.47333	0.28991	3	8	5	4.66667	3.53553	9	13	4	11.6667	2.82843	2	3
0.95	3.98	3.03	2.53	1.51918	2	5	3	4	1.73205	2	17	15	12	8.66025	1	2
0.98	2.05	1.07	1.56	0.54065	2	5	3	3.66667	1.52753	1	15	14	9.33333	7.37111	1	3
1.1	1.65	0.55	1.46667	0.38891	4	8	4	5.33333	2.82843	12	16	4	13.3333	2.82843	3	3
1.12	2.1	0.98	1.77333	0.69296	5	8	3	6	2.12132	4	16	12	12	8.48528	2	3
0.93	2.58	1.65	1.90333	0.86408	1	3	2	2.33333	1.1547	1	16	15	6	8.66025	1	2
1.3	2.33	1.03	1.815	0.72832	6	9	3	7.5	2.12132	4	14	10	9	7.07107	2	3
1	2.54	1.54	1.976	0.70718	3	6	3	4.4	1.69558	2	14	12	10.4	5.51135	1	3
1.3	3.04	1.74	2.14	0.97558	2	9	7	5.6	3.9211	4	15	11	10.6	6.7361	2	3
1.1	2.2	1.1	1.96333	0.5023	2	8	6	5	2.86039	3	13	10	9.33333	4.28528	2	3
0.82	2.18	1.36	1.33333	0.73874	4	5	1	4.33333	0.57735	1	15	14	6	7.81025	1	2
1.14	1.91	0.77	1.542	0.43254	3	8	5	5.4	2.69258	6	13	7	9	3.3541	2	3
1.3	2.2	0.9	1.904	0.41084	1	9	8	3.8	3.65718	1	10	9	5.2	5.08675	1	3
1.12	1.9	0.78	1.64	0.55154	3	8	5	4.66667	3.53553	4	14	10	10.6667	7.07107	2	3
1.17	2.55	1.38	2.09	0.97581	4	8	4	5.33333	2.82843	8	16	8	13.3333	5.65685	2	3
1.22	1.61	0.39	1.48	0.27577	3	8	5	4.66667	3.53553	12	13	1	12.6667	0.70711	2	3
1.83	2.58	0.75	2.33	0.53033	3	4	1	3.33333	0.70711	8	16	8	13.3333	5.65685	2	3
0.89	2.01	1.12	1.754	0.54185	4	6	2	4.8	0.93541	2	14	12	10.4	5.51135	1	3
1.24	1.61	0.37	1.48667	0.26163	3	8	5	4.66667	3.53553	13	14	1	13.3333	0.70711	2	3
0.82	2.05	1.23	1.52	0.63238	4	5	1	4.66667	0.57735	1	15	14	9.33333	7.37111	1	3
1.61	1.9	0.29	1.70667	0.20506	3	4	1	3.33333	0.70711	11	13	2	12.3333	1.41421	2	3
1.1	2.2	1.1	1.96333	0.5023	2	8	6	4.5	2.63715	3	13	10	8.83333	4.39525	2	3
0.89	2.1	1.21	1.69667	0.8556	5	6	1	5.33333	0.70711	2	16	14	11.3333	9.89949	1	2
0.82	2.54	1.72	2.25333	1.21622	5	6	1	5.83333	0.70711	1	11	10	9.33333	7.07107	1	3
0.82	2.55	1.73	1.97	0.99594	4	6	2	5	1	1	16	15	9.33333	7.63763	1	3
0.89	3.44	2.55	2.28667	1.3133	2	6	4	3.83333	1.97094	2	17	15	10.6667	7.55493	1	3
1.27	1.9	0.63	1.57	0.31607	3	8	5	5	2.64575	4	17	13	11.6667	6.80686	2	3
1.14	2.2	1.06	1.97	0.48357	2	8	6	4.5	2.63715	6	13	7	9.33333	3.43776	2	3
1.14	2.2	1.06	1.72833	0.47666	3	8	5	4.5	2.30612	6	13	7	10.8333	3.25437	2	3
1.17	2.2	1.03	1.975	0.46953	2	8	6	4.5	2.63715	8	13	5	9.66667	3.01511	2	3
1.62	3.44	1.82	2.22667	1.28693	2	6	4	4.66667	2.82843	13	16	3	14	2.12132	2	2
0.95	2.54	1.59	2.01	1.1243	5	6	1	5.66667	0.70711	2	11	9	8	6.36396	1	3
1.91	2.28	0.37	2.12667	0.19296	3	5	2	4	1	9	15	6	11.3333	3.21455	2	3
0.82	2	1.18	1.60667	0.83439	5	6	1	5.66667	0.70711	1	12	11	8.33333	7.77817	1	3
1.5	2.55	1.05	1.85	0.74246	4	6	2	5.33333	1.41421	5	16	11	8.66667	7.77817	2	3
1.12	3.44	2.32	2.34	1.30557	2	8	6	4.6	3.5	4	16	12	11.2	7.34847	2	3
0.79	2.1	1.31	1.27333	0.71933	3	6	3	4.66667	1.52753	1	16	15	6	8.66025	1	2
1.22	1.9	0.68	1.55333	0.3402	3	5	2	4	1	3	14	11	7	6.08276	2	3
1.25	1.96	0.71	1.50667	0.39374	3	8	5	5.33333	2.51661	2	15	13	10.3333	7.23418	1	3
2.19	2.36	0.17	2.24667	0.12021	3	6	3	4	2.12132	6	15	9	12	6.36396	2	3
1.27	1.61	0.34	1.49667	0.24042	3	8	5	4.66667	3.53553	13	17	4	14.3333	2.82843	2	3
0.93	3.44	2.51	2.60333	1.77484	2	3	1	2.33333	0.70711	1	16	15	11	10.6066	1	2
1.1	2.2	1.1	1.81667	0.479	4	8	4	4.83333	2.06828	10	16	6	11.6667	2.78887	3	3
1.3	2.2	0.9	1.99667	0.40892	2	9	7	5.16667	3.16946	4	13	9	9.5	3.89055	2	3
1.23	1.61	0.38	1.48333	0.2687	3	8	5	4.66667	3.53553	13	13	0	13	0	2	3
1.66	3.44	1.78	2.84667	1.25865	2	4	2	2.66667	1.41421	6	16	10	12.6667	7.07107	2	3
1.36	2.2	0.84	1.956	0.38735	4	9	5	5.4	2.3184	7	14	7	11	3.3541	2	3
1.12	2.54	1.42	1.90333	0.72127	5	8	3	6.33333	1.52753	4	15	11	10	5.56776	2	3
1.13	2.2	1.07	1.96833	0.48825	2	8	6	5	2.86039	5	13	8	9.66667	3.5162	2	3
1.9	2.58	0.68	2.35333	0.48083	3	4	1	3.33333	0.70711	11	16	5	14.3333	3.53553	2	3
1.17	1.31	0.14	1.26333	0.09899	3	8	5	4.66667	3.53553	2	8	6	4	4.24264	1	3
0.79	2.28	1.49	1.612	0.84296	5	6	1	5.6	0.61237	1	16	15	8.8	8.41873	1	3
1.2	1.9	0.7	1.78333	0.49497	4	8	4	4.66667	2.82843	10	11	1	10.8333	0.70711	3	3
1.13	1.31	0.18	1.25	0.12728	3	8	5	4.66667	3.53553	2	5	3	3	2.12132	1	3
1.24	1.65	0.41	1.5	0.22605	3	8	5	5	2.64575	12	14	2	13	1	2	3
1.57	2.54	0.97	1.73167	0.68589	2	6	4	2.66667	2.82843	2	11	9	3.5	6.36396	1	3
0.95	1.62	0.67	1.39667	0.47376	5	6	1	5.66667	0.70711	2	13	11	9.33333	7.77817	1	2
0.82	2.18	1.36	1.56333	0.68879	4	5	1	4.33333	0.57735	1	15	14	9.33333	7.37111	1	3
1.61	3.44	1.83	2.925	1.05596	2	4	2	2.5	1.08012	13	16	3	15.3333	1.56347	2	2
1.83	2.55	0.72	2.31	0.50912	4	4	0	4	0	8	16	8	13.3333	5.65685	2	3
1.61	3.44	1.83	2.92667	1.05362	2	3	1	2.33333	0.66667	13	16	3	15.3333	1.56347	2	2
1.22	2.2	0.98	1.98333	0.44618	2	5	3	4	1.80907	3	13	10	8.83333	4.39525	2	3
1.1	1.9	0.8	1.76667	0.56569	4	8	4	4.66667	2.82843	11	16	5	11.8333	3.53553	3	3
1.12	3.44	2.32	2.228	1.30342	2	8	6	5.2	3.39116	4	16	12	11	7.15891	2	3
0.89	2.55	1.66	1.99667	1.1738	2	6	4	3.33333	2.82843	2	14	12	10	8.48528	1	2
1.24	2.28	1.04	1.87	0.59521	3	8	5	5.66667	2.29624	9	14	5	11.5	3.19801	2	3
1.38	3.04	1.66	2.36	0.86971	2	9	7	5.33333	3.51188	6	17	11	12.6667	5.85947	2	3
2.19	2.36	0.17	2.24667	0.12021	3	6	3	4	2.12132	6	15	9	12	6.36396	2	3
2.28	2.33	0.05	2.305	0.03536	5	6	1	5.5	0.70711	9	14	5	11.5	3.53553	2	3
1.1	1.61	0.51	1.44	0.36062	3	8	5	4.66667	3.53553	3	13	10	9.66667	7.07107	2	3
1.22	1.65	0.43	1.57833	0.30406	4	5	1	4.16667	0.70711	3	12	9	10.5	6.36396	3	3
1.91	2.18	0.27	2.09	0.19092	4	4	0	4	0	10	15	5	13.3333	3.53553	2	3
1.33	1.36	0.03	1.35	0.02121	4	5	1	4.33333	0.70711	3	4	1	3.33333	0.70711	3	3
1.38	2.19	0.81	1.92	0.57276	3	9	6	5	4.24264	6	15	9	12	6.36396	2	3
0.82	2.18	1.36	1.51667	0.68061	4	4	0	4	0	1	15	14	7.66667	7.02377	1	3
1.3	2.55	1.25	1.96667	0.62915	4	9	5	6	2.64575	4	16	12	11.6667	6.65833	2	3
0.82	2.18	1.36	1.43667	0.68879	3	4	1	3.66667	0.57735	1	15	14	6	7.81025	1	2
0.79	2	1.21	1.59667	0.8556	6	6	0	6	0	1	12	11	8.33333	7.77817	1	3
1.1	2.28	1.18	1.85333	0.65432	4	8	4	5.66667	2.08167	3	15	12	9	6	2	3
1.13	2.2	1.07	1.96833	0.48825	2	8	6	4.5	2.63715	5	13	8	9.16667	3.72339	2	3
1.1	2.2	1.1	1.904	0.5136	4	8	4	5.2	1.83712	8	16	8	12	4.1833	2	3
1.5	3.44	1.94	2.59333	1.00537	2	6	4	3.33333	2.41209	5	16	11	11.8333	6.19751	2	3
1.66	3.16	1.5	2.66	1.06066	3	4	1	3.33333	0.70711	6	17	11	13.3333	7.77817	2	3
1	2.28	1.28	2.06667	0.9051	4	6	2	5.66667	1.41421	2	10	8	8.66667	5.65685	1	3
1.13	2.28	1.15	2.00833	0.5209	2	8	6	4.5	2.63715	5	13	8	9.66667	3.5162	2	3
1.54	1.9	0.36	1.78	0.25456	3	4	1	3.33333	0.70711	4	14	10	10.6667	7.07107	2	3
1.25	1.96	0.71	1.71333	0.40154	5	8	3	6	1.73205	11	15	4	13.3333	2.08167	2	3
0.82	2.58	1.76	1.44333	0.98592	3	5	2	3.66667	1.1547	1	16	15	6	8.66025	1	2
0.79	2.55	1.76	1.96	1.01326	4	6	2	5.33333	1.1547	1	16	15	9.33333	7.63763	1	3
2	2.66	0.66	2.44	0.46669	5	6	1	5.33333	0.70711	12	17	5	15.3333	3.53553	2	3
1.55	1.81	0.26	1.635	0.13431	4	4	0	4	0	5	13	8	8	3.79473	2	3
1.12	2.54	1.42	1.87333	0.71396	5	8	3	6.33333	1.52753	4	14	10	9.66667	5.1316	2	3
1.1	1.9	0.8	1.63333	0.56569	3	8	5	4.66667	3.53553	3	14	11	10.3333	7.77817	2	3
1.24	1.81	0.57	1.56667	0.294	4	8	4	5.33333	2.3094	12	14	2	13	1	2	3
1.14	1.96	0.82	1.47	0.43278	3	8	5	5.33333	2.51661	2	14	12	7.33333	6.1101	1	3
1.23	2.58	1.35	2.13	0.95459	3	8	5	4.66667	3.53553	13	16	3	15	2.12132	2	3
2.55	3.44	0.89	3.14333	0.62933	2	2	0	2	0	14	16	2	15.3333	1.41421	2	2
1.1	2.1	1	1.76667	0.70711	5	8	3	6	2.12132	11	16	5	14.3333	3.53553	2	3
0.89	2.33	1.44	1.62333	0.72037	4	6	2	5.33333	1.1547	2	14	12	9.33333	6.4291	1	3
1.12	1.9	0.78	1.645	0.33265	4	8	4	4.66667	1.90693	4	12	8	10.3333	3.66804	3	3
1.1	1.65	0.55	1.46667	0.38891	4	8	4	5.33333	2.82843	11	12	1	11.6667	0.70711	3	3
1.1	1.9	0.8	1.51667	0.40104	3	8	5	5	2.64575	3	14	11	8	5.56776	2	3
1.81	1.91	0.1	1.87667	0.07071	4	4	0	4	0	10	13	3	11	2.12132	2	3
1.22	1.65	0.43	1.49333	0.23756	3	8	5	5	2.64575	12	13	1	12.3333	0.57735	2	3
2.16	3.44	1.28	3.01333	0.9051	2	5	3	3	2.12132	6	16	10	12.6667	7.07107	2	3
1.91	2.04	0.13	1.95333	0.09192	2	4	2	3.33333	1.41421	10	13	3	11	2.12132	2	3
1.14	3.98	2.84	2.55667	1.42001	2	8	6	4.66667	3.05505	6	17	11	13	6.08276	2	3
1.22	1.96	0.74	1.69333	0.41102	4	8	4	5.66667	2.08167	11	14	3	12.3333	1.52753	2	3
1.24	2.55	1.31	2.11333	0.92631	4	8	4	5.33333	2.82843	14	16	2	15.3333	1.41421	2	3
1.22	2.05	0.83	1.54333	0.44433	4	8	4	5.66667	2.08167	3	15	12	10	6.245	2	3
0.95	2.18	1.23	1.67667	0.6447	4	5	1	4.33333	0.57735	2	15	13	9.33333	6.65833	1	3
1.2	2.2	1	1.86667	0.70711	5	8	3	6	2.12132	8	10	2	8.66667	1.41421	3	3
1.23	1.9	0.67	1.78833	0.47376	4	8	4	4.66667	2.82843	11	13	2	11.3333	1.41421	3	3
1.17	2.1	0.93	1.79	0.65761	5	8	3	6	2.12132	8	16	8	13.3333	5.65685	2	3
1.2	3.44	2.24	2.53333	1.17938	2	8	6	4.66667	3.05505	10	17	7	14.3333	3.78594	2	3
1.25	2.1	0.85	1.81667	0.60104	5	8	3	6	2.12132	15	16	1	15.6667	0.70711	2	3
1.3	1.91	0.61	1.65833	0.26735	3	9	6	4.33333	2.7303	4	13	9	10.5	4.09545	2	3
0.82	2.54	1.72	1.82	0.89353	5	6	1	5.33333	0.57735	1	16	15	9.33333	7.63763	1	3
0.89	2.18	1.29	1.67	0.6014	4	6	2	4.4	1	2	15	13	9.2	6.34429	1	3
1.24	2.58	1.34	2.13333	0.94752	3	8	5	4.66667	3.53553	14	16	2	15.3333	1.41421	2	3
1.12	2.55	1.43	1.79833	0.50017	2	8	6	4	2.2532	4	14	10	9.66667	5.15901	2	3
1.14	1.91	0.77	1.63167	0.3295	3	8	5	4.16667	2.26635	6	13	7	10.8333	3.25437	2	3
1.3	1.65	0.35	1.53333	0.24749	4	6	2	4.66667	1.41421	4	12	8	9.33333	5.65685	3	3
1.2	2.54	1.34	2.31667	0.94752	6	8	2	6.33333	1.41421	9	11	2	10.6667	1.41421	3	3
0.89	2.01	1.12	1.51667	0.57178	4	6	2	4.66667	1.1547	2	14	12	9.33333	6.4291	1	3
0.79	2.54	1.75	1.81	0.91033	5	6	1	5.66667	0.57735	1	16	15	9.33333	7.63763	1	3
1.1	1.9	0.8	1.63333	0.46188	3	8	5	5	2.64575	11	16	5	13.6667	2.51661	2	3
1.25	1.96	0.71	1.70333	0.39374	4	8	4	5.66667	2.08167	11	15	4	13.3333	2.08167	2	3
1.13	2.55	1.42	1.93	0.49211	2	8	6	3.66667	2.51151	5	14	9	10.8333	3.55722	2	3
1	2.02	1.02	1.65	0.56471	4	6	2	5	1	2	15	13	9.33333	6.65833	1	3
1	2.05	1.05	1.35	0.74246	4	5	1	4.33333	0.70711	2	15	13	6.33333	9.19239	1	2
1	2.05	1.05	1.65	0.56789	4	5	1	4.33333	0.57735	2	15	13	9.33333	6.65833	1	3
1.2	2.2	1	1.73833	0.45691	3	8	5	4.5	2.30612	10	13	3	11.5	1.91881	2	3
1.1	2.05	0.95	1.68333	0.51072	4	8	4	5.66667	2.08167	11	16	5	14	2.64575	2	3
1.25	1.69	0.44	1.54333	0.31113	5	8	3	6	2.12132	12	15	3	13	2.12132	3	3
1.1	3.44	2.34	2.504	1.65463	2	8	6	4.4	4.24264	3	16	13	10.8	9.19239	2	3
1.3	2.01	0.71	1.796	0.32592	4	9	5	5	2.5	4	14	10	9.6	4.84768	2	3
1.14	3.44	2.3	2.236	1.29281	2	8	6	5.2	3.39116	6	16	10	11.8	5.93717	2	3
2.02	3.44	1.42	2.694	0.79889	2	6	4	4	2.23607	15	16	1	15.6	0.61237	2	2
1.13	3.44	2.31	2.232	1.29811	2	8	6	5.2	3.39116	5	16	11	11.4	6.5479	2	3
1.13	2.01	0.88	1.65	0.41443	4	8	4	4.8	2	5	14	9	9.4	4.78278	2	3
1.2	2.18	0.98	1.844	0.44753	4	8	4	4.8	2	8	15	7	11	4.10792	2	3
1.13	1.91	0.78	1.538	0.43835	3	8	5	5.4	2.69258	5	13	8	8.6	3.9211	2	3
1.17	2.01	0.84	1.658	0.39894	4	8	4	4.8	2	7	14	7	10	4.10792	2	3
1.6	2.58	0.98	2.182	0.59416	2	5	3	3.6	1.5	5	16	11	11.2	6.39336	2	3
1.2	2.01	0.81	1.664	0.38759	4	8	4	4.8	2	7	14	7	10.2	3.98434	2	3
1.14	3.04	1.9	1.684	1.00049	2	8	6	6.2	3.20713	6	15	9	9.4	5.58058	2	3
1.2	2.18	0.98	1.732	0.48434	4	8	4	4.8	2	7	15	8	10.6	4.58258	2	3
1.27	2.58	1.31	2.046	0.61622	3	8	5	4.4	2.3184	11	17	6	14.2	3.29773	2	3
1.81	2.28	0.47	1.998	0.33234	4	6	2	4.8	1.41421	10	13	3	11.8	2.12132	2	3
1	3.04	2.04	1.816	1.4425	2	4	2	3.2	1.41421	2	15	13	7.2	9.19239	1	2
1.3	1.9	0.6	1.5	0.42426	4	9	5	7.33333	3.53553	4	11	7	6.33333	4.94975	3	3
1.6	2.58	0.98	2.182	0.59416	2	5	3	3.6	1.5	5	16	11	11.2	6.39336	2	3
1.2	3.04	1.84	1.684	0.95445	2	8	6	6.2	3.20713	10	15	5	11.6	2.75162	2	3
0.95	2.18	1.23	1.682	0.57735	4	5	1	4.2	0.5	2	15	13	9.2	6.34429	1	3
1.2	2.01	0.81	1.828	0.42278	4	8	4	4.8	2.13809	9	14	5	12.2	2.9761	2	3
0.93	3.44	2.51	2.782	1.3016	2	5	3	2.8	1.55839	1	17	16	13.2	8.16788	1	2
1.83	2.19	0.36	2.07	0.25456	3	4	1	3.33333	0.70711	8	15	7	12.6667	4.94975	2	3
1.55	3.44	1.89	2.81	1.33643	2	4	2	2.66667	1.41421	7	16	9	13	6.36396	2	3
1.9	2.55	0.65	2.33333	0.45962	4	4	0	4	0	11	16	5	14.3333	3.53553	2	3
0.89	3.98	3.09	2.61	1.57445	2	6	4	4	2	2	17	15	12	8.66025	1	2
1.17	2.54	1.37	2.01333	0.73786	6	8	2	6.66667	1.1547	8	14	6	11	3	2	3

0.82	2.19	1.37	1.52	0.68549	3	4	1	3.66667	0.57735	1	15	14	7.66667	7.02377	1	3
1.14	3.04	1.9	1.616	0.98335	2	8	6	6.4	3.11677	6	15	9	9.2	5.30498	2	3
1.9	3.16	1.26	2.93	0.65437	2	6	4	3.33333	1.76383	7	17	10	15	5.16398	2	3
1.1	2.2	1.1	1.77	0.58796	4	8	4	5.66667	2.08167	3	14	11	8.33333	5.50757	2	3
0.82	2.05	1.23	1.47333	0.61857	4	5	1	4.66667	0.57735	1	15	14	7.66667	7.02377	1	3
1.13	1.9	0.77	1.64333	0.54447	6	8	2	6.66667	1.41421	5	7	2	6.33333	1.41421	3	3
0.79	3.98	3.19	2.57667	1.62918	2	6	4	4	2	1	17	16	11.6667	9.2376	1	2
1.13	1.9	0.77	1.77167	0.54447	4	8	4	4.66667	2.82843	5	11	6	10	4.24264	3	3
1.3	2.05	0.75	1.8	0.53033	5	6	1	5.33333	0.70711	4	15	11	11.3333	7.77817	2	3
0.82	2.02	1.2	1.38333	0.60335	3	6	3	4.33333	1.52753	1	15	14	6	7.81025	1	2
1.1	2.58	1.48	2.08667	1.04652	3	8	5	4.66667	3.53553	11	16	5	14.3333	3.53553	2	3
1.22	2.01	0.79	1.59	0.39737	4	5	1	4.33333	0.57735	3	14	11	7	6.08276	2	3
1.3	1.9	0.6	1.61	0.3005	3	6	3	4.33333	1.52753	4	14	10	7.66667	5.50757	2	3
1.12	1.91	0.79	1.77833	0.55861	4	8	4	4.66667	2.82843	4	10	6	9	4.24264	3	3
1.22	2.01	0.79	1.59	0.39737	4	8	4	5.33333	2.3094	4	14	10	10	5.2915	2	3
1.14	2.2	1.06	1.97	0.48357	2	8	6	5	2.86039	6	13	7	9.83333	3.16946	2	3

[illegible]

1.17	2.55	1.38	2.06	0.51061	2	8	6	4	2.63117	8	14	6	11	2.8011	2	3	1	[illegible]
1.17	2.54	1.37	1.89	0.68768	5	8	3	6.33333	1.52753	8	14	6	11	3	2	3	1	[illegible]
0.98	2.19	1.21	1.71333	0.57122	2	8	6	4	2.2532	1	15	14	9.5	6.21722	1	3	2	[illegible]
1.17	2.2	1.03	1.85	0.58898	4	8	4	5.66667	2.08167	8	15	7	11	3.60555	2	3	1	[illegible]
1.2	2.55	1.35	2.065	0.49857	2	8	6	4	2.63117	9	14	5	11.3333	2.41788	2	3	1	[illegible]
1.2	2.54	1.34	1.9	0.67201	5	8	3	6.33333	1.52753	10	14	4	11.6667	2.08167	2	3	1	[illegible]
1.83	2.55	0.72	2.07	0.50912	2	4	2	3.33333	1.41421	8	14	6	10	4.24264	2	3	1	[illegible]
1.1	2.55	1.45	2.04833	0.53891	2	8	6	4	2.63117	9	14	5	11.5	2.32875	2	3	1	[illegible]
1.6	2.18	0.58	1.93	0.29816	4	5	1	4.33333	0.57735	5	15	10	11.3333	5.50757	2	3	1	[illegible]
1.22	2.55	1.33	2.06833	0.49058	2	8	6	4	2.63117	9	14	5	11.6667	2.32048	2	3	1	[illegible]
1.6	2.18	0.58	1.98667	0.41012	4	5	1	4.33333	0.70711	5	15	10	11.6667	7.07107	2	3	1	[illegible]
0.89	2.02	1.13	1.60333	0.62067	4	6	2	5.33333	1.1547	2	15	13	9.33333	6.65833	1	3	2	[illegible]
1.22	2.55	1.33	2.06833	0.49058	2	5	3	3.5	1.76505	3	14	11	10.1667	4.42458	2	3	1	[illegible]
0.95	2.2	1.25	1.78333	0.88388	5	5	0	5	0	2	10	8	7.33333	5.65685	1	3	2	[illegible]
0.89	2.01	1.12	1.53	0.57689	4	6	2	5	1	2	14	12	9.33333	6.4291	1	3	2	[illegible]
1.2	2.18	0.98	1.76	0.50478	4	8	4	5.33333	2.3094	9	15	6	11.6667	3.05505	2	3	1	[illegible]
[illegible]																		

2.55	3.44	0.89	3.14333	0.62933	2	2	0	2	0	14	16	2	15.3333	1.41421	2	2	0	2	0	12.0107	15.9994	3.9887
1.2	2.01	0.81	1.875	0.57276	4	8	4	4.66667	2.82843	10	14	4	13.3333	2.82843	2	3	1	2.17	0.70711	72.64	157.25	84.61
1.9	2.58	0.68	2.35333	0.48083	3	3	0	3	0	14	16	2	15.3333	1.41421	2	2	0	2	0	28.0855	32.065	3.9795
1.1	2.2	1.1	1.83333	0.77782	6	8	2	6.66667	1.41421	3	9	6	7	4.24264	3	3	0	3	0	138.905	192.217	53.3115
2.36	2.58	0.22	2.50667	0.15556	3	6	3	4	2.12132	6	16	10	12.6667	7.07107	2	3	1	2.33	0.70711	32.065	183.84	151.775
1.23	2.01	0.78	1.71333	0.42218	4	8	4	5.33333	2.3094	11	14	3	12.6667	1.52753	2	3	1	2.67	0.57735	63.546	164.93	101.384
1.33	2.55	1.22	1.92667	0.61044	3	5	2	4	1	4	16	12	11.3333	6.4291	2	3	1	2.33	0.57735	28.0855	91.224	63.1385
1.12	2.01	0.89	1.67667	0.48521	4	8	4	5.33333	2.3094	4	14	10	9.66667	5.1316	2	3	1	2.67	0.57735	63.546	140.116	76.57
2.54	2.58	0.04	2.55333	0.02828	3	6	3	5	2.12132	11	16	5	12.6667	3.53553	2	3	1	2.67	0.70711	32.065	196.967	164.902
1.2	1.65	0.45	1.48667	0.24906	3	8	5	5	2.64575	10	13	3	11.6667	1.52753	2	3	1	2.67	0.57735	26.9815	157.25	130.268
1.22	2.33	1.11	1.81667	0.55967	4	8	4	6	2	11	14	3	12.3333	1.52753	2	3	1	2.67	0.57735	63.546	207.2	143.654
1.1	2.2	1.1	2.01667	0.77782	6	8	2	6.33333	1.41421	3	9	6	8	4.24264	3	3	0	3	0	138.905	192.217	53.3115
1.12	2.2	1.08	1.825	0.46091	4	8	4	5	1.80907	4	13	9	10.5	4.09545	2	3	1	2.5	0.6396	69.723	140.116	70.393
1.27	2.2	0.93	1.89	0.65761	6	8	2	6.66667	1.41421	9	17	8	11.6667	5.65685	3	3	0	3	0	174.967	192.217	17.25
0.89	2.96	2.07	1.89	0.96764	1	6	5	3.16667	2.89984	1	17	16	4	7.45898	1	2	1	1.17	0.47673	1.00794	137.327	136.319
1.5	2.28	0.78	2.02	0.55154	6	6	0	6	0	5	10	5	8.33333	3.53553	3	3	0	3	0	180.948	195.084	14.1361
1.14	2.1	0.96	1.76333	0.5404	5	8	3	6	1.73205	6	16	10	12.3333	5.50757	2	3	1	2.33	0.57735	121.76	144.242	22.482
1.13	2.2	1.07	1.84	0.6149	3	8	5	5.33333	2.51661	5	15	10	10	5	2	3	1	2.67	0.57735	30.9738	140.908	109.934
1.12	2.1	0.98	1.75667	0.55194	5	8	3	6	1.73205	4	16	12	11.6667	6.65833	2	3	1	2.33	0.57735	121.76	140.116	18.356
1.33	2.58	1.25	1.93667	0.62581	3	5	2	3.66667	1.1547	4	16	12	11.3333	6.4291	2	3	1	2.33	0.57735	28.0855	91.224	63.1385
1.38	1.9	0.52	1.81333	0.3677	4	9	5	4.83333	3.53553	6	11	5	10.1667	3.53553	3	3	0	3	0	63.546	238.029	174.483
1.55	2.18	0.63	1.76	0.44548	4	4	0	4	0	7	15	8	9.66667	5.65685	2	3	1	2.67	0.70711	54.938	74.9216	19.9836
1.36	2.54	1.18	1.93333	0.59071	3	6	3	4.33333	1.52753	3	14	11	9.33333	5.68624	2	3	1	2.67	0.57735	28.0855	196.967	168.881
1.1	1.9	0.8	1.73167	0.41396	4	8	4	4.83333	2.06828	11	16	5	12	2.58199	3	3	0	3	0	63.546	173.04	109.494
1.12	1.31	0.19	1.24667	0.13435	3	8	5	4.66667	3.53553	2	4	2	2.66667	1.41421	1	3	2	1.67	1.41421	24.305	140.116	115.811
1.93	3.44	1.51	3.14167	0.77985	2	5	3	2.66667	1.56347	11	17	6	15.3333	2.78887	2	3	1	2.17	0.52705	15.9994	107.868	91.8688
1.5	2.18	0.68	1.95333	0.48083	4	6	2	4.66667	1.41421	5	15	10	11.6667	7.07107	2	3	1	2.33	0.70711	74.9216	180.948	106.026
1.33	2.1	0.77	1.77667	0.39954	3	5	2	4.33333	1.1547	4	16	12	11.3333	6.4291	2	3	1	2.33	0.57735	28.0855	127.6	99.5145
1.33	2.1	0.77	1.81333	0.42099	4	5	1	4.66667	0.57735	4	16	12	11.3333	6.4291	2	3	1	2.33	0.57735	72.64	127.6	54.96
1.13	2.2	1.07	1.84333	0.7566	6	8	2	6.66667	1.41421	5	9	4	7.66667	2.82843	3	3	0	3	0	140.908	192.217	51.3094
1.93	3.44	1.51	2.43333	1.06773	2	5	3	4	2.12132	11	16	5	12.6667	3.53553	2	3	1	2.67	0.70711	15.9994	107.868	91.8688
1.23	1.69	0.46	1.53667	0.32527	5	8	3	6	2.12132	12	13	1	12.3333	0.70711	3	3	0	3	0	112.411	164.93	52.5193
0.89	3.44	2.55	2.385	1.37859	2	6	4	3.33333	1.90693	2	16	14	10.6667	7.14779	1	3	2	2.17	0.87905	15.9994	137.327	121.328
1.33	3.04	1.71	2.34333	0.8979	2	5	3	4	1.73205	4	17	13	12	7	2	3	1	2.33	0.57735	14.0067	126.904	112.898
1.22	2.2	0.98	1.98333	0.44618	2	6	4	4.5	2.30612	3	13	10	9.33333	4.28528	2	3	1	2.67	0.60302	10.811	192.217	181.406
1.61	2.2	0.59	1.80667	0.41719	3	5	2	3.66667	1.41421	8	13	5	11.3333	3.53553	2	3	1	2.33	0.70711	26.9815	101.07	74.0885
1.33	2.05	0.72	1.79667	0.40464	4	5	1	4.66667	0.57735	4	15	11	11	6.08276	2	3	1	2.33	0.57735	72.64	121.76	49.12
1.1	2.28	1.18	1.88667	0.83439	6	8	2	6.66667	1.41421	10	11	1	10.3333	0.70711	3	3	0	3	0	158.925	195.084	36.1586
1.1	2.2	1.1	1.79333	0.46563	2	8	6	4.33333	2.32048	3	13	10	9.66667	4.38529	2	3	1	2.5	0.58835	10.811	138.905	128.094
1.38	2.58	1.2	2.05	0.61213	3	9	6	5	3.4641	6	16	10	12.3333	5.50757	2	3	1	2.33	0.57735	30.9738	238.029	207.055
0.93	3.04	2.11	2.17333	1.10428	2	3	1	2.33333	0.57735	1	15	14	10	7.81025	1	2	1	1.67	0.57735	12.0107	22.9898	10.9791
0.93	2.02	1.09	1.5	0.54672	3	6	3	4.33333	1.52753	1	15	14	7.66667	7.02377	1	3	2	2	1	22.9898	208.98	185.991
1.12	2.55	1.43	2.025	0.51658	2	8	6	4.33333	2.85549	4	14	10	10.3333	4.05728	2	3	1	2.5	0.58835	10.811	192.217	181.406
1.1	2.1	1	1.75	0.56347	5	8	3	6	1.73205	3	16	13	11.3333	7.23418	2	3	1	2.33	0.57735	121.76	138.905	17.1455
0.95	1.61	0.66	1.39	0.46669	3	5	2	3.66667	1.41421	2	13	11	9.33333	7.77817	1	2	1	1.67	0.70711	26.9815	87.62	60.6385
0.95	1.61	0.66	1.39	0.46669	3	5	2	3.66667	1.41421	2	13	11	9.33333	7.77817	1	2	1	1.67	0.70711	26.9815	87.62	60.6385
1.6	2.18	0.58	1.89333	0.29006	3	5	2	4	1	5	15	10	11.3333	5.50757	2	3	1	2.33	0.57735	28.0855	92.9064	64.8209
1.36	2.2	0.84	1.92	0.59397	4	6	2	5.33333	1.41421	3	9	6	7	4.24264	3	3	0	3	0	44.9559	192.217	147.261
1.1	1.96	0.86	1.57	0.43555	4	8	4	5.66667	2.08167	12	16	4	14	2	2	3	1	2.67	0.57735	65.409	173.04	107.631
1.33	2.55	1.22	1.96333	0.61134	4	5	1	4.33333	0.57735	4	16	12	11.3333	6.4291	2	3	1	2.33	0.57735	72.64	91.224	18.584
1.17	1.61	0.44	1.46333	0.31113	3	8	5	4.66667	3.53553	8	13	5	11.3333	3.53553	2	3	1	2.33	0.70711	26.9815	150.36	123.378
1.9	2.04	0.14	1.99333	0.09899	2	5	3	3	2.12132	7	13	6	11	4.24264	2	3	1	2.33	0.70711	10.811	98	87.189
0.82	3.04	2.22	2.44333	0.88799	2	5	3	2.83333	1.42775	1	17	16	12.6667	6.25115	1	2	1	1.83	0.43853	12.0107	126.904	114.894

1.12	2.2	1.08	1.74	0.55749	3	8	5	5.33333	2.51661	4	14	10	8.66667	5.03322	2	3	1	2.67	0.57735	28.0855	140.116	112.031	89.7572	56.8656	1.1	1.85	0.75	1.42	0.38837
1.3	2.55	1.25	2.01333	0.64345	3	9	6	5.33333	3.21455	4	16	12	11.6667	6.65833	2	3	1	2.33	0.57735	30.9738	232.038	201.064	113.991	105.01	1	1.8	0.8	1.32	0.42525
1.2	1.9	0.7	1.78333	0.49497	4	8	4	4.66667	2.82843	9	11	2	10.6667	1.41421	3	3	0	3	0	63.546	151.964	88.418	78.2823	62.521	1.35	1.85	0.5	1.43	0.35355
1.25	2.54	1.29	1.91667	0.64609	5	8	3	6.33333	1.52753	11	15	4	13.3333	2.08167	2	3	1	2.67	0.57735	118.71	196.967	78.2566	161.537	39.6492	1.35	1.75	0.4	1.52	0.20817
1.25	1.9	0.65	1.58667	0.32563	3	8	5	4.66667	2.88675	13	15	2	14	1	2	3	1	2.33	0.57735	26.9815	168.934	141.953	74.6671	81.6396	1.1	1.75	0.65	1.37	0.34034
1.3	2.01	0.71	1.53667	0.50205	4	6	2	5.33333	1.41421	4	14	10	7.33333	7.07107	2	3	1	2.67	0.70711	72.64	178.49	105.85	143.207	74.8473	1.25	1.55	0.3	1.45	0.21213
1.91	2.19	0.28	2.09667	0.19799	3	4	1	3.33333	0.70711	10	15	5	13.3333	3.53553	2	3	1	2.33	0.70711	30.9738	58.6934	27.7196	40.2136	19.6007	1	1.35	0.35	1.12	0.24749
1.31	3.98	2.67	3.09	1.88798	2	3	1	2.33333	0.70711	2	17	15	12	10.6066	1	2	1	1.67	0.70711	18.9984	24.305	5.3066	20.7673	3.75233	0.5	1.5	1	0.83	0.70711
1.22	1.9	0.68	1.75667	0.3113	4	8	4	4.66667	1.90693	11	13	2	11.8333	1.14812	2	3	1	2.67	0.60302	63.546	162.5	98.954	82.0973	46.1321	1.3	1.75	0.45	1.4	0.20226
1.57	1.93	0.36	1.69	0.25456	2	5	3	3	2.12132	2	11	9	5	6.36396	1	3	2	1.67	1.41421	9.01218	107.868	98.856	41.9642	69.9018	1.05	1.6	0.55	1.23	0.38891
0.95	3.44	2.49	2.51167	1.25472	2	5	3	3.16667	1.55212	2	16	14	12	6.39602	1	3	2	2.17	0.87905	15.9994	87.62	71.6206	43.785	36.9961	0.6	2	1.4	1.08	0.67756
0.93	2.54	1.61	1.82667	0.82051	3	6	3	4.33333	1.52753	1	14	13	8.66667	6.80686	1	3	2	2	1	22.9898	196.967	173.977	97.5321	89.6197	1.25	1.8	0.55	1.47	0.29297
0.93	3.44	2.51	1.7	1.54863	2	3	1	2.66667	0.66667	1	16	15	5.83333	9.67528	1	2	1	1.33	0.66667	14.0067	22.9898	8.98307	20.3275	5.38627	0.6	1.8	1.2	1.41	0.7836
1.2	2.28	1.08	1.88667	0.59677	4	8	4	6	2	9	15	6	11.3333	3.21455	2	3	1	2.67	0.57735	74.9216	195.084	120.162	140.657	60.874	1.15	1.85	0.7	1.45	0.36056
0.89	1.93	1.04	1.75667	0.73539	5	6	1	5.16667	0.70711	2	11	9	9.5	6.36396	1	3	2	2.67	1.41421	107.868	137.327	29.4588	112.778	20.8305	1.6	2.15	0.55	1.69	0.38891
1.1	2.01	0.91	1.55333	0.45501	4	8	4	5.33333	2.3094	3	14	11	8	5.56776	2	3	1	2.67	0.57735	54.938	138.905	83.9674	88.8278	44.2625	1.25	1.95	0.7	1.53	0.36856
1.1	1.83	0.73	1.58667	0.51619	4	8	4	5.33333	2.82843	8	11	3	9	2.12132	3	3	0	3	0	55.845	158.925	103.08	90.2051	72.8888	1.4	1.75	0.35	1.52	0.24749
1.22	2.58	1.36	2.12667	0.96167	3	8	5	4.66667	3.53553	12	16	4	14.6667	2.82843	2	3	1	2.33	0.70711	32.065	162.5	130.435	75.5433	92.2315	1	1.75	0.75	1.25	0.53033
1.23	3.44	2.21	2.61	1.20329	2	8	6	4.33333	3.21455	13	17	4	15.3333	2.08167	2	3	1	2.33	0.57735	15.9994	164.93	148.931	72.1276	80.956	0.6	1.75	1.15	1.12	0.58381
1.5	2.04	0.54	1.68	0.38184	2	6	4	4.66667	2.82843	5	13	8	7.66667	5.65685	2	3	1	2.67	0.70711	10.811	180.948	170.137	124.236	120.305	0.85	1.45	0.6	1.25	0.42426
0.98	1	0.02	0.98667	0.01414	2	4	2	2.66667	1.41421	1	2	1	1.33333	0.70711	1	1	0	1	0	6.941	40.078	33.137	17.9867	23.4314	1.45	1.8	0.35	1.57	0.24749
1.5	1.9	0.4	1.63333	0.28284	3	6	3	5	2.12132	5	14	9	8	6.36396	2	3	1	2.67	0.70711	28.0855	180.948	152.862	129.994	108.09	1.1	1.45	0.35	1.33	0.24749
1.33	2.2	0.87	1.91	0.61518	5	6	1	5.66667	0.70711	4	9	5	7.33333	3.53553	3	3	0	3	0	91.224	192.217	100.993	158.553	71.4128	1.35	1.55	0.2	1.42	0.14142
1	1.69	0.69	1.46	0.4879	4	5	1	4.66667	0.70711	2	12	10	8.66667	7.07107	1	3	2	2.33	1.41421	40.078	112.411	72.333	88.3	51.1472	1.55	1.8	0.25	1.63	0.17678
1.36	2.1	0.74	1.85333	0.52326	5	9	4	6.33333	2.82843	7	16	9	13	6.36396	2	3	1	2.33	0.70711	127.6	237	109.4	164.067	77.3575	1.4	1.75	0.35	1.52	0.24749
1.54	2.05	0.51	1.88	0.36062	4	5	1	4.66667	0.70711	4	15	11	11.3333	7.77817	2	3	1	2.33	0.70711	47.867	121.76	73.893	97.129	52.2502	1.4	1.45	0.05	1.43	0.03536
1.54	2.2	0.66	1.76	0.46669	1	4	3	3	2.12132	1	4	3	3	2.12132	1	3	2	2.33	1.41421	1.00794	47.867	46.8591	32.2473	33.1344	0.25	1.4	1.15	1.02	0.81317
1.33	1.9	0.57	1.52	0.40305	3	5	2	4.33333	1.41421	4	14	10	7.33333	7.07107	2	3	1	2.67	0.70711	28.0855	91.224	63.1385	70.1778	44.6457	1.1	1.55	0.45	1.4	0.3182
1.83	2.04	0.21	1.9	0.14849	2	4	2	3.33333	1.41421	8	13	5	9.66667	3.53553	2	3	1	2.67	0.70711	10.811	55.845	45.034	40.8337	31.8438	0.85	1.4	0.55	1.22	0.38891
1.6	3.98	2.38	3.504	1.68291	2	5	3	2.6	2.12132	5	17	12	14.6	8.48528	2	3	1	2.2	0.70711	18.9984	92.9064	73.908	33.78	52.2608	0.5	1.45	0.95	0.69	0.67175
0.89	3.44	2.55	2.38333	1.46256	2	6	4	3.83333	2.38366	2	16	14	10.3333	8.01136	1	3	2	1.83	0.87905	15.9994	137.327	121.328	71.512	72.2245	0.6	2.15	1.55	1.25	0.89086
1.22	1.61	0.39	1.48	0.27577	3	5	2	3.66667	1.41421	3	13	10	9.66667	7.07107	2	3	1	2.33	0.70711	26.9815	88.9059	61.9243	47.623	43.7871	1.25	1.8	0.55	1.43	0.38891
0.89	1.65	0.76	1.39667	0.5374	4	6	2	4.66667	1.41421	2	12	10	8.66667	7.07107	1	3	2	2.33	1.41421	65.409	137.327	71.918	89.3817	50.8537	1.35	2.15	0.8	1.62	0.56569
0.82	2.55	1.73	1.45	0.95598	2	5	3	3.66667	1.52753	1	16	15	6	8.66025	1	2	1	1.33	0.57735	6.941	85.4678	78.5268	57.1229	43.5805	1.15	2.35	1.2	1.65	0.6245
1.25	1.83	0.58	1.63667	0.41012	4	8	4	5.33333	2.82843	8	15	7	10.3333	4.94975	3	3	0	3	0	55.845	168.934	113.089	93.5414	79.9661	1.4	1.75	0.35	1.52	0.24749
1.14	2.28	1.14	1.9	0.8061	5	8	3	6	2.12132	6	9	3	8	2.12132	3	3	0	3	0	102.906	144.242	41.3365	116.684	29.2293	1.35	1.85	0.5	1.52	0.35355
1.33	1.61	0.28	1.42333	0.19799	3	5	2	4.33333	1.41421	4	13	9	7	6.36396	2	3	1	2.67	0.70711	26.9815	91.224	64.2425	69.8098	45.4263	1.25	1.55	0.3	1.45	0.21213
1.22	1.9	0.68	1.55667	0.34005	3	5	2	4	1	3	14	11	8	5.56776	2	3	1	2.67	0.57735	28.0855	88.9059	60.8204	57.3098	30.4795	1.1	1.8	0.7	1.43	0.35119
0.89	2.54	1.65	1.87333	0.86939	3	6	3	5	1.73205	2	15	13	9.33333	6.65833	1	3	2	2	1	30.9738	196.967	165.993	121.756	84.0848	1	2.15	1.15	1.5	0.58949
1.1	2.54	1.44	1.87333	0.59029	4	8	4	5	2.16025	11	16	5	11.8333	2.63523	3	3	0	3	0	63.546	196.967	133.421	104.032	81.5586	1.35	1.75	0.4	1.42	0.21082
1.3	1.9	0.6	1.5	0.42426	3	6	3	5	2.12132	4	14	10	7.33333	7.07107	2	3	1	2.67	0.70711	28.0855	178.49	150.405	128.355	106.352	1.1	1.55	0.45	1.4	0.3182

1.1	2.28	1.18	1.88667	0.83439	5	8	3	6	2.12132	[illegible]
2.2	3.04	0.84	2.76	0.59397	2	6	4	3.33333	2.82843	[illegible]
0.98	2.01	1.03	1.6	0.54617	2	4	2	3.33333	1.1547	[illegible]
1.91	2.19	0.28	2.09667	0.19799	3	4	1	3.33333	0.70711	[illegible]
1.5	1.57	0.07	1.52333	0.0495	2	6	4	4.66667	2.82843	[illegible]
1.27	3.98	2.71	2.61	1.35525	2	8	6	4.33333	3.21455	[illegible]
1.1	1.91	0.81	1.775	0.57276	4	8	4	4.66667	2.82843	[illegible]
0.79	3.44	2.65	2.64833	1.17678	1	6	5	2.66667	1.95789	[illegible]
1.24	1.65	0.41	1.58167	0.28991	4	8	4	4.66667	2.82843	[illegible]
0.79	3.44	2.65	2.04333	1.3308	2	6	4	4	2	[illegible]
1.22	2.05	0.83	1.53333	0.45081	5	5	0	5	0	[illegible]
1.2	1.81	0.61	1.60667	0.43134	4	8	4	5.33333	2.82843	[illegible]
2.28	3.04	0.76	2.78667	0.5374	2	6	4	3.33333	2.82843	[illegible]
1.54	1.57	0.03	1.56	0.02121	2	4	2	2.66667	1.41421	[illegible]
0.93	2.58	1.65	1.49667	0.93853	2	3	1	2.66667	0.57735	[illegible]
1.17	2.2	1.03	1.85667	0.72832	5	8	3	6	2.12132	[illegible]
0.82	3.44	2.62	1.73	1.48192	2	5	3	3.33333	1.52753	[illegible]
1.22	2.28	1.06	1.88167	0.61083	4	5	1	4.83333	0.47673	[illegible]
1.22	1.83	0.61	1.62667	0.43134	4	5	1	4.33333	0.70711	[illegible]
2.54	3.04	0.5	2.87333	0.35355	2	6	4	3.33333	2.82843	[illegible]
0.79	2.58	1.79	2.26167	0.84222	3	6	3	5	1.80907	[illegible]
1.17	2.01	0.84	1.59667	0.42016	3	8	5	5	2.64575	[illegible]
1.12	1.91	0.79	1.74167	0.40926	4	8	4	4.83333	2.06828	[illegible]
1.22	2.28	1.06	1.92667	0.74953	5	6	1	5.66667	0.70711	[illegible]
1.2	2.28	1.08	1.92	0.76368	5	8	3	6	2.12132	[illegible]
1.2	2.28	1.08	2.02	0.48878	2	8	6	4.5	2.63715	[illegible]
1.2	1.31	0.11	1.27333	0.07778	3	8	5	4.66667	3.53553	[illegible]
1.6	1.66	0.06	1.64	0.04243	4	5	1	4.33333	0.70711	[illegible]
2.28	3.44	1.16	3.05333	0.82024	2	5	3	3	2.12132	[illegible]
1.33	1.83	0.5	1.66333	0.35355	4	5	1	4.33333	0.70711	[illegible]
1	2.28	1.28	1.98667	0.58091	2	5	3	3.83333	1.71888	[illegible]
1.31	2.19	0.88	1.75667	0.42341	3	4	1	3.5	0.6396	[illegible]
2.1	2.2	0.1	2.13333	0.07071	5	5	0	5	0	[illegible]
0.93	2.18	1.25	1.76333	0.88388	3	4	1	3.66667	0.70711	[illegible]
1.33	1.57	0.24	1.53	0.16971	2	5	3	2.5	2.12132	[illegible]
1.1	1.65	0.55	1.55833	0.38891	4	8	4	4.66667	2.82843	[illegible]
1.3	2.2	0.9	1.9	0.6364	5	9	4	6.33333	2.82843	[illegible]
1.12	1.61	0.49	1.44667	0.34648	3	8	5	4.66667	3.53553	[illegible]
1.22	2.33	1.11	1.82667	0.56217	5	8	3	6.33333	1.52753	[illegible]
0.98	2.19	1.21	1.71	0.57606	2	8	6	4	2.2532	[illegible]
1.78	3.44	1.66	2.72667	0.85424	2	5	3	3.66667	1.52753	[illegible]
1.25	1.93	0.68	1.79667	0.34609	4	8	4	4.83333	2.06828	[illegible]
0.89	3.98	3.09	2.51	1.55045	2	6	4	4.33333	2.08167	[illegible]
1.93	3.98	2.05	3.29667	1.44957	2	5	3	3	2.12132	[illegible]
2.28	3.04	0.76	2.78667	0.5374	2	5	3	3	2.12132	[illegible]
1.3	3.44	2.14	2.28	1.0813	2	9	7	5.33333	3.51188	[illegible]
1.1	2.54	1.44	1.99	0.77788	6	8	2	6.66667	1.1547	[illegible]
0.98	2.05	1.07	1.53333	0.53594	2	5	3	3	1.73205	[illegible]
0.93	1.93	1	1.59667	0.70711	3	5	2	4.33333	1.41421	[illegible]
1.25	1.91	0.66	1.69	0.46669	4	8	4	5.33333	2.82843	[illegible]
1.31	2.2	0.89	1.90333	0.62933	1	3	2	1.66667	1.41421	[illegible]
1.2	3.44	2.24	2.6	1.22049	2	8	6	4.33333	3.21455	[illegible]
1.3	3.04	1.74	2.43333	0.98231	2	6	4	4	2	[illegible]
1.91	2.28	0.37	2.12333	0.1914	4	5	1	4.33333	0.57735	[illegible]
1.36	2.18	0.82	1.88	0.45211	4	9	5	6	2.64575	[illegible]
1.3	2.18	0.88	1.86	0.48662	4	9	5	6	2.64575	[illegible]
1.22	2.55	1.33	2.10667	0.94045	4	8	4	5.33333	2.82843	[illegible]
1.22	2.1	0.88	1.80667	0.62225	5	8	3	6	2.12132	[illegible]
1.1	2.28	1.18	1.88667	0.83439	6	8	2	6.66667	1.41421	[illegible]
1.12	1.65	0.53	1.56167	0.37477	4	8	4	4.66667	2.82843	[illegible]
1.25	1.91	0.66	1.8	0.46669	4	8	4	4.66667	2.82843	[illegible]
1	1.81	0.81	1.54	0.57276	4	4	0	4	0	[illegible]
2.2	3.44	1.24	3.02667	0.87681	2	6	4	3.33333	2.82843	[illegible]
0.95	1.93	0.98	1.60333	0.69296	5	5	0	5	0	[illegible]
2.04	2.55	0.51	2.465	0.36062	2	2	0	2	0	[illegible]
1.54	2.2	0.66	1.98	0.46669	1	4	3	2	2.12132	[illegible]
1.17	1.96	0.79	1.67667	0.43981	4	8	4	5.66667	2.08167	[illegible]
2	3.44	1.44	2.72	1.01823	2	6	4	4	2.82843	[illegible]
0.98	2.19	1.21	1.73	0.54848	2	5	3	3.5	1.1266	[illegible]
1.38	2.04	0.66	1.825	0.2815	2	9	7	4.16667	2.99241	[illegible]
1.2	2.58	1.38	2.12	0.97581	3	8	5	4.66667	3.53553	[illegible]
1.57	2.28	0.71	1.68833	0.50205	2	6	4	2.66667	2.82843	[illegible]
1.78	3.44	1.66	2.79333	0.88867	2	5	3	3.33333	1.52753	[illegible]
1.24	2.54	1.3	1.89667	0.53076	4	8	4	5	2.16025	[illegible]
0.98	2.19	1.21	1.57333	0.60534	2	4	2	3	1	[illegible]
0.89	1.61	0.72	1.37	0.50912	3	6	3	4	2.12132	[illegible]
1.27	1.83	0.56	1.64333	0.39598	4	8	4	5.33333	2.82843	[illegible]
1.24	1.81	0.57	1.56667	0.294	4	8	4	5.33333	2.3094	[illegible]
1.13	1.83	0.7	1.59667	0.49497	4	8	4	5.33333	2.82843	[illegible]
1.2	2.01	0.81	1.62	0.40583	4	8	4	5.33333	2.3094	[illegible]
1.6	3.44	1.84	2.82667	1.30108	2	5	3	3	2.12132	[illegible]
1.13	2.55	1.42	2.05333	0.52675	2	8	6	4.33333	2.85549	[illegible]
1.31	3.98	2.67	3.09	1.88798	2	3	1	2.33333	0.70711	[illegible]
1.2	2.2	1	1.98	0.45551	2	8	6	4.5	2.63715	[illegible]
1.12	2.2	1.08	2.02	0.76368	6	8	2	6.33333	1.41421	[illegible]
1.22	1.91	0.69	1.68	0.4879	4	5	1	4.33333	0.70711	[illegible]
0.82	3.44	2.62	1.62667	1.62158	2	5	3	4	2	[illegible]
1.1	2.2	1.1	1.82167	0.46928	4	8	4	5	1.80907	[illegible]
2.2	3.44	1.24	3.02667	0.87681	2	5	3	3	2.12132	[illegible]
1.3	2.54	1.24	1.71333	0.87681	6	9	3	8	2.12132	[illegible]
1.1	1.78	0.68	1.55333	0.48083	5	8	3	6	2.12132	[illegible]
1.3	3.16	1.86	2.5	1.04096	2	6	4	3.66667	2.08167	[illegible]
1.2	1.65	0.45	1.575	0.3182	4	8	4	4.66667	2.82843	[illegible]
1.81	2.1	0.29	1.97333	0.14844	4	5	1	4.33333	0.57735	[illegible]
0.95	2.02	1.07	1.62333	0.5862	4	6	2	5	1	[illegible]
2.04	2.55	0.51	2.465	0.36062	2	2	0	2	0	[illegible]
0.93	2.05	1.12	1.43	0.56956	3	5	2	3.66667	1.1547	[illegible]
1.33	2.55	1.22	2.17833	0.74826	4	5	1	4.16667	0.52705	[illegible]
1.2	2.28	1.08	2.02	0.48878	2	8	6	4.5	2.63715	[illegible]
1	3.16	2.16	2.44	1.52735	3	4	1	3.33333	0.70711	[illegible]
2.04	2.55	0.51	2.465	0.36062	2	2	0	2	0	[illegible]
0.95	3.98	3.03	2.63	1.54172	2	5	3	3.66667	1.52753	[illegible]
1.31	2.2	0.89	1.94167	0.52513	1	4	3	1.83333	1.71594	[illegible]
1.1	1.91	0.81	1.64	0.57276	4	8	4	5.33333	2.82843	[illegible]
0.89	3.44	2.55	2.87167	1.32967	2	6	4	2.83333	2.06828	[illegible]
0.95	2.55	1.6	2.01667	1.13137	2	5	3	3	2.12132	[illegible]
1.5	2.19	0.69	1.96	0.4879	3	9	6	5	4.24264	[illegible]
1.2	1.65	0.45	1.49	0.25159	4	8	4	6	2	[illegible]
1.22	2.54	1.32	1.89333	0.53905	4	8	4	5	2.16025	[illegible]
1.55	2.04	0.49	1.71333	0.34648	2	4	2	3.33333	1.41421	[illegible]
2.2	3.44	1.24	2.61333	0.87681	2	5	3	4	2.12132	[illegible]
2.33	3.98	1.65	3.09	0.83265	2	6	4	4	2	[illegible]
1.3	3.04	1.74	2.33333	0.91484	2	9	7	5.33333	3.51188	[illegible]
1	1.9	0.9	1.73	0.46583	4	5	1	4.16667	0.52705	[illegible]
1.31	1.65	0.34	1.53667	0.24042	3	4	1	3.66667	0.70711	[illegible]
0.95	1.93	0.98	1.76667	0.69296	5	5	0	5	0	[illegible]
2.33	3.44	1.11	3.07	0.78489	2	6	4	3.33333	2.82843	[illegible]
1.9	3.04	1.14	2.28	0.8061	2	6	4	4.66667	2.82843	[illegible]
1.14	1.83	0.69	1.6	0.4879	4	8	4	5.33333	2.82843	[illegible]
1.31	1.9	0.59	1.50667	0.41719	3	3	0	3	0	[illegible]
0.82	2.18	1.36	1.51667	0.68061	4	5	1	4.33333	0.57735	[illegible]
1.12	1.31	0.19	1.24667	0.13435	3	8	5	4.66667	3.53553	[illegible]
1.96	2.2	0.24	2.04	0.16971	5	5	0	5	0	[illegible]
1.69	2.66	0.97	2.33667	0.68589	5	5	0	5	0	[illegible]
1.24	1.9	0.66	1.79	0.46669	4	8	4	4.66667	2.82843	[illegible]
1.24	2.2	0.96	1.84	0.40988	4	8	4	4.83333	2.06828	[illegible]
1.1	2.55	1.45	2.06667	1.0253	4	8	4	5.33333	2.82843	[illegible]
1.3	1.61	0.31	1.40333	0.2192	3	9	6	7	4.24264	[illegible]
1.1	2.55	1.45	2.02167	0.52467	2	8	6	4.33333	2.85549	[illegible]
1.27	1.96	0.69	1.71	0.38223	4	8	4	5.66667	2.08167	[illegible]
1.33	3.04	1.71	2.44333	0.965	2	5	3	3.66667	1.52753	[illegible]
1.9	3.44	1.54	2.92667	1.08894	2	3	1	2.33333	0.70711	[illegible]
1.2	2.28	1.08	1.92	0.76368	6	8	2	6.66667	1.41421	[illegible]
1.24	1.96	0.72	1.72	0.50912	5	8	3	6	2.12132	[illegible]
1.33	2.54	1.21	1.89333	0.60929	4	6	2	5	1	[illegible]
1.27	2.55	1.28	2.12333	0.9051	4	8	4	5.33333	2.82843	[illegible]
2	2.58	0.58	2.29	0.41012	3	6	3	4.5	2.12132	[illegible]
1.2	1.9	0.7	1.66667	0.49497	3	8	5	4.66667	3.53553	[illegible]
1	2.54	1.54	2.02667	1.08894	4	6	2	5.33333	1.41421	[illegible]
1.9	3.44	1.54	2.92667	1.08894	2	6	4	3.33333	2.82843	[illegible]
1	2.28	1.28	1.85333	0.9051	4	6	2	5.33333	1.41421	[illegible]
0.98	2.18	1.2	1.57	0.60025	2	4	2	3.33333	1.1547	[illegible]
1.54	1.9	0.36	1.78	0.25456	4	4	0	4	0	[illegible]
0.82	2.54	1.72	1.82	0.89353	4	6	2	5	1	[illegible]
0.82	3.44	2.62	1.91167	1.34084	2	5	3	3.16667	1.25576	[illegible]
2.2	3.04	0.84	2.76	0.59397	2	5	3	3	2.12132	[illegible]
1.14	3.44	2.3	2.67333	1.62635	2	8	6	4	4.24264	[illegible]
2.28	2.33	0.05	2.305	0.03536	5	6	1	5.5	0.70711	[illegible]
1.17	1.9	0.73	1.63333	0.40278	3	8	5	5	2.64575	[illegible]
1	2.96	1.96	2.00857	1.163	4	4	0	4	0	[illegible]
1	3.16	2.16	2.09571	1.27882	3	4	1	3.42857	0.63246	[illegible]
1.1	1.65	0.55	1.45667	0.30925	4	8	4	6	2	[illegible]
0.89	2	1.11	1.63	0.78489	6	6	0	6	0	[illegible]
1.1	3.98	2.88	2.74667	1.48382	2	8	6	4.33333	3.21455	[illegible]
0.93	2.1	1.17	1.805	0.51348	3	5	2	4.16667	0.87905	[illegible]
0.82	2.05	1.23	1.62333	0.69616	4	6	2	5	1	[illegible]
1.25	1.9	0.65	1.68333	0.37528	3	8	5	5	2.64575	[illegible]
1.25	2.28	1.03	2.02833	0.46595	2	8	6	4.5	2.63715	[illegible]
1.22	2.28	1.06	2.02333	0.47964	2	5	3	4	1.80907	[illegible]
1.12	2.28	1.16	1.89333	0.82024	6	8	2	6.66667	1.41421	[illegible]
0.89	1.96	1.07	1.38667	0.5391	3	6	3	4.66667	1.52753	[illegible]
0.93	2.18	1.25	1.55333	0.62501	3	4	1	3.66667	0.57735	[illegible]

1.2	3.98	2.78	2.78	1.42843	2	8	6	4.33333	3.21455	9	17	8	14.3333	4.6188	2	3	1	2.33	0.57735	18.9984	151.964	132.966	68.8051	[illegible]
0.93	2.19	1.26	1.55667	0.63003	3	4	1	3.33333	0.57735	1	15	14	7.66667	7.02377	1	3	2	2	1	22.9898	54.938	31.9483	36.3005	[illegible]
0.98	2.18	1.2	1.66333	0.61712	2	4	2	3.33333	1.1547	1	15	14	8	7	1	3	2	2	1	6.941	74.9216	67.9806	45.9025	[illegible]
2.2	3.04	0.84	2.76	0.59397	2	6	4	3.33333	2.82843	8	15	7	12.6667	4.94975	2	3	1	2.33	0.70711	14.0067	190.23	176.223	72.7478	[illegible]
2.55	3.04	0.49	2.83	0.34648	2	2	0	2	0	14	15	1	14.5714	0.70711	2	2	0	2	0	12.0107	14.0067	1.996	13.1513	[illegible]
0.79	2.66	1.87	1.78833	1.11798	2	6	4	4.16667	2.01133	1	17	16	9	10.2336	1	2	1	1.5	0.6396	6.941	132.905	125.964	87.9168	[illegible]
1.83	1.96	0.13	1.87333	0.09192	4	5	1	4.33333	0.70711	8	14	6	10	4.24264	2	3	1	2.67	0.70711	55.845	118.71	62.865	76.8	[illegible]
1.57	2.96	1.39	2.49667	0.98288	2	4	2	3.33333	1.41421	2	17	15	12	10.6066	1	2	1	1.67	0.70711	9.01218	79.904	70.8918	56.2734	[illegible]
2.02	3.44	1.42	2.87333	0.75215	2	6	4	3.66667	2.08167	15	17	2	16	1	2	2	0	2	0	15.9994	208.98	192.981	86.8109	[illegible]
1.14	1.91	0.77	1.65333	0.54447	4	8	4	5.33333	2.82843	6	10	4	8.66667	2.82843	3	3	0	3	0	58.6934	144.242	85.5486	87.2096	[illegible]
1.38	3.04	1.66	2.15333	0.83578	2	9	7	4.33333	4.04145	6	15	9	11.3333	4.72582	2	3	1	2.33	0.57735	10.811	238.029	227.218	87.6155	[illegible]
2	2.96	0.96	2.64	0.67882	4	6	2	4.66667	1.41421	12	17	5	15.3333	3.53553	2	3	1	2.33	0.70711	79.904	200.59	120.686	120.133	[illegible]
1	2.33	1.33	1.77667	0.69256	4	6	2	5.33333	1.1547	2	14	12	9.33333	6.4291	1	3	2	2	1	40.078	207.2	167.122	149.289	[illegible]
1.23	1.96	0.73	1.70667	0.41308	5	8	3	6	1.73205	11	14	3	12.6667	1.52753	2	3	1	2.67	0.57735	107.868	164.93	57.0621	130.503	[illegible]
0.93	3.44	2.51	2.485	1.2805	2	4	2	2.83333	1.14812	1	16	15	10.8333	7.25196	1	3	2	2.17	0.87905	15.9994	55.845	39.8456	30.4463	[illegible]
1.91	2.58	0.67	2.35667	0.47376	3	4	1	3.33333	0.70711	10	16	6	14	4.24264	2	3	1	2.33	0.70711	32.065	58.6934	26.6284	40.9411	[illegible]
1.38	1.93	0.55	1.81833	0.27766	4	9	5	5	2.58199	6	11	5	10.1667	2.63523	3	3	0	3	0	63.546	238.029	174.483	100.014	[illegible]
1.5	2.18	0.68	1.86	0.34176	3	6	3	4.33333	1.52753	5	15	10	11.3333	5.50757	2	3	1	2.33	0.57735	28.0855	180.948	152.862	94.6517	[illegible]
1.36	2.54	1.18	1.95333	0.59003	4	6	2	5	1	3	14	11	9.33333	5.68624	2	3	1	2.67	0.57735	44.9559	196.967	152.011	120.211	[illegible]
1.6	2.55	0.95	2.00714	0.67175	2	5	3	3.71429	2.12132	5	14	9	8.85714	6.36396	2	3	1	2.57	0.70711	12.0107	92.9064	80.8957	58.2368	[illegible]
2.55	3.04	0.49	2.83	0.34648	2	2	0	2	0	14	15	1	14.5714	0.70711	2	2	0	2	0	12.0107	14.0067	1.996	13.1513	[illegible]
1.63	2.18	0.55	1.99667	0.38891	4	4	0	4	0	5	15	10	11.6667	7.07107	2	3	1	2.33	0.70711	50.9415	74.9216	23.9801	66.9282	[illegible]
1.23	2.54	1.31	1.895	0.5349	4	8	4	5	2.16025	11	13	2	11.3333	1.05409	3	3	0	3	0	63.546	196.967	133.421	102.68	[illegible]
1.14	2.01	0.87	1.865	0.61518	4	8	4	4.66667	2.82843	6	14	8	12.6667	5.65685	2	3	1	2.17	0.70711	72.64	144.242	71.602	84.5737	[illegible]
1	2.02	1.02	1.64	0.55749	4	6	2	4.66667	1.1547	2	15	13	9.33333	6.65833	1	3	2	2	1	40.078	208.98	168.902	104.201	[illegible]
1.13	1.91	0.78	1.65	0.55154	4	8	4	5.33333	2.82843	5	10	5	8.33333	3.53553	3	3	0	3	0	58.6934	140.908	82.2142	86.0981	[illegible]
1.27	2.54	1.27	1.90833	0.51849	4	8	4	5	2.16025	10	17	7	11.3333	3.62093	3	3	0	3	0	58.6934	196.967	138.273	101.118	[illegible]
1.22	1.65	0.43	1.49667	0.24007	4	8	4	6	2	12	13	1	12.3333	0.57735	2	3	1	2.67	0.57735	65.409	204.383	138.974	144.097	[illegible]
1	3.16	2.16	2.09429	1.27866	3	4	1	3.57143	0.63246	2	17	15	10.2857	9.20869	1	2	1	1.57	0.63246	35.453	74.9216	39.4686	43.0735	[illegible]
1.38	2.2	0.82	1.92667	0.57983	6	9	3	7	2.12132	6	8	2	7.33333	1.41421	3	3	0	3	0	190.23	238.029	47.7989	206.163	[illegible]
1.12	2.2	1.08	2.02	0.76368	6	8	2	6.33333	1.41421	4	9	5	8.16667	3.53553	3	3	0	3	0	140.116	192.217	52.101	183.534	[illegible]
1.9	3.44	1.54	2.92667	1.08894	2	3	1	2.33333	0.70711	14	16	2	15.3333	1.41421	2	2	0	2	0	15.9994	28.0855	12.0861	20.0281	[illegible]
1.1	2.58	1.48	2.08667	1.04652	3	8	5	4.66667	3.53553	3	16	13	11.6667	9.19239	2	3	1	2.33	0.70711	32.065	138.905	106.84	67.6785	[illegible]
2.28	3.44	1.16	3.05333	0.82024	2	6	4	3.33333	2.82843	10	16	6	14	4.24264	2	3	1	2.33	0.70711	15.9994	195.084	179.085	75.6943	[illegible]
1.2	2.2	1	1.98	0.45551	2	8	6	5	2.86039	9	13	4	10.5	2.30612	2	3	1	2.67	0.60302	10.811	192.217	181.406	125.921	[illegible]
1.81	2.66	0.85	2.39167	0.53792	4	5	1	4.66667	0.66667	11	17	6	15.3333	3.43188	2	3	1	2.17	0.52705	63.546	126.904	63.3585	106.814	[illegible]
1.2	2.02	0.82	1.70667	0.44287	4	8	4	6	2	9	15	6	11.6667	3.05505	2	3	1	2.67	0.57735	63.546	208.98	145.434	141.497	[illegible]
2.33	3.98	1.65	3.15667	0.82501	2	6	4	3.66667	2.08167	14	17	3	16	1.73205	2	2	0	2	0	18.9984	207.2	188.202	87.2171	[illegible]
1.28	2.04	0.76	1.93143	0.5374	2	9	7	3	4.94975	8	13	5	12.2857	3.53553	2	3	1	2.14	0.70711	10.811	244	233.189	44.1237	[illegible]
0.98	1.9	0.92	1.43143	0.41889	2	3	1	2.85714	0.46291	1	14	13	5.28571	7.3046	1	2	1	1.29	0.59761	6.941	28.0855	21.1445	22.9046	[illegible]
1	2.04	1.04	1.89143	0.73539	2	4	2	2.28571	1.41421	2	13	11	11.4286	7.77817	1	2	1	1.86	0.70711	10.811	40.078	29.267	14.992	[illegible]
1.9	2.04	0.14	2.02	0.09899	2	3	1	2.14286	0.70711	13	14	1	13.1429	0.70711	2	2	0	2	0	10.811	28.0855	17.2745	13.2788	[illegible]
1.28	3.44	2.16	2.46	1.0938	2	9	7	5.33333	3.51188	8	17	9	13.6667	4.93288	2	3	1	2.33	0.57735	15.9994	244	228.001	128.968	[illegible]
1.17	3.98	2.81	2.86333	1.49112	2	8	6	4	3.4641	8	17	9	13.6667	4.93288	2	3	1	2.33	0.57735	15.9994	150.36	134.361	61.7859	[illegible]
0.82	2.58	1.76	1.40667	1.24451	3	4	1	3.66667	0.70711	1	16	15	6	10.6066	1	2	1	1.33	0.70711	32.065	39.0983	7.0333	36.7539	[illegible]
1.17	2.01	0.84	1.57333	0.42099	4	8	4	5.33333	2.3094	4	14	10	8.66667	5.03322	2	3	1	2.67	0.57735	47.867	150.36	102.493	90.289	[illegible]
1.12	1.9	0.78	1.77	0.55154	4	8	4	4.66667	2.82843	4	11	7	9.83333	4.94975	3	3	0	3	0	63.546	140.116	76.57	76.3077	[illegible]
0.89	2.33	1.44	1.51	0.74054	3	6	3	5	1.73205	2	14	12	6	6.9282	1	2	1	1.33	0.57735	24.305	207.2	182.895	122.944	[illegible]
1.12	1.65	0.53	1.46333	0.29771	4	8	4	6	2	4	13	9	9.66667	4.93288	2	3	1	2.67	0.57735	65.409	204.383	138.974	136.636	[illegible]
0.82	3.16	2.34	2.35429	1.35357	3	5	2	3.57143	0.96362	1	17	16	11.4286	9.26977	1	3	2	1.86	0.84515	35.453	106.42	70.967	46.6327	[illegible]
1.96	2.18	0.22	2.08571	0.15556	4	5	1	4.42857	0.70711	14	15	1	14.5714	0.70711	2	2	0	2	0	74.9216	118.71	43.7884	93.6881	[illegible]
1.5	3.04	1.54	2.445	0.88304	2	6	4	3.33333	2.41209	5	15	10	11.5	5.89684	2	3	1	2.33	0.60302	12.0107	180.948	168.937	69.3211	[illegible]
2.2	3.16	0.96	2.84	0.67882	3	5	2	3.66667	1.41421	10	17	7	14.6667	4.94975	2	3	1	2.33	0.70711	35.453	106.42	70.967	59.1087	[illegible]
0.89	3.44	2.55	1.97714	1.28754	2	6	4	4	2.22288	2	16	14	9.14286	7.54983	1	3	2	1.71	0.84017	14.0067	137.327	123.32	74.4861	[illegible]
1.14	2.04	0.9	1.91143	0.6364	2	8	6	2.85714	4.24264	6	13	7	12	4.94975	2	3	1	2.14	0.70711	10.811	144.242	133.431	29.8726	[illegible]
1.23	1.9	0.67	1.67667	0.47376	4	8	4	5.33333	2.82843	11	13	2	11.6667	1.41421	3	3	0	3	0	63.546	164.93	101.384	97.3408	[illegible]
1.12	2.01	0.89	1.68667	0.49238	4	8	4	5.66667	2.08167	4	14	10	9.66667	5.1316	2	3	1	2.67	0.57735	72.64	140.116	67.476	106.875	[illegible]
1.12	1.96	0.84	1.66	0.46861	4	8	4	5.66667	2.08167	4	14	10	9.66667	5.1316	2	3	1	2.67	0.57735	63.546	140.116	76.57	107.457	[illegible]
1.27	2.58	1.31	2.14333	0.92631	3	8	5	4.66667	3.53553	16	17	1	16.3333	0.70711	2	3	1	2.33	0.70711	32.065	174.967	142.902	79.699	[illegible]
1.2	1.93	0.73	1.78833	0.37242	4	8	4	4.83333	2.06828	10	11	1	10.8333	0.52705	3	3	0	3	0	63.546	157.25	93.704	86.5504	[illegible]
2.36	3.04	0.68	2.74857	0.48083	2	6	4	3.71429	2.82843	6	15	9	11.1429	6.36396	2	3	1	2.43	0.70711	14.0067	183.84	169.833	86.7924	[illegible]
1.23	2.01	0.78	1.73333	0.43662	4	8	4	5.66667	2.08167	13	14	1	13.6667	0.57735	2	3	1	2.33	0.57735	72.64	164.93	92.2903	118.76	[illegible]
1.36	2.04	0.68	1.94286	0.48083	2	9	7	3	4.94975	7	13	6	12.1429	4.24264	2	3	1	2.14	0.70711	10.811	237	226.189	43.1237	[illegible]
1.33	2.28	0.95	1.64667	0.67175	5	5	0	5	0	4	9	5	5.66667	3.53553	3	3	0	3	0	91.224	102.906	11.6815	95.1178	[illegible]
1.23	2.54	1.31	1.90167	0.53506	4	8	4	5	2.16025	10	13	3	10.6667	1.56347	3	3	0	3	0	58.6934	196.967	138.273	99.4451	[illegible]
1.62	2.1	0.48	1.935	0.20477	5	6	1	5.16667	0.47673	11	16	5	13	2.86039	2	3	1	2.5	0.6396	107.868	204.383	96.5151	130.531	[illegible]
1.23	2.33	1.1	1.82	0.55435	4	8	4	6	2	11	14	3	12.6667	1.52753	2	3	1	2.67	0.57735	63.546	207.2	143.654	145.225	[illegible]
1.13	3.44	2.31	2.51	1.21897	2	8	6	4.66667	3.05505	5	17	12	12.6667	6.65833	2	3	1	2.33	0.57735	15.9994	140.908	124.908	78.937	[illegible]
1.14	1.96	0.82	1.66667	0.45709	4	8	4	5.66667	2.08167	6	14	8	10.3333	4.04145	2	3	1	2.67	0.57735	63.546	144.242	80.696	108.833	[illegible]
2.16	2.58	0.42	2.44	0.29698	3	5	2	3.66667	1.41421	6	16	10	12.6667	7.07107	2	3	1	2.33	0.70711	32.065	95.94	63.875	53.3567	[illegible]
1.66	2.96	1.3	2.4	0.66843	3	4	1	3.66667	0.57735	6	17	11	13	6.08276	2	3	1	2.33	0.57735	32.065	79.904	47.839	54.655	[illegible]
1.27	2.1	0.83	1.82333	0.5869	5	8	3	6	2.12132	16	17	1	16.3333	0.70711	2	3	1	2.33	0.70711	127.6	174.967	47.367	143.389	[illegible]
1.22	1.9	0.68	1.67333	0.48083	3	5	2	3.66667	1.41421	3	14	11	10.3333	7.77817	2	3	1	2.33	0.70711	28.0855	88.9059	60.8204	48.359	[illegible]
1.17	1.96	0.79	1.67667	0.43981	4	8	4	5.66667	2.08167	8	14	6	11	3	2	3	1	2.67	0.57735	63.546	150.36	86.814	110.872	[illegible]
1.5	3.04	1.54	2.36	0.89982	2	6	4	3.33333	2.41209	5	15	10	11.3333	5.79969	2	3	1	2.33	0.60302	10.811	180.948	170.137	69.1211	[illegible]
1.1	2.54	1.44	1.88333	0.72831	4	8	4	6	2	11	16	5	13.6667	2.51661	2	3	1	2.67	0.57735	72.64	196.967	124.327	147.549	[illegible]
1.3	2.58	1.28	2.02	0.65483	3	9	6	5.33333	3.21455	4	16	12	11.6667	6.65833	2	3	1	2.33	0.57735	32.065	232.038	199.973	113.008	[illegible]
1.13	2.2	1.07	1.79333	0.57934	5	8	3	6	1.73205	5	15	10	10	5	2	3	1	2.67	0.57735	106.42	140.908	34.4876	123.029	[illegible]
1.55	1.91	0.36	1.78667	0.20502	3	4	1	3.66667	0.57735	7	14	7	10.3333	3.51188	2	3	1	2.67	0.57735	28.0855	58.6934	30.6079	47.239	[illegible]
1.24	2.33	1.09	1.82333	0.54903	4	8	4	6	2	11	14	3	13	1.73205	2	3	1	2.67	0.57735	63.546	207.2	143.654	146.002	[illegible]
1.62	2.28	0.66	1.95	0.46669	6	6	0	6	0	10	13	3	11.5	2.12132	2	3	1	2.5	0.70711	195.084	204.383	9.2993	199.734	[illegible]
0.79	3.44	2.65	2.95167	1.37478	2	6	4	2.83333	2.06828	1	17	16	13.6667	8.02773	1	2	1	1.83	0.52705	15.9994	132.905	116.906	38.726	[illegible]
0.82	2.2	1.38	1.67857	0.82123	1	4	3	2.14286	1.79284	1	2	1	1.14286	0.46291	1	1	0	1	0	1.00794	39.0983	38.0904	15.2191	[illegible]
1.54	2.2	0.66	2.01143	0.39443	1	5	4	2.42857	2.22004	1	10	9	3.14286	4.08831	1	3	2	1.86	1.30931	1.00794	106.42	105.412	29.4551	[illegible]
1.3	2.04	0.74	1.93429	0.52326	2	9	7	3	4.94975	4	13	9	11.7143	6.36396	2	3	1	2.14	0.70711	10.811	232.038	221.227	42.4149	[illegible]
1.2	2.04	0.84	1.92	0.59397	2	8	6	2.85714	4.24264	10	13	3	12.5714	2.12132	2	3	1	2.14	0.70711	10.811	157.25	146.439	31.7309	[illegible]
0.98	2.96	1.98	2.23286	1.16367	2	4	2	3.42857	1.19523	1	17	16	11.1429	9.39225	1	3	2	1.86	0.84515	6.941	79.904	72.963	55.6204	[illegible]
1.28	1.65	0.37	1.52667	0.26163	4	9	5	5.66667	3.53553	8	12	4	10.6667	2.82843	3	3	0	3	0	65.409	244	178.591	124.939	[illegible]
1.24	2.54	1.3	1.90333	0.53089	4	8	4	5	2.16025	10	14	4	10.8333	2.06828	3	3	0	3	0	58.6934	196.967	138.273	99.8332	[illegible]
1.38	2.02	0.64	1.80667	0.45255	6	9	3	7	2.12132	6	15	9	12	6.36396	2	3	1	2.33	0.70711	208.98	238.029	29.0485	218.663	[illegible]
0.79	2.05	1.26	1.46333	0.63446	4	6	2	5	1	1	15	14	7.66667	7.02377	1	3	2	2	1	54.938	132.905	77.9674	103.201	[illegible]
1.96	3.44	1.48	2.94667	1.04652	2	5	3	3	2.12132	14	16	2	15.3333	1.41421	2	2	0	2	0	15.9994	118.71	102.711	50.2363	[illegible]
1.62	3.44	1.82	2.22667	1.28693	2	6	4	4.66667	2.82843	13	16	3	14	2.12132	2	2	0	2	0	15.9994	204.383	188.384	141.589	[illegible]
0.82	2.18	1.36	1.31571	0.79712	2	4	2	3.71429	0.92582	1	15	14	5.14286	8.25919	1	2	1	1.29	0.59761	9.01218	74.9216	65.9094	45.0355	[illegible]
1.25	2.04	0.79	1.92714	0.55861	2	8	6	2.85714	4.24264	13	15	2	13.2857	1.41421	2	3	1	2.14	0.70711	10.811	168.934	158.123	33.4	[illegible]
1.22	2.04	0.82	1.92286	0.57983	2	8	6	2.85714	4.24264	12	13	1	12.8571	0.70711	2	3	1	2.14	0.70711	10.811	162.5	151.689	32.4809	[illegible]
1.23	2.04	0.81	1.92429	0.57276	2	8	6	2.85714	4.24264	13	13	0	13	0	2	3	1	2.14	0.70711	10.811	164.93	154.119	32.828	[illegible]
1.24	2.04	0.8	1.92571	0.56569	2	8	6	2.85714	4.24264	13	14	1	13.1429	0.70711	2	3	1	2.14	0.70711	10.811	167.259	156.448	33.1607	[illegible]
0.82	2.96	2.14	2.24	1.23572	4	5	1	4.14286	0.46291	1	17	16	11.4286	9.26977	1	3	2	1.86	0.84515	39.0983	106.42	67.3217	72.0332	[illegible]
1.1	2.04	0.94	1.90571	0.66468	2	8	6	2.85714	4.24264	11	13	2	12.7143	1.41421	2	3	1	2.14	0.70711	10.811	158.925	148.114	31.9702	[illegible]
0.95	2.19	1.24	1.32143	0.72682	2	5	3	2.71429	1.36277	1	15	14	5.14286	8.25919	1	2	1	1.29	0.59761	6.941	87.62	80.679	25.3331	[illegible]
1	2.18	1.18	1.804	0.53925	4	4	0	4	0	2	15	13	9.6	6.15427	1	3	2	2.2	0.93541	40.078	74.9216	34.8436	60.3222	[illegible]
0.79	2.19	1.4	1.51	0.70086	3	6	3	4.33333	1.52753	1	15	14	7.66667	7.02377	1	3	2	2	1	30.9738	132.905	101.932	72.9391	[illegible]
1.65	3.44	1.79	2.99833	0.96147	2	4	2	2.5	1.08012	12	16	4	15.3333	2.10819	2	3	1	2.17	0.52705	15.9994	65.409	49.4096	26.9119	[illegible]
0.89	2.18	1.29	1.83571	0.53572	4	6	2	4.28571	0.92582	2	15	13	10.8571	5.31171	1	3	2	2.43	0.96362	63.546	137.327	73.781	77.3363	[illegible]
0.98	2.96	1.98	2.19286	1.19471	2	4	2	3.42857	1.19523	1	17	16	11	9.48683	1	3	2	1.86	0.84515	6.941	79.904	72.963	55.4909	[illegible]
0.79	2.2	1.41	1.67	0.83696	1	6	5	2.71429	2.89087	1	2	1	1.14286	0.46291	1	1	0	1	0	1.00794	132.905	131.898	42.0211	[illegible]
0.82	2.19	1.37	1.38	0.85892	3	6	3	4	1.22474	1	15	14	6.57143	8.59817	1	3	2	1.57	0.96362	30.9738	200.59	169.616	59.8472	[illegible]
1.31	3.98	2.67	2.70143	1.58944	2	3	1	2.42857	0.63246	2	17	15	10.2857	9.20869	1	2	1	1.57	0.63246	14.0067	24.305	10.2983	20.5596	[illegible]
1.12	2.2	1.08	1.745	0.47628	4	8	4	5	1.80907	4	12	8	10	3.61814	3	3	0	3	0	65.409	140.116	74.707	91.5305	[illegible]
1.1	2.58	1.48	2.08667	1.04652	3	8	5	4.66667	3.53553	16	16	0	16	0	2	3	1	2.33	0.70711	32.065	173.04	140.975	79.0567	[illegible]
1.9	2.55	0.65	2.33333	0.45962	3	4	1	3.66667	0.70711	14	16	2	15.3333	1.41421	2	2	0	2	0	28.0855	78.96	50.8745	62.0018	[illegible]
1.38	2.2	0.82	1.92667	0.57983	6	9	3	7	2.12132	6	9	3	8	2.12132	3	3	0	3	0	192.217	238.029	45.8119	207.488	[illegible]
1.23	2.33	1.1	1.83	0.55678	5	8	3	6.33333	1.52753	11	14	3	12.6667	1.52753	2	3	1	2.67	0.57735	107.868	207.2	99.3318	160	[illegible]
1.2	2.54	1.34	1.92	0.67557	6	8	2	6.66667	1.1547	9	15	6	11.6667	3.05505	2	3	1	2.67	0.57735	151.964	208.98	57.0164	185.97	[illegible]
1.22	1.83	0.61	1.72833	0.43134	4	5	1	4.16667	0.70711	3	8	5	7.16667	3.53553	3	3	0	3	0	55.845	88.9059	33.0609	61.3551	[illegible]
1.38	3.04	1.66	2.17333	0.83243	2	9	7	5.33333	3.51188	6	16	10	12.3333	5.50757	2	3	1	2.33	0.57735	14.0067	238.029	224.022	126.545	[illegible]
1.38	2.05	0.67	1.82667	0.47376	5	9	4	6.33333	2.82843	6	15	9	12	6.36396	2	3	1	2.33	0.70711	121.76	238.029	116.269	160.516	[illegible]
1.22	2.28	1.06	2.02	0.56804	5	8	3	6.16667	1.2693	10	13	3	10.8333	1.71594	2	3	1	2.83	0.52705	114.818	195.084	80.266	176.276	[illegible]
0.89	1.93	1.04	1.58333	0.73539	5	6	1	5.33333	0.70711	2	11	9	8	6.36396	1	3	2	2.33	1.41421	107.868	137.327	29.4588	117.688	[illegible]
1.22	2.54	1.32	1.88667	0.6601	3	6	3	4.66667	1.52753	3	14	11	9.33333	5.68624	2	3	1	2.67	0.57735	28.0855	196.967	168.881	104.653	[illegible]
0.82	2.05	1.23	1.47333	0.61857	4	5	1	4.33333	0.57735	1	15	14	7.66667	7.02377	1	3	2	2	1	39.0983	121.76	82.6617	71.9321	[illegible]
1.1	2.28	1.18	2.08333	0.83439	6	8	2	6.33333	1.41421	3	10	7	8.83333	4.94975	3	3	0	3	0	138.905	195.084	56.1785	185.721	[illegible]
1.1	1.83	0.73	1.58667	0.51619	4	8	4	5.33333	2.82843	8	16	8	10.6667	5.65685	3	3	0	3	0	55.845	173.04	117.195	94.91	[illegible]
1.1	1.9	0.8	1.76667	0.56569	4	8	4	4.66667	2.82843	3	11	8	9.66667	5.65685	3	3	0	3	0	63.546	138.905	75.3595	76.1059	[illegible]
1.13	1.61	0.48	1.45	0.33941	3	8	5	4.66667	3.53553	5	13	8	10.3333	5.65685	2	3	1	2.33	0.70711	26.9815	140.908	113.926	64.9569	[illegible]
2.33	2.55	0.22	2.47667	0.15556	4	6	2	4.66667	1.41421	14	16	2	15.3333	1.41421	2	2	0	2	0	78.96	207.2	128.24	121.707	[illegible]
0.89	2.54	1.65	1.87	0.86758	4	6	2	5.33333	1.1547	2	15	13	9.33333	6.65833	1	3	2	2	1	74.9216	196.967	122.045	136.405	[illegible]
0.89	2.05	1.16	1.66333	0.82024	5	6	1	5.33333	0.70711	2	15	13	10.6667	9.19239	1	2	1	1.67	0.70711	121.76	137.327	15.567	126.949	[illegible]
0.95	3.44	2.49	2.665	1.55584	2	9	7	3.66667	3.71184	2	16	14	12.3333	7.73161	1	3	2	2	0.8165	15.9994	244	228.001	65.9363	[illegible]
1.2	3.44	2.24	2.69333	1.58392	2	8	6	4	4.24264	9	16	7	13.6667	4.94975	2	3	1	2.33	0.70711	15.9994	151.964	135.965	61.3209	[illegible]
2.02	3.44	1.42	2.70667	0.71115	2	6	4	4.33333	2.08167	15	17	2	16	1	2	2	0	2	0	15.9994	208.98	192.981	117.295	[illegible]
1.36	2.2	0.84	1.92	0.59397	6	9	3	7	2.12132	7	9	2	8.33333	1.41421	3	3	0	3	0	192.217	237	44.783	207.145	[illegible]
1.22	1.9	0.68	1.67333	0.48083	4	5	1	4.33333	0.70711	3	11	8	8.33333	5.65685	3	3	0	3	0	63.546	88.9059	25.3599	71.9993	[illegible]
1.66	3.44	1.78	2.68667	0.92094	2	4	2	3.33333	1.1547	6	17	11	13	6.08276	2	3	1	2.33	0.57735	15.9994	79.904	63.9046	49.2998	[illegible]
1.33	2.1	0.77	1.87833	0.45155	4	5	1	4.83333	0.52705	4	16	12	12	8	2	3	1	2.33	0.66667	47.867	127.6	79.733	108.248	[illegible]
1.1	1.9	0.8	1.74667	0.41366	4	8	4	4.83333	2.06828	11	13	2	11.3333	1.05409	2	3	1	2.83	0.52705	63.546	158.925	95.3794	87.9879	[illegible]
1.93	3.44	1.51	3.14167	0.77985	2	5	3	2.66667	1.56347	11	17	6	15.3333	2.78887	2	3	1	2.17	0.52705	15.9994	107.868	91.8688	34.5531	[illegible]
0.82	2	1.18	1.60667	0.83439	4	6	2	5.33333	1.41421	1	12	11	8.33333	7.77817	1	3	2	2.33	1.41421	39.0983	200.59	161.492	146.759	[illegible]
1.27	1.96	0.69	1.72	0.39	5	8	3	6	1.73205	11	17	6	14	3	2	3	1	2.67	0.57735	107.868	174.967	67.0988	133.848	[illegible]
1.62	2.1	0.48	1.935	0.20477	5	6	1	5.16667	0.47673	11	16	5	13	2.86039	2	3	1	2.5	0.6396	107.868	204.383	96.5151	130.531	[illegible]
1.22	2.54	1.32	1.92333	0.66425	4	6	2	5	1	3	14	11	9.33333	5.68624	2	3	1	2.67	0.57735	72.64	196.967	124.327	119.504	[illegible]
1.22	2.66	1.44	1.89667	0.7239	4	5	1	4.66667	0.57735	3	17	14	11	7.2111	2	3	1	2.33	0.57735	69.723	126.904	57.1815	95.1778	[illegible]
1.9	2.55	0.65	2.24333	0.39508	4	4	0	4	0	11	16	5	14	2.86039	2	3	1	2.33	0.60302	63.546	78.96	15.414	72.7687	[illegible]
0.95	3.98	3.03	2.97	2.14253	2	5	3	3	2.12132	2	17	15	12	10.6066	1	2	1	1.67	0.70711	18.9984	87.62	68.6216	41.8723	[illegible]
1.9	2.55	0.65	2.11667	0.45962	2	6	4	4.66667	2.82843	7	14	7	9.33333	4.94975	2	3	1	2.67	0.70711	12.0107	186.207	174.196	128.142	[illegible]
1.28	1.9	0.62	1.636	0.29629	3	9	6	4.6	2.80624	7	14	7	10	4.10792	2	3	1	2.6	0.61237	28.0855	244	215.915	82.0094	[illegible]
1.12	2.33	1.21	1.79333	0.61647	5	8	3	6.33333	1.52753	4	14	10	9.66667	5.1316	2	3	1	2.67	0.57735	107.868	207.2	99.3318	151.728	[illegible]
1.24	1.9	0.66	1.755	0.34329	4	8	4	4.83333	2.06828	11	14	3	11.6667	1.56347	3	3	0	3	0	63.546	167.259	103.713	88.9757	[illegible]
1.81	2.2	0.39	1.94	0.27577	4	6	2	4.66667	1.41421	8	13	5	11.3333	3.53553	2	3	1	2.33	0.70711	69.723	190.23	120.507	109.892	[illegible]
1.14	1.65	0.51	1.48	0.36062	4	8	4	5.33333	2.82843	6	12	6	10	4.24264	3	3	0	3	0	65.409	144.242	78.833	91.6867	[illegible]
1.23	1.96	0.73	1.69667	0.40526	4	8	4	5.66667	2.08167	11	14	3	12.6667	1.52753	2	3	1	2.67	0.57735	63.546	164.93	101.384	115.729	[illegible]
1.25	2.28	1.03	1.93667	0.72832	5	8	3	6	2.12132	9	15	6	11	4.24264	3	3	0	3	0	102.906	168.934	66.0287	124.915	[illegible]
1.22	1.65	0.43	1.50667	0.30406	4	8	4	5.33333	2.82843	12	12	0	12	0	3	3	0	3	0	65.409	162.5	97.091	97.7727	[illegible]
1.9	2.58	0.68	2.13667	0.38423	3	5	2	4	1	11	16	5	12.6667	2.88675	2	3	1	2.67	0.57735	32.065	107.868	75.8032	67.8264	[illegible]
1.55	2.05	0.5	1.71667	0.35355	4	5	1	4.33333	0.70711	7	15	8	9.66667	5.65685	2	3	1	2.67	0.70711	54.938	121.76	66.822	77.212	[illegible]
1.1	2.54	1.44	1.88	0.59093	4	8	4	5	2.16025	10	11	1	10.3333	0.66667	3	3	0	3	0	58.6934	196.967	138.273	98.4443	[illegible]
1.66	2.04	0.38	1.87714	0.2687	2	4	2	2.85714	1.41421	6	13	7	10	4.94975	2	3	1	2.43	0.70711	10.811	51.9961	41.1851	28.4618	[illegible]
1.96	2.58	0.62	2.27429	0.29004	3	5	2	4.42857	1.11803	9	16	7	12.4286	3.79144	2	3	1	2.43	0.61237	32.065	118.71	86.645	87.1809	[illegible]
1.61	2.58	0.97	2.17	0.6265	3	4	1	3.14286	0.46291	12	16	4	14.5714	2.22004	2	3	1	2.14	0.46291	26.9815	65.409	38.4275	35.376	[illegible]
0.82	2.18	1.36	1.32714	0.80432	4	4	0	4	0	1	15	14	6.57143	8.59817	1	3	2	1.57	0.96362	39.0983	74.9216	35.8233	53.0922	[illegible]
0.95	2.18	1.23	1.84429	0.50931	4	5	1	4.14286	0.46291	2	15	13	10.8571	5.31171	1	3	2	2.43	0.96362	63.546	87.62	24.074	70.2353	[illegible]

0.98	2.02	1.04	1.36667	0.60085	2	6	4	3.83333	2.38366	1	15	14	6	8.1909	1	3
1.91	2.58	0.67	2.22143	0.35363	3	6	3	4.28571	1.43614	10	16	6	12.8571	3.20156	2	3
1.1	2.16	1.06	1.69429	0.45149	3	8	5	4.28571	2.31455	6	16	10	11.4286	4.73588	2	3
0.82	3.44	2.62	1.80571	1.5184	2	4	2	3.71429	0.92582	1	17	16	7.71429	10.2644	1	2
1.61	2.58	0.97	2.22	0.57249	3	6	3	3.42857	1.38873	12	16	4	14.5714	2.22004	2	3
1.62	2.55	0.93	2.09857	0.55183	4	6	2	4.85714	1.26491	13	16	3	14.5714	1.78885	2	2
1.31	2.55	1.24	2.10429	0.69177	3	4	1	3.57143	0.65465	2	16	14	13.1429	6.26213	1	2
1.33	1.61	0.28	1.45	0.19799	3	5	2	4.14286	1.41421	4	13	9	7.85714	6.36396	2	3
1.1	1.9	0.8	1.54714	0.38018	3	8	5	4.42857	2.79508	11	14	3	12.7143	1.43614	2	3
0.93	2.19	1.26	1.44286	0.78723	3	6	3	3.42857	1.38873	1	15	14	6.57143	8.59817	1	3
0.89	1.96	1.07	1.40143	0.572	3	6	3	4.85714	1.5411	2	14	12	8.57143	7.0578	1	2
1.23	2.28	1.05	1.77286	0.41914	5	8	3	5.42857	1.5667	9	13	4	12.4286	2.08893	2	3
1.1	2.96	1.86	1.99857	1.10478	4	8	4	5.71429	2.52982	3	17	14	10.4286	8.39047	2	3
1.91	2.55	0.64	2.21286	0.34048	4	6	2	4.57143	1.11803	10	16	6	12.8571	3.20156	2	3
1.62	2.58	0.96	2.01857	0.46383	3	6	3	4.28571	1.43614	10	16	6	12.5714	3.09233	2	3
0.95	3.44	2.49	1.66	1.1986	2	5	3	4	1.58114	2	16	14	7.71429	8.73826	1	2
2.02	2.58	0.56	2.25714	0.26898	3	6	3	4.71429	1.43614	10	16	6	13.1429	3.40037	2	3
1.22	2.55	1.33	1.95143	0.62925	2	5	3	2.85714	1.67705	3	14	11	10.4286	5.83631	2	3
1.1	2.28	1.18	1.75429	0.47455	5	8	3	5.42857	1.5667	9	16	7	12.8571	2.81231	2	3
1.3	1.81	0.51	1.47	0.36062	4	9	5	7.33333	3.53553	4	13	9	7	6.36396	2	3

[illegible]

0.98	3.44	2.46	2.24857	1.31644	1	8	7	3.57143	3.38683	1	16	15	8.28571	8.13489	1	3	[illegible]
1.22	1.9	0.68	1.67333	0.48083	3	8	5	4.66667	3.53553	12	14	2	13.3333	1.41421	2	3	[illegible]
1.55	2.36	0.81	1.94571	0.36068	2	6	4	3.14286	1.92725	6	13	7	10.2857	4.16619	2	3	[illegible]
0.89	3.98	3.09	3.33375	1.80029	2	6	4	2.625	2.06621	2	17	15	14.75	7.71612	1	2	[illegible]
1.91	2.01	0.1	1.94333	0.07071	4	4	0	4	0	10	14	4	11.3333	2.82843	2	3	[illegible]
0.95	2.18	1.23	1.8725	0.86974	4	5	1	4.25	0.70711	2	15	13	11.75	9.19239	1	2	[illegible]
1.1	3.44	2.34	2.7575	1.05653	2	8	6	3.875	2.79029	15	17	2	15.875	0.74001	2	3	[illegible]
1.66	2.19	0.53	1.94375	0.32509	3	4	1	3.5	0.64889	6	15	9	11.375	5.4652	2	3	[illegible]
1.78	2.1	0.32	1.94	0.22627	5	5	0	5	0	13	16	3	14.5	2.12132	2	2	[illegible]
1.66	2.28	0.62	1.815	0.43841	4	6	2	4.5	1.41421	6	10	4	7	2.82843	3	3	[illegible]
1.78	2.55	0.77	2.25143	0.46483	4	6	2	4.57143	0.96362	12	16	4	14.5714	2.22004	2	3	[illegible]
1.12	1.83	0.71	1.59333	0.50205	4	8	4	5.33333	2.82843	4	8	4	6.66667	2.82843	3	3	[illegible]
0.95	3.44	2.49	2.49857	1.48225	2	5	3	3.14286	1.79284	2	16	14	10.8571	8.22887	1	3	[illegible]
2.01	2.18	0.17	2.11	0.07106	4	5	1	4.57143	0.65465	14	16	2	15.4286	0.96362	2	2	[illegible]
0.82	3.98	3.16	2.79429	1.87418	2	6	4	3.42857	2.22004	1	17	16	11.7143	9.23889	1	3	[illegible]
0.82	2.58	1.76	2.02	0.71648	3	5	2	3.75	0.8584	1	16	15	11.625	6.01533	1	3	[illegible]
1.23	2.33	1.1	1.82	0.55435	4	8	4	6	2	11	14	3	12.6667	1.52753	2	3	[illegible]
2.36	2.96	0.6	2.87429	0.42426	4	6	2	4.28571	1.41421	6	17	11	15.4286	7.77817	2	3	[illegible]
[illegible]																		

1.1	1.93	0.83	1.515	0.5869	5	8	3	6.5	2.12132	11	16	5	13.5	3.53553	3	3	0	3	0	107.868	173.04	65.1718	140.454	46.0834	1.6	1.75	0.15	1.68	0.10607	17	71	54	44	38.1838	1.63E-08	2.50E-07	2.34E-07	1.33E-07	1.65E-07	1590	2600
1.1	3.44	2.34	2.7325	1.04511	2	8	6	4	2.76026	11	17	6	15.25	2.1157	2	3	1	2.13	0.40825	15.9994	208.98	192.981	90.0985	102.418	0.6	1.75	1.15	1.06	0.60376	26	101	75	87.75	29.6696	0	0	0	0	0	0	0
1.78	2.55	0.77	2.165	0.54447	4	5	1	4.5	0.70711	13	16	3	14.5	2.12132	2	2	0	2	0	78.96	114.818	35.858	96.889	25.3554	1.15	1.55	0.4	1.35	0.28284	79	93	14	86	9.89949	1.00E-08	8.00E-08	7.00E-08	4.50E-08	4.95E-08	1215	3350
2.2	3.98	1.78	3.535	1.25865	2	6	4	3	2.82843	9	17	8	15	5.65685	2	3	1	2.25	0.70711	18.9984	192.217	173.219	62.3031	122.484	0.5	1.35	0.85	0.71	0.60104	66	102	36	93	25.4558	0	0	0	0	0	0	0
1.1	3.44	2.34	2.695	1.03984	2	8	6	4.125	2.79029	11	17	6	15.25	2.1157	2	3	1	2.13	0.40825	15.9994	208.98	192.981	95.9735	103.32	0.6	1.75	1.15	1.09	0.61909	26	101	75	87.625	29.6065	0	0	0	0	0	0	0
1.3	2.19	0.89	1.745	0.62933	3	6	3	4.5	2.12132	4	15	11	9.5	7.77817	2	3	1	2.5	0.70711	30.9738	178.49	147.516	104.732	104.31	1	1.55	0.55	1.28	0.38891	50	90	40	70	28.2843	1.00E-07	3.40E-07	2.40E-07	2.20E-07	1.70E-07	0	0
1.81	2.58	0.77	2.25286	0.49979	3	4	1	3.42857	0.65465	8	16	8	14	3.67423	2	3	1	2.14	0.46291	32.065	69.723	37.658	46.2216	22.3544	1	1.4	0.4	1.14	0.222	61	94	33	85.5714	15.2456	1.00E-07	1.00E+23	1.00E+23	5.71E+22	6.55E+22	0	0
1.22	2.96	1.74	2.25333	0.91484	3	8	5	5	2.64575	12	17	5	15	2.64575	2	3	1	2.33	0.57735	32.065	162.5	130.435	91.4897	65.9848	1	1.75	0.75	1.3	0.39686	24	98	74	72	41.6173	9.26E-07	1.00E+23	1.00E+23	3.33E+22	5.77E+22	0	0
0.93	3.44	2.51	1.78286	1.34111	2	5	3	3.42857	1.38873	1	17	16	7.71429	10.2644	1	2	1	1.43	0.65465	15.9994	126.904	110.905	51.6811	63.014	0.6	1.8	1.2	1.51	0.54511	11	101	90	48.4286	57.1964	0	0	0	0	0	0	0
1.66	1.81	0.15	1.6975	0.10607	4	4	0	4	0	6	13	7	7.75	4.94975	2	3	1	2.75	0.70711	51.9961	69.723	17.7269	56.4278	12.5348	1.3	1.4	0.1	1.37	0.07071	57	81	24	63	16.9706	1.27E-07	1.40E-07	1.30E-08	1.30E-07	9.19E-09	2740	5940
1.2	1.69	0.49	1.52667	0.34648	5	8	3	6	2.12132	9	12	3	11	2.12132	3	3	0	3	0	112.411	151.964	39.553	125.595	27.9682	1.55	1.85	0.3	1.65	0.21213	18	75	57	56	40.3051	7.00E-08	9.00E-07	8.30E-07	3.47E-07	5.87E-07	0	0
1.54	2.55	1.01	2.11714	0.71418	4	4	0	4	0	4	16	12	10.8571	8.48528	2	3	1	2.43	0.70711	47.867	78.96	31.093	65.6344	21.9861	1.15	1.4	0.25	1.26	0.17678	51	93	42	75	29.6985	1.00E-08	4.00E-07	3.90E-07	1.77E-07	2.76E-07	3350	4140
1.9	2.19	0.29	2.06571	0.20506	3	6	3	4.28571	2.12132	7	15	8	11.5714	5.65685	2	3	1	2.43	0.70711	30.9738	186.207	155.233	97.5023	109.766	1	1.35	0.35	1.15	0.24749	58	90	32	76.2857	22.6274	1.00E-07	1.80E-07	8.00E-08	1.34E-07	5.66E-08	0	0
1.31	2.01	0.7	1.54333	0.49497	3	4	1	3.33333	0.70711	2	14	12	6	8.48528	1	2	1	1.33	0.70711	24.305	72.64	48.335	40.4167	34.178	1.25	1.5	0.25	1.42	0.17678	73	84	11	76.6667	7.77817	4.40E-08	0.0005	0.0005	0.00017	0.00035	4602	5400
1.69	2.55	0.86	2.20625	0.43367	4	5	1	4.25	0.53452	11	16	5	14	2.69037	2	3	1	2.38	0.59761	63.546	118.71	55.164	84.2566	23.7036	1.15	1.55	0.4	1.29	0.18452	72	93	21	84.25	11.4933	1.00E-08	1.15E-07	1.05E-07	3.24E-08	4.52E-08	2310	3570
1.83	2.18	0.35	2.005	0.24749	4	4	0	4	0	8	15	7	11.5	4.94975	2	3	1	2.5	0.70711	55.845	74.9216	19.0766	65.3833	13.4892	1.15	1.4	0.25	1.28	0.17678	61	89	28	75	19.799	1.00E-07	3.30E-07	2.30E-07	2.15E-07	1.63E-07	0	0
1.78	2.55	0.77	2.2025	0.44465	4	5	1	4.5	0.63246	11	16	5	14	2.68328	2	3	1	2.25	0.54772	78.96	114.818	35.858	95.1515	20.7154	1.15	1.6	0.45	1.36	0.26972	71	93	22	84	11.9331	1.00E-08	8.00E-08	7.00E-08	2.91E-08	3.73E-08	1215	3350
1.33	2.05	0.72	1.69	0.50912	5	5	0	5	0	4	15	11	9.5	7.77817	2	3	1	2.5	0.70711	91.224	121.76	30.536	106.492	21.5922	1.45	1.55	0.1	1.5	0.07071	49	88	39	68.5	27.5772	4.00E-07	4.33E-07	3.30E-08	4.17E-07	2.33E-08	3420	3800
1.54	2.58	1.04	2.0525	0.64841	2	4	2	3.25	1.04881	4	16	12	9.5	7.01427	2	3	1	2.5	0.63246	12.0107	47.867	35.8563	34.9524	18.6358	0.7	1.4	0.7	1.13	0.37283	51	95	44	72.75	27.5155	4.00E-07	1.00E+23	1.00E+23	2.50E+22	5.48E+22	0	0
1.13	2.05	0.92	1.43667	0.65054	5	8	3	7	2.12132	5	15	10	8.33333	7.07107	2	3	1	2.67	0.70711	121.76	140.908	19.1476	134.525	13.5394	1.45	1.85	0.4	1.72	0.28284	31	88	57	50	40.3051	4.00E-07	7.00E-07	3.00E-07	6.00E-07	2.12E-07	2280	3420
0.98	2.28	1.3	1.84667	0.91924	2	6	4	4.66667	2.82843	1	10	9	7	6.36396	1	3	2	2.33	1.41421	6.941	195.084	188.143	132.37	133.037	1.35	1.45	0.1	1.38	0.07071	12	68	56	49.3333	39.598	9.50E-08	1.06E-07	1.10E-08	1.02E-07	7.78E-09	2680	6000
1.36	2.2	0.84	2.095	0.36053	1	5	4	2.875	2.46555	1	10	9	4.625	5.4652	1	3	2	2	1.29777	1.00794	106.42	105.412	46.031	63.3286	0.25	1.6	1.35	0.85	0.78271	19	103	84	79.75	36.127	0	0	0	0	0	0	0
1.55	3.04	1.49	2.146	1.05359	2	4	2	3.2	1.41421	7	15	8	10.2	5.65685	2	3	1	2.6	0.70711	14.0067	54.938	40.9313	38.5655	28.9428	0.65	1.4	0.75	1.1	0.53033	60	100	40	76	28.2843	0	0	0	0	0	333.6	5150
1.24	1.61	0.37	1.5175	0.26163	3	8	5	4.25	3.53553	13	14	1	13.25	0.70711	2	3	1	2.25	0.70711	26.9815	167.259	140.277	62.0509	99.1911	1.25	1.75	0.5	1.38	0.35355	22	80	58	65.5	41.0122	2.70E-08	8.60E-07	8.33E-07	2.35E-07	5.89E-07	2830	5100
1.2	3.04	1.84	2.24667	0.96093	2	8	6	4.33333	3.16228	9	17	8	13	3.59487	2	3	1	2.33	0.5547	10.811	151.964	141.153	70.4427	73.3384	0.65	1.85	1.2	1.17	0.6016	18	100	82	70	43.6278	0	0	0	0	0	0	0
1.2	2.01	0.81	1.62	0.40583	4	8	4	5.33333	2.3094	9	14	5	11.6667	2.51661	2	3	1	2.67	0.57735	65.409	151.964	86.555	96.671	48.0214	1.25	1.85	0.6	1.48	0.32146	18	84	66	59.3333	36.0185	6.00E-08	0.0005	0.0005	0.00017	0.00029	0	0
1.1	1.65	0.55	1.46667	0.38891	4	8	4	5.33333	2.82843	3	12	9	9	6.36396	3	3	0	3	0	65.409	138.905	73.4965	89.9078	51.9699	1.35	1.95	0.6	1.55	0.42426	33	76	43	61.6667	30.4056	6.00E-08	6.15E-07	5.55E-07	2.45E-07	3.92E-07	2475	3700
1.38	2.58	1.2	1.99	0.60025	3	9	6	5.33333	3.21455	6	16	10	12	5.2915	2	3	1	2.33	0.57735	32.065	238.029	205.964	114.245	109.103	1	1.75	0.75	1.33	0.38188	45	94	49	74.3333	25.8908	2.80E-07	1.00E+23	1.00E+23	3.33E+22	5.77E+22	0	0
1.1	1.96	0.86	1.67333	0.60811	5	8	3	6	2.12132	11	14	3	13	2.12132	2	3	1	2.33	0.70711	118.71	158.925	40.2154	132.115	28.4365	1.45	1.75	0.3	1.55	0.21213	26	83	57	64	40.3051	1.15E-07	1.15E-06	1.04E-06	4.60E-07	7.32E-07	2500	2620
0.79	3.16	2.37	2.03833	1.43713	2	6	4	3.16667	1.71888	1	17	16	9	10.2336	1	2	1	1.5	0.6396	6.941	132.905	125.964	42.1911	54.3986	1	2.6	1.6	1.42	0.72426	8	99	91	54.8333	56.5239	9.50E-08	1E+10	1E+10	5E+09	6.4E+09	0	0
2.2	3.04	0.84	2.48	0.59397	2	5	3	4	2.12132	10	15	5	11.6667	3.53553	2	3	1	2.67	0.70711	14.0067	106.42	92.4133	75.6156	65.3461	0.65	1.4	0.75	1.15	0.53033	69	100	31	79.3333	21.9203	0	0	0	0	0	333.6	3070
1.14	2.54	1.4	2.00333	0.75501	6	8	2	6.66667	1.1547	6	14	8	10.3333	4.04145	2	3	1	2.67	0.57735	144.242	207.2	62.958	182.803	33.7844	1.35	1.85	0.5	1.67	0.27538	30	82	52	60.6667	27.2274	2.20E-08	6.43E-07	6.21E-07	2.92E-07	3.18E-07	1260	2330
1.78	2.58	0.8	2.22286	0.37914	3	5	2	4.42857	1.11803	9	16	7	12.1429	3.64863	2	3	1	2.43	0.61237	32.065	114.818	82.753	86.0689	42.7032	1	1.55	0.55	1.31	0.26131	65	94	29	77.2857	14.9185	4.30E-08	1.00E+23	1.00E+23	2.86E+22	5.59E+22	0	0
1.38	3.98	2.6	3.244	1.20347	2	9	7	3.4	3.5	6	17	11	14.4	5.27968	2	3	1	2.2	0.5	15.9994	238.029	222.03	61.6049	110.278	0.5	1.75	1.25	0.79	0.6026	45	102	57	90.2	28.2555	0	0	0	0	0	0	0
0.79	3.44	2.65	2.22833	1.2055	2	6	4	3.66667	1.7581	1	16	15	11	6.39602	1	3	2	2.33	0.95346	15.9994	132.905	116.906	59.257	50.1499	0.6	2.6	2	1.31	0.85447	8	101	93	71	39.676	0	0	0	0	0	0	0
1.38	2.1	0.72	1.83	0.3923	4	9	5	6	2.64575	6	16	10	12	5.2915	2	3	1	2.33	0.57735	72.64	238.029	165.389	146.09	84.2305	1.25	1.75	0.5	1.47	0.25658	45	92	47	73.6667	25.1462	2.80E-07	0.0005	0.0005	0.0002	0.00026	2610	5400
0.93	2.19	1.26	1.39857	0.74429	3	5	2	3.28571	0.92582	1	15	14	6.57143	8.59817	1	3	2	1.57	0.96362	22.9898	112.411	89.4212	38.0454	40.4257	1	1.8	0.8	1.54	0.46194	11	90	79	42.7143	48.8299	4.90E-08	1.00E-07	5.10E-08	6.66E-08	2.95E-08	0	0
1.38	2.2	0.82	1.80429	0.32734	4	9	5	4.85714	2.57611	6	13	7	11.5714	3.72949	2	3	1	2.29	0.6742	69.723	238.029	168.306	99.0091	86.7831	1.3	1.75	0.45	1.38	0.23209	45	81	36	74.1429	18.8004	1.08E-07	2.80E-07	1.72E-07	1.55E-07	7.77E-08	2740	3155
0.79	3.98	3.19	2.64286	2.04432	2	6	4	3.42857	2.32993	1	17	16	10.2857	10.2644	1	2	1	1.57	0.65465	18.9984	132.905	113.907	54.5546	66.2274	0.5	2.6	2.1	1.29	1.24384	8	102	94	62.8571	59.8808	0	0	0	0	0	0	0
1.1	3.44	2.34	2.64571	1.24936	2	8	6	3.42857	2.72554	8	16	8	13.7143	4.78091	2	3	1	2.43	0.65465	15.9994	173.04	157.041	49.8182	70.4115	0.6	1.75	1.15	0.99	0.61688	17	101	84	77.5714	40.0428	0	0	0	0	0	317.5	4910
1.14	2.28	1.14	1.76	0.45715	5	8	3	5.42857	1.5667	6	13	7	11.4286	3.89639	2	3	1	2.29	0.6742	102.906	144.242	41.3365	117.32	17.5125	1.35	1.85	0.5	1.56	0.20226	30	79	49	70	25.4147	4.30E-08	6.43E-07	6.00E-07	1.55E-07	2.98E-07	1215	4700
0.82	3.44	2.62	2.39714	1.60931	2	9	7	3.57143	3.15096	1	16	15	10.2857	8.96421	1	3	2	1.86	0.84515	15.9994	238.029	222.03	54.3176	100.108	0.6	2.2	1.6	1.22	0.96686	10	101	91	67	53.8656	0	0	0	0	0	317.5	3155
1.91	2.54	0.63	2.02857	0.27771	4	6	2	4.85714	0.84515	10	14	4	12.4286	2.43487	2	3	1	2.43	0.65465	58.6934	196.967	138.273	112.742	57.1812	1.35	1.45	0.1	1.41	0.06547	67	83	16	76.5714	9.89589	2.20E-08	1.15E-07	9.30E-08	8.94E-08	4.41E-08	1740	4970
1.63	2.58	0.95	2.32429	0.44653	3	5	2	3.71429	1.16496	5	16	11	11.5714	6.77706	2	3	1	2.43	0.65465	32.065	95.94	63.875	53.0116	36.8952	1	1.45	0.45	1.18	0.27581	54	94	40	77.4286	25.3264	5.50E-08	1.00E+23	1.00E+23	5.71E+22	6.55E+22	0	0
1.24	3.44	2.2	2.66571	1.20702	2	8	6	3.42857	2.72554	8	16	8	13.4286	4.6291	2	3	1	2.43	0.65465	15.9994	167.259	151.26	48.9924	67.8879	0.6	1.75	1.15	0.99	0.61688	22	101	79	78.2857	38.1753	0	0	0	0	0	317.5	4910
1.63	2.58	0.95	2.17286	0.67175	3	4	1	3.42857	0.70711	5	16	11	11.2857	7.77817	2	3	1	2.43	0.70711	32.065	50.9415	18.8765	40.1549	13.3477	1	1.35	0.35	1.15	0.24749	54	94	40	76.8571	28.2843	2.00E-07	1.00E+23	1.00E+23	5.71E+22	7.07E+22	0	0
1.61	2.58	0.97	2.17571	0.61842	3	5	2	3.28571	0.92582	12	16	4	14.5714	2.22004	2	3	1	2.14	0.46291	26.9815	112.411	85.4295	42.0906	38.0906	1	1.55	0.55	1.15	0.25981	75	94	19	87.2857	10.4574	2.70E-08	1.00E+23	1.00E+23	5.71E+22	6.55E+22	0	0
1.61	2.55	0.94	2.15286	0.60687	3	4	1	3.71429	0.59761	12	16	4	14.5714	2.22004	2	3	1	2.14	0.46291	26.9815	78.96	51.9785	62.1732	30.0606	1.15	1.35	0.2	1.21	0.09636	76	93	17	86.8571	9.5244	1.00E-08	6.00E-08	5.00E-08	2.20E-08	2.27E-08	3350	5100
0.93	3.44	2.51	1.86857	1.44702	2	4	2	3.14286	0.84515	1	17	16	7.71429	10.2644	1	2	1	1.43	0.65465	15.9994	79.904	63.9046	38.2524	34.9882	0.6	1.8	1.2	1.44	0.58995	11	101	90	48.7143	57.6225	0	0	0	0	0	0	0
0.82	2.18	1.36	1.33286	0.80854	4	5	1	4.14286	0.46291	1	15	14	6.57143	8.59817	1	3	2	1.57	0.96362	39.0983	112.411	73.3127	59.8068	35.1407	1.15	2.2	1.05	1.81	0.62192	10	89	79	41.8571	48.9971	7.00E-08	3.30E-07	2.60E-07	1.47E-07	1.53E-07	0	0

1.22	2.05	0.83	1.8125	0.43712	5	8	3	5.75	1.64317	11	15	4	13.25	2.25832	2	3	[illegible]
0.93	3.44	2.51	2.7425	1.33154	2	3	1	2.5	0.63246	1	17	16	12.5	8.41427	1	2	[illegible]
0.79	2.58	1.79	1.7925	1.01451	3	8	5	5	2.68328	1	16	15	11.25	7.76531	1	3	[illegible]
2.2	3.98	1.78	3.535	1.25865	2	5	3	2.75	2.12132	10	17	7	15.25	4.94975	2	3	[illegible]
1.2	3.44	2.24	2.7075	1.01102	2	8	6	4.125	2.79029	10	17	7	15.125	2.50238	2	3	[illegible]
1.96	2.58	0.62	2.27	0.43841	3	5	2	4	1.41421	14	16	2	15	1.41421	2	2	[illegible]
2.01	2.1	0.09	2.06429	0.05471	4	6	2	5.14286	0.84515	14	16	2	15.4286	0.96362	2	2	[illegible]
1.54	2.1	0.56	1.89429	0.31803	4	5	1	4.57143	0.65465	4	16	12	11.4286	7.03055	2	3	[illegible]
0.82	3.44	2.62	2.715	1.39145	2	4	2	2.75	1.04881	1	17	16	12.5	8.41427	1	2	[illegible]
2.01	2.2	0.19	2.105	0.13435	4	5	1	4.5	0.70711	10	14	4	12	2.82843	2	3	[illegible]
2.01	2.28	0.27	2.145	0.19092	4	6	2	5	1.41421	10	14	4	12	2.82843	2	3	[illegible]
1.1	2.54	1.44	1.82	1.01823	6	8	2	7	1.41421	11	16	5	13.5	3.53553	3	3	[illegible]
1.13	3.44	2.31	2.73625	1.03622	2	8	6	4	2.76026	5	17	12	14.5	4.49338	2	3	[illegible]
0.82	2.02	1.2	1.12	0.84853	5	6	1	5.25	0.70711	1	15	14	4.5	9.89949	1	2	[illegible]
1.3	2.18	0.88	1.74	0.62225	4	6	2	5	1.41421	4	15	11	9.5	7.77817	2	3	[illegible]
1.81	2.1	0.29	2.01714	0.20506	4	5	1	4.71429	0.70711	13	16	3	15.1429	2.12132	2	2	[illegible]
1.25	2.55	1.3	1.78714	0.48065	2	8	6	4.57143	2.63523	8	15	7	12.5714	3.40751	2	3	[illegible]
1.24	3.44	2.2	2.7125	0.99973	2	8	6	4.125	2.79029	14	17	3	15.625	1.05785	2	3	[illegible]
1.25	3.16	1.91	2.6825	1.35057	3	8	5	4.25	3.53553	15	17	2	16.5	1.41421	2	3	[illegible]
1.1	3.44	2.34	2.095	1.00102	2	8	6	4.25	2.62996	3	16	13	11.5	5.91608	2	3	[illegible]
[illegible]																	

0.79	2.58	1.79	1.795	1.01226	3	8	5	5	2.68328	1	16	15	11.5	7.82304	1	3	[illegible]
0.98	2.18	1.2	1.51	0.682	2	4	2	3	1.26491	1	15	14	7	7.79744	1	3	[illegible]
1.6	2.54	0.94	1.835	0.66468	5	6	1	5.25	0.70711	5	11	6	6.5	4.24264	3	3	[illegible]
1.36	2.55	1.19	2.06	0.71818	2	5	3	3.125	1.5131	3	14	11	9.125	6.63127	2	3	[illegible]
0.95	2.2	1.25	1.6775	0.57386	5	6	1	5.25	0.54772	2	13	11	9.25	5.68331	1	3	[illegible]
2.05	3.44	1.39	2.97667	0.98288	2	5	3	3	2.12132	15	16	1	15.6667	0.70711	2	2	[illegible]
0.89	3.44	2.55	2.618	1.40886	2	6	4	3.2	2.13809	2	16	14	11.8	7.48331	1	3	[illegible]
0.82	2.18	1.36	1.57625	0.73292	4	5	1	4.375	0.59761	1	15	14	8.25	7.19126	1	3	[illegible]
0.89	2.2	1.31	1.6625	0.60441	5	6	1	5.5	0.63246	2	13	11	9.25	5.68331	1	3	[illegible]
0.93	2.55	1.62	1.88	0.97678	3	5	2	3.75	0.8584	1	16	15	10.25	9.30761	1	2	[illegible]
0.82	3.44	2.62	2.631	1.35812	2	5	3	2.75	1.54006	1	16	15	11.8	7.75258	1	3	[illegible]
0.93	3.44	2.51	2.55	1.47639	2	6	4	3	2.0702	1	16	15	10.8	8.67673	1	3	[illegible]
1.9	2.58	0.68	2.25875	0.42115	3	5	2	3.625	0.90321	11	16	5	14	3.04354	2	3	[illegible]
0.98	2.58	1.6	1.935	0.8617	2	5	3	3.25	1.3784	1	16	15	9.5	8.41427	1	3	[illegible]
1.63	2.54	0.91	1.8575	0.64347	4	6	2	4.5	1.41421	5	11	6	6.5	4.24264	3	3	[illegible]
1.63	1.96	0.33	1.7125	0.23335	4	5	1	4.25	0.70711	5	14	9	7.25	6.36396	2	3	[illegible]

[illegible]

1	2.55	1.55	1.85625	0.93664	4	4	0	4	0	2	16	14	10.25	8.45421	1	3	2	1.75
1.81	3.44	1.63	2.8075	0.8641	2	6	4	3.5	2.09762	11	16	5	14	2.68328	2	3	1	2.25
1.1	1.9	0.8	1.5	0.56569	4	8	4	6	2.82843	11	16	5	13.5	3.53553	3	3	0	3
2.04	2.55	0.51	2.48625	0.36062	2	2	0	2	0	13	14	1	13.875	0.70711	2	2	0	2
2.04	3.04	1	2.54	0.70711	2	2	0	2	0	13	15	2	14	1.41421	2	2	0	2
0.79	3.16	2.37	2.26	1.24737	3	6	3	4.25	1.64317	1	17	16	11.5	8.27043	1	3	2	2
1.65	2.18	0.53	2.005	0.27372	4	4	0	4	0	12	15	3	14	1.54919	2	3	1	2.25
1.9	2.55	0.65	2.225	0.45962	2	3	1	2.5	0.70711	14	14	0	14	0	2	2	0	2
1.24	2.2	0.96	1.765	0.43308	4	8	4	5.25	2.07364	10	14	4	12.5	1.89737	2	3	1	2.5
0.98	3.16	2.18	1.7825	1.42221	2	3	1	2.25	0.59409	1	17	16	6.75	10.2153	1	2	1	1.38
1.2	3.44	2.24	2.2725	0.95378	2	8	6	4.5	2.51661	10	16	6	13.25	3.20156	2	3	1	2.5
1.61	3.44	1.83	2.6725	1.00604	1	3	2	2	0.89443	1	16	15	11.5	7.82304	1	2	1	1.75
1.13	3.04	1.91	1.642	0.99281	2	8	6	6.2	3.20713	5	15	10	8.6	5.95219	2	3	1	2.6
1.78	1.91	0.13	1.845	0.09192	4	5	1	4.5	0.70711	10	13	3	11.5	2.12132	2	3	1	2.5
1.96	2.55	0.59	2.30857	0.36915	4	6	2	4.71429	1.16496	12	16	4	14.5714	2.32993	2	3	1	2.29
0.89	2.58	1.69	2.0625	0.87847	3	6	3	4.25	1.64317	2	16	14	11	7.26636	1	3	2	2
1.1	2.54	1.44	2.18	1.01823	6	8	2	6.5	1.41421	11	16	5	12.25	3.53553	3	3	0	3
0.89	3.44	2.55	2.1875	1.62563	2	6	4	3.625	2.14599	2	16	14	9	9.0844	1	2	1	1.5
1.14	1.9	0.76	1.52	0.5374	3	8	5	5.5	3.53553	6	14	8	10	5.65685	2	3	1	2.5
1.81	1.91	0.1	1.8725	0.07071	4	4	0	4	0	10	13	3	11.125	2.12132	2	3	1	2.63
0.93	2.55	1.62	1.9075	0.86618	3	5	2	4	0.89443	1	16	15	9.5	8.41427	1	3	2	2
1.14	2.2	1.06	1.99375	0.59002	1	8	7	2.125	3.63212	1	13	12	3.125	6.39411	1	3	2	1.38
1.54	2.58	1.04	2.32	0.73539	3	4	1	3.25	0.70711	4	16	12	13	8.48528	2	3	1	2.25
1.13	2.2	1.07	1.9925	0.59447	1	8	7	2.125	3.63212	1	13	12	3	6.27572	1	3	2	1.38
1.54	2.2	0.66	1.87	0.41742	1	5	4	3.5	1.89737	1	10	9	4.75	4.13521	1	3	2	2.5
2.04	3.04	1	2.54	0.70711	2	2	0	2	0	13	15	2	14	1.41421	2	2	0	2
0.95	3.04	2.09	2.1425	1.16552	2	5	3	3.25	1.64317	2	15	13	9	7.64199	1	3	2	2
0.79	2.18	1.39	1.76125	0.56002	4	6	2	4.75	0.8584	1	15	14	11.75	5.57249	1	3	2	2.38
0.89	3.98	3.09	3.38125	1.716	2	6	4	2.875	2.40992	2	17	15	14.125	8.28112	1	3	2	2
1.63	2.58	0.95	2.105	0.67175	3	4	1	3.5	0.70711	5	16	11	10.5	7.77817	2	3	1	2.5
0.93	3.44	2.51	2.4575	1.33496	2	6	4	3.25	2.07364	1	16	15	12	8.04984	1	2	1	1.75
0.89	3.44	2.55	2.20625	1.60172	2	6	4	3.25	1.80642	2	16	14	9	9.0844	1	2	1	1.5

1.3	3.44	2.14	2.2075	0.90138	2	9	7	4.5	3.10913	4	16	12	11.5	5.44671	2	3
1.22	2.55	1.33	1.93667	0.67099	2	5	3	3	1.73205	3	14	11	10	6.08276	2	3
1.12	2.02	0.9	1.42	0.6364	6	8	2	7.33333	1.41421	4	15	11	7.66667	7.77817	2	3
0.82	3.44	2.62	2.715	1.39145	2	5	3	3	1.54919	1	17	16	12.5	8.41427	1	2
0.82	2.55	1.73	1.9375	0.89681	4	4	0	4	0	1	16	15	10.25	7.91833	1	3
1.62	2.55	0.93	2.10875	0.45821	4	6	2	4.25	0.8584	11	16	5	13.125	3.00438	2	3
1.62	2.2	0.58	2.055	0.41012	5	6	1	5.25	0.70711	10	13	3	10.75	2.12132	2	3
0.82	2.18	1.36	1.765	0.5491	4	5	1	4.625	0.62828	1	15	14	11.75	5.57249	1	3
0.93	3.44	2.51	2.2525	1.50693	2	8	6	3.75	3.14643	1	16	15	10.75	7.76531	1	3
1.1	2.18	1.08	1.84	0.55943	4	8	4	5	2.19089	11	15	4	13	2.52982	2	3
2.2	3.16	0.96	2.92	0.67882	3	5	2	3.5	1.41421	8	17	9	14.75	6.36396	2	3
1.81	3.44	1.63	2.7225	0.92418	1	4	3	2.25	1.3784	1	16	15	11.5	7.82304	1	2
0.82	2.18	1.36	1.765	0.5491	4	5	1	4.5	0.64889	1	15	14	11.75	5.57249	1	3
1.22	2.18	0.96	1.87	0.49623	4	5	1	4.25	0.54772	3	15	12	11	6.19677	2	3
1.54	2.2	0.66	1.9425	0.35059	1	4	3	2.5	1.89737	1	8	7	3.5	3.63318	1	3
1.3	2.55	1.25	2.2375	0.88388	4	6	2	4.5	1.41421	4	16	12	13	8.48528	2	3
0.98	2.19	1.21	1.2825	0.8556	2	3	1	2.25	0.70711	1	15	14	4.5	9.89949	1	2
1.54	2.55	1.01	1.8975	0.52356	2	5	3	3.75	1.3784	4	14	10	9	6.32456	2	3
0.98	2.58	1.6	2.08	0.82946	2	4	2	3	0.89443	1	16	15	12	8.04984	1	2
1.36	3.44	2.08	2.695	1.08006	2	6	4	3.5	2.09762	3	16	13	11.5	6.72309	2	3
1.54	2	0.46	1.655	0.32527	4	6	2	4.5	1.41421	4	12	8	6	5.65685	3	3
[illegible]	[illegible]	[illegible]	[illegible]	[illegible]	[illegible]	[illegible]	[illegible]	[illegible]	[illegible]	[illegible]	[illegible]	[illegible]	[illegible]	[illegible]	[illegible]	[illegible]

[illegible]

1.17	2.66	1.49	2.13667	0.83811	3	8	5	5.33333	2.51661	8	17	9	13.6667	4.93288	2	3	[illegible]
1.33	2.01	0.68	1.8125	0.35692	4	5	1	4.25	0.54772	4	14	10	10.75	5.1672	2	3	[illegible]
1.54	2.18	0.64	1.7	0.45255	4	4	0	4	0	4	15	11	6.75	7.77817	2	3	[illegible]
1.38	3.44	2.06	2.2275	0.87504	2	9	7	4.5	3.10913	6	16	10	12	4.54606	2	3	[illegible]
0.82	3.98	3.16	3.36	1.76726	2	4	2	2.5	1.35873	1	17	16	14.75	8.21818	1	2	[illegible]
1.5	2.55	1.05	1.84	0.54266	2	6	4	4.5	2.09762	5	14	9	9.25	5.39444	2	3	[illegible]
1.3	2.66	1.36	2.32	0.96167	5	6	1	5.25	0.70711	4	17	13	13.75	9.19239	2	3	[illegible]
0.98	3.04	2.06	1.9525	1.0129	1	2	1	1.625	0.59761	1	15	14	4.5	7.48331	1	2	[illegible]
1.54	2.96	1.42	2.605	1.00409	4	4	0	4	0	4	17	13	13.75	9.19239	2	3	[illegible]
0.98	1.61	0.63	1.295	0.44548	2	3	1	2.5	0.70711	1	13	12	7	8.48528	1	2	[illegible]
2.16	3.44	1.28	3.12	0.9051	2	5	3	2.75	2.12132	6	16	10	13.5	7.07107	2	3	[illegible]
1.81	1.91	0.1	1.8725	0.07071	4	4	0	4	0	10	13	3	11.125	2.12132	2	3	[illegible]
1.6	3.44	1.84	2.78727	1.18787	2	5	3	3.09091	1.98431	5	16	11	13.2727	5.74728	2	3	[illegible]
1.69	2.55	0.86	2.2125	0.42872	4	5	1	4.125	0.40825	11	16	5	14	2.69037	2	3	[illegible]
1.6	2.55	0.95	1.89	0.49404	2	5	3	4	1.54919	5	14	9	9.25	5.39444	2	3	[illegible]
1.1	2.18	1.08	1.84	0.55943	4	8	4	5	2.19089	11	16	5	14.25	2.42899	2	3	[illegible]
2.54	2.96	0.42	2.75	0.29698	4	6	2	5	1.41421	11	17	6	14	4.24264	2	3	[illegible]
1.1	2.2	1.1	1.65	0.77782	5	8	3	6.5	2.12132	10	16	6	13	4.24264	3	3	[illegible]
1.27	1.69	0.42	1.585	0.29698	5	8	3	5.75	2.12132	12	17	5	13.25	3.53553	3	3	[illegible]
[illegible]																	

1.2	2.2	1	1.83	0.47649	5	8	3	6	1.54919	9	14	5	11.5	3.16228	2	3	1	2.5	0.63246	118.71	192.217	73.507
2.36	3.16	0.8	3.04571	0.56569	3	6	3	3.42857	2.12132	6	17	11	15.4286	7.77817	2	3	1	2.14	0.70711	35.453	183.84	148.387
0.95	3.98	3.03	3.31125	1.83852	2	5	3	2.625	1.74312	2	17	15	13.75	8.97004	1	3	2	2	0.78446	18.9984	87.62	68.6216
1.78	3.44	1.66	2.8	0.878	2	6	4	3.75	2.25832	11	16	5	14	2.68328	2	3	1	2.25	0.54772	15.9994	196.967	180.967
1	2.2	1.2	1.78	0.58296	4	5	1	4.75	0.54772	2	14	12	10	6.19677	1	3	2	2	0.89443	40.078	118.71	78.632
1.1	2.55	1.45	1.74429	0.54767	2	8	6	4.57143	2.63523	8	14	6	11.4286	2.91548	2	3	1	2.57	0.57735	12.0107	158.925	146.915
1.14	2.1	0.96	1.86	0.67882	5	8	3	5.75	2.12132	6	16	10	13.5	7.07107	2	3	1	2.25	0.70711	127.6	144.242	16.642
1.63	3.44	1.81	2.63625	1.04859	2	4	2	3	1.29777	5	16	11	12.75	4.84496	2	3	1	2.5	0.64889	15.9994	63.546	47.5466
1.12	1.65	0.53	1.5175	0.37477	4	8	4	5	2.82843	4	12	8	10	5.65685	3	3	0	3	0	65.409	140.116	74.707
1.1	2.28	1.18	1.735	0.53077	5	8	3	5.75	1.64317	9	16	7	12.75	3.14643	2	3	1	2.5	0.63246	102.906	173.04	70.1345
1.12	3.16	2.04	2.65	1.4425	3	8	5	4.25	3.53553	4	17	13	13.75	9.19239	2	3	1	2.25	0.70711	35.453	140.116	104.663
1.22	2.2	0.98	1.76	0.44281	4	8	4	5.25	2.07364	10	13	3	12	1.54919	2	3	1	2.5	0.63246	69.723	162.5	92.777
2.01	2.28	0.27	2.2125	0.19092	4	6	2	5.5	1.41421	10	14	4	11	2.82843	2	3	1	2.75	0.70711	72.64	195.084	122.444
1.23	1.91	0.68	1.57	0.48083	4	8	4	6	2.82843	10	13	3	11.5	2.12132	3	3	0	3	0	58.6934	164.93	106.237
1.23	2.2	0.97	1.7625	0.43794	4	8	4	5.25	2.07364	10	13	3	12.25	1.64317	2	3	1	2.5	0.63246	69.723	164.93	95.2073
0.95	2.02	1.07	1.67	0.55274	5	6	1	5.5	0.63246	2	15	13	11	6.75278	1	3	2	2	0.89443	87.62	208.98	121.36
0.79	2	1.21	1.395	0.8556	6	6	0	6	0	1	12	11	6.5	7.77817	1	3	2	2	1.41421	132.905	200.59	67.6845
2.33	2.58	0.25	2.455	0.17678	3	6	3	4.5	2.12132	14	16	2	15	1.41421	2	2	0	2	0	32.065	207.2	175.135
1.22	3.44	2.22	2.125	0.96231	2	5	3	3.5	1.29099	3	16	13	11.5	5.91608	2	3	1	2.5	0.57735	15.9994	88.9059	72.9065
0.82	3.44	2.62	2.39714	1.60931	2	9	7	3.85714	3.27327	1	16	15	10.2857	8.96421	1	3	2	1.86	0.84515	15.9994	238.029	222.03
1.14	2.01	0.87	1.7675	0.46116	4	8	4	5	2.19089	6	14	8	11	4.19524	2	3	1	2.5	0.63246	58.6934	144.242	85.5486
1.1	2.2	1.1	1.82	0.56879	5	8	3	5.75	1.64317	3	13	10	9	4.64758	2	3	1	2.75	0.54772	106.42	138.905	32.4855
1.25	1.93	0.68	1.76	0.48083	5	8	3	5.75	2.12132	11	15	4	12	2.82843	3	3	0	3	0	107.868	168.934	61.066
1.3	2.55	1.25	1.7775	0.65911	2	6	4	4.75	2.07364	4	14	10	9	6.32456	2	3	1	2.5	0.63246	12.0107	178.49	166.479
1.12	2.05	0.93	1.7825	0.48919	4	8	4	5.5	1.89737	4	15	11	11	5.72713	2	3	1	2.5	0.63246	58.6934	140.116	81.4226
2.04	2.55	0.51	2.48625	0.36062	2	2	0	2	0	13	14	1	13.875	0.70711	2	2	0	2	0	10.811	12.0107	1.1997
1.17	2.19	1.02	1.8625	0.52744	3	8	5	4.5	2.60768	8	15	7	12.25	3.72827	2	3	1	2.5	0.63246	30.9738	150.36	119.386
1.24	1.91	0.67	1.575	0.47376	4	8	4	6	2.82843	10	14	4	12	2.82843	3	3	0	3	0	58.6934	167.259	108.566

1.65	3.98	2.33	3.46625	1.41267	2	5	3	2.625	1.74312	10	17	7	15.5	4.151	2	3
1.1	1.9	0.8	1.6825	0.42693	3	8	5	4.5	2.60768	3	14	11	9.75	5.82237	2	3
1.14	3.44	2.3	2.1475	0.96393	2	8	6	4.5	2.51661	6	16	10	11.25	4.99166	2	3
1.2	3.44	2.24	2.1625	0.94327	2	8	6	4.5	2.51661	8	16	8	12.25	3.86221	2	3
2.1	2.66	0.56	2.405	0.32564	5	5	0	5	0	10	17	7	15	3.68782	2	3
1.12	2.54	1.42	1.83	1.00409	6	8	2	7	1.41421	4	11	7	7.5	4.94975	3	3
1.54	2.54	1	2.1	0.56682	4	6	2	5.25	1.04881	4	13	9	9.75	4.32435	2	3
0.82	3.44	2.62	2.235	1.59904	2	4	2	3	1.29777	1	16	15	9.125	9.14071	1	3
1.6	1.91	0.31	1.7575	0.19295	3	5	2	4	0.89443	5	13	8	9.5	3.63318	2	3
1.36	2.2	0.84	1.93	0.43433	4	5	1	4.75	0.54772	3	14	11	9.25	5.00999	2	3
1.38	3.44	2.06	3.01857	1.0985	2	9	7	3	3.65563	6	16	10	14.2857	5.15223	2	3
1.2	2.55	1.35	1.91	0.71881	2	8	6	4.28571	3.08221	6	14	8	10.5714	4.12311	2	3
1.81	2.58	0.77	2.225	0.45224	3	5	2	3.75	1.04881	11	16	5	14	2.68328	2	3
1.93	2.58	0.65	2.14667	0.45962	3	5	2	4.33333	1.41421	11	16	5	12.6667	3.53553	2	3
1.78	2.55	0.77	2.195	0.45224	4	5	1	4.25	0.54772	11	16	5	14	2.68328	2	3
0.79	3.98	3.19	3.35875	1.7765	2	6	4	2.875	2.40992	1	17	16	13.875	8.9249	1	3
0.93	3.04	2.11	1.47625	1.1148	2	3	1	2.375	0.59761	1	15	14	4.5	7.48331	1	2
1.13	3.44	2.31	2.1475	0.96755	2	8	6	4.25	2.62996	5	16	11	11	5.35413	2	3
1.78	2.55	0.77	2.1725	0.47769	4	5	1	4.25	0.54772	13	16	3	14.5	1.89737	2	2
1.24	2.1	0.86	1.885	0.60811	5	8	3	5.75	2.12132	14	16	2	15.5	1.41421	2	3
1.3	2.2	0.9	1.8625	0.6364	5	9	4	6.5	2.82843	4	10	6	7.75	4.24264	3	3
0.89	3.44	2.55	2.795	1.17582	2	6	4	2.625	1.43456	2	16	14	14.125	5.0476	1	3
0.82	3.16	2.34	2.07	0.74592	2	4	2	3.5	0.67928	1	17	16	10.8966	6.69201	1	3

[illegible]

1.1	3.44	2.34	2.66	1.31844	2	8	6	3.6	3.11677	3	16	13	12	7.02038	2	3
1.88	3.44	1.56	2.66	1.10309	2	4	2	3	1.41421	9	16	7	12.5	4.94975	2	3
0.98	3.44	2.46	2.435	1.33343	2	4	2	2.5	1.09545	1	16	15	10.5	7.82304	1	3
0.82	3.44	2.62	1.19429	1.85262	2	5	3	4.57143	2.12132	1	16	15	3.14286	10.6066	1	2
1.33	3.98	2.65	3.38875	1.62218	2	5	3	2.625	1.74312	4	17	13	14.625	7.01372	2	3
0.95	3.44	2.49	2.76909	1.24285	2	5	3	2.63636	1.32574	2	16	14	13.3636	6.43617	1	3
0.93	3.44	2.51	2.62647	0.95372	1	3	2	1.58824	0.77311	1	16	15	7.88235	9.44859	1	2
1.1	3.44	2.34	2.71857	1.38043	2	8	6	3.85714	3.54799	3	16	13	11.9286	7.74407	2	3
1	3.44	2.44	2.707	1.20732	2	4	2	2.8	1.29212	2	16	14	12.15	6.9191	1	3
0.98	3.44	2.46	2.78687	1.18257	2	3	1	2.3125	0.63131	1	16	15	14.375	4.90785	1	2
1	3.44	2.44	2.666	1.33672	2	4	2	2.8	1.30931	2	16	14	12.8	7.28991	1	2
2.04	2.2	0.16	2.15	0.11314	1	2	1	1.3125	0.70711	1	13	12	4.75	8.48528	1	2
1.2	3.16	1.96	2.44903	1.16242	2	8	6	4.48387	3.21809	10	17	7	14.3548	4.15136	2	3
1.55	3.44	1.89	2.83467	1.00313	2	6	4	2.93333	1.81325	7	16	9	14.2667	3.88665	2	3
0.98	3.44	2.46	2.715	1.03844	1	3	2	1.875	0.74001	1	16	15	10.375	8.96421	1	2
1	2.01	1.01	1.53125	0.54278	3	4	1	3.75	0.53452	2	14	12	9.25	6.94879	1	2
1.3	2.66	1.36	2.32	0.96167	5	9	4	6	2.82843	4	17	13	13.75	9.19239	2	3
1.9	3.98	2.08	3.5	0.94742	2	6	4	2.625	1.75594	7	17	10	15.5	4.37163	2	3
1.36	3.44	2.08	2.608	1.47078	2	4	2	2.8	1.41421	3	16	13	10.8	9.19239	2	3
1.78	3.44	1.66	2.776	1.1738	2	5	3	3.2	2.12132	13	16	3	14.8	2.12132	2	2
0.93	3.44	2.51	2.60967	1.16653	1	4	3	2.23333	0.9334	1	16	15	10.9333	8.18024	1	3
0.79	3.44	2.65	2.50571	1.53886	2	6	4	3.57143	2.43487	1	16	15	10.5714	8.77089	1	3
1	3.44	2.44	2.55143	1.37576	2	4	2	2.85714	1.30931	2	16	14	11.4286	7.2752	1	3

0.98	2.01	1.03	1.41	0.53563	2	8	6	4.66667	3.05505	1	14	13	9.66667	7.50555	1	3
1.91	3.44	1.53	2.99	0.91162	2	4	2	2.66667	1.34164	10	16	6	15.1111	2.63629	2	3
1.2	3.44	2.24	2.86222	1.01138	2	8	6	3.44444	2.55343	10	17	7	15.4444	2.48193	2	3
1.1	2.2	1.1	1.75333	0.57839	5	8	3	6	1.73205	10	16	6	13.3333	3.05505	2	3
0.89	3.44	2.55	1.99667	1.02788	2	6	4	4	1.54919	2	16	14	11.3333	6.07454	1	3
0.98	2.01	1.03	1.54667	0.52272	2	4	2	3.33333	1.1547	1	14	13	9	7	1	3
0.95	3.44	2.49	2.85444	1.25305	2	5	3	3	2.01246	2	16	14	14.2222	6.17657	1	2
0.98	2.01	1.03	1.39667	0.54243	2	8	6	4.66667	3.05505	1	14	13	8.33333	6.65833	1	3
0.89	3.44	2.55	2.48111	1.3853	2	8	6	3.55556	2.62298	2	16	14	11.3333	6.86731	1	3
0.82	3.16	2.34	2.46111	1.43462	3	4	1	3.33333	0.67082	1	17	16	12.3333	9.67471	1	3
1.54	2.04	0.5	1.7	0.3222	2	4	2	3.77778	0.89443	4	14	10	7.22222	6.49615	2	3
0.89	3.98	3.09	3.03444	1.92552	2	6	4	3.11111	2.36643	2	17	15	13.1111	8.73212	1	3
1.83	2.19	0.36	1.95	0.25456	3	4	1	3.66667	0.70711	8	15	7	10.3333	4.94975	2	3
1.1	2.2	1.1	1.82667	0.62939	4	8	4	5.66667	2.08167	10	16	6	13.6667	3.21455	2	3
0.93	1.96	1.03	1.23889	0.63312	3	5	2	3.55556	1.18322	1	14	13	5.11111	8.30963	1	3
0.79	3.44	2.65	2.16333	1.63248	2	6	4	3.77778	2.58199	1	16	15	9.11111	9.45604	1	2
0.93	3.04	2.11	2.29609	0.7599	1	4	3	1.56522	1.11035	1	15	14	5.04348	8.33009	1	3
0.79	3.16	2.37	2.52667	1.38662	3	6	3	4	2.01246	1	17	16	12.5556	9.46573	1	3

1.13	2.2	1.07	1.67333	0.53519	5	8	3	6	1.73205	5	12	7	9	3.60555	3	3	0	3	0	106.42	140.908
1.22	2.2	0.98	1.68	0.49275	5	8	3	6.33333	1.52753	10	13	3	11.6667	1.52753	2	3	1	2.67	0.57735	106.42	204.383
2.36	2.55	0.19	2.42333	0.13435	2	6	4	4.66667	2.82843	6	14	8	8.66667	5.65685	2	3	1	2.67	0.70711	12.0107	183.84
1.3	1.9	0.6	1.7	0.34641	3	6	3	5	1.73205	4	14	10	8.33333	5.1316	2	3	1	2.67	0.57735	28.0855	186.207
1.12	2.58	1.46	2.09333	1.03238	3	8	5	4.66667	3.53553	4	16	12	12	8.48528	2	3	1	2.33	0.70711	32.065	140.116
1.17	2.2	1.03	1.79	0.54617	5	8	3	6.33333	1.52753	8	12	4	10	2	3	3	0	3	0	106.42	200.59
1.14	1.9	0.76	1.55	0.38354	3	8	5	5	2.64575	6	13	7	10	3.60555	2	3	1	2.67	0.57735	26.9815	144.242
1.12	2.2	1.08	1.7	0.54443	5	8	3	6	1.73205	4	13	9	9	4.58258	2	3	1	2.67	0.57735	106.42	140.116
0.79	3.16	2.37	2.42667	1.48896	3	9	6	4.33333	2.92404	1	17	16	12	10.1292	1	3	2	1.89	0.80623	35.453	232.038
1.55	2.01	0.46	1.72	0.25239	4	5	1	4.33333	0.57735	5	14	9	8.66667	4.72582	2	3	1	2.67	0.57735	54.938	92.9064
1.1	1.91	0.81	1.54	0.40951	3	8	5	5	2.64575	10	16	6	13	3	2	3	1	2.67	0.57735	26.9815	173.04
1.24	1.96	0.72	1.71	0.40731	5	8	3	6	1.73205	11	14	3	13	1.73205	2	3	1	2.67	0.57735	107.868	167.259
1.1	2.54	1.44	1.68889	0.55651	4	8	4	5.88889	1.8846	11	16	5	14	1.9298	2	3	1	2.44	0.58722	72.64	196.967
1.25	1.9	0.65	1.58667	0.32563	3	8	5	5	2.64575	11	15	4	13	2	2	3	1	2.67	0.57735	26.9815	168.934
0.93	2.19	1.26	1.35667	0.72176	3	5	2	3.66667	1.1547	1	15	14	6	7.81025	1	2	1	1.33	0.57735	22.9898	87.62
1.12	2.28	1.16	1.57	0.62217	3	8	5	5.66667	2.51661	2	10	8	5.33333	4.16333	1	3	2	2.33	1.1547	24.305	195.084
1.24	2.2	0.96	1.74	0.48125	5	8	3	6	1.73205	10	14	4	12.3333	2.08167	2	3	1	2.67	0.57735	106.42	167.259
0.95	3.98	3.03	2.45778	1.24169	2	5	3	3.33333	1.29904	2	17	15	11.3333	6.7708	1	3	2	2	0.75	15.9994	87.62
1.3	2.2	0.9	1.8	0.45826	3	6	3	4.66667	1.52753	4	14	10	8.66667	5.03322	2	3	1	2.67	0.57735	28.0855	178.49
0.95	3.98	3.03	2.45111	1.24094	2	5	3	3.55556	1.31101	2	17	15	11.3333	6.7708	1	3	2	2	0.75	15.9994	87.62
0.93	3.44	2.51	2.22556	1.54426	2	3	1	2.44444	0.6455	1	16	15	9.11111	9.45604	1	2	1	1.56	0.6455	12.0107	22.9898
1.14	2.28	1.14	1.79333	0.58799	5	8	3	6.33333	1.52753	6	14	8	10	4	2	3	1	2.67	0.57735	118.71	195.084
1.55	3.44	1.89	2.95	1.01584	2	4	2	2.66667	1.34164	7	16	9	14.7778	3.95601	2	3	1	2.11	0.44721	15.9994	74.9216
1.25	1.93	0.68	1.69333	0.38423	3	8	5	5.33333	2.51661	11	15	4	13.3333	2.08167	2	3	1	2.67	0.57735	28.0855	168.934

[illegible]

1.2	2.2	1	1.72667	0.50213	5	8	3	6	1.73205	10	13	3	11	1.73205	2	3	[illegible]
1.88	2.04	0.16	1.93333	0.11314	2	4	2	3.33333	1.41421	9	13	4	10.3333	2.82843	2	3	[illegible]
1.13	3.44	2.31	2.30889	1.0876	2	8	6	4.22222	2.55604	5	16	11	11.5556	5.82523	2	3	[illegible]
0.93	3.44	2.51	2.18667	1.53707	2	6	4	2.88889	1.55456	1	16	15	8.55556	9.18785	1	3	[illegible]
1.88	2.05	0.17	1.99333	0.12021	4	5	1	4.66667	0.70711	9	15	6	13	4.24264	2	3	[illegible]
1.36	2.55	1.19	2.025	0.84146	2	4	2	2.88235	1.41421	3	14	11	9.14706	7.77817	2	3	[illegible]
1.2	1.91	0.71	1.63	0.37802	4	8	4	5.66667	2.08167	10	13	3	11	1.73205	2	3	[illegible]
0.82	3.44	2.62	2.17667	1.61355	2	5	3	3.33333	1.93649	1	16	15	9.11111	9.45604	1	2	[illegible]
0.82	3.44	2.62	2.59556	1.21347	2	6	4	3.33333	1.38873	1	17	16	12.2222	7.91247	1	3	[illegible]
1.25	2.01	0.76	1.795	0.40135	4	8	4	5	2.19089	10	15	5	13.25	2.42899	2	3	[illegible]
1.24	2.28	1.04	1.82667	0.53267	5	8	3	6	1.73205	9	14	5	12.3333	2.88675	2	3	[illegible]
1.31	2.2	0.89	1.96111	0.52123	1	4	3	1.77778	1.61245	1	8	7	2	3.07409	1	3	[illegible]
1.3	2.28	0.98	1.84667	0.49973	5	6	1	5.33333	0.57735	4	14	10	9	5	2	3	[illegible]
0.98	2.04	1.06	1.45111	0.74953	2	2	0	2	0	1	13	12	6.33333	8.48528	1	2	[illegible]
1.2	1.81	0.61	1.64556	0.35762	4	8	4	4.88889	2.36643	7	13	6	11.6667	2.92404	2	3	[illegible]
1.27	1.91	0.64	1.59667	0.32021	3	8	5	5	2.64575	10	17	7	13.3333	3.51188	2	3	[illegible]
1.96	3.98	2.02	2.75556	0.89891	1	5	4	1.88889	1.43925	1	17	16	9.11111	8.79123	1	2	[illegible]
0.82	3.44	2.62	2.17667	1.61355	2	5	3	3.33333	1.93649	1	16	15	9.11111	9.45604	1	2	[illegible]
0.95	3.44	2.49	2.18333	0.97432	2	5	3	3.55556	1.19896	2	16	14	11.1111	6.08019	1	3	[illegible]
1.2	2.2	1	1.67	0.50269	3	8	5	5.33333	2.51661	10	13	3	11	1.73205	2	3	[illegible]
1.5	2.58	1.08	2.29778	0.62935	3	6	3	3.88889	1.8303	5	16	11	12.8889	6.55362	2	3	[illegible]
[illegible]																	

0.89	3.44	2.55	2.49556	1.38025	2	8	6	4	3.1749	2	16	14	[illegible]
1.2	2.1	0.9	1.47889	0.56606	4	8	4	6.88889	2.26936	10	16	6	[illegible]
1.24	1.81	0.57	1.58	0.3005	4	8	4	5.66667	2.08167	12	14	2	[illegible]
1.33	2.28	0.95	1.80667	0.47501	4	5	1	4.66667	0.57735	4	13	9	[illegible]
1.23	2.05	0.82	1.47889	0.50807	4	8	4	6.88889	2.26936	8	15	7	[illegible]
1.62	3.98	2.36	3.22444	1.52439	2	6	4	3	2.32379	13	17	4	[illegible]
0.89	3.44	2.55	2.55	1.48228	2	6	4	3.2	2.13809	2	16	14	[illegible]
1.3	2.28	0.98	1.79667	0.49014	4	9	5	6	2.64575	4	13	9	[illegible]
1.25	2.05	0.8	1.49222	0.49485	4	8	4	6.88889	2.26936	8	15	7	[illegible]
0.79	2.55	1.76	1.94556	0.84606	4	6	2	4.44444	1.03775	1	16	15	[illegible]
1.91	2.28	0.37	2.12667	0.19296	3	5	2	4	1	9	15	6	[illegible]
1.1	2.33	1.23	1.87667	0.67575	5	8	3	6.33333	1.52753	10	16	6	[illegible]
1.2	2.2	1	1.69667	0.50003	5	8	3	6	1.73205	10	12	2	[illegible]
0.82	3.98	3.16	2.98	2.02103	2	9	7	3.22222	3.1305	1	17	16	[illegible]
1.14	3.44	2.3	2.63636	1.49096	2	8	6	4	3.76743	5	16	11	[illegible]
1.17	2.2	1.03	1.71667	0.51791	5	8	3	6.33333	1.52753	8	13	5	[illegible]
[illegible]													

1.33	2.2	0.87	1.84667	0.45742	4	6	2	5	1	4	14	10	8.66667	5.03322	2	3	[illegible]
0.82	3.44	2.62	2.5075	1.35916	2	6	4	3.5	2.09762	1	16	15	11.75	7.91833	1	2	[illegible]
1.14	2.01	0.87	1.80444	0.50771	4	8	4	4.88889	2.36643	6	14	8	11.8889	4.66905	2	3	[illegible]
1.1	2.2	1.1	1.66333	0.55048	5	8	3	6	1.73205	3	12	9	8.33333	4.72582	3	3	[illegible]
0.82	3.98	3.16	2.69778	1.28885	2	4	2	2.22222	0.71842	1	17	16	11.1111	7.7418	1	2	[illegible]
1	3.44	2.44	1.49556	1.14075	2	4	2	3.77778	0.89443	2	16	14	6.22222	8.53229	1	2	[illegible]
1.78	2.66	0.88	2.04286	0.37352	5	5	0	5	0	13	17	4	14.8571	2.09762	2	2	[illegible]
1.12	3.44	2.32	2.75167	1.39232	2	8	6	3.33333	3.12694	4	16	12	12.1667	7.67753	2	3	[illegible]
2.2	3.98	1.78	3.545	1.18251	2	6	4	2.875	2.40992	10	17	7	15.25	4.75556	2	3	[illegible]
1.23	2.58	1.35	1.905	0.95459	3	8	5	5.5	3.53553	13	16	3	14.5	2.12132	2	3	[illegible]
2.04	3.04	1	2.28	0.44086	1	2	1	1.54167	0.63201	1	15	14	7.83333	8.01953	1	2	[illegible]
1.24	2.2	0.96	1.69667	0.4817	4	8	4	5.66667	2.08167	10	14	4	12	2	3	3	[illegible]
1.13	2.01	0.88	1.745	0.45865	4	8	4	5	2.19089	5	14	9	10.25	4.9295	2	3	[illegible]
1.62	2.55	0.93	2.1375	0.53016	4	6	2	4.5	1.09545	8	16	8	13.25	4.13521	2	3	[illegible]
0.79	3.16	2.37	2.43556	1.47572	3	9	6	4.33333	2.92404	1	17	16	12.2222	9.80816	1	3	[illegible]
0.79	3.98	3.19	3.07333	1.9046	2	6	4	3.22222	2.48998	1	17	16	12.6667	9.39149	1	3	[illegible]
0.79	2.96	2.17	2.36667	1.27715	4	6	2	4.55556	1.18322	1	17	16	13.1111	9.30591	1	2	[illegible]
1.12	1.9	0.78	1.57	0.40361	4	8	4	5.66667	2.08167	4	12	8	9	4.3589	3	3	[illegible]
1.33	2.2	0.87	1.90667	0.49943	3	6	3	4.66667	1.52753	4	15	11	9	5.56776	2	3	[illegible]
0.82	3.98	3.16	3.08	1.88843	2	5	3	2.77778	1.61245	1	17	16	12.6667	9.39149	1	3	[illegible]
1.14	3.44	2.3	2.68	1.35589	2	8	6	3.25	2.72246	6	16	10	12.5	6.21194	2	3	[illegible]
[illegible]																	

0.82	3.44	2.62	2.17667	1.61355	2	5	3	3.33333	1.93649	1	16	15	9.11111	9.45604	1	2
0.79	2.66	1.87	2.09333	1.15737	5	6	1	5.33333	0.67082	1	17	16	12	10.1292	1	3
0.95	3.44	2.49	2.53067	1.52274	2	5	3	3.2	1.97974	2	16	14	10.8	8.66305	1	3
1.38	3.44	2.06	2.924	1.31372	2	9	7	3.2	3.63146	5	16	11	13.2	7.11952	2	3
0.98	3.44	2.46	2.64455	1.30853	2	4	2	2.40909	0.85261	1	16	15	12.9091	7.13099	1	2
0.79	3.44	2.65	2.91857	1.14137	2	6	4	2.53571	1.34222	1	16	15	14	6.30006	1	3
1.13	2.55	1.42	2.01714	0.84222	4	8	4	5.14286	2.39046	5	16	11	11.4286	6.9949	2	3
1.1	2.2	1.1	1.76667	0.58595	5	8	3	6.33333	1.52753	3	12	9	8.33333	4.72582	3	3
1.28	1.91	0.63	1.6	0.31512	3	9	6	5.33333	3.21455	8	13	5	10.3333	2.51661	2	3
1.12	1.96	0.84	1.7375	0.4517	4	8	4	5.5	1.89737	4	14	10	10.5	5.17687	2	3
1.9	3.98	2.08	3.4675	1.3929	2	5	3	2.625	1.74312	11	17	6	15.875	3.27579	2	3
1.2	3.44	2.24	2.58	1.3444	2	8	6	4	3.4641	9	16	7	13.2857	4.22577	2	3
1.12	1.91	0.79	1.60333	0.4236	4	8	4	5.66667	2.08167	4	13	9	9	4.58258	2	3
1.1	2.01	0.91	1.79556	0.53127	4	8	4	4.88889	2.36643	3	14	11	11.2222	6.3914	2	3
1	3.04	2.04	1.89583	1.29616	2	4	2	3.16667	1.30664	2	15	13	7.83333	8.21955	1	3
0.89	3.44	2.55	2.775	1.37472	2	6	4	2.83333	1.83615	2	16	14	12.0833	8.01695	1	3
1.13	3.44	2.31	2.6084	1.63342	2	8	6	4.16	4.24264	5	16	11	12.04	7.77817	2	3
1.88	3.44	1.56	2.98667	0.91968	2	4	2	2.66667	1.34164	9	16	7	15	3.07409	2	3
2.19	3.44	1.25	3.05615	0.84752	2	5	3	2.46154	1.24035	8	16	8	15.1538	3.10087	2	3
0.98	3.44	2.46	2.58435	1.22339	2	4	2	2.21739	0.62335	1	17	16	11.4783	8.19407	1	2
0.95	3.44	2.49	2.67909	1.10881	2	5	3	2.63636	1.34751	2	16	14	13.2727	5.13502	1	3
1.2	2.18	0.98	1.935	0.69296	4	8	4	5	2.82843	9	15	6	13.5	4.24264	2	3
0.82	3.98	3.16	3.08889	1.88036	2	6	4	3.11111	2.26936	1	17	16	12.6667	9.39149	1	3

1.13	1.81	0.68	1.385	0.48083	4	8	4	6.5	2.82843	5	13	8	8	5.65685	2	3
2	3.98	1.98	3.52	1.25485	2	6	4	2.875	2.40992	9	17	8	15.375	4.56997	2	3
0.82	3.98	3.16	3.295	1.8904	2	4	2	2.5	1.35873	1	17	16	13.625	9.37878	1	3
1.83	2.19	0.36	1.97667	0.18903	3	4	1	3.66667	0.57735	8	15	7	11	3.60555	2	3
0.82	3.98	3.16	3.00667	1.97854	2	4	2	2.66667	1.34164	1	17	16	12	10.1292	1	3
1.14	2.28	1.14	1.93429	0.46706	5	8	3	5.42857	1.38873	6	14	8	11.4286	4.11443	2	3
1.91	1.96	0.05	1.935	0.03536	4	5	1	4.5	0.70711	10	14	4	12	2.82843	2	3
2.02	3.44	1.42	2.97375	0.8521	2	6	4	3	2.37635	14	16	2	15.5	0.97014	2	2
1.3	2.1	0.8	1.65556	0.56569	5	6	1	5.55556	0.70711	4	16	12	9.33333	8.48528	2	3
0.93	3.98	3.05	2.56778	1.47674	2	3	1	2.44444	0.60858	1	17	16	11.1111	8.76441	1	2
1.55	2.55	1	1.796	0.51766	2	5	3	3.8	1.30931	7	14	7	9.6	4.27618	2	3
1.22	2.02	0.8	1.52	0.56569	6	8	2	7.25	1.41421	12	15	3	13.125	2.12132	2	3
1.23	2.02	0.79	1.52625	0.55861	6	8	2	7.25	1.41421	13	15	2	13.75	1.41421	2	3
1.3	1.9	0.6	1.79714	0.30913	4	9	5	4.85714	2.57611	4	13	9	10.2857	3.96576	2	3
1.33	1.88	0.55	1.64	0.31967	4	5	1	4.6	0.61237	4	13	9	7.8	4.28661	2	3
1	2.28	1.28	1.59714	0.67663	4	6	2	4.57143	1.11803	2	13	11	7.42857	5.98435	1	3
1	2.2	1.2	1.60375	0.60666	4	6	2	4.5	1.06904	2	13	11	7.875	5.93416	1	3
1	2.28	1.28	1.62375	0.63734	4	5	1	4.25	0.53452	2	13	11	7.875	5.93416	1	3
0.82	3.98	3.16	3.04667	1.92431	2	6	4	2.88889	1.94936	1	17	16	12.3333	9.67471	1	3
1.22	1.91	0.69	1.6725	0.33721	4	5	1	4.75	0.54772	3	13	10	9.75	5.1672	2	3

1.62	3.44	1.82	2.81875	1.0849	2	6	4	3.125	2.04803	5	16	11	14.25	4.84195	2	3
0.93	3.44	2.51	2.688	1.34099	2	3	1	2.4	0.65465	1	16	15	12.8	7.90118	1	2
0.82	3.44	2.62	2.59143	1.41781	2	5	3	2.57143	1.34164	1	16	15	11.7143	8.66025	1	2
1.12	1.9	0.78	1.78857	0.55154	4	8	4	4.57143	2.82843	4	11	7	10	4.94975	3	3
2.04	3.04	1	2.31458	0.4344	1	2	1	1.45833	0.63557	1	15	14	6.875	8.18691	1	2
1.55	3.44	1.89	2.7075	1.2576	2	6	4	2.9	1.64287	7	16	9	12.8	5.93819	2	3
1.23	1.96	0.73	1.741	0.51619	5	8	3	5.9	2.12132	13	14	1	13.7	0.70711	2	3
1.61	1.9	0.29	1.84957	0.20506	3	3	0	3	0	13	14	1	13.8261	0.70711	2	2
0.82	3.04	2.22	2.21182	0.5206	1	4	3	1.54545	1.13057	1	15	14	5.18182	8.2617	1	2
1.6	3.44	1.84	2.82941	1.16248	2	5	3	3.05882	2.00338	5	16	11	12.8235	6.45253	2	3
1.6	3.44	1.84	2.82941	1.16248	2	5	3	3.05882	2.00338	5	16	11	12.8235	6.45253	2	3
1	2.2	1.2	1.9	0.43774	3	6	3	3.90909	1.83162	2	14	12	11.5455	5.20546	1	3
1.55	3.44	1.89	2.875	0.94829	2	6	4	3.4	2.36352	7	16	9	13.8	4.57881	2	3
1.88	2.01	0.13	1.95583	0.09192	4	4	0	4	0	9	14	5	11.9167	3.53553	2	3
1.88	3.04	1.16	2.46	0.82024	2	4	2	3	1.41421	9	15	6	12	4.24264	2	3
1.96	3.98	2.02	3.17	1.15561	2	5	3	3.16667	1.71888	14	17	3	16	1.80907	2	2
0.98	3.44	2.46	2.48824	1.32523	2	8	6	2.70588	2.40966	1	16	15	11.6471	7.28011	1	3
0.79	3.98	3.19	2.8	1.91849	2	6	4	3	2.03419	1	17	16	11.8	9.41019	1	2
0.79	3.98	3.19	2.805	1.40917	2	6	4	3.28571	2.08514	1	17	16	11.5714	7.82415	1	3
0.98	3.44	2.46	2.56235	1.21896	2	3	1	2.03922	0.24181	1	17	16	12.2745	7.12897	1	2
1.3	3.44	2.14	2.78	1.33147	2	9	7	3.22222	3.1305	4	16	12	12	8.04984	2	3
0.82	3.44	2.62	2.31	1.51708	2	4	2	2.83333	1.14812	1	16	15	10.6667	8.79049	1	2
2.04	2.2	0.16	2.13043	0.11314	1	2	1	1.43478	0.70711	1	13	12	6.21739	8.48528	1	2
0.79	3.16	2.37	2.294	1.42788	3	8	5	4.6	2.75162	1	17	16	12.2	8.55236	1	3
2.28	3.44	1.16	2.95714	0.73786	2	6	4	3.71429	2.61861	10	16	6	14.5714	2.72554	2	3
0.79	3.44	2.65	2.85833	1.27369	2	8	6	3.5	3.04027	1	17	16	13.1667	7.3116	1	3
0.82	3.44	2.62	2.17667	1.61355	2	5	3	3.33333	1.93649	1	16	15	9.11111	9.45604	1	2
1.9	3.44	1.54	2.84571	0.92701	2	6	4	3.14286	1.92725	6	16	10	13.1429	4.81812	2	3
1	3.44	2.44	2.84909	1.13073	2	4	2	2.54545	1.05887	2	16	14	13.9091	5.75774	1	3
1.55	3.44	1.89	2.92111	1.06205	2	5	3	2.88889	1.8303	7	16	9	14.7778	3.95601	2	3
1.3	2.2	0.9	1.84514	0.3646	5	9	4	5.45714	1.72339	4	13	9	11.1143	3.89495	2	3
1.65	1.88	0.23	1.71571	0.16263	4	4	0	4	0	9	12	3	11.1429	2.12132	3	3
1.2	1.81	0.61	1.59214	0.43134	4	8	4	5.42857	2.82843	9	13	4	11.5714	2.82843	2	3
0.98	3.98	3	2.8	1.35533	1	2	1	1.75	0.51355	1	17	16	9	9.21537	1	2
0.89	2.02	1.13	1.56333	0.61198	5	6	1	5.75758	0.53149	2	15	13	9.93939	7.11428	1	3
0.82	2.58	1.76	2.04286	0.95251	3	8	5	3.71429	1.64677	1	16	15	11.9286	8.04068	1	3
1	3.44	2.44	2.644	1.35714	2	4	2	2.6	1.06904	2	16	14	12.8	7.28991	1	2
1.25	3.44	2.19	2.776	1.09802	2	8	6	2.45	1.74331	13	16	3	14.9	1.86034	2	3
0.89	3.44	2.55	2.665	1.2423	2	6	4	3.06667	1.79419	2	16	14	12.2667	6.27967	1	3
1.83	3.44	1.61	2.7468	0.67732	1	4	3	1.76	0.90955	1	16	15	9.52	8.48876	1	3
0.93	3.98	3.05	3.01333	1.95317	2	9	7	3	3.07409	1	17	16	12.2222	9.80816	1	3
1	3.44	2.44	2.73077	0.95602	1	6	5	2.23077	1.66491	1	17	16	9.69231	8.68049	1	3
1.13	3.44	2.31	2.81857	1.19566	2	8	6	3.07143	2.92839	5	16	11	13.2857	5.82448	2	3
0.93	3.44	2.51	2.50529	1.30316	2	4	2	2.41176	0.86136	1	16	15	11.2941	7.7875	1	3
0.89	3.44	2.55	2.602	1.44126	2	6	4	3.46667	2.4432	2	16	14	11.3333	8.00481	1	3
1.83	3.44	1.61	2.9029	0.90007	2	5	3	2.96774	1.59545	8	17	9	14.7742	3.86349	2	3
1	3.44	2.44	2.64571	1.26536	2	4	2	2.85714	1.30931	2	16	14	12	6.74537	1	3

1.36	1.91	0.55	1.664	0.31589	4	9	5	6.2	2.89396	7	13	6	9.4	2.80624	2	3	1	2.8	0.5	58.6934	237	178.307
1.2	2.2	1	1.716	0.56045	5	8	3	6.2	1.83712	9	13	4	10.2	1.83712	2	3	1	2.8	0.5	106.42	151.964	45.544
1.17	2.2	1.03	1.874	0.47088	3	8	5	5.2	2.42384	8	14	6	10.8	3.29773	2	3	1	2.6	0.61237	28.0855	192.217	164.132
1.55	2.66	1.11	2.266	0.49714	4	6	2	4.6	0.94281	7	17	10	14.2	4.30762	2	3	1	2.2	0.4714	54.938	208.98	154.042
1.1	1.96	0.86	1.702	0.60811	5	8	3	5.9	2.12132	3	14	11	10.7	7.77817	2	3	1	2.3	0.70711	118.71	138.905	20.1955
1.25	1.9	0.65	1.616	0.37754	4	8	4	5.8	2.29129	11	15	4	13	2.23607	2	3	1	2.8	0.5	63.546	168.934	105.388
1.14	2.2	1.06	1.912	0.49405	4	8	4	5.6	1.87083	6	14	8	10.4	3.9211	2	3	1	2.6	0.61237	72.64	192.217	119.577
1.12	2.2	1.08	1.864	0.49432	3	8	5	5.2	2.42384	4	14	10	10	4.67707	2	3	1	2.6	0.61237	28.0855	192.217	164.132
0.89	3.44	2.55	2.228	1.38305	2	6	4	4.4	1.87083	2	17	15	10.8	8.99305	1	2	1	1.6	0.61237	15.9994	137.327	121.328
1.1	2.2	1.1	1.904	0.5136	4	8	4	5.6	1.87083	9	14	5	11.4	2.80624	2	3	1	2.6	0.61237	72.64	192.217	119.577
1.3	1.96	0.66	1.808	0.31873	4	9	5	5.4	2.3184	4	14	10	10.4	4.58258	2	3	1	2.6	0.61237	58.6934	232.038	173.345
1	2.28	1.28	1.668	0.71896	4	6	2	5	1.11803	2	13	11	7.4	5.67891	1	3	2	2	1.11803	40.078	195.084	155.006
1.2	3.44	2.24	2.678	1.36466	2	8	6	4.2	3.74166	10	16	6	13.7	3.85141	2	3	1	2.4	0.66667	15.9994	196.967	180.967
1.25	2.55	1.3	2.086	0.59522	2	8	6	3.2	3	13	15	2	13.8	0.93541	2	3	1	2.2	0.5	10.811	168.934	158.123
2	2.58	0.58	2.386	0.3275	3	6	3	3.6	1.60357	12	16	4	15	2.0702	2	3	1	2.2	0.53452	30.9738	200.59	169.616
1.14	2.28	1.14	2.012	0.54786	4	8	4	5.2	1.83712	6	15	9	10.8	4.5	2	3	1	2.6	0.61237	74.9216	144.242	69.3204
1.2	2.28	1.08	1.912	0.4931	3	8	5	5.2	2.42384	9	14	5	11.4	2.69258	2	3	1	2.6	0.61237	28.0855	195.084	166.999
1.23	2.2	0.97	1.886	0.44297	3	8	5	5.2	2.42384	9	14	5	11.8	2.89396	2	3	1	2.6	0.61237	28.0855	192.217	164.132
1.22	2.2	0.98	1.94	0.4588	2	8	6	4.8	3	9	13	4	11.2	2.29129	2	3	1	2.6	0.61237	10.811	192.217	181.406
1.24	1.91	0.67	1.616	0.38831	4	8	4	5.8	2.29129	10	14	4	12.2	2.29129	2	3	1	2.8	0.5	58.6934	167.259	108.566
1.2	3.44	2.24	1.75429	1.02613	2	8	6	6.57143	2.72554	9	16	7	11.7143	4.16619	2	3	1	2.57	0.65465	15.9994	208.98	192.981
1.2	2.55	1.35	2.076	0.61728	2	8	6	3.2	3	10	14	4	12.8	1.83712	2	3	1	2.2	0.5	10.811	157.25	146.439
1.1	2.55	1.45	2.056	0.66203	2	8	6	3.2	3	11	14	3	13	1.36931	2	3	1	2.2	0.5	10.811	158.925	148.114
1.27	2.55	1.28	2.09	0.58646	2	8	6	3.2	3	13	17	4	14.2	1.83712	2	3	1	2.2	0.5	10.811	174.967	164.156
1.2	2.2	1	1.88	0.45689	3	8	5	5.2	2.42384	9	14	5	11.2	2.89396	2	3	1	2.6	0.61237	28.0855	192.217	164.132
0.89	3.44	2.55	2.512	1.5117	2	8	6	3.7	2.87977	2	16	14	10.8	8.4241	1	3	2	2	0.83045	15.9994	138.905	122.906
0.95	2.54	1.59	1.85	0.76596	3	6	3	4.6	1.69558	2	13	11	10	5.12348	1	3	2	2.2	0.93541	26.9815	196.967	169.985
1.2	2.54	1.34	1.888	0.75043	5	8	3	6.6	1.5	10	14	4	11.2	1.83712	2	3	1	2.8	0.5	118.71	196.967	78.2566
1.1	2.55	1.45	2.056	0.66203	2	8	6	3.2	3	3	14	11	11.4	5.27968	2	3	1	2.2	0.5	10.811	138.905	128.094
1.17	2.2	1.03	1.918	0.47941	4	8	4	5.6	1.87083	8	14	6	10.8	3.29773	2	3	1	2.6	0.61237	72.64	192.217	119.577
1.13	2.2	1.07	1.922	0.50302	2	8	6	4.8	3	5	13	8	9.8	3.74166	2	3	1	2.6	0.61237	10.811	192.217	181.406
1.17	1.96	0.79	1.782	0.38352	4	8	4	5.2	1.83712	8	14	6	11.2	3	2	3	1	2.6	0.61237	58.6934	150.36	91.6666
1.22	2.28	1.06	1.916	0.4841	3	6	3	4.6	1.69558	3	14	11	10.2	5.02494	2	3	1	2.6	0.61237	28.0855	195.084	166.999
1.22	2.54	1.32	1.904	0.67308	3	8	5	5.2	2.42384	11	13	2	12	1.11803	2	3	1	2.6	0.61237	26.9815	196.967	169.985
1.1	2.2	1.1	1.92	0.51931	5	8	3	5.6	1.5	3	15	12	10.6	5.51135	2	3	1	2.6	0.61237	106.42	138.905	32.4855
1.38	2.2	0.82	1.788	0.45842	5	9	4	7	2.09165	6	13	7	8.6	3.22102	2	3	1	2.8	0.5	114.818	238.029	123.211
1.5	1.57	0.07	1.528	0.0495	2	6	4	4.4	2.82843	2	5	3	3.8	2.12132	1	3	2	2.2	1.41421	9.01218	180.948	171.936
1.23	1.9	0.67	1.608	0.38966	4	8	4	5.8	2.29129	11	13	2	12.2	1.22474	2	3	1	2.8	0.5	63.546	164.93	101.384
1.5	2.04	0.54	1.748	0.30684	2	6	4	4	2.23607	5	13	8	8.4	4.71699	2	3	1	2.6	0.61237	10.811	180.948	170.137
1.13	1.96	0.83	1.77	0.4014	4	8	4	5.2	1.83712	5	14	9	11	4.10792	2	3	1	2.6	0.61237	63.546	140.908	77.3617
1.22	2.04	0.82	1.824	0.38443	2	8	6	4	2.73861	10	13	3	11.6	1.69558	2	3	1	2.6	0.61237	10.811	162.5	151.689
1.24	2.28	1.04	1.92	0.47513	3	8	5	5.2	2.42384	10	14	4	12.4	2.44949	2	3	1	2.6	0.61237	28.0855	195.084	166.999
1.1	2.54	1.44	1.88	0.7127	3	8	5	5.2	2.42384	3	13	10	10.2	4.63681	2	3	1	2.6	0.61237	26.9815	196.967	169.985
1.36	1.9	0.54	1.576	0.38184	3	9	6	6.6	4.24264	7	14	7	9.8	4.94975	2	3	1	2.6	0.70711	28.0855	237	208.915
1.12	2.2	1.08	1.748	0.51268	3	8	5	4.8	2.29129	4	13	9	10	4.10792	2	3	1	2.6	0.61237	26.9815	140.116	113.134
1.1	2.28	1.18	1.892	0.53866	3	8	5	5.2	2.42384	3	14	11	10.2	5.02494	2	3	1	2.6	0.61237	28.0855	195.084	166.999
0.98	1.9	0.92	1.412	0.50339	2	8	6	4.8	3.29773	1	14	13	8.2	6.34429	1	3	2	2.2	0.93541	6.941	144.242	137.301
1.12	2.54	1.42	1.884	0.70589	3	8	5	5.2	2.42384	4	13	9	10.4	4.15331	2	3	1	2.6	0.61237	26.9815	196.967	169.985
1.22	1.9	0.68	1.604	0.39573	4	5	1	4.6	0.61237	3	13	10	8.2	5.38516	2	3	1	2.8	0.5	63.546	114.818	51.272
1.17	2.54	1.37	1.894	0.68923	3	8	5	5.2	2.42384	8	13	5	11.2	2.29129	2	3	1	2.6	0.61237	26.9815	196.967	169.985
1.27	1.9	0.63	1.624	0.36543	4	8	4	5.8	2.29129	11	17	6	13.8	3.39116	2	3	1	2.8	0.5	63.546	174.967	111.421
1.38	2.28	0.9	1.82	0.50374	5	9	4	7	2.09165	6	13	7	9	3.3541	2	3	1	2.8	0.5	114.818	238.029	123.211
1.27	1.91	0.64	1.628	0.37017	4	8	4	5.8	2.29129	10	17	7	13.4	3.9211	2	3	1	2.8	0.5	58.6934	174.967	116.274
1.13	2.2	1.07	1.91	0.49894	4	8	4	5.6	1.87083	5	14	9	10.2	4.28661	2	3	1	2.6	0.61237	72.64	192.217	119.577
1.28	1.9	0.62	1.528	0.43841	3	9	6	6.6	4.24264	8	14	6	10.4	4.24264	2	3	1	2.6	0.70711	28.0855	244	215.915
1.38	2.2	0.82	1.96	0.37774	4	9	5	5.8	2.29129	6	14	8	10.4	3.9211	2	3	1	2.6	0.61237	72.64	238.029	165.389
1.17	1.91	0.74	1.588	0.43073	4	8	4	5.8	2.29129	8	13	5	9.8	2.29129	2	3	1	2.8	0.5	58.6934	150.36	91.6666
1.22	1.9	0.68	1.604	0.39573	4	8	4	5.8	2.29129	11	13	2	11.8	0.93541	2	3	1	2.8	0.5	63.546	162.5	98.954
0.93	1.96	1.03	1.367	0.63605	2	5	3	3.3	1.86159	1	14	13	6.2	8.36248	1	2	1	1.4	0.64327	6.941	118.71	111.769
1.12	1.96	0.84	1.772	0.40846	4	8	4	5.2	1.83712	4	14	10	10.4	4.58258	2	3	1	2.6	0.61237	58.6934	140.116	81.4226
1.14	2.28	1.14	2.016	0.54981	3	8	5	4.8	2.29129	6	15	9	10.8	4.5	2	3	1	2.6	0.61237	30.9738	144.242	113.268
1.14	2.2	1.06	1.868	0.48492	3	8	5	5.2	2.42384	6	14	8	10.4	3.9211	2	3	1	2.6	0.61237	28.0855	192.217	164.132
1.13	1.9	0.77	1.568	0.45037	4	8	4	5.8	2.29129	5	13	8	9	4.1833	2	3	1	2.8	0.5	63.546	140.908	77.3617
1.2	2.54	1.34	1.9	0.67948	3	8	5	5.2	2.42384	10	13	3	11.6	1.5	2	3	1	2.6	0.61237	26.9815	196.967	169.985
1.23	2.54	1.31	1.864	0.73419	5	8	3	6.6	1.5	11	13	2	12.2	1.22474	2	3	1	2.8	0.5	114.818	196.967	82.1486
1.13	1.9	0.77	1.438	0.54447	3	8	5	6	3.53553	5	14	9	8.6	6.36396	2	3	1	2.6	0.70711	28.0855	140.908	112.822
0.82	3.98	3.16	2.806	1.90769	2	4	2	2.6	1.05045	1	17	16	11.8	9.41019	1	2	1	1.7	0.60172	18.9984	39.0983	20.0999
1.1	1.96	0.86	1.768	0.41843	4	8	4	5.2	1.83712	3	14	11	10.2	5.02494	2	3	1	2.6	0.61237	58.6934	138.905	80.2121
1.12	2.28	1.16	1.92	0.53104	5	8	3	5.6	1.5	4	14	10	10	4.67707	2	3	1	2.6	0.61237	102.906	140.116	37.2105
1.12	3.44	2.32	2.348	0.89995	2	8	6	4.4	2.64575	4	16	12	13.4	5.55778	2	3	1	2.2	0.4714	15.9994	208.98	192.981
1.13	2.54	1.41	1.886	0.70252	3	8	5	5.2	2.42384	5	13	8	10.6	3.67423	2	3	1	2.6	0.61237	26.9815	196.967	169.985
1.22	2.04	0.82	1.824	0.38443	2	5	3	3.4	1.5	3	13	10	9.8	4.56892	2	3	1	2.6	0.61237	10.811	88.9059	78.0949
1.1	2.01	0.91	1.486	0.43421	3	8	5	5.2	2.89396	3	14	11	9.2	6.34429	2	3	1	2.4	0.61237	26.9815	138.905	111.924
1.24	1.9	0.66	1.612	0.3836	4	8	4	5.8	2.29129	11	14	3	12.6	1.69558	2	3	1	2.8	0.5	63.546	167.259	103.713
1.22	2.2	0.98	1.724	0.54895	5	5	0	5	0	3	13	10	7.8	5.08675	2	3	1	2.8	0.5	88.9059	114.818	25.9121
1.1	1.91	0.81	1.56	0.47322	4	8	4	5.8	2.29129	10	13	3	11	1.36931	2	3	1	2.8	0.5	58.6934	158.925	100.232
1.25	1.91	0.66	1.62	0.38226	4	8	4	5.8	2.29129	10	15	5	12.6	2.80624	2	3	1	2.8	0.5	58.6934	168.934	110.241
1.14	1.91	0.77	1.576	0.44893	4	8	4	5.8	2.29129	6	13	7	9	3.3541	2	3	1	2.8	0.5	58.6934	144.242	85.5486
1.13	1.91	0.78	1.572	0.455	4	8	4	5.8	2.29129	5	13	8	8.6	3.9211	2	3	1	2.8	0.5	58.6934	140.908	82.2142
1.38	2.28	0.9	1.992	0.4112	4	9	5	5.8	2.29129	6	14	8	10.8	3.74166	2	3	1	2.6	0.61237	72.64	238.029	165.389
1.1	2.2	1.1	1.904	0.5136	4	8	4	5.6	1.87083	3	14	11	9.8	5.08675	2	3	1	2.6	0.61237	72.64	192.217	119.577
0.82	3.98	3.16	2.781	1.93703	2	4	2	2.7	1.18176	1	17	16	10.8	9.99655	1	3	2	1.8	0.78784	18.9984	44.9559	25.9575
1.23	1.91	0.68	1.612	0.39437	4	8	4	5.8	2.29129	10	13	3	11.8	1.83712	2	3	1	2.8	0.5	58.6934	164.93	106.237
1.1	2.28	1.18	2.008	0.56973	3	8	5	4.8	2.29129	3	15	12	10.2	5.61249	2	3	1	2.6	0.61237	30.9738	138.905	107.932
1.22	2.54	1.32	1.86	0.73959	5	6	1	5.4	0.61237	3	13	10	8.2	5.38516	2	3	1	2.8	0.5	88.9059	196.967	108.061
2.2	3.98	1.78	3.624	1.25865	2	5	3	2.6	2.12132	10	17	7	15.6	4.94975	2	3	1	2.2	0.70711	18.9984	106.42	87.4216
0.82	3.98	3.16	2.828	1.88575	2	4	2	2.7	1.18176	1	17	16	11.3	9.5059	1	3	2	1.8	0.78784	18.9984	55.845	36.8466
1.14	2.28	1.14	1.9	0.52034	3	8	5	5.2	2.42384	6	14	8	10.8	3.74166	2	3	1	2.6	0.61237	28.0855	195.084	166.999
1.3	2.2	0.9	1.9	0.41079	3	9	6	5.4	2.80624	4	14	10	10	4.67707	2	3	1	2.6	0.61237	28.0855	232.038	203.953
1.3	2.2	0.9	1.944	0.41628	4	9	5	5.8	2.29129	4	14	10	10	4.67707	2	3	1	2.6	0.61237	72.64	232.038	159.398
0.98	3.44	2.46	2.792	1.27622	2	5	3	2.6	1.60357	1	17	16	13.2	8.16788	1	2	1	1.8	0.53452	6.941	126.904	119.963
1.1	1.9	0.8	1.556	0.46861	4	8	4	5.8	2.29129	11	13	2	11.4	1	2	3	1	2.8	0.5	63.546	158.925	95.3794
1.6	3.44	1.84	2.604	0.78292	2	5	3	4.4	1.60357	5	17	12	14.4	6.30193	2	3	1	2.2	0.53452	15.9994	126.904	110.905
1.13	2.28	1.15	2.014	0.55479	3	8	5	4.8	2.29129	5	15	10	10.6	4.84768	2	3	1	2.6	0.61237	30.9738	140.908	109.934
1.23	1.91	0.68	1.502	0.48083	4	8	4	6.4	2.82843	10	13	3	11.8	2.12132	3	3	0	3	0	58.6934	164.93	106.237
1.22	1.91	0.69	1.608	0.40042	4	5	1	4.6	0.61237	3	13	10	7.8	5.08675	2	3	1	2.8	0.5	58.6934	114.818	56.1246
0.98	2.58	1.6	2.108	0.81388	2	4	2	3.2	0.8165	1	16	15	10.9	8.57321	1	3	2	2.2	0.8165	6.941	47.867	40.926
2.2	3.44	1.24	3.192	0.87681	2	6	4	2.8	2.82843	8	16	8	14.4	5.65685	2	3	1	2.2	0.70711	15.9994	190.23	174.231
0.89	3.44	2.55	2.664	1.34787	2	6	4	3.6	2.57307	2	16	14	12.4	7.33908	1	3	2	1.9	0.70711	15.9994	208.98	192.981
1.38	2.96	1.58	2.644	1.11723	4	9	5	5	3.53553	6	17	11	14.8	7.77817	2	3	1	2.2	0.70711	79.904	238.029	158.125
2.55	3.98	1.43	3.366	0.68573	2	3	1	2.4	0.61237	14	17	3	16.4	1.5	2	2	0	2	0	12.0107	35.453	23.4423
1.24	2.54	1.3	1.868	0.7288	5	8	3	6.6	1.5	11	14	3	12.6	1.69558	2	3	1	2.8	0.5	114.818	196.967	82.1486
0.98	2.05	1.07	1.44	0.62685	2	8	6	4.4	2.80624	1	15	14	7.6	7.88987	1	3	2	1.8	0.93541	6.941	144.242	137.301
0.82	3.98	3.16	2.932	1.77487	2	4	2	2.8	1.30931	1	17	16	12.6	8.34951	1	3	2	2	0.84515	18.9984	63.546	44.5476
0.98	2.05	1.07	1.436	0.62991	2	8	6	4.4	2.80624	1	15	14	7.2	8.07775	1	3	2	1.8	0.93541	6.941	140.116	133.175
1.2	2.28	1.08	1.956	0.49602	4	8	4	5.6	1.87083	9	14	5	11.4	2.69258	2	3	1	2.6	0.61237	72.64	195.084	122.444
0.82	3.98	3.16	2.862	1.85558	2	4	2	2.8	1.30931	1	17	16	11.8	8.88015	1	3	2	2	0.84515	18.9984	54.938	35.9396
1.2	1.93	0.73	1.492	0.51619	5	8	3	6.8	2.12132	9	11	2	9.8	1.41421	3	3	0	3	0	107.868	151.964	44.0958
0.79	3.98	3.19	2.794	1.92505	2	6	4	3.1	2.07198	1	17	16	11.2	9.57367	1	3	2	1.8	0.78784	18.9984	132.905	113.907
1.22	1.91	0.69	1.608	0.40042	4	8	4	5.8	2.29129	10	13	3	11.4	1.5	2	3	1	2.8	0.5	58.6934	162.5	103.807
1.3	1.9	0.6	1.54	0.42426	3	9	6	6.6	4.24264	4	14	10	8	7.07107	2	3	1	2.6	0.70711	28.0855	232.038	203.953
0.89	3.44	2.55	2.613	1.41674	2	9	7	3.9	3.24037	2	16	14	12	7.61124	1	3	2	1.9	0.70711	15.9994	238.029	222.03
1.12	2.05	0.93	1.808	0.43706	4	8	4	5.2	1.83712	4	15	11	10.8	5.08675	2	3	1	2.6	0.61237	58.6934	140.116	81.4226
1.14	1.9	0.76	1.572	0.4443	4	8	4	5.8	2.29129	6	13	7	9.4	3.58818	2	3	1	2.8	0.5	63.546	144.242	80.696
1.17	2.04	0.87	1.814	0.40901	2	8	6	4	2.73861	8	13	5	10.8	2.42384	2	3	1	2.6	0.61237	10.811	150.36	139.549
1.1	1.91	0.81	1.424	0.57276	4	8	4	6.4	2.82843	10	11	1	10.6	0.70711	3	3	0	3	0	58.6934	158.925	100.232
0.82	3.98	3.16	2.769	1.95272	2	8	6	3.3	2.563	1	17	16	11.9	9.44038	1	3	2	1.8	0.78784	18.9984	167.259	148.261
0.98	2.55	1.57	2.09	0.79456	2	4	2	3.8	0.8165	1	16	15	10.9	8.57321	1	3	2	2.2	0.8165	6.941	78.96	72.019
0.79	3.98	3.19	2.794	1.93222	2	6	4	3.2	2.1009	1	17	16	11.1	9.65705	1	3	2	1.8	0.78784	18.9984	132.905	113.907
2.2	3.98	1.78	3.624	1.25865	2	6	4	2.8	2.82843	9	17	8	15.4	5.65685	2	3	1	2.2	0.70711	18.9984	192.217	173.219
1.83	2.16	0.33	2.046	0.15075	2	5	3	3.6	1.69558	6	13	7	9.2	3.98434	2	3	1	2.6	0.61237	10.811	95.94	85.129
0.98	2.05	1.07	1.438	0.62836	2	8	6	4.4	2.80624	1	15	14	7.4	7.96869	1	3	2	1.8	0.93541	6.941	140.908	133.967
1.5	2.1	0.6	1.98	0.42426	5	6	1	5.2	0.70711	5	16	11	13.8	7.77817	2	3	1	2.2	0.70711	127.6	180.948	53.3479
1.22	2.28	1.06	1.96	0.48651	4	8	4	5.6	1.87083	10	14	4	12	2.23607	2	3	1	2.6	0.61237	72.64	195.084	122.444
1.54	2.66	1.12	2.436	0.79196	4	5	1	4.8	0.70711	4	17	13	14.4	9.19239	2	3	1	2.2	0.70711	47.867	126.904	79.0375
1	3.44	2.44	2.574	1.16361	2	4	2	3	1.05409	2	17	15	11.8	6.9282	1	3	2	2	0.74536	15.9994	55.845	39.8456
1.9	2.28	0.38	2.052	0.2687	3	6	3	4.2	2.12132	10	14	4	12.4	2.82843	2	3	1	2.4	0.70711	28.0855	195.084	166.999
1.1	1.9	0.8	1.522	0.45058	3	8	5	5	3.06186	13	16	3	14.6	1.5	2	3	1	2.4	0.61237	26.9815	173.04	146.058
0.93	3.98	3.05	2.828	1.93665	2	3	1	2.4	0.66667	1	17	16	11.8	9.75249	1	2	1	1.7	0.62361	18.9984	26.9815	7.98314
1.61	1.9	0.29	1.842	0.145	3	6	3	4.6	1.5	7	13	6	9.8	3	2	3	1	2.8	0.5	26.9815	186.207	159.225
1.81	2.55	0.74	2.164	0.4156	4	5	1	4.2	0.5	13	16	3	14.8	1.83712	2	2	0	2	0	69.723	127.6	57.877
2.01	2.28	0.27	2.172	0.19092	4	6	2	5.2	1.41421	10	14	4	11.6	2.82843	2	3	1	2.6	0.70711	72.64	195.084	122.444
1.55	2.18	0.63	1.802	0.44548	4	4	0	4	0	7	15	8	10.2	5.65685	2	3	1	2.6	0.70711	54.938	74.9216	19.9836
1.63	2.55	0.92	2.324	0.41089	3	5	2	3.9	0.70711	5	16	11	14.2	4.46442	2	3	1	2.2	0.52523	30.9738	107.868	76.8944
1.12	2.2	1.08	1.888	0.4984	5	8	3	6	1.36931	4	14	10	10	4.67707	2	3	1	2.6	0.61237	118.71	192.217	73.507
1.1	1.9	0.8	1.58	0.56569	3	8	5	5	3.53553	11	14	3	12.8	2.12132	2	3	1	2.4	0.70711	28.0855	158.925	130.84
0.82	3.98	3.16	2.852	1.30882	2	4	2	2.6	0.94281	1	17	16	13.2	7.20339	1	2	1	1.8	0.4714	15.9994	39.0983	23.0989
0.79	3.98	3.19	2.822	1.8968	2	6	4	3.1	2.07198	1	17	16	11.3	9.5059	1	3	2	1.8	0.78784	18.9984	132.905	113.907
0.89	3.44	2.55	2.582	1.46048	2	8	6	3.8	2.98271	2	16	14	11.4	7.88276	1	3	2	2	0.83045	15.9994	195.084	179.085
0.82	2.66	1.84	2.01	1.07922	4	8	4	5.4	1.81265	1	17	16	13.4	8.34951	1	3	2	2	0.84515	39.0983	168.934	129.836
0.79	3.16	2.37	2.562	1.22668	3	6	3	4.2	1.96396	1	17	16	12.6	8.34951	1	3	2	2	0.84515	35.453	196.967	161.514
0.79	2.2	1.41	1.792	0.74467	1	6	5	2.2	2.59119	1	2	1	1.2	0.53452	1	1	0	1	0	1.00794	132.905	131.898
0.82	2.2	1.38	1.743	0.7831	1	4	3	1.9	1.6028	1	13	12	2.2	4.72703	1	2	1	1.1	0.39392	1.00794	39.0983	38.0904
1.96	2.2	0.24	2.152	0.16971	1	5	4	1.8	2.82843	1	14	13	3.6	9.19239	1	2	1	1.2	0.70711	1.00794	118.71	117.702
2.02	3.44	1.42	2.872	1.00409	2	6	4	3.6	2.82843	15	16	1	15.6	0.70711	2	2	0	2	0	15.9994	208.98	192.981
2.28	3.98	1.7	3.64	1.20208	2	6	4	2.8	2.82843	10	17	7	15.6	4.94975	2	3	1	2.2	0.70711	18.9984	195.084	176.086
1.1	2.55	1.45	1.97	0.94925	4	8	4	5.6	2.61861	3	16	13	13.4	6.94879	2	3	1	2.4	0.65465	78.96	173.04	94.08
0.93	3.98	3.05	2.912	1.77718	2	5	3	2.8	1.55839	1	17	16	12.8	8.29802	1	3	2	2	0.84515	18.9984	112.411	93.4126
0.82	3.98	3.16	2.768	1.95407	2	8	6	3.3	2.563	1	17	16	11.8	9.41019	1	3	2	1.8	0.78784	18.9984	164.93	145.932
0.82	3.98	3.16	2.768	1.95407	2	8	6	3.3	2.563	1	17	16	11.8	9.41019	1	3	2	1.8	0.78784	18.9984	164.93	145.932
2.2	3.98	1.78	3.624	1.25865	2	5	3	2.6	2.12132	8	17	9	15.2	6.36396	2	3	1	2.2	0.70711	18.9984	101.07	82.0716
1.91	2.58	0.67	2.178	0.47376	3	4	1	3.6	0.70711	10	16	6	12.4	4.24264	2	3	1	2.6	0.70711	32.065	58.6934	26.6284
1.69	3.44	1.75	2.594	0.89427	1	5	4	2.2	1.83712	1	16	15	9.2	8.56592	1	3	2	1.8	0.93541	1.00794	112.411	111.403
0.82	2.18	1.36	1.636	0.96167	4	5	1	4.4	0.70711	1	15	14	9.4	9.89949	1	2	1	1.6	0.70711	74.9216	85.4678	10.5462
1.2	1.81	0.61	1.444	0.43134	4	8	4	6.4	2.82843	9	13	4	10.6	2.82843	2	3	1	2.6	0.70711	69.723	151.964	82.241
0.82	3.98	3.16	2.795	1.99682	2	4	2	2.7	1.22474	1	17	16	11.8	9.75249	1	2	1	1.7	0.62361	18.9984	39.0983	20.0999
1.31	1.81	0.5	1.53	0.28668	3	4	1	3.8	0.5	2	13	11	6.8	6.34429	1	3	2	2.2	0.93541	24.305	69.723	45.418
1.22	2.2	0.98	2.07	0.42799	2	5	3	4.4	1.7693	3	13	10	9.9	3.81359	2	3	1	2.8	0.58977	10.811	106.42	95.609
1.12	1.91	0.79	1.568	0.46107	4	8	4	5.8	2.29129	4	13	9	8.2	4.5	2	3	1	2.8	0.5	58.6934	140.116	81.4226
0.79	2.18	1.39	1.624	0.98288	4	6	2	4.8	1.41421	1	15	14	9.4	9.89949	1	2	1	1.6	0.70711	74.9216	132.905	57.9839
1.12	2.2	1.08	1.684	0.60671	5	8	3	6.2	1.83712	4	13	9	8.2	4.5	2	3	1	2.8	0.5	106.42	140.116	33.696
1.66	2.1	0.44	1.972	0.23275	3	5	2	4.4	1.06904	6	16	10	13.6	5.18239	2	3	1	2.2	0.53452	28.0855	127.6	99.5145

1.24	2.2	0.96	1.624	0.67882	5	8	3	6.8	2.12132	8	14	6	11.6	4.24264	3	3	[illegible]
1.54	3.44	1.9	2.738	1.15543	2	4	2	2.8	1.30931	4	16	12	12	6.76123	2	3	[illegible]
0.95	3.44	2.49	2.514	1.52272	2	6	4	3.4	2.32993	2	16	14	10.8	8.55236	1	3	[illegible]
2.02	3.44	1.42	2.872	1.00409	2	6	4	3.6	2.82843	15	16	1	15.6	0.70711	2	2	[illegible]
1.1	3.44	2.34	2.608	1.3793	2	8	6	4	3.38062	3	16	13	12.8	6.72947	2	3	[illegible]
0.79	2.96	2.17	2.178	1.29268	4	8	4	5.2	2.13809	1	17	16	12.8	8.29802	1	3	[illegible]
1.1	1.9	0.8	1.462	0.45719	3	8	5	5	3.06186	2	16	14	12.4	6.59545	1	3	[illegible]
2.2	2.55	0.35	2.48	0.24749	2	6	4	2.8	2.82843	9	14	5	13	3.53553	2	3	[illegible]
1.55	3.44	1.89	3.006	0.98356	2	4	2	2.6	1.06904	7	17	10	14.4	4.97135	2	3	[illegible]
1.6	2.1	0.5	1.9	0.35355	5	5	0	5	0	5	16	11	11.6	7.77817	2	3	[illegible]
0.79	2.96	2.17	1.913	1.33232	2	6	4	4.2	1.778	1	17	16	9	10.16	1	2	[illegible]
1.62	2.55	0.93	2.262	0.50366	2	6	4	4	1.69031	13	16	3	14.8	1.96396	2	2	[illegible]
0.95	2.2	1.25	1.878	0.58866	5	6	1	5.4	0.61237	2	15	13	10.4	5.94769	1	3	[illegible]
2.2	2.55	0.35	2.48	0.24749	2	6	4	2.8	2.82843	9	14	5	13	3.53553	2	3	[illegible]
2.01	2.58	0.57	2.39	0.32116	3	5	2	3.6	1.06904	10	16	6	14.4	3.11677	2	3	[illegible]
1.12	2.2	1.08	1.894	0.52294	5	8	3	5.6	1.60357	4	15	11	11.8	5.81869	2	3	[illegible]
[illegible]	[illegible]	[illegible]	[illegible]	[illegible]	[illegible]	[illegible]	[illegible]	[illegible]	[illegible]	[illegible]	[illegible]	[illegible]	[illegible]	[illegible]	[illegible]	[illegible]	[illegible]

0.82	3.16	2.34	2.22714	1.43894	3	4	1	3.28571	0.59761	1	17	16	10.2857	10.2644	1	2	1	1.57	0.65465	24.305	39.0983	14.7933	34.9019	6.09772	1	2.2	1.2	[illegible]
1.17	1.78	0.61	1.3225	0.43134	5	8	3	7.25	2.12132	8	13	5	9.25	3.53553	2	3	1	2.75	0.70711	114.818	150.36	35.542	141.475	25.132	1.55	1.85	0.3	[illegible]
0.79	3.98	3.19	2.775	1.94754	2	6	4	3.1	2.07198	1	17	16	10.8	9.99655	1	3	2	1.8	0.78784	18.9984	132.905	113.907	44.7747	58.7149	0.5	2.6	2.1	[illegible]
1.3	1.61	0.31	1.50667	0.2192	3	9	6	5	4.24264	4	13	9	10	6.36396	2	3	1	2.33	0.70711	26.9815	232.038	205.057	95.3337	144.997	1.25	1.8	0.55	[illegible]
1.38	2.1	0.72	1.84333	0.40204	5	9	4	6.33333	2.3094	6	16	10	12.3333	5.50757	2	3	1	2.33	0.57735	121.76	238.029	116.269	162.463	65.5071	1.4	1.75	0.35	[illegible]
2.55	3.04	0.49	2.83	0.34648	2	2	0	2	0	14	15	1	14.5714	0.70711	2	2	0	2	0	12.0107	14.0067	1.996	13.1513	1.41139	0.65	0.7	0.05	[illegible]
1.96	2.18	0.22	2.05429	0.15556	4	5	1	4.57143	0.70711	14	15	1	14.4286	0.70711	2	2	0	2	0	74.9216	118.71	43.7884	99.9435	30.9631	1.15	1.45	0.3	[illegible]
1.23	2.1	0.87	1.81	0.61518	5	8	3	6	2.12132	13	16	3	15	2.12132	2	3	1	2.33	0.70711	127.6	164.93	37.3303	140.043	26.3965	1.4	1.75	0.35	[illegible]
2.1	3.44	1.34	2.99333	0.94752	2	5	3	3	2.12132	16	16	0	16	0	2	2	0	2	0	15.9994	127.6	111.601	53.1996	78.9135	0.6	1.4	0.8	[illegible]
0.93	3.44	2.51	2.975	1.30143	2	3	1	2.33333	0.66667	1	17	16	13.6667	8.02773	1	2	1	1.83	0.52705	15.9994	35.453	19.4536	20.4067	10.1778	0.6	1.8	1.2	[illegible]
1.22	1.91	0.69	1.795	0.4879	4	5	1	4.16667	0.70711	3	10	7	8.83333	4.94975	3	3	0	3	0	58.6934	88.9059	30.2125	63.7288	21.3634	1.35	1.8	0.45	[illegible]
1.27	1.9	0.63	1.585	0.44548	3	8	5	5.5	3.53553	14	17	3	15.5	2.12132	2	3	1	2.5	0.70711	28.0855	174.967	146.882	101.526	103.861	1.1	1.75	0.65	[illegible]
1.1	2.2	1.1	1.972	0.54511	4	8	4	5.2	1.83712	3	15	12	9.8	5.77711	2	3	1	2.6	0.61237	74.9216	138.905	63.9839	98.1777	29.3533	1.15	1.95	0.8	[illegible]
1.13	1.91	0.78	1.52	0.55154	4	8	4	6	2.82843	5	10	5	7.5	3.53553	3	3	0	3	0	58.6934	140.908	82.2142	99.8005	58.1343	1.35	1.85	0.5	[illegible]
0.95	3.04	2.09	2.09	1.21362	2	5	3	3.5	1.89737	2	15	13	9	7.64199	1	3	2	2	0.89443	14.0067	91.224	77.2173	51.7144	47.724	0.65	2	1.35	[illegible]
1.1	2.01	0.91	1.788	0.43362	4	8	4	4.8	2	10	14	4	11.8	2.29129	2	3	1	2.6	0.61237	58.6934	158.925	100.232	84.3184	47.2766	1.25	1.75	0.5	[illegible]
1.22	2.28	1.06	1.916	0.4841	3	8	5	4.8	2.29129	9	14	5	11.6	2.80624	2	3	1	2.6	0.61237	28.0855	162.5	134.415	84.8964	64.0457	1.1	1.75	0.65	[illegible]
1.3	2.2	0.9	1.9	0.41079	3	9	6	5.4	2.80624	4	14	10	9.6	4.84768	2	3	1	2.6	0.61237	28.0855	232.038	203.953	133.734	109.502	1.1	1.8	0.7	[illegible]
0.95	2.01	1.06	1.67	0.48425	4	5	1	4.6	0.61237	2	14	12	10.8	5.61249	1	3	2	2.2	0.93541	72.64	112.411	39.771	91.5444	22.3676	1.25	2	0.75	[illegible]
1.63	2.54	0.91	2.358	0.64347	4	6	2	5.6	1.41421	5	11	6	9.8	4.24264	3	3	0	3	0	50.9415	196.967	146.025	167.762	103.255	1.35	1.35	0	[illegible]
1.27	2.28	1.01	1.926	0.46176	3	8	5	4.8	2.29129	9	17	8	12.6	3.9211	2	3	1	2.6	0.61237	28.0855	174.967	146.882	87.3898	68.8868	1.1	1.75	0.65	[illegible]
1.55	1.96	0.41	1.85167	0.19329	4	5	1	4.16667	0.52705	7	14	7	10.8333	2.87711	2	3	1	2.83	0.52705	54.938	118.71	63.772	71.3053	30.3091	1.35	1.45	0.1	[illegible]
0.93	2.05	1.12	1.51	0.56107	3	5	2	4	1	1	15	14	7.66667	7.02377	1	3	2	2	1	22.9898	121.76	98.7702	66.5626	50.4008	1.4	1.8	0.4	[illegible]
1.24	2.28	1.04	1.88833	0.59902	4	8	4	5.83333	2.01133	9	14	5	11.5	3.19801	2	3	1	2.83	0.47673	72.64	167.259	94.619	119.312	45.4781	1.25	1.75	0.5	[illegible]
1.12	2.28	1.16	2.08667	0.82024	6	8	2	6.33333	1.41421	4	10	6	9	4.24264	3	3	0	3	0	140.116	195.084	54.968	185.923	38.8682	1.35	1.85	0.5	[illegible]
1.12	2.2	1.08	1.725	0.48344	3	8	5	4.5	2.30612	4	13	9	10.5	4.09545	2	3	1	2.5	0.6396	26.9815	140.116	113.134	72.3168	59.7464	1.25	1.85	0.6	[illegible]
1.2	2.2	1	1.88	0.45689	3	8	5	4.8	2.29129	10	14	4	11.6	2.44949	2	3	1	2.6	0.61237	28.0855	157.25	129.165	85.2522	62.7891	1.1	1.8	0.7	[illegible]
0.98	2.54	1.56	1.6975	0.6937	2	6	4	4	1.82574	1	14	13	7	6.48074	1	3	2	1.75	0.95743	6.941	196.967	190.026	86.7306	88.3893	1.35	1.5	0.15	[illegible]
1.63	2.18	0.55	1.905	0.38891	4	4	0	4	0	5	15	10	10	7.07107	2	3	1	2.5	0.70711	50.9415	74.9216	23.9801	62.9316	16.9565	1.15	1.35	0.2	[illegible]
1.69	2.19	0.5	2.02	0.2583	3	5	2	3.75	1.04881	12	15	3	14	1.54919	2	3	1	2.25	0.54772	30.9738	112.411	81.4372	61.7496	42.7995	1	1.55	0.55	[illegible]
1.81	2.16	0.35	2.0725	0.24749	4	5	1	4.75	0.70711	6	13	7	7.75	4.94975	2	3	1	2.75	0.70711	69.723	95.94	26.217	89.3858	18.5382	1.3	1.45	0.15	[illegible]
1.12	2.58	1.46	2.09333	1.03238	3	8	5	4.66667	3.53553	4	16	12	12	8.48528	2	3	1	2.33	0.70711	32.065	140.116	108.051	68.082	76.4036	1	1.85	0.85	[illegible]
1.24	1.96	0.72	1.744	0.50912	5	8	3	5.9	2.12132	14	14	0	14	0	2	3	1	2.3	0.70711	118.71	167.259	48.549	133.275	34.3293	1.45	1.75	0.3	[illegible]
0.93	2.58	1.65	1.48	1.16673	3	3	0	3	0	1	16	15	6	10.6066	1	2	1	1.33	0.70711	22.9898	32.065	9.07523	26.0148	6.41716	1	1.8	0.8	[illegible]
2.19	2.19	0	2.19	0	3	3	0	3	0	15	15	0	15	0	2	2	0	2	0	30.9738	30.9738	0	30.9738	0	1	1	0	[illegible]
1.69	2.96	1.27	2.53667	0.89803	4	5	1	4.33333	0.70711	12	17	5	15.3333	3.53553	2	3	1	2.33	0.70711	79.904	112.411	32.507	90.7397	22.9859	1.15	1.55	0.4	[illegible]
0.95	3.04	2.09	1.65667	1.1981	2	5	3	3	1.73205	1	15	14	6	7.81025	1	2	1	1.33	0.57735	6.941	87.62	80.679	36.1892	44.6802	0.65	2	1.35	[illegible]
1.22	1.81	0.59	1.56	0.30512	4	5	1	4.33333	0.57735	3	13	10	9.33333	5.50757	2	3	1	2.67	0.57735	65.409	88.9059	23.4968	74.6793	12.508	1.3	1.8	0.5	[illegible]
1.25	2.01	0.76	1.6	0.38354	4	8	4	5.33333	2.3094	4	15	11	11	6.08276	2	3	1	2.67	0.57735	47.867	168.934	121.067	96.4804	63.9577	1.25	1.75	0.5	[illegible]
1.2	2.55	1.35	1.94167	0.46612	2	8	6	3.66667	2.51151	10	14	4	11.6667	2	2	3	1	2.5	0.58835	10.811	157.25	146.439	51.3783	61.0619	0.7	1.8	1.1	[illegible]
2.2	2.58	0.38	2.34571	0.19508	3	6	3	4.71429	1.43614	10	16	6	12.8571	3.20156	2	3	1	2.43	0.61237	32.065	207.2	175.135	113.97	82.3105	1	1.8	0.8	[illegible]
0.95	1.78	0.83	1.1575	0.5869	5	5	0	5	0	2	13	11	4.75	7.77817	1	2	1	1.25	0.70711	87.62	114.818	27.198	94.4195	19.2319	1.55	2	0.45	[illegible]
1.9	3.44	1.54	2.765	0.87056	2	5	3	3.25	1.64317	9	16	7	13	3.89872	2	3	1	2.5	0.63246	15.9994	102.906	86.9061	49.6126	46.0172	0.6	1.35	0.75	[illegible]

[illegible]

1.78	1.91	0.13	1.832	0.09192	4	5	1	4.6	0.70711	10	13	3	11.8	2.12132	2	3
1.13	3.44	2.31	2.652	1.32129	2	8	6	3.6	3.11677	5	16	11	13.2	5.69461	2	3
1.36	2.28	0.92	1.944	0.42232	3	9	6	5	2.73861	7	14	7	10.6	3.58818	2	3
1.96	2.54	0.58	2.44333	0.41012	5	6	1	5.83333	0.70711	11	14	3	11.5	2.12132	2	3
1.61	2.55	0.94	2.20286	0.55366	3	6	3	4	1.22474	12	16	4	14.5714	2.22004	2	3
2.16	3.98	1.82	3.72	1.28693	2	5	3	2.42857	2.12132	6	17	11	15.4286	7.77817	2	3
0.98	1.96	0.98	1.26	0.69296	2	5	3	2.85714	2.12132	1	14	13	4.71429	9.19239	1	2
1.9	3.44	1.54	2.365	0.72136	2	6	4	4.25	1.70783	11	16	5	14.5	2.38048	2	3
2.54	2.96	0.42	2.75	0.29698	4	6	2	5	1.41421	11	17	6	14	4.24264	2	3
0.79	2.96	2.17	1.9225	1.31509	2	6	4	4	1.78885	1	17	16	9	10.1193	1	2
1.91	3.44	1.53	2.68	0.96137	2	5	3	3.25	1.64317	10	16	6	13.25	3.50714	2	3
0.82	2.58	1.76	2.13125	0.70542	3	5	2	3.875	1.14434	1	16	15	12.75	6.03955	1	3
1.1	3.44	2.34	2.255	0.99497	2	8	6	4.25	2.62996	3	16	13	11.5	6.13732	2	3
0.89	2.28	1.39	1.8825	0.72666	4	6	2	5	1.26491	2	15	13	10.5	6.72309	1	3
1.55	3.44	1.89	2.86909	1.07182	2	4	2	2.54545	1.07529	7	16	9	14.1818	4.68375	2	3
1.13	2.18	1.05	1.862	0.48175	4	8	4	4.8	2	5	15	10	11	4.67707	2	3

[illegible]

1.1	2.28	1.18	1.97818	0.5398	2	8	6	4.45455	2.70963	3	13	10	9.36364	4.4129	2	3	[illegible]
0.82	2.58	1.76	2.26545	0.7257	3	4	1	3.18182	0.52223	1	16	15	13.5455	6.59201	1	3	[illegible]
1.24	2.28	1.04	2.02333	0.55867	5	8	3	6.16667	1.2693	10	14	4	11.1667	2.36878	2	3	[illegible]
1.12	2.28	1.16	2.03636	0.4533	3	8	5	4.54545	1.91741	4	14	10	10.3636	4.11239	2	3	[illegible]
0.93	3.44	2.51	2.58182	1.26395	2	5	3	2.81818	1.29777	1	16	15	12.3636	7.1635	1	3	[illegible]
1.24	3.44	2.2	2.76	1.26679	2	8	6	3.27273	3.10745	14	16	2	15.2727	1.32288	2	3	[illegible]
1.38	2.2	0.82	1.76727	0.32581	4	9	5	5	2.90887	6	13	7	11.2727	4.36771	2	3	[illegible]
1.24	1.9	0.66	1.72	0.3615	3	8	5	4.72727	2.51992	11	14	3	12.9091	1.77482	2	3	[illegible]
1.3	1.9	0.6	1.73636	0.32863	3	6	3	4.18182	1.46629	4	14	10	10.1818	4.91426	2	3	[illegible]
2	2.58	0.58	2.40364	0.33033	3	6	3	3.54545	1.59099	12	16	4	15.0909	2.06912	2	3	[illegible]
1.27	2.1	0.83	1.53727	0.4925	4	8	4	6.72727	2.35186	10	17	7	15.5455	3.63146	2	3	[illegible]
1.25	1.83	0.58	1.71	0.3309	4	8	4	4.72727	2.35339	8	15	7	12.9091	2.63847	2	3	[illegible]
1.2	3.44	2.24	2.77	1.02703	2	8	6	3.875	2.79029	9	17	8	15	2.89499	2	3	[illegible]
1.23	2.2	0.97	1.86636	0.4895	4	8	4	5.45455	1.98746	10	14	4	12.2727	2.16795	2	3	[illegible]
0.98	3.44	2.46	2.82091	1.18037	2	4	2	2.45455	1.05887	1	16	15	14.1818	5.76825	1	2	[illegible]
0.98	3.44	2.46	2.19545	1.54307	2	5	3	2.27273	1.13389	1	16	15	9.09091	9.71744	1	2	[illegible]
0.89	3.44	2.55	2.52818	1.33515	2	6	4	2.81818	1.92627	2	16	14	11.6364	7.57593	1	2	[illegible]
1.9	3.44	1.54	2.93091	0.93326	2	4	2	2.72727	1.32288	11	16	5	14.9091	2.58602	2	3	[illegible]
1.28	1.9	0.62	1.69333	0.43841	3	9	6	5	4.24264	8	14	6	12	4.24264	2	3	[illegible]
1.28	2.28	1	1.94667	0.70711	5	9	4	6.33333	2.82843	8	9	1	8.66667	0.70711	3	3	[illegible]
[illegible]																	

1.17	3.98	2.81	2.77	1.44503	2	8	6	4.33333	3.21455	8	17	9	14	5.19615	2	3	1	2.33	0.57735	18.9984	150.36	131.362	68.2705	71.5661	0.5	1.85	1.35	1.12	0.68252	28	102	74	76.3333	41.8848	0	0	0	0	0	0	0	0	0	0	0.0089	13	12.9911	4.34553	7.49499	53.53	1345	1291.47	523.377	713.991	0	0	0	0	0	0	0	0	0	0	105.735	129	3.68686	3.68686	7.7787	1.5708	1.5708	1.5708	-2.8044
0.89	3.44	2.55	2.42	1.36847	2	6	4	3.5	2.09762	2	16	14	11	7.26636	1	3	2	2	0.89443	15.9994	137.327	121.328	57.0048	62.6654	0.6	2.15	1.55	1.18	0.81056	14	101	87	70.75	45.01	0	0	0	0	0	317.5	4970	4652.5	1806.25	2406.39	0.02658	91	90.9734	27.2633	47.4631	54.8	1728	1673.2	709.4	889.718	0	0	0	0	0	0	0	0	0	0	124.077	63	4.69777	5.42838	5.42838	1.5708	2.03022	1.5708	-1.8945
1.23	2.01	0.78	1.75727	0.40127	4	8	4	5.09091	2.19089	11	14	3	12.6364	1.59687	2	3	1	2.64	0.59161	63.546	164.93	101.384	94.5032	53.2539	1.25	1.75	0.5	1.42	0.25199	23	84	61	63	30.775	1.72E-08	0.0005	0.0005	0.00018	0.0003	2760	5400	2640	4014.55	1346.71	16	400	384	171.636	213.375	1211.4	1734	522.6	1407.15	257.845	0	0	0	0	0	6.00E-06	1.65E-05	1.05E-05	1.12E-05	5.51E-06	192.962	71	4.18437	6.64134	7.97608	1.83621	2.0002	1.5708	-0.6307
1.66	2.55	0.89	2.21714	0.51316	4	4	0	4	0	6	16	10	12.2857	5.73212	2	3	1	2.43	0.65465	51.9961	78.96	26.9639	68.5037	16.0493	1.15	1.4	0.25	1.26	0.16366	57	93	36	78.7143	21.8828	1.00E-08	1.27E-07	1.17E-07	5.24E-08	6.57E-08	3350	5940	2590	4165.71	1315.06	0.52	94	93.48	36.5829	55.3826	494	2180	1686	1111.14	954.662	8.3	170	161.7	76.1714	103.756	0	0	0	0	0	123.067	12	3.54823	6.1312	6.58855	1.84343	2.02219	1.5708	-0.301
1.22	2.01	0.79	1.75455	0.40669	4	5	1	4.27273	0.54772	3	14	11	9.90909	5.43599	2	3	1	2.64	0.59161	63.546	88.9059	25.3599	73.7692	12.357	1.25	1.8	0.55	1.44	0.27884	25	84	59	63.4545	29.7035	1.72E-08	0.0005	0.0005	0.00018	0.0003	3300	5400	2100	4161.82	1158.54	17	400	383	171.909	213.074	1211.4	1799	587.6	1424.88	292.025	0	0	0	0	0	6.00E-06	1.65E-05	1.05E-05	1.11E-05	5.52E-06	195.295	71	4.2035	6.66767	8.00527	1.83646	2.00034	1.5708	-0.6394
1.27	3.44	2.17	2.76545	1.25446	2	8	6	3.27273	3.10745	14	17	3	15.8182	1.28695	2	3	1	2.18	0.53033	15.9994	174.967	158.968	47.1001	83.1162	0.6	1.75	1.15	0.9	0.6086	20	101	81	83.3636	41.8961	0	0	0	0	0	0	0	0	0	0	0.02658	150	149.973	30.1987	78.0909	54.8	1925	1870.2	691.6	1162.5	0	0	0	0	0	0	0	0	0	0	135.892	12	4.69902	5.53461	5.53461	1.69745	1.83548	1.69745	-3.5505
1.25	1.96	0.71	1.74455	0.37378	4	8	4	5.45455	1.98746	11	15	4	13.1818	2.08567	2	3	1	2.64	0.59161	63.546	168.934	105.388	112.348	51.5071	1.35	1.75	0.4	1.5	0.19875	21	83	62	62.0909	31.4794	1.72E-08	6.76E-07	6.59E-07	2.32E-07	3.38E-07	0	0	0	0	0	17	400	383	174.455	211.083	505.08	1818	1312.92	1173.22	660.115	45	140	95	84.2727	52.191	1.33E-05	2.20E-05	8.70E-06	1.76E-05	4.35E-06	225.684	12	4.19232	7.98209	8.09138	1.83285	1.06098	1.5708	-0.3426
0.93	3.98	3.05	3.18909	1.9774	2	9	7	2.81818	3.03822	1	17	16	13.0909	9.91696	1	3	2	1.91	0.78446	18.9984	238.029	219.031	39.636	95.7293	0.5	1.8	1.3	0.85	0.87206	11	102	91	80.2727	55.6085	0	0	0	0	0	0	0	0	0	0	0.0277	140	139.972	27.9292	81.4335	53.53	1405.3	1351.77	234.116	594.358	0	0	0	0	0	0	0	0	0	0	137.274	139	5.12983	5.12983	6.35373	1.98634	1.98634	1.5708	-3.398
1.12	2.28	1.16	2.06091	0.67773	3	8	5	6.09091	1.89128	4	15	11	9.36364	4.42458	2	3	1	2.91	0.43853	30.9738	195.084	164.11	170.171	74.3724	1	1.85	0.85	1.41	0.35151	32	90	58	63.4545	24.5482	1.00E-07	7.40E-07	6.40E-07	2.21E-07	3.73E-07	0	0	0	0	0	0.236	72	71.764	54.3851	44.0647	317.3	2041.4	1724.1	1707.68	877.797	11	230	219	172.273	143.859	0	0	0	0	0	208.739	38	4.31453	5.46787	9.26091	1.5708	1.87047	1.5708	-0.9017
1.27	1.9	0.63	1.72818	0.34507	3	8	5	4.72727	2.51992	11	17	6	13.7273	2.92404	2	3	1	2.64	0.59161	28.0855	174.967	146.882	81.0388	73.1424	1.1	1.75	0.65	1.37	0.31603	20	85	65	62.5455	32.759	1.72E-08	0.001	0.001	0.00036	0.00059	0	0	0	0	0	16	400	384	204.364	193.163	1357.77	1925	567.23	1632.19	280.067	48	140	92	100.364	44.6677	2.60E-06	1.65E-05	1.39E-05	9.65E-06	7.29E-06	176.906	71	4.06376	6.47397	7.73448	1.83662	2.0026	1.5708	-0.6261
1.13	2.2	1.07	1.7	0.44852	5	8	3	5.54545	1.76505	5	13	8	11.2727	4.69451	2	3	1	2.27	0.67937	106.42	140.908	34.4876	118.798	16.3105	1.4	1.85	0.45	1.59	0.19734	31	79	48	69.3636	27.9257	8.00E-08	7.00E-07	6.20E-07	1.95E-07	3.63E-07	1215	3070	1855	1577.27	949.939	13	82	69	68.5455	40.177	429.75	1828.05	1398.3	698.368	708.488	0	0	0	0	0	6.70E-06	3.21E-05	2.54E-05	2.56E-05	1.62E-05	280.311	123	4.75734	4.75734	12.3855	1.5708	1.5708	1.5708	-0.473
0.82	2.54	1.72	1.96909	0.67764	4	6	2	5.27273	0.82248	1	13	12	11.1818	4.47214	1	3	2	2.27	0.82248	39.0983	196.967	157.868	137.807	65.9826	1.35	2.2	0.85	1.54	0.30643	10	79	69	69.4545	25.6991	2.20E-08	8.00E-08	5.80E-08	5.85E-08	3.68E-08	1215	2000	785	1477.27	394.498	82	320	238	170.182	151.221	336.53	1337.33	1000.8	751.305	591.948	0	0	0	0	0	0	0	0	0	0	270.339	187	8.28328	8.28317	4.54969	1.5708	1.5708	2.0944	-0.2841
1.9	2.1	0.2	2.01	0.11794	4	5	1	4.66667	0.60302	11	16	5	14	2.86039	2	3	1	2.33	0.60302	63.546	127.6	64.054	104.767	37.502	1.35	1.45	0.1	1.39	0.04395	72	92	20	83.8333	11.4515	1.72E-08	0.0001	1.00E-04	5.00E-05	6.39E-05	2500	3570	1070	2911.67	597.559	3	400	397	146	231.568	505.08	1357.77	852.69	898.1	427.273	58	140	82	88.8333	46.3901	0	0	0	0	0	172.013	44	4.35067	6.0791	7.50134	1.86502	1.98799	1.5708	-0.154
1.1	2.54	1.44	1.88333	0.72831	4	8	4	6	2	3	14	11	9.33333	5.68624	2	3	1	2.67	0.57735	72.64	196.967	124.327	136.171	62.2084	1.25	1.95	0.7	1.52	0.37859	33	84	51	62.3333	26.3502	2.20E-08	0.0005	0.0005	0.00017	0.00029	1740	5400	3660	3205	1936.12	13	320	307	131	165.357	1193	1337.33	144.33	1247.24	78.5579	0	0	0	0	0	6.00E-06	1.42E-05	8.20E-06	1.08E-05	4.26E-06	144.496	186	4.52305	4.52306	8.15569	1.5708	1.5708	2.09439	-0.883
1.25	3.44	2.19	2.57286	1.02855	2	8	6	3.71429	3.16228	14	16	2	15.1429	0.74536	2	3	1	2.29	0.52705	12.0107	168.934	156.924	58.5559	81.4576	0.6	1.75	1.15	0.96	0.58618	21	101	80	77	41.3797	0	0	0	0	0	0	0	0	0	0	0.02583	140	139.974	24.8721	55.5111	54.8	3800	3745.2	1095.96	1568.74	0	0	0	0	0	0	0	0	0	0	92.2872	164	3.63145	3.63146	8.08071	1.5708	1.5708	2.0944	-2.4094
2.02	2.55	0.53	2.338	0.37477	4	6	2	4.8	1.41421	15	16	1	15.6	0.70711	2	2	0	2	0	78.96	208.98	130.02	130.968	91.9383	1.15	1.6	0.45	1.33	0.3182	87	93	6	90.6	4.24264	1.00E-08	1.30E-06	1.29E-06	5.26E-07	9.12E-07	1790	3350	1560	2726	1103.09	0.52	8	7.48	3.512	5.28916	494	544.4	50.4	514.16	35.6382	8.3	31	22.7	17.38	16.0513	0	0	0	0	0	145.789	166	9.89467	9.89467	9.89467	0.42656	0.42656	0.42656	-0.3875
1.1	2.55	1.45	1.90636	0.82234	2	8	6	4.54545	3.41822	10	16	6	14	2.63579	2	3	1	2.55	0.62828	12.0107	173.04	161.029	79.0546	92.1242	0.7	1.75	1.05	1.2	0.60213	17	95	78	61.5455	44.355	7.20E-08	1.00E-05	9.93E-06	4.65E-06	6.16E-06	1590	18350	16760	9822.73	9933.72	39	140	101	94.3636	57.3151	1097	3800	2703	2440.36	1590.44	31	180	149	59	71.9812	7.10E-06	2.63E-05	1.92E-05	1.52E-05	1.09E-05	162.5	25	3.62415	11.9062	3.76595	1.5708	1.5708	1.5708	0.02679
0.89	3.98	3.09	3.34636	1.63641	2	6	4	2.36364	1.75412	2	17	15	14.9091	6.64773	1	2	1	1.91	0.43853	10.811	137.327	126.516	28.2669	52.8238	0.5	2.15	1.65	0.71	0.72218	14	102	88	91.0909	38.3346	0	0	0	0	0	0	0	0	0	0	0.0277	27	26.9723	6.5656	16.633	53.53	2349	2295.47	556.931	1352.44	0	0	0	0	0	0	0	0	0	0	125.967	12	4.51998	4.88036	6.56716	1.90213	1.9515	1.5708	-3.4871
2.1	3.44	1.34	2.99333	0.94752	2	5	3	3	2.12132	16	16	0	16	0	2	2	0	2	0	15.9994	127.6	111.601	53.1996	78.9135	0.6	1.4	0.8	0.87	0.56569	92	101	9	98	6.36396	0	0	0	0	0	317.5	2610	2292.5	1081.67	1621.04	0.02658	3	2.97342	1.01772	2.10253	54.8	722.66	667.86	277.42	472.248	0	0	0	0	0	0	0	0	0	0	68.8203	63	3.26114	3.43751	6.35193	1.31119	1.5708	1.5708	-0.7074
1	3.04	2.04	1.618	1.09454	2	5	3	3.8	1.30931	2	15	13	7.2	8.5105	1	2	1	1.4	0.65465	14.0067	121.76	107.753	51.2001	49.0377	0.65	1.8	1.15	1.5	0.59612	16	100	84	47.2	51.3142	0	0	0	0	0	333.6	3810	3476.4	3036.72	1817.34	0.02583	200	199.974	124.805	123.483	63.05	1115	1051.95	862.366	545.139	0	0	0	0	0	0	0	0	0	0	189.072	221	5.73952	5.73952	5.73952	1.5708	1.5708	1.5708	0.53881
2.01	2.28	0.27	2.2125	0.19092	4	6	2	5.5	1.41421	10	14	4	11	2.82843	2	3	1	2.75	0.70711	72.64	195.084	122.444	164.473	86.581	1.25	1.35	0.1	1.33	0.07071	68	84	16	72	11.3137	1.06E-07	0.0005	0.0005	0.00013	0.00035	2680	5400	2720	3360	1923.33	60	72	12	69	8.48528	1211.4	2041.4	830	1833.9	586.899	0	0	0	0	0	6.00E-06	8.80E-06	2.80E-06	8.10E-06	1.98E-06	125.388	140	5.57338	5.57338	5.64141	2.08745	2.08745	1.5708	-0.3212
1.3	2.01	0.71	1.64667	0.35529	4	6	2	4.66667	1.1547	4	14	10	7.66667	5.50757	2	3	1	2.67	0.57735	50.9415	178.49	127.549	100.691	68.2443	1.25	1.55	0.3	1.38	0.15275	50	84	34	62.6667	18.5831	2.00E-07	0.0005	0.0005	0.00017	0.00029	3010	5400	2390	4323.33	1212.45	23	60	37	38	19.4679	1211.4	2506	1294.6	1966.8	673.835	0	0	0	0	0	5.90E-06	8.40E-06	2.50E-06	6.77E-06	1.42E-06	99.7143	139	3.72246	3.72246	7.66238	1.81616	1.81616	1.5708	-0.6013
1.57	2.66	1.09	2.29667	0.77075	2	5	3	4	2.12132	2	17	15	12	10.6066	1	2	1	1.67	0.70711	9.01218	126.904	117.892	87.607	83.3624	1.05	1.4	0.35	1.28	0.24749	77	97	20	90.3333	14.1421	3.80E-08	1E+15	1E+15	6.7E+14	7.1E+14	0	0	0	0	0	0.449	190	189.551	63.6327	134.033	386.85	1560	1173.15	777.9	829.542	7.7	130	122.3	48.4667	86.4792	0	0	0	0	0	247.774	72	6.56966	6.13269	7.61661	2.0167	1.98514	1.5708	-0.6902
1.2	2.28	1.08	1.76	0.48365	5	8	3	5.75	1.64317	9	13	4	11	2.52982	2	3	1	2.5	0.63246	102.906	151.964	49.0585	121.126	23.3457	1.35	1.85	0.5	1.58	0.22583	18	79	61	60.25	31.6907	4.30E-08	9.00E-07	8.57E-07	2.76E-07	4.56E-07	0	0	0	0	0	14	150	136	82	60.821	429.75	2237	1807.25	1048.88	933.978	0	0	0	0	0	8.20E-06	3.50E-05	2.68E-05	2.69E-05	1.37E-05	182.88	63	4.32245	5.61907	8.15709	1.5708	1.5708	1.176	-0.6465
1.69	3.44	1.75	2.79286	0.73151	2	6	4	3.85714	1.91485	12	17	5	15.1429	2.01384	2	3	1	2.14	0.40825	15.9994	207.2	191.201	89.9594	93.4134	0.6	1.8	1.2	1.19	0.56507	75	101	26	91.2857	12.0393	0	0	0	0	0	206	2310	2104	839.571	866.753	0.0089	97	96.9911	23.8673	39.1224	54.8	600.61	545.81	321.177	284.88	0	0	0	0	0	0	0	0	0	0	154.239	12	3.85468	6.50658	7.02294	1.8488	1.20427	1.87152	-1.4146
1.3	3.04	1.74	2.29429	1.23037	2	9	7	5	4.94975	4	15	11	10.2857	7.77817	2	3	1	2.43	0.70711	14.0067	232.038	218.031	107.449	154.171	0.65	1.8	1.15	1.14	0.81317	47	100	53	77.2857	37.4767	0	0	0	0	0	333.6	2490	2156.4	1257.77	1524.81	0.02583	54	53.9742	23.1576	38.1655	63.05	2115	2051.95	942.457	1450.95	0	0	0	0	0	0	0	0	0	0	117.652	166	9.34375	9.34375	9.34375	0.41715	0.41715	0.41715	-1.7307
1.33	2.01	0.68	1.67	0.48083	4	5	1	4.5	0.70711	4	14	10	9	7.07107	2	3	1	2.5	0.70711	72.64	91.224	18.584	81.932	13.1409	1.25	1.55	0.3	1.4	0.21213	49	84	35	66.5	24.7487	4.33E-07	0.0005	0.0005	0.00025	0.00035	3800	5400	1600	4600	1131.37	23	60	37	41.5	26.163	1211.4	2128	916.6	1669.7	648.134	0	0	0	0	0	5.70E-06	6.00E-06	3.00E-07	5.85E-06	2.12E-07	152.189	62	7.12215	3.93408	5.4316	1.5708	1.5708	1.5708	-0.8046
0.95	2.33	1.38	1.766	0.64969	3	6	3	4.6	1.69558	2	14	12	11.2	5.77711	1	2	1	1.8	0.5	26.9815	207.2	180.218	111.197	101.817	1.25	2	0.75	1.62	0.38851	15	82	67	67.8	33.0189	2.70E-08	2.10E-07	1.83E-07	1.22E-07	1.03E-07	0	0	0	0	0	35	235	200	115	122.474	600.61	1050	449.39	823.632	233.753	0	0	0	0	0	2.25E-05	2.89E-05	6.40E-06	2.53E-05	3.68E-06	143.841	8	4.56413	4.56413	7.62156	1.87466	1.87466	1.57156	-0.0615
1.38	1.9	0.52	1.656	0.26084	3	9	6	4.6	2.80624	6	14	8	9.6	4.51387	2	3	1	2.6	0.61237	28.0855	238.029	209.943	80.8152	99.3986	1.1	1.75	0.65	1.35	0.30104	45	85	40	67	19.6055	2.80E-07	0.001	0.001	0.0004	0.00061	2200	5150	2950	3571	1669.47	7.8	150	142.2	68.52	83.6207	1405.3	1687	281.7	1563.46	136.35	100	120	20	108	12.2474	2.60E-06	2.17E-05	1.91E-05	1.25E-05	1.07E-05	74.3152	139	3.9741	3.9741	5.48068	1.94181	1.94181	1.5708	-0.3813
1.66	2.55	0.89	1.92	0.46656	2	4	2	3.5	1.09545	6	14	8	9.75	4.76445	2	3	1	2.5	0.63246	12.0107	69.723	57.7123	46.4315	26.7523	0.7	1.4	0.7	1.2	0.36878	57	95	38	72.5	20.5815	1.27E-07	1.00E-05	9.87E-06	2.60E-06	5.41E-06	2740	18350	15610	8242.5	7564.18	29	140	111	89.25	50.003	302.91	3800	3497.09	2115.73	1566.06	0	0	0	0	0	4.90E-06	0.00012	0.00012	3.42E-05	6.27E-05	88.9642	194	2.86753	2.86753	12.493	1.5708	1.5708	2.09439	-0.128
1.3	3.04	1.74	2.29667	0.89724	2	9	7	4.33333	4.04145	4	15	11	11	6.08276	2	3	1	2.33	0.57735	12.0107	232.038	220.027	86.0185	126.461	0.65	1.8	1.15	1.05	0.65	47	100	53	80.6667	29.2632	0	0	0	0	0	333.6	18350	18016.4	7057.87	9838.53	0.02583	140	139.974	64.6753	70.5951	63.05	3800	3736.95	1992.68	1871.48	0	0	0	0	0	0	0	0	0	0	99.6519	12	3.93447	4.03329	7.23332	1.5708	1.66253	2.08031	-1.01
1.91	3.44	1.53	3.25	0.6666	2	4	2	2.36364	0.98058	10	17	7	15.6364	2.78043	2	3	1	2.09	0.43853	15.9994	58.6934	42.694	23.4177	20.4364	0.6	1.35	0.75	0.74	0.37481	67	101	34	97.5455	14.7804	0	0	0	0	0	206	4970	4764	720.182	2051.06	0.0089	91	90.9911	8.29368	39.896	54.8	1728	1673.2	228.145	726.687	0	0	0	0	0	0	0	0	0	0	131.444	148	4.66221	4.66221	7.48359	1.88756	1.5708	2.0944	-0.4124
1.38	2.58	1.2	2.00333	0.60136	3	9	6	5.66667	3.05505	6	16	10	12.3333	5.50757	2	3	1	2.33	0.57735	32.065	238.029	205.964	130.618	103.267	1	1.75	0.75	1.4	0.37749	45	94	49	75.6667	26.727	2.80E-07	1.00E+23	1.00E+23	3.33E+22	5.77E+22	0	0	0	0	0	0.205	27	26.795	17.0683	14.6809	388.36	1405.3	1016.94	899.147	508.486	7.7	100	92.3	49.9	46.6544	0	0	0	0	0	138.276	129	4.0665	4.0665	8.36189	1.5708	1.5708	1.5708	-0.9968
1.55	3.04	1.49	1.9	0.77349	2	4	2	3.6	1.06904	7	15	8	9.8	4.65986	2	3	1	2.6	0.65465	14.0067	69.723	55.7163	49.7088	25.0517	0.65	1.4	0.75	1.23	0.39097	60	100	40	72.2	21.5208	0	0	0	0	0	333.6	5150	4816.4	3704.72	2574.71	0.02583	29	28.9742	10.4852	13.0086	63.05	1519	1455.95	984.592	880.484	0	0	0	0	0	0	0	0	0	0	53.8239	221	3.77567	3.77566	3.77562	1.5708	1.5708	1.5708	-0.3399
1.38	3.44	2.06	2.87818	1.45664	2	9	7	3.90909	4.94975	6	16	10	13.2727	7.07107	2	3	1	2.27	0.70711	15.9994	238.029	222.03	76.5529	156.999	0.6	1.75	1.15	0.91	0.81317	45	101	56	85.7273	39.598	0	0	0	0	0	317.5	3155	2837.5	1091.36	2006.42	0.02658	27	26.9734	7.38297	19.0731	54.8	1405.3	1350.5	423.118	954.948	0	0	0	0	0	0	0	0	0	0	153.331	12	6.52385	6.5365	4.15463	1.57156	1.56927	2.09552	-2.9935
1.13	1.96	0.83	1.72273	0.44667	5	8	3	5.81818	1.64317	5	14	9	10.4545	4.39886	2	3	1	2.64	0.59161	107.868	140.908	33.0394	120.821	16.1604	1.45	1.85	0.4	1.61	0.19462	31	83	52	64.4545	25.9692	1.63E-08	7.00E-07	6.84E-07	2.39E-07	3.51E-07	2280	2600	320	2476.36	156.908	13	430	417	184.273	229.945	505.08	1234.93	729.85	962.185	425.158	29	100	71	65.3636	35.144	6.70E-06	2.20E-05	1.53E-05	1.67E-05	7.70E-06	301.534	71	4.36117	8.39755	9.49602	1.8025	2.0288	1.5708	-0.1797
1.96	3.98	2.02	3.07	0.97961	2	5	3	2.90909	1.55519	14	17	3	15.6364	1.36399	2	2	0	2	0	15.9994	118.71	102.711	46.1906	52.8934	0.5	1.45	0.95	0.84	0.46892	83	102	19	95.3636	9.77182	0	0	0	0	0	0	0	0	0	0	0.02658	67	66.9734	18.3114	35.3661	53.53	505.08	451.55	201.121	237.679	0	0	0	0	0	0	0	0	0	0	172.506	146	4.54267	6.79281	6.79281	1.7956	2.01027	1.7956	-2.3298
1.5	2.58	1.08	2.142	0.65538	2	6	4	4	2.09165	5	16	11	11.2	6.39336	2	3	1	2.4	0.61237	12.0107	180.948	168.937	87.6073	95.7041	0.7	1.45	0.75	1.12	0.36358	52	95	43	77.4	25.9278	1.35E-07	1.00E+23	1.00E+23	4.00E+22	6.12E+22	0	0	0	0	0	0.205	140	139.795	50.882	64.1122	388.36	3800	3411.64	2231.34	1895.34	7.7	200	192.3	89.68	113.186	0	0	0	0	0	84.4219	166	3.28701	3.28701	9.2198	1.5708	1.75001	2.09439	-1.0488
1.5	2.58	1.08	2.142	0.65538	2	6	4	4	2.09165	5	16	11	11.2	6.39336	2	3	1	2.4	0.61237	12.0107	180.948	168.937	87.6073	95.7041	0.7	1.45	0.75	1.12	0.36358	52	95	43	77.4	25.9278	1.35E-07	1.00E+23	1.00E+23	4.00E+22	6.12E+22	0	0	0	0	0	0.205	140	139.795	50.882	64.1122	388.36	3800	3411.64	2231.34	1895.34	7.7	200	192.3	89.68	113.186	0	0	0	0	0	83.8062	164	3.28873	3.28875	8.94722	1.57078	1.5708	2.0944	-1.049
1.2	3.98	2.78	2.87333	1.47409	2	8	6	3.75	3.4641	9	17	8	14	4.3589	2	3	1	2.33	0.57735	15.9994	151.964	135.965	62.3206	77.6479	0.5	1.85	1.35	0.98	0.75222	18	102	84	73.6667	48.2113	0	0	0	0	0	0	0	0	0	0	0.02658	14	13.9734	4.68476	8.06723	53.53	1099	1045.47	402.443	603.236	0	0	0	0	0	0	0	0	0	0	84.4324	166	6.96887	6.96887	6.96887	0.55847	0.55847	0.55847	-3.2058
0.79	3.04	2.25	2.1175	1.22181	2	6	4	3.75	2.25832	1	15	14	9	7.79744	1	3	2	2	0.89443	14.0067	132.905	118.899	63.4563	65.057	0.65	2.6	1.95	1.34	1.01032	8	100	92	65.25	48.3436	0	0	0	0	0	0	0	0	0	0	0.02583	54	53.9742	22.5129	29.5613	63.05	2750	2686.95	794.423	1433.45	0	0	0	0	0	0	0	0	0	0	179.348	227	6.33001	6.33001	6.33001	1.5708	2.0944	2.0944	-0.8532
1.81	2.66	0.85	2.46364	0.5106	4	5	1	4.81818	0.58835	10	17	7	15.6364	3.57878	2	3	1	2.09	0.43853	69.723	126.904	57.1815	114.646	33.5025	1.3	1.4	0.1	1.38	0.05883	69	97	28	91.5455	14.3151	1.08E-07	1E+15	1E+15	7.3E+14	6.8E+14	0	0	0	0	0	0.449	72	71.551	12.1447	33.31	302.91	1828.05	1525.14	502.606	641.232	0	0	0	0	0	0	0	0	0	0	503.269	12	7.95503	8.40333	8.40333	1.67655	1.1266	1.67655	-0.5558

[illegible]

1.6	3.44	1.84	2.86167	1.15984	2	5	3	2.83333	1.71594	5	16	11	13.6667	5.696	2	3	1	2.17	0.52705	15.9994	92.9064	76.907
0.82	3.98	3.16	3.28333	1.64458	2	4	2	2.66667	1.33333	1	17	16	14.3333	8.43274	1	2	1	1.83	0.52705	18.9984	79.904	60.9056
1.12	3.44	2.32	2.75167	1.39232	2	8	6	3.33333	3.12694	4	16	12	12.1667	7.67753	2	3	1	2.33	0.66667	15.9994	140.116	124.117
1.24	3.44	2.2	2.865	1.21364	2	8	6	3.16667	3.10018	14	16	2	15.5	1.08012	2	3	1	2.17	0.52705	15.9994	167.259	151.26
1.96	3.98	2.02	3.30667	1.42836	2	5	3	3	2.12132	14	17	3	16	2.12132	2	2	0	2	0	18.9984	118.71	99.7116
1.24	3.44	2.2	2.77167	1.34612	2	8	6	3.33333	3.12694	5	16	11	13.8333	5.68135	2	3	1	2.33	0.66667	15.9994	167.259	151.26
2.33	3.16	0.83	2.88333	0.5869	3	6	3	4	2.12132	14	17	3	16	2.12132	2	2	0	2	0	35.453	207.2	171.747
1.55	2.58	1.03	2.23667	0.72832	3	4	1	3.33333	0.70711	7	16	9	13	6.36396	2	3	1	2.33	0.70711	32.065	54.938	22.873
1.31	3.44	2.13	2.78833	1.31114	2	4	2	2.5	1.08012	2	16	14	12	8.16497	1	3	2	2	0.8165	15.9994	51.9961	35.9967
1.66	3.44	1.78	2.85167	1.17673	2	5	3	2.83333	1.71594	6	16	10	13.6667	5.27046	2	3	1	2.33	0.66667	15.9994	112.411	96.4116
1.23	3.44	2.21	2.765	1.35842	2	8	6	3.5	3.24037	5	16	11	13.6667	5.696	2	3	1	2.33	0.66667	15.9994	164.93	148.931
2.02	2.55	0.53	2.285	0.37477	4	6	2	5	1.41421	15	16	1	15.5	0.70711	2	2	0	2	0	78.96	208.98	130.02
1.63	3.44	1.81	2.725	0.94637	1	4	3	2	1.2792	1	16	15	9.16667	8.90608	1	3	2	1.83	0.87905	1.00794	50.9415	49.9336
1.5	3.44	1.94	2.88333	1.13495	2	6	4	2.66667	2.10819	5	16	11	13.6667	5.696	2	3	1	2.17	0.52705	10.811	180.948	170.137
1.22	3.98	2.76	3.29	1.95161	2	5	3	2.75	2.12132	3	17	14	13.5	9.89949	2	3	1	2.25	0.70711	18.9984	88.9059	69.9074
1.38	3.44	2.06	2.85833	1.19143	2	9	7	3.5	3.62859	6	16	10	14	5.16398	2	3	1	2.17	0.52705	15.9994	238.029	222.03
1.36	3.44	2.08	2.885	1.16057	2	4	2	2.5	1.08012	3	16	13	13.6667	6.76593	2	3	1	2.17	0.52705	15.9994	44.9559	28.9565
2.2	2.55	0.35	2.43333	0.24749	4	5	1	4.33333	0.70711	10	16	6	14	4.24264	2	3	1	2.33	0.70711	78.96	106.42	27.46
2.2	2.58	0.38	2.45333	0.2687	3	5	2	3.66667	1.41421	10	16	6	14	4.24264	2	3	1	2.33	0.70711	32.065	106.42	74.355
1.91	2.58	0.67	2.22333	0.3371	3	4	1	3.66667	0.57735	10	16	6	13.6667	3.21455	2	3	1	2.33	0.57735	32.065	74.9216	42.8566
1.33	2.18	0.85	1.89667	0.60104	4	5	1	4.33333	0.70711	4	15	11	11.3333	7.77817	2	3	1	2.33	0.70711	74.9216	91.224	16.3024
0.95	3.44	2.49	2.50083	1.4932	2	5	3	3.08333	1.74956	2	16	14	10.5	8.66448	1	3	2	1.92	0.84824	15.9994	87.62	71.6206
1.6	2.66	1.06	2.48333	0.74953	5	5	0	5	0	5	17	12	15	8.48528	2	3	1	2.17	0.70711	92.9064	126.904	33.9981
1.2	2.66	1.46	2.17333	1.03238	5	8	3	6	2.12132	9	17	8	14.3333	5.65685	2	3	1	2.33	0.70711	126.904	151.964	25.0595
0.98	3.44	2.46	2.47167	1.40802	2	2	0	2	0	1	16	15	10.6667	8.79049	1	2	1	1.67	0.60302	6.941	15.9994	9.0584
0.95	3.98	3.03	3.08667	1.80938	2	5	3	2.83333	1.71594	2	17	15	13.6667	7.81736	1	3	2	2	0.8165	18.9984	87.62	68.6216
1.6	2.55	0.95	2.23333	0.67175	4	5	1	4.33333	0.70711	5	16	11	12.3333	7.77817	2	3	1	2.33	0.70711	78.96	92.9064	13.9464
1.54	1.91	0.37	1.78333	0.21079	3	4	1	3.66667	0.57735	4	14	10	9.33333	5.03322	2	3	1	2.67	0.57735	28.0855	58.6934	30.6079

[illegible]

1.38	3.44	2.06	2.84	1.21863	2	9	7	3.5	3.62859	6	16	10	13.5	5.40062	2	3	[illegible]
2.2	3.04	0.84	2.46667	0.53433	1	4	3	1.66667	1.56347	1	17	16	6	10.0333	1	2	[illegible]
0.79	3.44	2.65	2.61583	1.69099	2	6	4	3.33333	2.70449	1	16	15	11.3333	9.57377	1	3	[illegible]
2.2	3.44	1.24	2.61333	0.87681	1	2	1	1.33333	0.70711	1	16	15	6	10.6066	1	2	[illegible]
2.05	2.66	0.61	2.43	0.33151	3	5	2	4.33333	1.1547	15	17	2	16	1	2	2	[illegible]
1.1	3.44	2.34	2.84167	1.27673	2	8	6	3.16667	3.10018	15	16	1	15.8333	0.52705	2	3	[illegible]
1.69	3.44	1.75	2.85667	1.23744	2	5	3	3	2.12132	12	16	4	14.6667	2.82843	2	3	[illegible]
2.02	3.16	1.14	2.58667	0.57003	3	6	3	4	1.73205	15	17	2	16	1	2	2	[illegible]
1.5	3.44	1.94	2.14667	1.37179	2	6	4	4.66667	2.82843	5	16	11	8.66667	7.77817	2	3	[illegible]
2.02	3.44	1.42	2.99333	0.89572	2	6	4	3	2.16025	15	16	1	15.6667	0.66667	2	2	[illegible]
1.25	3.44	2.19	2.86667	1.20918	2	8	6	3.16667	3.10018	15	16	1	15.6667	0.66667	2	3	[illegible]
2.33	2.96	0.63	2.75	0.44548	4	6	2	4.66667	1.41421	14	17	3	16	2.12132	2	2	[illegible]
2.16	3.44	1.28	3.01333	0.9051	2	5	3	3	2.12132	6	16	10	12.6667	7.07107	2	3	[illegible]
1.9	2.19	0.29	2.09333	0.20506	3	4	1	3.33333	0.70711	11	15	4	13.6667	2.82843	2	3	[illegible]
0.98	3.44	2.46	2.28667	1.56178	2	6	4	2.33333	1.46504	1	16	15	9	9.50994	1	3	[illegible]
1.3	3.44	2.14	2.82667	1.25088	2	6	4	2.83333	2.06828	4	16	12	13.6667	6.20036	2	3	[illegible]
[illegible]	[illegible]	[illegible]	[illegible]	[illegible]	[illegible]	[illegible]	[illegible]	[illegible]	[illegible]	[illegible]	[illegible]	[illegible]	[illegible]	[illegible]	[illegible]	[illegible]	[illegible]

0.98	3.44	2.46	2.45333	1.46087	2	3	1	2.13333	0.44381	1	16	15	10.8667	9.11875	1	2	1	1.67	0.61546	6.941	30.9738	24.0328	14.9765	9.78776	0.6	1.45	0.85	0.94	0.50366	12	101	89	69.8667	53.6272	0	0	0	0	0	0	0
1.28	1.91	0.63	1.7	0.44548	4	9	5	5.66667	3.53553	8	10	2	9.33333	1.41421	3	3	0	3	0	58.6934	244	185.307	120.462	131.032	1.35	1.75	0.4	1.48	0.28284	43	67	24	59	16.9706	7.20E-08	1.50E-06	1.43E-06	5.48E-07	1.01E-06	2260	4970
0.98	3.44	2.46	2.11412	1.49513	2	4	2	2.23529	0.83527	1	16	15	8.70588	9.44999	1	2	1	1.53	0.647	6.941	72.64	65.699	18.4002	26.2558	0.6	1.45	0.85	1.08	0.52282	12	101	89	57.1176	55.54	0	0	0	0	0	317.5	6000
0.98	3.44	2.46	1.98095	1.53489	2	4	2	2.19048	0.77664	1	16	15	7.7619	9.49478	1	3	2	1.57	0.87287	6.941	65.409	58.468	15.9602	21.9567	0.6	1.45	0.85	1.12	0.53748	12	101	89	52	56.2435	0	0	0	0	0	317.5	6000
0.98	3.44	2.46	2.45333	1.46087	2	3	1	2.13333	0.44381	1	16	15	10.8667	9.11875	1	2	1	1.67	0.61546	6.941	30.9738	24.0328	14.9765	9.78776	0.6	1.45	0.85	0.94	0.50366	12	101	89	69.8667	53.6272	0	0	0	0	0	0	0
0.98	3.44	2.46	2.18571	1.45734	2	2	0	2	0	1	16	15	9.14286	9.09945	1	2	1	1.57	0.63246	6.941	15.9994	9.0584	11.376	5.36712	0.6	1.45	0.85	1	0.50892	12	101	89	60.7143	54.2808	0	0	0	0	0	317.5	6000
1.36	1.9	0.54	1.696	0.2492	3	9	6	4.6	2.80624	6	14	8	9.4	4.71699	2	3	1	2.6	0.61237	28.0855	237	208.915	79.4326	99.3826	1.1	1.75	0.65	1.35	0.30104	44	85	41	65.6	20.67	1.27E-07	0.001	0.001	0.0004	0.00061	0	16200
1	2.96	1.96	1.80333	1.12263	3	4	1	3.83333	0.47673	2	17	15	9	9.04534	1	2	1	1.5	0.6396	28.0855	79.904	51.8185	51.3546	26.3874	1.1	1.8	0.7	1.47	0.42693	16	98	82	54.8333	49.9841	3.40E-08	1.00E+18	1.00E+18	3.33E+17	6.03E+17	0	0
1.9	3.44	1.54	3.03273	0.69222	2	6	4	3.18182	1.76963	7	17	10	14.8182	4.6848	2	3	1	2.18	0.48666	15.9994	186.207	170.208	55.7888	78.3549	0.6	1.35	0.75	0.92	0.34269	58	101	43	92.2727	20.4174	0	0	0	0	0	206	4700
1.38	2.01	0.63	1.74	0.3245	4	9	5	5.66667	2.88675	6	14	8	9.33333	4.16333	2	3	1	2.67	0.57735	55.845	238.029	182.184	122.171	100.686	1.25	1.75	0.5	1.47	0.25658	45	84	39	63.3333	19.6044	1.00E-07	0.0005	0.0005	0.00017	0.00029	3155	5400
1.12	1.91	0.79	1.74417	0.45805	3	8	5	4.16667	1.91185	2	10	8	8.16667	5.05828	1	3	2	2.67	1.17444	24.305	140.116	115.811	59.7472	43.1072	1.35	1.85	0.5	1.42	0.22361	32	73	41	65.0833	16.1004	4.40E-08	7.40E-07	6.96E-07	1.23E-07	2.94E-07	2100	4970
1.25	2.28	1.03	1.71333	0.52272	3	8	5	5.33333	2.51661	9	15	6	12.3333	3.05505	2	3	1	2.67	0.57735	26.9815	168.934	141.953	99.6071	71.0338	1.25	1.75	0.5	1.45	0.26458	21	80	59	55.3333	30.6649	2.70E-08	6.76E-07	6.49E-07	2.49E-07	3.70E-07	0	0
1.23	2.01	0.78	1.71667	0.42442	4	8	4	5.33333	2.3094	10	14	4	12.3333	2.08167	2	3	1	2.67	0.57735	58.6934	164.93	106.237	98.7546	57.7326	1.25	1.75	0.5	1.45	0.26458	23	84	61	58	31.4802	7.20E-08	0.0005	0.0005	0.00017	0.00029	2760	5400
1.24	2.01	0.77	1.72	0.41869	4	8	4	5.33333	2.3094	10	14	4	12.6667	2.3094	2	3	1	2.67	0.57735	58.6934	167.259	108.566	99.5308	59.0674	1.25	1.75	0.5	1.45	0.26458	22	84	62	57.6667	32.0364	7.20E-08	0.0005	0.0005	0.00017	0.00029	2830	5400
1.23	1.96	0.73	1.7	0.4078	4	8	4	5.66667	2.08167	10	14	4	12.3333	2.08167	2	3	1	2.67	0.57735	58.6934	164.93	106.237	114.111	53.2676	1.35	1.75	0.4	1.52	0.20817	23	83	60	57.6667	31.0698	7.20E-08	8.14E-07	7.42E-07	3.34E-07	4.17E-07	2500	4970
1.28	1.61	0.33	1.5275	0.23335	3	9	6	4.5	4.24264	8	13	5	11.75	3.53553	2	3	1	2.25	0.70711	26.9815	244	217.018	81.2362	153.455	1.25	1.75	0.5	1.38	0.35355	43	80	37	70.75	26.163	2.70E-08	1.50E-06	1.47E-06	3.95E-07	1.04E-06	2260	5100
1.23	2.54	1.31	1.66667	0.92631	6	8	2	7.33333	1.41421	11	13	2	12.3333	1.41421	3	3	0	3	0	164.93	196.967	32.0362	175.609	22.653	1.35	1.75	0.4	1.62	0.28284	23	70	47	38.6667	33.234	2.20E-08	8.14E-07	7.92E-07	5.50E-07	5.60E-07	1740	2760
1.13	2.54	1.41	1.6	0.99702	6	8	2	7.33333	1.41421	5	11	6	7	4.24264	3	3	0	3	0	140.908	196.967	56.0589	159.594	39.6396	1.35	1.85	0.5	1.68	0.35355	31	70	39	44	27.5772	2.20E-08	7.00E-07	6.78E-07	4.74E-07	4.79E-07	1740	2280
0.89	1.96	1.07	1.28333	0.58859	4	6	2	5	1	2	14	12	6	6.9282	1	2	1	1.33	0.57735	40.078	137.327	97.249	98.705	51.6187	1.45	2.15	0.7	1.8	0.35	14	83	69	37.6667	39.2726	3.40E-08	3.40E-07	3.06E-07	1.63E-07	1.59E-07	1620	3810
0.89	2.33	1.44	1.97	1.01823	6	6	0	6	0	2	14	12	11	8.48528	1	2	1	1.75	0.70711	137.327	207.2	69.873	189.732	49.4077	1.8	2.15	0.35	1.89	0.24749	14	82	68	65	48.0833	2.10E-07	3.40E-07	1.30E-07	2.43E-07	9.19E-08	1260	1620
0.89	2.33	1.44	1.37	1.01823	6	6	0	6	0	2	14	12	6	8.48528	1	2	1	1.33	0.70711	137.327	207.2	69.873	160.618	49.4077	1.8	2.15	0.35	2.03	0.24749	14	82	68	36.6667	48.0833	2.10E-07	3.40E-07	1.30E-07	2.97E-07	9.19E-08	1260	1620
1	2.2	1.2	1.72	0.63498	4	5	1	4.66667	0.57735	2	14	12	8.66667	6.1101	1	3	2	2	1	40.078	118.71	78.632	88.4027	42.2991	1.4	1.8	0.4	1.55	0.21794	16	83	67	56	35.3412	3.40E-08	1.15E-07	8.10E-08	8.57E-08	4.49E-08	2500	3810
1.55	1.57	0.02	1.56333	0.01414	2	4	2	2.66667	1.41421	2	7	5	3.66667	3.53553	1	3	2	1.67	1.41421	9.01218	54.938	45.9259	24.3208	32.4745	1.05	1.4	0.35	1.17	0.24749	60	77	17	71.3333	12.0208	3.80E-08	1.44E-06	1.40E-06	5.05E-07	9.91E-07	5150	13000
1.57	1.6	0.03	1.5775	0.02121	2	5	3	2.75	2.12132	2	5	3	2.75	2.12132	1	3	2	1.5	1.41421	9.01218	92.9064	83.8942	29.9857	59.3222	1.05	1.45	0.4	1.15	0.28284	53	77	24	71	16.9706	3.80E-08	1.52E-07	1.14E-07	6.65E-08	8.06E-08	3480	13000
1.57	2.36	0.79	1.83333	0.55861	2	6	4	3.33333	2.82843	2	6	4	3.33333	2.82843	1	3	2	1.67	1.41421	9.01218	183.84	174.828	67.2881	123.622	1.05	1.35	0.3	1.15	0.21213	55	77	22	69.6667	15.5563	3.80E-08	5.40E-08	1.60E-08	4.33E-08	1.13E-08	5174	13000
0.98	2.02	1.04	1.33333	0.59475	2	6	4	4	2	1	15	14	6	7.81025	1	2	1	1.33	0.57735	6.941	208.98	202.039	85.3331	108.356	1.45	1.8	0.35	1.62	0.17559	12	87	75	38.3333	42.194	3.40E-08	1.30E-06	1.27E-06	4.76E-07	7.14E-07	1790	6000
1	1.91	0.91	1.70833	0.40924	4	4	0	4	0	2	13	11	10.1667	4.97722	1	3	2	2.17	0.87905	40.078	69.723	29.645	61.1056	13.5838	1.3	1.8	0.5	1.4	0.23061	16	81	65	65.5	29.4287	3.40E-08	1.40E-07	1.06E-07	9.97E-08	5.41E-08	2740	4970
1	2.33	1.33	1.44333	0.94045	4	6	2	4.66667	1.41421	2	14	12	6	8.48528	1	2	1	1.33	0.70711	40.078	207.2	167.122	95.7853	118.173	1.8	1.8	0	1.8	0	16	82	66	38	46.669	3.40E-08	2.10E-07	1.76E-07	9.27E-08	1.24E-07	1260	3810
0.82	3.44	2.62	2.9275	1.17182	2	4	2	2.5	1.06543	1	16	15	13.8333	6.85368	1	3	2	2	0.56949	15.9994	50.9415	34.9421	23.5138	16.0731	0.6	2.2	1.6	0.86	0.65127	10	101	91	88.3333	37.4801	0	0	0	0	0	0	0
0.89	2.28	1.39	1.78333	0.77526	4	6	2	5.33333	1.1547	2	15	13	9	6.55744	1	3	2	2	1	74.9216	195.084	120.162	135.778	60.0962	1.15	2.15	1	1.55	0.52915	14	89	75	57	38.6911	1.06E-07	3.40E-07	2.34E-07	2.59E-07	1.32E-07	0	0
0.79	2.1	1.31	1.73167	0.75544	5	8	3	5.66667	1.56347	1	16	15	12.5	7.90569	1	3	2	2	0.8165	127.6	157.25	29.65	133.426	15.3147	1.4	2.6	1.2	1.67	0.62539	8	92	84	67.1667	50.2687	2.10E-07	0.0001	9.98E-05	6.69E-05	6.62E-05	0	0
1.78	2.2	0.42	2.06	0.29698	5	5	0	5	0	10	13	3	11	2.12132	2	3	1	2.67	0.70711	106.42	114.818	8.398	109.219	5.93828	1.4	1.55	0.15	1.45	0.10607	69	79	10	72.3333	7.07107	8.00E-08	1.08E-07	2.80E-08	9.87E-08	1.98E-08	1215	3070
1.1	1.78	0.68	1.32667	0.48083	5	8	3	7	2.12132	13	16	3	15	2.12132	2	3	1	2.67	0.70711	114.818	173.04	58.222	153.633	41.1692	1.55	1.75	0.2	1.68	0.14142	17	79	62	37.6667	43.8406	8.00E-08	2.50E-07	1.70E-07	1.93E-07	1.20E-07	1215	1590
1.2	2.96	1.76	2.27333	0.94156	4	8	4	5.66667	2.08167	9	17	8	14.3333	4.6188	2	3	1	2.33	0.57735	79.904	151.964	72.06	119.591	36.5825	1.15	1.85	0.7	1.47	0.35473	18	98	80	71	45.9021	9.00E-07	1.00E+18	1.00E+18	3.34E+17	5.77E+17	0	0
0.98	3.44	2.46	2.18778	1.50865	2	4	2	2.22222	0.8165	1	16	15	9.11111	9.45604	1	2	1	1.56	0.6455	6.941	72.64	65.699	18.2668	25.5811	0.6	1.45	0.85	1.05	0.52856	12	101	89	59.5556	55.6455	0	0	0	0	0	317.5	6000
1.65	3.44	1.79	2.84333	1.26572	2	4	2	2.66667	1.41421	12	16	4	14.6667	2.82843	2	3	1	2.33	0.70711	15.9994	65.409	49.4096	32.4693	34.9379	0.6	1.35	0.75	0.85	0.53033	76	101	25	92.6667	17.6777	0	0	0	0	0	317.5	3700
0.82	3.44	2.62	2.15333	1.31062	1	4	3	2.33333	1.52753	1	16	15	6	8.66025	1	2	1	1.33	0.57735	1.00794	39.0983	38.0904	18.7019	19.1884	0.25	2.2	1.95	1.02	1.03963	10	103	93	71.3333	53.1256	0	0	0	0	0	317.5	2000
0.79	3.16	2.37	2.26167	0.9845	3	6	3	4.16667	1.25576	1	17	16	12.3333	6.74822	1	3	2	2.17	0.80861	35.453	132.905	97.4525	76.3013	46.6425	1	2.6	1.6	1.45	0.63549	8	99	91	74.5	37.6466	1.72E-08	1E+15	1E+15	1.7E+14	4.4E+14	0	0
1.24	2.28	1.04	1.98	0.47589	3	8	5	4.83333	2.14264	9	14	5	11.5	3.19801	2	3	1	2.67	0.60302	28.0855	167.259	139.174	88.6911	62.067	1.1	1.75	0.65	1.33	0.27798	22	85	63	64.5	26.8709	4.30E-08	0.001	0.001	0.00033	0.0006	2200	4700
1	2.28	1.28	1.77667	0.68237	4	6	2	5	1	2	15	13	9	6.55744	1	3	2	2	1	40.078	195.084	155.006	118.974	77.5405	1.35	1.8	0.45	1.53	0.23629	16	88	72	57.3333	37.1663	3.40E-08	4.00E-07	3.66E-07	1.80E-07	1.94E-07	2680	3810
0.98	3.44	2.46	2.06138	1.58222	2	3	1	2.06897	0.33968	1	16	15	8.10345	9.8781	1	2	1	1.48	0.66986	6.941	28.0855	21.1445	12.1475	8.22861	0.6	1.45	0.85	1.07	0.5464	12	101	89	53.8621	58.3204	0	0	0	0	0	317.5	6000
0.98	3.44	2.46	2.13556	1.53107	2	4	2	2.22222	0.8165	1	16	15	8	9.3675	1	3	2	1.67	0.86603	6.941	47.867	40.926	15.5143	15.8606	0.6	1.45	0.85	1.07	0.54256	12	101	89	55.8889	54.5474	0	0	0	0	0	317.5	6000
1.69	3.44	1.75	3.005	0.94282	2	5	3	2.66667	1.56347	12	16	4	15.3333	2.10819	2	3	1	2.17	0.52705	15.9994	112.411	96.4116	34.7456	49.8156	0.6	1.55	0.95	0.83	0.50291	75	101	26	95.5	13.4598	0	0	0	0	0	0	0
2.28	2.66	0.38	2.53333	0.2687	5	6	1	5.33333	0.70711	10	17	7	14.6667	4.94975	2	3	1	2.33	0.70711	126.904	195.084	68.1795	149.631	48.2102	1.35	1.4	0.05	1.38	0.03536	68	97	29	87.3333	20.5061	1.06E-07	1E+15	1E+15	6.7E+14	7.1E+14	0	0
1.66	3.44	1.78	2.88667	1.111	2	4	2	2.66667	1.33333	6	16	10	13.5	5.40062	2	3	1	2.33	0.66667	15.9994	63.546	47.5466	29.9233	28.2441	0.6	1.4	0.8	0.86	0.51707	57	101	44	88.8333	25.0921	0	0	0	0	0	317.5	5940
1	2.28	1.28	1.82333	0.71445	3	6	3	4.33333	1.52753	2	15	13	9	6.55744	1	3	2	2	1	30.9738	195.084	164.11	88.7119	92.2333	1	1.8	0.8	1.38	0.40104	16	90	74	58	38	3.40E-08	1.06E-07	7.20E-08	8.00E-08	3.99E-08	0	0
1.2	2.28	1.08	1.84333	0.56889	5	8	3	6.33333	1.52753	9	15	6	11.3333	3.21455	2	3	1	2.67	0.57735	121.76	195.084	73.324	156.269	36.8511	1.35	1.85	0.5	1.55	0.26458	18	88	70	58	36.0555	1.06E-07	9.00E-07	7.94E-07	4.69E-07	4.01E-07	0	0
1.55	3.44	1.89	2.91167	1.08562	2	5	3	2.83333	1.71594	6	16	10	12.8333	6.34648	2	3	1	2.33	0.66667	15.9994	95.94	79.9406	35.8126	43.0168	0.6	1.45	0.85	0.88	0.55038	56	101	45	86.6667	28.7131	0	0	0	0	0	317.5	6190
2.36	3.16	0.8	2.93	0.46869	3	6	3	3.5	1.58114	6	17	11	15	5.71548	2	3	1	2.17	0.52705	32.065	183.84	151.775	59.6195	78.5834	1	1.35	0.35	1.06	0.18447	55	99	44	90.8333	22.8096	5.40E-08	1.00E+23	1.00E+23	1.67E+22	5.27E+22	0	0
1.62	3.16	1.54	2.8	0.81208	3	6	3	4	2	11	17	6	15.3333	3.43188	2	3	1	2.17	0.52705	35.453	204.383	168.93	90.527	110.19	1	1.9	0.9	1.21	0.47332	70	99	29	90.6667	16.9837	2.20E-08	1E+10	1E+10	6.7E+09	6.7E+09	206	1740

1.25	1.61	0.36	1.43207	0.20722	3	8	5	3.17241	1.26131	2	15	13	7	7.6847	1	3	[illegible]
1.31	1.93	0.62	1.45552	0.23674	3	5	2	3.06897	0.50452	2	13	11	6.86207	7.47359	1	3	[illegible]
1.31	1.61	0.3	1.4431	0.20439	2	3	1	2.96552	0.25226	2	13	11	6.55172	7.48999	1	2	[illegible]
0.98	3.44	2.46	2.13556	1.53107	2	4	2	2.22222	0.8165	1	16	15	8	9.3675	1	3	[illegible]
1.31	2.96	1.65	1.49103	0.43356	3	4	1	3.03448	0.25226	2	17	15	7.06897	7.83698	1	2	[illegible]
1.31	1.78	0.47	1.45034	0.2193	3	5	2	3.06897	0.50452	2	13	11	6.93103	7.56307	1	2	[illegible]
1.31	1.63	0.32	1.43483	0.20784	3	4	1	3.03448	0.25518	2	13	11	6.27586	7.39013	1	3	[illegible]
1.31	2.2	0.89	1.46483	0.27851	3	5	2	3.06897	0.50452	2	13	11	6.82759	7.44129	1	3	[illegible]
1.14	1.61	0.47	1.41793	0.21432	3	8	5	3.17241	1.27589	2	13	11	6.31034	7.38288	1	3	[illegible]
1.27	1.61	0.34	1.43276	0.20612	3	8	5	3.17241	1.26131	2	17	15	7.06897	7.83698	1	3	[illegible]
1.31	2.02	0.71	1.45862	0.24938	3	6	3	3.10345	0.75679	2	15	13	7	7.6847	1	2	[illegible]
1.31	1.61	0.3	1.43414	0.21213	3	3	0	3	0	2	13	11	6.55172	7.77817	1	2	[illegible]
1.1	1.61	0.51	1.4269	0.21902	3	8	5	3.17241	1.26131	2	16	14	7.03448	7.75711	1	3	[illegible]
1.31	1.61	0.3	1.44448	0.21213	3	3	0	3	0	2	13	11	6.93103	7.77817	1	2	[illegible]
1.31	1.96	0.65	1.45655	0.24078	3	5	2	3.06897	0.50452	2	14	12	6.96552	7.61995	1	2	[illegible]
1.31	2.96	1.65	1.49103	0.43356	3	4	1	3.03448	0.25226	2	17	15	7.06897	7.83698	1	2	[illegible]
[illegible]	[illegible]	[illegible]	[illegible]	[illegible]	[illegible]	[illegible]	[illegible]	[illegible]	[illegible]	[illegible]	[illegible]	[illegible]	[illegible]	[illegible]	[illegible]	[illegible]	[illegible]

0.79	2.96	2.17	1.90667	1.34986	2	6	4	4.33333	1.7581	1	17	16	[illegible]
0.98	2.28	1.3	1.7525	0.77725	1	5	4	1.875	1.64637	1	9	8	[illegible]
0.98	3.44	2.46	2.36	1.48048	2	4	2	2.25	0.8584	1	16	15	[illegible]
1.31	1.61	0.3	1.44069	0.20228	3	6	3	3.10345	0.75679	2	13	11	[illegible]
0.98	3.44	2.46	2.475	1.30946	2	2	0	2	0	1	16	15	[illegible]
1.13	1.61	0.48	1.41759	0.21521	3	8	5	3.17241	1.27589	2	13	11	[illegible]
0.98	3.44	2.46	2.24	1.51884	2	8	6	3.5	3.28634	1	16	15	[illegible]
0.98	3.04	2.06	1.8	1.10316	1	2	1	1.75	0.54772	1	15	14	[illegible]
0.98	3.44	2.46	2.36533	1.50256	2	8	6	4	3.69274	1	16	15	[illegible]
0.98	3.44	2.46	2.68	1.37053	2	4	2	2.5	1.18818	1	16	15	[illegible]
0.98	2.55	1.57	1.965	0.82192	2	5	3	3.75	1.3784	1	16	15	[illegible]
0.98	2.2	1.22	1.3675	0.6314	2	6	4	3.25	2.07364	1	9	8	[illegible]
0.98	1.9	0.92	1.475	0.54122	2	8	6	4	2.96648	1	14	13	[illegible]
0.98	2.01	1.03	1.55	0.59012	2	8	6	4.5	2.75681	1	14	13	[illegible]
0.98	3.04	2.06	1.8	1.10316	1	2	1	1.75	0.54772	1	15	14	[illegible]
0.98	3.44	2.46	2.26833	1.50621	2	5	3	2.5	1.43019	1	16	15	[illegible]
0.98	3.44	2.46	1.68167	1.51158	2	6	4	2.33333	1.58565	1	16	15	[illegible]
1.1	1.61	0.51	1.4269	0.21902	3	8	5	3.17241	1.26131	2	13	11	[illegible]
0.98	1.9	0.92	1.2925	0.47526	2	3	1	2.5	0.63246	1	14	13	[illegible]
0.95	2.01	1.06	1.4875	0.66105	2	5	3	3.75	1.3784	1	14	13	[illegible]
0.98	2.55	1.57	2.08778	0.81323	2	4	2	3.11111	1.29099	1	16	15	[illegible]
0.98	3.44	2.46	2.6025	1.27092	2	2	0	2	0	1	16	15	[illegible]
0.98	2.55	1.57	1.9725	0.81938	2	4	2	3.5	1.09545	1	16	15	[illegible]
0.98	3.44	2.46	2.13556	1.53107	2	4	2	2.22222	0.8165	1	16	15	[illegible]
0.98	3.44	2.46	2.027	1.5336	2	3	1	2.1	0.39392	1	16	15	[illegible]
0.98	2.01	1.03	1.525	0.61582	2	8	6	4.5	2.75681	1	16	15	[illegible]
0.98	3.44	2.46	2.17556	1.51174	2	3	1	2.11111	0.40825	1	16	15	[illegible]
0.98	2.54	1.56	2.055	0.63202	2	6	4	5	1.58944	1	14	13	[illegible]
0.98	2.28	1.3	1.72	0.79394	1	5	4	1.8	1.53128	1	9	8	[illegible]
0.98	2.2	1.22	1.855	0.64433	1	2	1	1.5	0.63246	1	13	12	[illegible]
0.89	2.01	1.12	1.4725	0.68108	2	6	4	4	1.78885	1	14	13	[illegible]
0.98	3.44	2.46	2.322	1.5258	2	5	3	2.3	1.18176	1	16	15	[illegible]
0.98	3.04	2.06	1.78	1.33367	2	4	2	2.6	1.24722	1	17	16	[illegible]
0.98	3.44	2.46	1.93545	1.53751	2	4	2	2.18182	0.76696	1	16	15	[illegible]
0.98	2.55	1.57	1.9225	0.84225	2	4	2	3.25	1.04881	1	16	15	[illegible]
0.98	2.58	1.6	1.9375	0.86016	2	3	1	2.75	0.54772	1	16	15	[illegible]
0.98	3.44	2.46	2.22333	1.50723	2	6	4	2.44444	1.63299	1	16	15	[illegible]
0.98	3.44	2.46	2.265	1.48952	2	8	6	3.5	3.28634	1	16	15	[illegible]
0.98	2.2	1.22	1.75636	0.78286	1	6	5	1.81818	1.87083	1	8	7	[illegible]
0.98	2.2	1.22	1.90444	0.48555	2	5	3	4.66667	1.34164	1	14	13	[illegible]
1.28	2.02	0.74	1.65	0.52326	6	9	3	7.5	2.12132	8	15	7	[illegible]
1.12	1.31	0.19	1.215	0.13435	3	8	5	5.5	3.53553	2	4	2	[illegible]
1.24	1.31	0.07	1.275	0.0495	3	8	5	5.5	3.53553	2	14	12	[illegible]
[illegible]	[illegible]	[illegible]	[illegible]	[illegible]	[illegible]	[illegible]	[illegible]	[illegible]	[illegible]	[illegible]	[illegible]	[illegible]	[illegible]

1.2	1.31	0.11	1.255	0.07778	3	8	5	5.5	3.53553	2	10	8	6	5.65685	1	3
1.25	1.31	0.06	1.3	0.04243	3	8	5	3.83333	3.53553	2	15	13	4.16667	9.19239	1	3
1.31	2.55	1.24	1.51667	0.87681	3	4	1	3.16667	0.70711	2	16	14	4.33333	9.89949	1	2
1.24	1.31	0.07	1.2925	0.0495	3	8	5	4.25	3.53553	2	14	12	5	8.48528	1	3
1.1	1.31	0.21	1.205	0.14849	3	8	5	5.5	3.53553	2	11	9	6.5	6.36396	1	3
1.1	1.31	0.21	1.205	0.14849	3	8	5	5.5	3.53553	2	3	1	2.5	0.70711	1	3
1.31	2.55	1.24	1.62	0.87681	3	4	1	3.25	0.70711	2	16	14	5.5	9.89949	1	2
1.1	1.31	0.21	1.24	0.14849	3	8	5	4.66667	3.53553	2	16	14	6.66667	9.89949	1	3
1.1	1.31	0.21	1.24	0.14849	3	8	5	4.66667	3.53553	2	11	9	5	6.36396	1	3
1.31	2.19	0.88	1.45667	0.62225	3	3	0	3	0	2	15	13	4.16667	9.19239	1	2
1.22	1.31	0.09	1.28	0.06364	3	8	5	4.66667	3.53553	2	12	10	5.33333	7.07107	1	3
1.31	2.28	0.97	1.47167	0.68589	3	6	3	3.5	2.12132	2	10	8	3.33333	5.65685	1	3
1.25	1.31	0.06	1.28	0.04243	3	8	5	5.5	3.53553	2	15	13	8.5	9.19239	1	3
1.2	1.31	0.11	1.29167	0.07778	3	8	5	3.83333	3.53553	2	10	8	3.33333	5.65685	1	3
1.31	2.19	0.88	1.60333	0.62225	3	3	0	3	0	2	15	13	6.33333	9.19239	1	2
1.31	2.18	0.87	1.6	0.61518	3	4	1	3.33333	0.70711	2	15	13	6.33333	9.19239	1	2
1.1	1.31	0.21	1.2575	0.14849	3	8	5	4.25	3.53553	2	11	9	4.25	6.36396	1	3
1.2	1.31	0.11	1.255	0.07778	3	8	5	5.5	3.53553	2	10	8	6	5.65685	1	3
1.31	2.28	0.97	1.795	0.68589	3	6	3	4.5	2.12132	2	10	8	6	5.65685	1	3

[illegible]

1.31	2.19	0.88	1.53	0.62225	3	3	0	3	0	2	15	13	5.25	9.19239	1	2	1
1.1	1.31	0.21	1.205	0.14849	3	8	5	5.5	3.53553	2	16	14	9	9.89949	1	3	1
1.31	2.28	0.97	1.63333	0.68589	3	6	3	4	2.12132	2	10	8	4.66667	5.65685	1	3	[illegible]
1	1.31	0.31	1.20667	0.2192	3	4	1	3.33333	0.70711	2	2	0	2	0	1	1	[illegible]
1.13	1.31	0.18	1.265	0.12728	3	8	5	4.25	3.53553	2	7	5	3.25	3.53553	1	3	[illegible]
1.31	2.28	0.97	1.63333	0.68589	3	6	3	4	2.12132	2	10	8	4.66667	5.65685	1	3	[illegible]
1.22	1.31	0.09	1.28	0.06364	3	5	2	3.66667	1.41421	2	3	1	2.33333	0.70711	1	3	[illegible]
1.31	2.55	1.24	1.72333	0.87681	3	4	1	3.33333	0.70711	2	16	14	6.66667	9.89949	1	2	[illegible]
1.22	1.31	0.09	1.2875	0.06364	3	5	2	3.5	1.41421	2	3	1	2.25	0.70711	1	3	[illegible]
1.23	1.31	0.08	1.27	0.05657	3	8	5	5.5	3.53553	2	13	11	7.5	7.77817	1	3	[illegible]
1.22	1.31	0.09	1.28	0.06364	3	8	5	4.66667	3.53553	2	12	10	5.33333	7.07107	1	3	[illegible]
1.1	1.31	0.21	1.275	0.14849	3	8	5	3.83333	3.53553	2	16	14	4.33333	9.89949	1	3	[illegible]
1.13	1.31	0.18	1.25	0.12728	3	8	5	4.66667	3.53553	2	5	3	3	2.12132	1	3	[illegible]
1.31	2.19	0.88	1.60333	0.62225	3	3	0	3	0	2	15	13	6.33333	9.19239	1	2	[illegible]
1.2	1.31	0.11	1.27333	0.07778	3	8	5	4.66667	3.53553	2	10	8	4.66667	5.65685	1	3	[illegible]
1.12	1.31	0.19	1.215	0.13435	3	8	5	5.5	3.53553	2	4	2	3	1.41421	1	3	[illegible]
1.31	2.18	0.87	1.6	0.61518	3	4	1	3.33333	0.70711	2	15	13	6.33333	9.19239	1	2	[illegible]
1.31	2.28	0.97	1.795	0.68589	3	6	3	4.5	2.12132	2	10	8	6	5.65685	1	3	[illegible]
1.12	1.31	0.19	1.24667	0.13435	3	8	5	4.66667	3.53553	2	4	2	2.66667	1.41421	1	3	[illegible]
1.1	1.31	0.21	1.275	0.14849	3	8	5	3.83333	3.53553	2	16	14	4.33333	9.89949	1	3	[illegible]
1.31	2.55	1.24	1.93	0.87681	3	4	1	3.5	0.70711	2	16	14	9	9.89949	1	2	[illegible]
1	1.31	0.31	1.25833	0.2192	3	4	1	3.16667	0.70711	2	2	0	2	0	1	1	[illegible]
1.31	2.55	1.24	1.93	0.87681	3	4	1	3.5	0.70711	2	16	14	9	9.89949	1	2	[illegible]

1.17	1.31	0.14	1.26333	0.09899	3	8	5	4.66667	3.53553	2	8	6	4	4.24264	1	3
1.31	2.55	1.24	1.72333	0.87681	3	4	1	3.33333	0.70711	2	16	14	6.66667	9.89949	1	2
1	1.31	0.31	1.20667	0.2192	3	4	1	3.33333	0.70711	2	2	0	2	0	1	1
1.1	1.31	0.21	1.275	0.14849	3	9	6	4	4.24264	2	3	1	2.16667	0.70711	1	3
1.13	1.31	0.18	1.25	0.12728	3	8	5	4.66667	3.53553	2	7	5	3.66667	3.53553	1	3
1.22	1.31	0.09	1.28	0.06364	3	5	2	3.66667	1.41421	2	3	1	2.33333	0.70711	1	3
1.1	1.31	0.21	1.275	0.14849	3	8	5	3.83333	3.53553	2	3	1	2.16667	0.70711	1	3
1.1	1.31	0.21	1.24	0.14849	3	8	5	4.66667	3.53553	2	11	9	5	6.36396	1	3
1.2	1.31	0.11	1.27333	0.07778	3	8	5	4.66667	3.53553	2	10	8	4.66667	5.65685	1	3
1.1	1.31	0.21	1.205	0.14849	3	8	5	5.5	3.53553	2	3	1	2.5	0.70711	1	3
1	1.31	0.31	1.20667	0.2192	3	4	1	3.33333	0.70711	2	2	0	2	0	1	1
1.31	2.28	0.97	1.63333	0.68589	3	6	3	4	2.12132	2	10	8	4.66667	5.65685	1	3
0.89	2.2	1.31	1.91667	0.67984	1	6	5	2.33333	2.78887	1	13	12	3.16667	6.24055	1	2
1.31	2.55	1.24	1.93	0.87681	3	4	1	3.5	0.70711	2	16	14	9	9.89949	1	2
1	1.31	0.31	1.20667	0.2192	3	4	1	3.33333	0.70711	2	2	0	2	0	1	1
0.82	1.31	0.49	1.14667	0.34648	3	5	2	3.66667	1.41421	1	2	1	1.66667	0.70711	1	1
1.31	2.28	0.97	1.47167	0.68589	3	6	3	3.5	2.12132	2	10	8	3.33333	5.65685	1	3
1.1	2.04	0.94	1.81167	0.50189	2	8	6	3.16667	3.10018	13	16	3	13.5	1.58114	2	3

1.31	2.28	0.97	1.795	0.68589	3	6	3	4.5	2.12132	2	10	8	6	5.65685	1	3	2	2	1.41421	24.305	195.084	170.779
1.2	1.31	0.11	1.255	0.07778	3	8	5	5.5	3.53553	2	10	8	6	5.65685	1	3	2	2	1.41421	24.305	157.25	132.945
1.17	1.31	0.14	1.24	0.09899	3	8	5	5.5	3.53553	2	8	6	5	4.24264	1	3	2	2	1.41421	24.305	150.36	126.055
1.24	1.31	0.07	1.28667	0.0495	3	8	5	4.66667	3.53553	2	14	12	6	8.48528	1	3	2	1.67	1.41421	24.305	167.259	142.954
1.31	2.19	0.88	1.75	0.62225	3	3	0	3	0	2	15	13	8.5	9.19239	1	2	1	1.5	0.70711	24.305	30.9738	6.66876
1.24	1.31	0.07	1.275	0.0495	3	8	5	5.5	3.53553	2	14	12	8	8.48528	1	3	2	2	1.41421	24.305	167.259	142.954
1.31	2.28	0.97	1.63333	0.68589	3	6	3	4	2.12132	2	10	8	4.66667	5.65685	1	3	2	1.67	1.41421	24.305	195.084	170.779
1.31	2.28	0.97	1.795	0.68589	3	6	3	4.5	2.12132	2	10	8	6	5.65685	1	3	2	2	1.41421	24.305	195.084	170.779
1.1	1.31	0.21	1.24	0.14849	3	8	5	4.66667	3.53553	2	11	9	5	6.36396	1	3	2	1.67	1.41421	24.305	158.925	134.62
1.13	1.31	0.18	1.25	0.12728	3	8	5	4.66667	3.53553	2	5	3	3	2.12132	1	3	2	1.67	1.41421	24.305	140.908	116.603
1.31	2.01	0.7	1.54333	0.49497	3	4	1	3.33333	0.70711	2	14	12	6	8.48528	1	2	1	1.33	0.70711	24.305	72.64	48.335
1.1	1.31	0.21	1.205	0.14849	3	8	5	5.5	3.53553	2	3	1	2.5	0.70711	1	3	2	2	1.41421	24.305	138.905	114.6
1.31	2.58	1.27	1.945	0.89803	3	3	0	3	0	2	16	14	9	9.89949	1	2	1	1.5	0.70711	24.305	32.065	7.76
1.17	1.31	0.14	1.26333	0.09899	3	8	5	4.66667	3.53553	2	8	6	4	4.24264	1	3	2	1.67	1.41421	24.305	150.36	126.055
1.31	2.28	0.97	1.795	0.68589	3	6	3	4.5	2.12132	2	10	8	6	5.65685	1	3	2	2	1.41421	24.305	195.084	170.779
1.14	1.31	0.17	1.25333	0.12021	3	8	5	4.66667	3.53553	2	6	4	3.33333	2.82843	1	3	2	1.67	1.41421	24.305	144.242	119.937
1.13	1.31	0.18	1.25	0.12728	3	8	5	4.66667	3.53553	2	7	5	3.66667	3.53553	1	3	2	1.67	1.41421	24.305	145	120.695
1.27	3.44	2.17	2.61833	0.93788	1	8	7	2.83333	2.83522	1	17	16	11.3333	8.61126	1	3	2	1.83	0.80861	1.00794	174.967	173.959
2.2	3.04	0.84	2.73667	0.41574	1	2	1	1.83333	0.47673	1	15	14	12.3333	6.50874	1	2	1	1.83	0.47673	1.00794	14.0067	12.9988
0.82	2.2	1.38	1.94333	0.71525	1	4	3	1.66667	1.56347	1	13	12	3	6.32456	1	2	1	1.17	0.52705	1.00794	39.0983	38.0904
1.93	3.04	1.11	2.42167	0.51509	1	5	4	2.33333	1.95789	1	17	16	7.66667	8.44591	1	3	2	1.67	0.91287	1.00794	107.868	106.86
1.31	2.2	0.89	1.90333	0.62933	1	3	2	1.66667	1.41421	1	2	1	1.33333	0.70711	1	1	0	1	0	1.00794	24.305	23.2971
0.95	3.04	2.09	2.06333	1.05168	1	5	4	2.66667	2.08167	1	15	14	6	7.81025	1	2	1	1.33	0.57735	1.00794	87.62	86.6121
1	2.2	1.2	1.56	0.72363	1	5	4	3.16667	2.01133	1	14	13	3.66667	5.93908	1	2	1	1.17	0.47673	1.00794	118.71	117.702
1	2.33	1.33	1.62167	0.79717	1	6	5	3.33333	2.29624	1	14	13	3.66667	5.93908	1	2	1	1.17	0.47673	1.00794	207.2	206.192
0.93	3.44	2.51	2.19	1.25503	1	3	2	2	1	1	16	15	6	8.66025	1	2	1	1.33	0.57735	1.00794	22.9898	21.9818
0.79	3.44	2.65	2.52917	1.09592	1	6	5	2.08333	1.5843	1	16	15	8.5	9	1	2	1	1.5	0.6	1.00794	132.905	131.898
0.79	2.58	1.79	1.85667	0.9431	1	6	5	3.33333	2.51661	1	16	15	6	8.66025	1	2	1	1.33	0.57735	1.00794	132.905	131.898
0.79	3.44	2.65	2.14333	1.32591	1	6	5	3	2.64575	1	16	15	6	8.66025	1	2	1	1.33	0.57735	1.00794	132.905	131.898
0.98	1.93	0.95	1.5625	0.55716	2	5	3	3.125	1.43925	1	14	13	8.375	7.19623	1	3	2	1.88	0.96362	6.941	107.868	100.927
1.13	1.31	0.18	1.22	0.12728	3	8	5	5.5	3.53553	2	7	5	4.5	3.53553	1	3	2	2	1.41421	24.305	145	120.695
0.98	3.44	2.46	2.34375	1.48486	2	5	3	2.375	1.28759	1	16	15	10.25	9.30761	1	2	1	1.63	0.62828	6.941	121.76	114.819
0.98	3.44	2.46	2.265	1.48952	2	8	6	3.5	3.28634	1	16	15	10.75	7.76531	1	3	2	2	0.89443	6.941	157.25	150.309
0.98	3.44	2.46	2.68	1.37053	2	4	2	2.5	1.18818	1	16	15	11.375	8.34372	1	3	2	2.13	0.82248	6.941	50.9415	44.0005
0.98	2.01	1.03	1.60375	0.59764	2	5	3	3.5	1.51186	1	14	13	8.375	7.19623	1	3	2	1.88	0.96362	6.941	107.868	100.927
1.13	1.31	0.18	1.22	0.12728	3	8	5	5.5	3.53553	2	5	3	3.5	2.12132	1	3	2	2	1.41421	24.305	140.908	116.603
1.31	2.28	0.97	1.47167	0.68589	3	6	3	3.5	2.12132	2	10	8	3.33333	5.65685	1	3	2	1.33	1.41421	24.305	195.084	170.779
1.2	1.31	0.11	1.255	0.07778	3	8	5	5.5	3.53553	2	10	8	6	5.65685	1	3	2	2	1.41421	24.305	157.25	132.945
0.98	2.01	1.03	1.5325	0.60771	2	8	6	4.5	2.75681	1	14	13	8.5	7.18331	1	3	2	2	0.89443	6.941	140.908	133.967
1.31	1.93	0.62	1.45552	0.23674	3	5	2	3.06897	0.50452	2	13	11	6.86207	7.47359	1	3	2	1.48	0.78044	24.305	107.868	83.5632
1.23	1.31	0.08	1.27	0.05657	3	8	5	5.5	3.53553	2	13	11	7.5	7.77817	1	3	2	2	1.41421	24.305	164.93	140.625
0.98	2.01	1.03	1.5425	0.59742	2	8	6	4.5	2.75681	1	14	13	9.25	6.77495	1	3	2	2	0.89443	6.941	150.36	143.419
1.31	2.01	0.7	1.54333	0.49497	3	4	1	3.33333	0.70711	2	14	12	6	8.48528	1	2	1	1.33	0.70711	24.305	72.64	48.335
0.79	2.96	2.17	1.94625	1.31778	2	6	4	3.5	1.71679	1	17	16	9	10.3822	1	2	1	1.5	0.64889	6.941	132.905	125.964
0.98	2.55	1.57	1.97125	0.64352	2	2	0	2	0	1	14	13	11.625	6.32151	1	2	1	1.88	0.51887	6.941	12.0107	5.0697
0.98	3.44	2.46	2.3675	1.38556	2	3	1	2.25	0.54772	1	16	15	11.5	7.82304	1	2	1	1.75	0.54772	6.941	26.9815	20.0405
1.17	1.31	0.14	1.24	0.09899	3	8	5	5.5	3.53553	2	8	6	5	4.24264	1	3	2	2	1.41421	24.305	150.36	126.055
0.98	2.01	1.03	1.535	0.60508	2	8	6	4.5	2.75681	1	14	13	8.75	7.00714	1	3	2	2	0.89443	6.941	144.242	137.301
1.2	1.31	0.11	1.27333	0.07778	3	8	5	4.66667	3.53553	2	9	7	4.33333	4.94975	1	3	2	1.67	1.41421	24.305	151.964	127.659
0.98	2.58	1.6	2.10444	0.82929	2	3	1	2.55556	0.6455	1	16	15	12	7.79423	1	2	1	1.78	0.54006	6.941	32.065	25.124
0.98	3.44	2.46	2.18778	1.50865	2	4	2	2.22222	0.8165	1	16	15	9.11111	9.45604	1	2	1	1.56	0.6455	6.941	72.64	65.699
1.31	3.98	2.67	1.52621	0.67208	2	3	1	2.96552	0.25226	2	17	15	7.06897	7.83698	1	2	1	1.45	0.68755	18.9984	26.9815	7.98314
1.31	2.19	0.88	1.60333	0.62225	3	3	0	3	0	2	15	13	6.33333	9.19239	1	2	1	1.33	0.70711	24.305	30.9738	6.66876
1.31	2.58	1.27	1.73333	0.89803	3	3	0	3	0	2	16	14	6.66667	9.89949	1	2	1	1.33	0.70711	24.305	32.065	7.76
1.13	1.31	0.18	1.25	0.12728	3	8	5	4.66667	3.53553	2	5	3	3	2.12132	1	3	2	1.67	1.41421	24.305	140.908	116.603
1.22	1.31	0.09	1.28	0.06364	3	5	2	3.66667	1.41421	2	3	1	2.33333	0.70711	1	3	2	1.67	1.41421	24.305	88.9059	64.6009
1.24	1.31	0.07	1.28667	0.0495	3	8	5	4.66667	3.53553	2	14	12	6	8.48528	1	3	2	1.67	1.41421	24.305	167.259	142.954
1.31	2.2	0.89	1.90333	0.62933	1	3	2	1.66667	1.41421	1	2	1	1.33333	0.70711	1	1	0	1	0	1.00794	24.305	23.2971
1.31	2.2	0.89	1.90333	0.62933	1	3	2	1.66667	1.41421	1	2	1	1.33333	0.70711	1	1	0	1	0	1.00794	24.305	23.2971
1.13	1.31	0.18	1.25	0.12728	3	8	5	4.66667	3.53553	2	5	3	3	2.12132	1	3	2	1.67	1.41421	24.305	140.908	116.603
0.98	3.98	3	3.01889	1.93894	2	5	3	2.33333	1.34164	1	17	16	12	10.1292	1	3	2	1.89	0.80623	6.941	91.224	84.283
0.95	1.31	0.36	1.13	0.25456	3	5	2	4	1.41421	2	2	0	2	0	1	1	0	1	0	24.305	87.62	63.315
1.2	1.31	0.11	1.27333	0.07778	3	8	5	4.66667	3.53553	2	9	7	4.33333	4.94975	1	3	2	1.67	1.41421	24.305	151.964	127.659
1.14	1.31	0.17	1.25333	0.12021	3	8	5	4.66667	3.53553	2	6	4	3.33333	2.82843	1	3	2	1.67	1.41421	24.305	144.242	119.937
1	2.2	1.2	1.935	0.6247	1	4	3	2	2	1	13	12	3.16667	6.24055	1	2	1	1.17	0.52705	1.00794	69.723	68.7151
1.22	1.31	0.09	1.265	0.06364	3	5	2	4	1.41421	2	3	1	2.5	0.70711	1	3	2	2	1.41421	24.305	88.9059	64.6009
0.79	2.96	2.17	1.89875	1.37933	2	6	4	4.5	1.71679	1	17	16	9	10.3822	1	2	1	1.5	0.64889	6.941	132.905	125.964
1.22	1.31	0.09	1.295	0.06364	3	8	5	3.83333	3.53553	2	12	10	3.66667	7.07107	1	3	2	1.33	1.41421	24.305	162.5	138.195
1.12	1.31	0.19	1.27833	0.13435	3	8	5	3.83333	3.53553	2	4	2	2.33333	1.41421	1	3	2	1.33	1.41421	24.305	140.116	115.811
1.31	2.28	0.97	1.795	0.68589	3	6	3	4.5	2.12132	2	10	8	6	5.65685	1	3	2	2	1.41421	24.305	195.084	170.779
1.1	1.31	0.21	1.205	0.14849	3	9	6	6	4.24264	2	3	1	2.5	0.70711	1	3	2	2	1.41421	24.305	227	202.695
1.24	1.31	0.07	1.275	0.0495	3	8	5	5.5	3.53553	2	14	12	8	8.48528	1	3	2	2	1.41421	24.305	167.259	142.954
0.79	2.2	1.41	1.9	0.73034	1	6	5	2.33333	2.78887	1	13	12	3	6.32456	1	2	1	1.17	0.52705	1.00794	132.905	131.898
1.22	1.31	0.09	1.28	0.06364	3	8	5	4.66667	3.53553	2	12	10	5.33333	7.07107	1	3	2	1.67	1.41421	24.305	162.5	138.195
1.13	1.31	0.18	1.25	0.12728	3	8	5	4.66667	3.53553	2	7	5	3.66667	3.53553	1	3	2	1.67	1.41421	24.305	145	120.695
1.22	1.31	0.09	1.265	0.06364	3	5	2	4	1.41421	2	3	1	2.5	0.70711	1	3	2	2	1.41421	24.305	88.9059	64.6009
1.17	1.31	0.14	1.26333	0.09899	3	8	5	4.66667	3.53553	2	8	6	4	4.24264	1	3	2	1.67	1.41421	24.305	150.36	126.055
0.79	3.44	2.65	2.37833	1.15276	1	6	5	2.16667	2.26635	1	16	15	6	9.04534	1	2	1	1.33	0.60302	1.00794	132.905	131.898
1.31	3.44	2.13	2.49417	1.07343	1	3	2	1.91667	0.93967	1	16	15	7.5	8.90374	1	2	1	1.42	0.6102	1.00794	24.305	23.2971
1.1	1.31	0.21	1.275	0.14849	3	8	5	3.83333	3.53553	2	3	1	2.16667	0.70711	1	3	2	1.33	1.41421	24.305	138.905	114.6
0.89	2.2	1.31	1.91167	0.62897	1	6	5	2	2.20564	1	8	7	1.83333	2.66103	1	3	2	1.17	0.7711	1.00794	137.327	136.319
1.22	1.31	0.09	1.28	0.06364	3	8	5	4.66667	3.53553	2	12	10	5.33333	7.07107	1	3	2	1.67	1.41421	24.305	162.5	138.195
1.22	1.31	0.09	1.28	0.06364	3	5	2	3.66667	1.41421	2	3	1	2.33333	0.70711	1	3	2	1.67	1.41421	24.305	88.9059	64.6009
1.2	1.31	0.11	1.29167	0.07778	3	8	5	3.83333	3.53553	2	9	7	3.16667	4.94975	1	3	2	1.33	1.41421	24.305	151.964	127.659
1.25	1.31	0.06	1.28	0.04243	3	8	5	5.5	3.53553	2	15	13	8.5	9.19239	1	3	2	2	1.41421	24.305	168.934	144.629
1.17	1.31	0.14	1.24	0.09899	3	8	5	5.5	3.53553	2	8	6	5	4.24264	1	3	2	2	1.41421	24.305	150.36	126.055
1.31	2.28	0.97	1.63333	0.68589	3	6	3	4	2.12132	2	10	8	4.66667	5.65685	1	3	2	1.67	1.41421	24.305	195.084	170.779
1.31	2.2	0.89	1.90333	0.62933	1	3	2	1.66667	1.41421	1	2	1	1.33333	0.70711	1	1	0	1	0	1.00794	24.305	23.2971
1.31	2.2	0.89	1.96083	0.49208	1	4	3	1.91667	1.8936	1	4	3	1.83333	1.83615	1	3	2	1.5	1.24212	1.00794	47.867	46.8591
1.31	2.2	0.89	1.9225	0.56888	1	4	3	1.75	1.56296	1	4	3	1.5	1.24212	1	3	2	1.17	0.79282	1.00794	47.867	46.8591
0.79	2.66	1.87	1.74875	1.185	2	6	4	5	1.58944	1	17	16	9	10.3822	1	2	1	1.5	0.64889	6.941	132.905	125.964
1.12	1.31	0.19	1.24667	0.13435	3	8	5	4.66667	3.53553	2	4	2	2.66667	1.41421	1	3	2	1.67	1.41421	24.305	140.116	115.811
0.98	3.44	2.46	2.332	1.2063	2	4	2	2.8	1.22474	1	16	15	11	6.84653	1	3	2	2.2	0.93541	6.941	63.546	56.605
1.1	1.31	0.21	1.24	0.14849	3	8	5	4.66667	3.53553	2	11	9	5	6.36396	1	3	2	1.67	1.41421	24.305	158.925	134.62
1.2	1.31	0.11	1.255	0.07778	3	8	5	5.5	3.53553	2	9	7	5.5	4.94975	1	3	2	2	1.41421	24.305	151.964	127.659
0.82	1.31	0.49	1.065	0.34648	3	5	2	4	1.41421	1	2	1	1.5	0.70711	1	1	0	1	0	24.305	85.4678	61.1628
1.22	3.44	2.22	2.77769	0.84186	1	5	4	2	1.1698	1	16	15	11.0769	7.85393	1	3	2	1.85	0.64889	1.00794	88.9059	87.8979
0.98	3.44	2.46	2.24778	1.51282	2	2	0	2	0	1	16	15	9.11111	9.45604	1	2	1	1.56	0.6455	6.941	15.9994	9.0584
1.31	3.44	2.13	2.43	1.24992	1	3	2	2.23077	0.88795	1	16	15	8.30769	9.08824	1	2	1	1.46	0.63549	1.00794	24.305	23.2971
1.1	1.31	0.21	1.24	0.14849	3	8	5	4.66667	3.53553	2	3	1	2.33333	0.70711	1	3	2	1.67	1.41421	24.305	138.905	114.6
0.98	3.04	2.06	1.896	1.27511	2	6	4	2.4	1.57568	1	15	14	7.2	8.66622	1	3	2	1.6	0.87099	6.941	186.207	179.266
0.98	3.44	2.46	2.792	1.27622	2	5	3	2.6	1.60357	1	17	16	13.2	8.16788	1	2	1	1.8	0.53452	6.941	126.904	119.963
0.98	3.44	2.46	2.328	1.52448	2	5	3	2.3	1.18176	1	16	15	9	9.37715	1	3	2	1.7	0.84077	6.941	95.94	88.999
1.2	1.31	0.11	1.29167	0.07778	3	8	5	3.83333	3.53553	2	10	8	3.33333	5.65685	1	3	2	1.33	1.41421	24.305	157.25	132.945
1.13	1.31	0.18	1.22	0.12728	3	8	5	5.5	3.53553	2	7	5	4.5	3.53553	1	3	2	2	1.41421	24.305	145	120.695
0.98	3.44	2.46	2.586	1.42442	2	4	2	2.4	1.06904	1	16	15	10.8	8.67673	1	3	2	2	0.84515	6.941	50.9415	44.0005
1.13	1.31	0.18	1.22	0.12728	3	8	5	5.5	3.53553	2	7	5	4.5	3.53553	1	3	2	2	1.41421	24.305	145	120.695
1.1	1.31	0.21	1.24	0.14849	3	9	6	5	4.24264	2	3	1	2.33333	0.70711	1	3	2	1.67	1.41421	24.305	227	202.695
1.13	1.31	0.18	1.22	0.12728	3	8	5	5.5	3.53553	2	7	5	4.5	3.53553	1	3	2	2	1.41421	24.305	145	120.695
0.98	3.44	2.46	2.24778	1.51282	2	2	0	2	0	1	16	15	9.11111	9.45604	1	2	1	1.56	0.6455	6.941	15.9994	9.0584
0.98	3.04	2.06	1.83636	1.2908	2	5	3	2.27273	1.15045	1	15	14	6.54545	8.72623	1	3	2	1.55	0.87447	6.941	95.94	88.999
0.98	3.44	2.46	2.67	1.33887	2	5	3	2.6	1.60357	1	16	15	12.8	7.90118	1	2	1	1.8	0.53452	6.941	121.76	114.819
1.13	1.31	0.18	1.22	0.12728	3	8	5	5.5	3.53553	2	5	3	3.5	2.12132	1	3	2	2	1.41421	24.305	140.908	116.603
0.95	1.31	0.36	1.13	0.25456	3	5	2	4	1.41421	2	2	0	2	0	1	1	0	1	0	24.305	87.62	63.315
0.98	3.04	2.06	1.49727	1.3016	2	4	2	2.18182	0.87706	1	16	15	4.90909	9.74482	1	2	1	1.27	0.67937	6.941	78.96	72.019
1.25	1.31	0.06	1.29	0.04243	3	8	5	4.66667	3.53553	2	15	13	6.33333	9.19239	1	3	2	1.67	1.41421	24.305	168.934	144.629
0.98	3.04	2.06	1.85455	1.30114	2	6	4	2.36364	1.53393	1	15	14	6.54545	8.72623	1	3	2	1.55	0.87447	6.941	183.84	176.899
0.98	3.44	2.46	2.61091	1.35967	2	4	2	2.54545	1.19257	1	16	15	10.9545	8.27782	1	3	2	2.14	0.83666	6.941	50.9415	44.0005
1.14	1.31	0.17	1.225	0.12021	3	8	5	5.5	3.53553	2	6	4	4	2.82843	1	3	2	2	1.41421	24.305	144.242	119.937
0.82	1.31	0.49	1.065	0.34648	3	5	2	4	1.41421	1	2	1	1.5	0.70711	1	1	0	1	0	24.305	85.4678	61.1628
1.22	1.31	0.09	1.28	0.06364	3	5	2	3.66667	1.41421	2	3	1	2.33333	0.70711	1	3	2	1.67	1.41421	24.305	88.9059	64.6009
1.13	1.31	0.18	1.25	0.12728	3	8	5	4.66667	3.53553	2	7	5	3.66667	3.53553	1	3	2	1.67	1.41421	24.305	145	120.695
1.17	1.31	0.14	1.24	0.09899	3	8	5	5.5	3.53553	2	8	6	5	4.24264	1	3	2	2	1.41421	24.305	150.36	126.055
1.17	1.31	0.14	1.28667	0.09899	3	8	5	3.83333	3.53553	2	8	6	3	4.24264	1	3	2	1.33	1.41421	24.305	150.36	126.055
0.98	2.2	1.22	1.775	0.74623	1	3	2	1.5	0.91287	1	13	12	2.2	4.89898	1	2	1	1.1	0.40825	1.00794	26.9815	25.9736
1.2	1.31	0.11	1.27333	0.07778	3	8	5	4.66667	3.53553	2	9	7	4.33333	4.94975	1	3	2	1.67	1.41421	24.305	151.964	127.659
1.22	1.31	0.09	1.265	0.06364	3	5	2	4	1.41421	2	3	1	2.5	0.70711	1	3	2	2	1.41421	24.305	88.9059	64.6009
0.82	1.31	0.49	1.14667	0.34648	3	5	2	3.66667	1.41421	1	2	1	1.66667	0.70711	1	1	0	1	0	24.305	85.4678	61.1628
0.95	1.31	0.36	1.19	0.25456	3	5	2	3.66667	1.41421	2	2	0	2	0	1	1	0	1	0	24.305	87.62	63.315
0.82	1.31	0.49	1.14667	0.34648	3	5	2	3.66667	1.41421	1	2	1	1.66667	0.70711	1	1	0	1	0	24.305	85.4678	61.1628
1	1.31	0.31	1.155	0.2192	3	4	1	3.5	0.70711	2	2	0	2	0	1	1	0	1	0	24.305	40.078	15.773
1.31	2.18	0.87	1.745	0.61518	3	4	1	3.5	0.70711	2	15	13	8.5	9.19239	1	2	1	1.5	0.70711	24.305	74.9216	50.6166
1.23	1.31	0.08	1.28333	0.05657	3	8	5	4.66667	3.53553	2	13	11	5.66667	7.77817	1	3	2	1.67	1.41421	24.305	164.93	140.625
1	1.31	0.31	1.155	0.2192	3	4	1	3.5	0.70711	2	2	0	2	0	1	1	0	1	0	24.305	40.078	15.773
1.31	2.28	0.97	1.63333	0.68589	3	6	3	4	2.12132	2	10	8	4.66667	5.65685	1	3	2	1.67	1.41421	24.305	195.084	170.779
1.13	1.31	0.18	1.25	0.12728	3	8	5	4.66667	3.53553	2	5	3	3	2.12132	1	3	2	1.67	1.41421	24.305	140.908	116.603
1.31	2.19	0.88	1.75	0.62225	3	3	0	3	0	2	15	13	8.5	9.19239	1	2	1	1.5	0.70711	24.305	30.9738	6.66876
0.79	2.66	1.87	1.79625	1.12349	2	6	4	4	2.05196	1	17	16	9	10.3822	1	2	1	1.5	0.64889	6.941	132.905	125.964
1.1	1.31	0.21	1.205	0.14849	3	8	5	5.5	3.53553	2	11	9	6.5	6.36396	1	3	2	2	1.41421	24.305	158.925	134.62
0.82	1.31	0.49	1.14667	0.34648	3	5	2	3.66667	1.41421	1	2	1	1.66667	0.70711	1	1	0	1	0	24.305	85.4678	61.1628
1.1	1.31	0.21	1.24	0.14849	3	9	6	5	4.24264	2	3	1	2.33333	0.70711	1	3	2	1.67	1.41421	24.305	227	202.695
1.31	2.58	1.27	1.945	0.89803	3	3	0	3	0	2	16	14	9	9.89949	1	2	1	1.5	0.70711	24.305	32.065	7.76
1.13	1.31	0.18	1.28	0.12728	3	8	5	3.83333	3.53553	2	5	3	2.5	2.12132	1	3	2	1.33	1.41421	24.305	140.908	116.603
1.1	1.31	0.21	1.24	0.14849	3	9	6	5	4.24264	2	3	1	2.33333	0.70711	1	3	2	1.67	1.41421	24.305	227	202.695
1.31	2.58	1.27	1.73333	0.89803	3	3	0	3	0	2	16	14	6.66667	9.89949	1	2	1	1.33	0.70711	24.305	32.065	7.76
1	1.31	0.31	1.20667	0.2192	3	4	1	3.33333	0.70711	2	2	0	2	0	1	1	0	1	0	24.305	40.078	15.773
1.1	1.31	0.21	1.24	0.14849	3	9	6	5	4.24264	2	3	1	2.33333	0.70711	1	3	2	1.67	1.41421	24.305	227	202.695
1.1	1.31	0.21	1.275	0.14849	3	8	5	3.83333	3.53553	2	3	1	2.16667	0.70711	1	3	2	1.33	1.41421	24.305	138.905	114.6
1.2	1.31	0.11	1.255	0.07778	3	8	5	5.5	3.53553	2	9	7	5.5	4.94975	1	3	2	2	1.41421	24.305	151.964	127.659
0.98	3.44	2.46	2.24778	1.51282	2	2	0	2	0	1	16	15	9.11111	9.45604	1	2	1	1.56	0.6455	6.941	15.9994	9.0584
1.12	1.31	0.19	1.215	0.13435	3	8	5	5.5	3.53553	2	4	2	3	1.41421	1	3	2	2	1.41421	24.305	140.116	115.811
1.13	1.31	0.18	1.28	0.12728	3	8	5	3.83333	3.53553	2	7	5	2.83333	3.53553	1	3	2	1.33	1.41421	24.305	145	120.695
1.13	1.31	0.18	1.25	0.12728	3	8	5	4.66667	3.53553	2	5	3	3	2.12132	1	3	2	1.67	1.41421	24.305	140.908	116.603
0.98	3.44	2.46	2.696	1.31903	2	4	2	2.4	1.06904	1	16	15	12.8	7.90118	1	2	1	1.8	0.53452	6.941	74.9216	67.9806
1.23	1.31	0.08	1.28333	0.05657	3	8	5	4.66667	3.53553	2	13	11	5.66667	7.77817	1	3	2	1.67	1.41421	24.305	164.93	140.625
1.1	1.31	0.21	1.205	0.14849	3	8	5	5.5	3.53553	2	3	1	2.5	0.70711	1	3	2	2	1.41421	24.305	138.905	114.6
1.25	1.31	0.06	1.28	0.04243	3	8	5	5.5	3.53553	2	15	13	8.5	9.19239	1	3	2	2	1.41421	24.305	168.934	144.629
0.98	2.2	1.22	1.76667	0.71524	2	5	3	3.5	1.91881	1	13	12	7.5	6.03399	1	3	2	2.17	1.14812	6.941	106.42	99.479
0.98	2.58	1.6	2.0475	0.82624	2	5	3	3.25	1.3784	1	16	15	12	8.04984	1	2	1	1.75	0.54772	6.941	121.76	114.819
1.14	1.31	0.17	1.25333	0.12021	3	8	5	4.66667	3.53553	2	6	4	3.33333	2.82843	1	3	2	1.67	1.41421	24.305	144.242	119.937
1.54	2.54	1	1.96571	0.49218	1	6	5	3	2.36643	1	11	10	3.71429	4.19524	1	3	2	2.14	1.26491	1.00794	196.967	195.959
1.22	1.31	0.09	1.28	0.06364	3	5	2	3.66667	1.41421	2	3	1	2.33333	0.70711	1	3	2	1.67	1.41421	24.305	88.9059	64.6009

0.98	2.58	1.6	1.92	0.83594	1	3	2	2	1	1	16	15	6	8.66025	1	2
0.98	2.28	1.3	1.80667	0.75559	2	6	4	4	2.55841	1	13	12	7.5	6.03399	1	3
1.13	1.31	0.18	1.25	0.12728	3	8	5	4.66667	3.53553	2	7	5	3.66667	3.53553	1	3
1.33	2.2	0.87	1.88714	0.48553	1	5	4	2.42857	2.22004	1	4	3	2.28571	1.96396	1	3
1.31	2.01	0.7	1.66	0.49497	3	4	1	3.5	0.70711	2	14	12	8	8.48528	1	2
1.2	1.31	0.11	1.27333	0.07778	3	8	5	4.66667	3.53553	2	9	7	4.33333	4.94975	1	3
1.12	1.31	0.19	1.24667	0.13435	3	8	5	4.66667	3.53553	2	4	2	2.66667	1.41421	1	3
1.22	1.31	0.09	1.265	0.06364	3	5	2	4	1.41421	2	3	1	2.5	0.70711	1	3
1.1	1.31	0.21	1.24	0.14849	3	8	5	4.66667	3.53553	2	11	9	5	6.36396	1	3
0.79	2.2	1.41	1.93	0.60228	1	6	5	2.14286	2.09762	1	13	12	6.14286	7.58947	1	2
1.1	1.31	0.21	1.2575	0.14849	3	9	6	4.5	4.24264	2	3	1	2.25	0.70711	1	3
1.31	2.01	0.7	1.54333	0.49497	3	4	1	3.33333	0.70711	2	14	12	6	8.48528	1	2
0.95	1.31	0.36	1.19	0.25456	3	5	2	3.66667	1.41421	2	2	0	2	0	1	1
1.14	1.31	0.17	1.28167	0.12021	3	8	5	3.83333	3.53553	2	6	4	2.66667	2.82843	1	3
0.95	1.31	0.36	1.13	0.25456	3	5	2	4	1.41421	2	2	0	2	0	1	1
0.79	2.2	1.41	1.79714	0.84264	1	6	5	3	3.08221	1	10	9	2.28571	4.16619	1	3
[illegible]																

1.31	1.9	0.59	1.605	0.41719	3	4	1	3.5	0.70711	2	11	9
1.31	2.28	0.97	1.5525	0.68589	3	5	2	3.5	1.41421	2	9	7
1.31	2.54	1.23	1.925	0.86974	3	6	3	4.5	2.12132	2	11	9
1.31	1.78	0.47	1.545	0.33234	3	5	2	4	1.41421	2	13	11
1.31	1.54	0.23	1.425	0.16263	3	4	1	3.5	0.70711	2	4	2
1.31	2.54	1.23	1.6175	0.86974	3	6	3	3.75	2.12132	2	11	9
1.31	1.9	0.59	1.605	0.41719	3	4	1	3.5	0.70711	2	11	9
1.31	1.93	0.62	1.62	0.43841	3	5	2	4	1.41421	2	11	9
1.31	1.69	0.38	1.5	0.2687	3	5	2	4	1.41421	2	12	10
1.31	2.05	0.74	1.68	0.52326	3	5	2	4	1.41421	2	15	13
1.31	1.9	0.59	1.4575	0.41719	3	4	1	3.25	0.70711	2	11	9
1.31	3.44	2.13	2.375	1.50614	2	3	1	2.5	0.70711	2	16	14
1.31	2.2	0.89	1.755	0.62933	3	5	2	4	1.41421	2	10	8
1.31	2	0.69	1.4825	0.4879	3	6	3	3.75	2.12132	2	12	10
1.31	1.78	0.47	1.545	0.33234	3	5	2	4	1.41421	2	13	11
1.31	1.54	0.23	1.425	0.16263	3	4	1	3.5	0.70711	2	4	2
1.31	2.05	0.74	1.68	0.52326	3	5	2	4	1.41421	2	15	13
1.31	1.54	0.23	1.425	0.16263	3	4	1	3.5	0.70711	2	4	2
1.31	1.91	0.6	1.46	0.42426	3	4	1	3.25	0.70711	2	10	8
1.31	2.54	1.23	1.925	0.86974	3	6	3	4.5	2.12132	2	11	9
1.31	2.2	0.89	1.755	0.62933	3	5	2	4	1.41421	2	10	8
1.31	2.1	0.79	1.705	0.55861	3	5	2	4	1.41421	2	16	14
1.31	2	0.69	1.655	0.4879	3	6	3	4.5	2.12132	2	12	10
1.31	1.96	0.65	1.635	0.45962	3	5	2	4	1.41421	2	14	12
1.31	2.2	0.89	1.755	0.62933	3	5	2	4	1.41421	2	10	8
1.31	2.33	1.02	1.82	0.72125	3	6	3	4.5	2.12132	2	14	12
1.31	2.05	0.74	1.68	0.52326	3	5	2	4	1.41421	2	15	13
1.31	1.81	0.5	1.56	0.35355	3	4	1	3.5	0.70711	2	13	11
1.31	1.36	0.05	1.335	0.03536	3	4	1	3.5	0.70711	2	3	1
1.31	1.93	0.62	1.62	0.43841	3	5	2	4	1.41421	2	11	9
1.31	2.02	0.71	1.665	0.50205	3	6	3	4.5	2.12132	2	15	13
1.31	2.1	0.79	1.705	0.55861	3	5	2	4	1.41421	2	16	14
1.31	1.96	0.65	1.635	0.45962	3	5	2	4	1.41421	2	14	12
0.98	1.31	0.33	1.145	0.23335	2	3	1	2.5	0.70711	1	2	1
1.31	2	0.69	1.655	0.4879	3	6	3	4.5	2.12132	2	12	10
1.31	2.28	0.97	1.795	0.68589	3	5	2	4	1.41421	2	9	7
1.31	1.81	0.5	1.56	0.35355	3	4	1	3.5	0.70711	2	13	11
1.31	2	0.69	1.655	0.4879	3	6	3	4.5	2.12132	2	12	10
1.31	1.91	0.6	1.61	0.42426	3	4	1	3.5	0.70711	2	10	8
1.31	1.96	0.65	1.635	0.45962	3	5	2	4	1.41421	2	14	12

1.31	2.05	0.74	1.55667	0.52326	3	5	2	3.66667	1.41421	2	15	13	6.33333	9.19239	1	2	[illegible]
0.98	1.31	0.33	1.2	0.23335	2	3	1	2.66667	0.70711	1	2	1	1.66667	0.70711	1	1	[illegible]
1.31	1.36	0.05	1.31833	0.03536	3	4	1	3.16667	0.70711	2	3	1	2.16667	0.70711	1	3	[illegible]
1.31	1.54	0.23	1.38667	0.16263	3	4	1	3.33333	0.70711	2	4	2	2.66667	1.41421	1	3	[illegible]
1.31	2.33	1.02	1.65	0.72125	3	6	3	4	2.12132	2	14	12	6	8.48528	1	2	[illegible]
1.31	1.36	0.05	1.32667	0.03536	3	4	1	3.33333	0.70711	2	3	1	2.33333	0.70711	1	3	[illegible]
1.31	1.78	0.47	1.46667	0.33234	3	5	2	3.66667	1.41421	2	13	11	5.66667	7.77817	1	2	[illegible]
1.31	1.54	0.23	1.38667	0.16263	3	4	1	3.33333	0.70711	2	4	2	2.66667	1.41421	1	3	[illegible]
1.31	1.81	0.5	1.39333	0.35355	3	4	1	3.16667	0.70711	2	13	11	3.83333	7.77817	1	2	[illegible]
1.31	2.33	1.02	1.65	0.72125	3	6	3	4	2.12132	2	14	12	6	8.48528	1	2	[illegible]
1.31	1.65	0.34	1.42333	0.24042	3	4	1	3.33333	0.70711	2	12	10	5.33333	7.07107	1	3	[illegible]
1.31	1.78	0.47	1.46667	0.33234	3	5	2	3.66667	1.41421	2	13	11	5.66667	7.77817	1	2	[illegible]
1.31	1.54	0.23	1.425	0.16263	3	4	1	3.5	0.70711	2	4	2	3	1.41421	1	3	[illegible]
1.31	1.78	0.47	1.46667	0.33234	3	5	2	3.66667	1.41421	2	13	11	5.66667	7.77817	1	2	[illegible]
1.31	1.69	0.38	1.5	0.2687	3	5	2	4	1.41421	2	12	10	7	7.07107	1	3	[illegible]
1.31	1.54	0.23	1.38667	0.16263	3	4	1	3.33333	0.70711	2	4	2	2.66667	1.41421	1	3	[illegible]
[illegible]																	

1.31	1.54	0.23	1.38667	0.16263	3	4	1	3.33333	0.70711	2	4	2	2.66667	1.41421	1	3	2	1.67	1.41421	24.305	47.867	23.562	32.159	16.6608	1.4	1.5	0.1	1.47	0.07071	51	73	22	65.6667	15.5563	4.40E-08	4.00E-07	3.56E-07	1.63E-07	2.52E-07	4140	4602
1.31	1.65	0.34	1.48	0.24042	3	4	1	3.5	0.70711	2	12	10	7	7.07107	1	3	2	2	1.41421	24.305	65.409	41.104	44.857	29.0649	1.35	1.5	0.15	1.43	0.10607	73	76	3	74.5	2.12132	4.40E-08	6.00E-08	1.60E-08	5.20E-08	1.13E-08	3700	4602
0.98	1.96	0.98	1.36	0.52574	2	8	6	5	3	1	14	13	7	6.55744	1	3	2	2	1	6.941	144.242	137.301	89.9643	73.0248	1.45	1.85	0.4	1.58	0.23094	12	83	71	41.6667	36.9098	9.50E-08	6.43E-07	5.48E-07	2.84E-07	3.11E-07	2330	6000
0.98	2.01	1.03	1.56833	0.57272	2	8	6	5	2.86039	1	14	13	10.1667	5.97343	1	3	2	2.17	0.87905	6.941	151.964	145.023	88.1315	64.9357	1.25	1.85	0.6	1.48	0.34378	12	84	72	50	43.5681	9.50E-08	0.0005	0.0005	0.00025	0.00032	0	0
0.98	2.58	1.6	1.96667	0.9185	2	5	3	3	1.2792	1	16	15	11	9.04534	1	2	1	1.67	0.60302	6.941	127.6	120.659	39.6128	52.3473	1	1.45	0.45	1.22	0.27798	12	94	82	66.3333	49.1547	9.50E-08	1.00E+23	1.00E+23	5.00E+22	6.40E+22	0	0
0.98	3.44	2.46	2.44286	1.53189	2	9	7	3	3.24037	1	16	15	10.2857	8.96421	1	3	2	1.86	0.84515	6.941	238.029	231.088	45.1298	104.309	0.6	1.75	1.15	1.01	0.63387	12	101	89	67.5714	52.8103	0	0	0	0	0	317.5	6000
1.31	1.96	0.65	1.52667	0.45962	3	5	2	3.66667	1.41421	2	14	12	6	8.48528	1	2	1	1.33	0.70711	24.305	118.71	94.405	55.7733	66.7544	1.45	1.5	0.05	1.48	0.03536	73	83	10	76.3333	7.07107	4.40E-08	1.15E-07	7.10E-08	6.77E-08	5.02E-08	2500	4602
1.31	2.1	0.79	1.705	0.55861	3	5	2	4	1.41421	2	16	14	9	9.89949	1	2	1	1.5	0.70711	24.305	127.6	103.295	75.9525	73.0406	1.4	1.5	0.1	1.45	0.07071	73	92	19	82.5	13.435	4.40E-08	0.0001	1.00E-04	5.00E-05	7.07E-05	2610	4602
1.31	2.1	0.79	1.705	0.55861	3	5	2	4	1.41421	2	16	14	9	9.89949	1	2	1	1.5	0.70711	24.305	127.6	103.295	75.9525	73.0406	1.4	1.5	0.1	1.45	0.07071	73	92	19	82.5	13.435	4.40E-08	0.0001	1.00E-04	5.00E-05	7.07E-05	2610	4602
0.98	1.31	0.33	1.145	0.23335	2	3	1	2.5	0.70711	1	2	1	1.5	0.70711	1	1	0	1	0	6.941	24.305	17.364	15.623	12.2782	1.45	1.5	0.05	1.47	0.03536	12	73	61	42.5	43.1335	4.40E-08	9.50E-08	5.10E-08	6.95E-08	3.61E-08	4602	6000
0.98	2.28	1.3	1.82875	0.75848	1	6	5	1.9375	2.11809	1	10	9	2.125	3.94135	1	3	2	1.25	0.87586	1.00794	195.084	194.076	27.1215	84.1371	0.25	1.45	1.2	0.76	0.77052	12	103	91	70.1875	54.0202	0	0	0	0	0	1270	6000
1.31	2.28	0.97	1.795	0.68589	3	5	2	4	1.41421	2	9	7	5.5	4.94975	1	3	2	2	1.41421	24.305	102.906	78.6005	63.6052	55.5789	1.35	1.5	0.15	1.43	0.10607	65	73	8	69	5.65685	4.30E-08	4.40E-08	1.00E-09	4.35E-08	7.07E-10	4602	4700
1.31	2.05	0.74	1.68	0.52326	3	5	2	4	1.41421	2	15	13	8.5	9.19239	1	2	1	1.5	0.70711	24.305	121.76	97.455	73.0325	68.9111	1.45	1.5	0.05	1.47	0.03536	73	88	15	80.5	10.6066	4.40E-08	4.00E-07	3.56E-07	2.22E-07	2.52E-07	3420	4602
1.31	2.1	0.79	1.57333	0.55861	3	5	2	3.66667	1.41421	2	16	14	6.66667	9.89949	1	2	1	1.33	0.70711	24.305	127.6	103.295	58.7367	73.0406	1.4	1.5	0.1	1.47	0.07071	73	92	19	79.3333	13.435	4.40E-08	0.0001	1.00E-04	3.34E-05	7.07E-05	2610	4602
1.31	1.54	0.23	1.38667	0.16263	3	4	1	3.33333	0.70711	2	4	2	2.66667	1.41421	1	3	2	1.67	1.41421	24.305	47.867	23.562	32.159	16.6608	1.4	1.5	0.1	1.47	0.07071	51	73	22	65.6667	15.5563	4.40E-08	4.00E-07	3.56E-07	1.63E-07	2.52E-07	4140	4602
1.31	1.54	0.23	1.38667	0.16263	3	4	1	3.33333	0.70711	2	4	2	2.66667	1.41421	1	3	2	1.67	1.41421	24.305	47.867	23.562	32.159	16.6608	1.4	1.5	0.1	1.47	0.07071	51	73	22	65.6667	15.5563	4.40E-08	4.00E-07	3.56E-07	1.63E-07	2.52E-07	4140	4602
1.31	2.2	0.89	1.755	0.62933	3	5	2	4	1.41421	2	10	8	6	5.65685	1	3	2	2	1.41421	24.305	106.42	82.115	65.3625	58.0641	1.4	1.5	0.1	1.45	0.07071	69	73	4	71	2.82843	4.40E-08	1.08E-07	6.40E-08	7.60E-08	4.53E-08	3070	4602
1.31	2	0.69	1.655	0.4879	3	6	3	4.5	2.12132	2	12	10	7	7.07107	1	3	2	2	1.41421	24.305	200.59	176.285	112.447	124.652	1.5	1.5	0	1.5	0	73	74	1	73.5	0.70711	4.40E-08	9.60E-07	9.16E-07	5.02E-07	6.48E-07	1407	4602
1.2	1.31	0.11	1.255	0.07778	3	8	5	5.5	3.53553	2	9	7	5.5	4.94975	1	3	2	2	1.41421	24.305	151.964	127.659	88.1345	90.2685	1.5	1.85	0.35	1.68	0.24749	18	73	55	45.5	38.8909	4.40E-08	9.00E-07	8.56E-07	4.72E-07	6.05E-07	0	0
0.98	1.31	0.33	1.145	0.23335	2	3	1	2.5	0.70711	1	2	1	1.5	0.70711	1	1	0	1	0	6.941	24.305	17.364	15.623	12.2782	1.45	1.5	0.05	1.47	0.03536	12	73	61	42.5	43.1335	4.40E-08	9.50E-08	5.10E-08	6.95E-08	3.61E-08	4602	6000
1.31	1.81	0.5	1.56	0.35355	3	4	1	3.5	0.70711	2	13	11	7.5	7.77817	1	2	1	1.5	0.70711	24.305	69.723	45.418	47.014	32.1154	1.3	1.5	0.2	1.4	0.14142	73	81	8	77	5.65685	4.40E-08	1.40E-07	9.60E-08	9.20E-08	6.79E-08	2740	4602
1.31	2	0.69	1.655	0.4879	3	6	3	4.5	2.12132	2	12	10	7	7.07107	1	3	2	2	1.41421	24.305	200.59	176.285	112.447	124.652	1.5	1.5	0	1.5	0	73	74	1	73.5	0.70711	4.40E-08	9.60E-07	9.16E-07	5.02E-07	6.48E-07	1407	4602
1.31	1.93	0.62	1.51667	0.43841	3	5	2	3.66667	1.41421	2	11	9	5	6.36396	1	3	2	1.67	1.41421	24.305	107.868	83.5632	52.1594	59.0881	1.5	1.6	0.1	1.53	0.07071	71	73	2	72.3333	1.41421	1.63E-08	4.40E-08	2.77E-08	3.48E-08	1.96E-08	2600	4602
1.31	3.44	2.13	2.02	1.50614	2	3	1	2.66667	0.70711	2	16	14	6.66667	9.89949	1	2	1	1.33	0.70711	15.9994	24.305	8.3056	21.5365	5.87295	0.6	1.5	0.9	1.2	0.6364	73	101	28	82.3333	19.799	0	0	0	0	0	317.5	4602
1.31	1.54	0.23	1.425	0.16263	3	4	1	3.5	0.70711	2	4	2	3	1.41421	1	3	2	2	1.41421	24.305	47.867	23.562	36.086	16.6608	1.4	1.5	0.1	1.45	0.07071	51	73	22	62	15.5563	4.40E-08	4.00E-07	3.56E-07	2.22E-07	2.52E-07	4140	4602
1.31	3.44	2.13	2.375	1.50614	2	3	1	2.5	0.70711	2	16	14	9	9.89949	1	2	1	1.5	0.70711	15.9994	24.305	8.3056	20.1522	5.87295	0.6	1.5	0.9	1.05	0.6364	73	101	28	87	19.799	0	0	0	0	0	317.5	4602
1.31	2.05	0.74	1.55667	0.52326	3	5	2	3.66667	1.41421	2	15	13	6.33333	9.19239	1	2	1	1.33	0.70711	24.305	121.76	97.455	56.79	68.9111	1.45	1.5	0.05	1.48	0.03536	73	88	15	78	10.6066	4.40E-08	4.00E-07	3.56E-07	1.63E-07	2.52E-07	3420	4602
1.31	1.96	0.65	1.635	0.45962	3	5	2	4	1.41421	2	14	12	8	8.48528	1	2	1	1.5	0.70711	24.305	118.71	94.405	71.5075	66.7544	1.45	1.5	0.05	1.47	0.03536	73	83	10	78	7.07107	4.40E-08	1.15E-07	7.10E-08	7.95E-08	5.02E-08	2500	4602
1.31	2.02	0.71	1.54667	0.50205	3	6	3	4	2.12132	2	15	13	6.33333	9.19239	1	2	1	1.33	0.70711	24.305	208.98	184.675	85.8635	130.585	1.5	1.6	0.1	1.53	0.07071	73	87	14	77.6667	9.89949	4.40E-08	1.30E-06	1.26E-06	4.63E-07	8.88E-07	1790	4602
1.31	2.2	0.89	1.755	0.62933	3	5	2	4	1.41421	2	10	8	6	5.65685	1	3	2	2	1.41421	24.305	106.42	82.115	65.3625	58.0641	1.4	1.5	0.1	1.45	0.07071	69	73	4	71	2.82843	4.40E-08	1.08E-07	6.40E-08	7.60E-08	4.53E-08	3070	4602
1.31	1.81	0.5	1.56	0.35355	3	4	1	3.5	0.70711	2	13	11	7.5	7.77817	1	2	1	1.5	0.70711	24.305	69.723	45.418	47.014	32.1154	1.3	1.5	0.2	1.4	0.14142	73	81	8	77	5.65685	4.40E-08	1.40E-07	9.60E-08	9.20E-08	6.79E-08	2740	4602
1.31	1.93	0.62	1.62	0.43841	3	5	2	4	1.41421	2	11	9	6.5	6.36396	1	3	2	2	1.41421	24.305	107.868	83.5632	66.0866	59.0881	1.5	1.6	0.1	1.55	0.07071	71	73	2	72	1.41421	1.63E-08	4.40E-08	2.77E-08	3.01E-08	1.96E-08	2600	4602
1.31	2.28	0.97	1.795	0.68589	3	5	2	4	1.41421	2	9	7	5.5	4.94975	1	3	2	2	1.41421	24.305	102.906	78.6005	63.6052	55.5789	1.35	1.5	0.15	1.43	0.10607	65	73	8	69	5.65685	4.30E-08	4.40E-08	1.00E-09	4.35E-08	7.07E-10	4602	4700
1.31	2.54	1.23	1.925	0.86974	3	6	3	4.5	2.12132	2	11	9	6.5	6.36396	1	3	2	2	1.41421	24.305	196.967	172.662	110.636	122.09	1.35	1.5	0.15	1.43	0.10607	70	73	3	71.5	2.12132	2.20E-08	4.40E-08	2.20E-08	3.30E-08	1.56E-08	1740	4602
1.31	1.81	0.5	1.47667	0.35355	3	4	1	3.33333	0.70711	2	13	11	5.66667	7.77817	1	2	1	1.33	0.70711	24.305	69.723	45.418	39.4443	32.1154	1.3	1.5	0.2	1.43	0.14142	73	81	8	75.6667	5.65685	4.40E-08	1.40E-07	9.60E-08	7.60E-08	6.79E-08	2740	4602
1.31	1.81	0.5	1.56	0.35355	3	4	1	3.5	0.70711	2	13	11	7.5	7.77817	1	2	1	1.5	0.70711	24.305	69.723	45.418	47.014	32.1154	1.3	1.5	0.2	1.4	0.14142	73	81	8	77	5.65685	4.40E-08	1.40E-07	9.60E-08	9.20E-08	6.79E-08	2740	4602
0.98	3.44	2.46	2.29125	1.51302	2	4	2	2.25	0.8584	1	16	15	9	9.2224	1	3	2	1.75	0.8584	6.941	50.9415	44.0005	16.9703	17.5294	0.6	1.45	0.85	1.01	0.5368	12	101	89	61.75	53.6018	0	0	0	0	0	317.5	6000
0.98	3.44	2.46	2.275	1.52596	2	6	4	2.5	1.71679	1	16	15	9	9.2224	1	3	2	1.75	0.8584	6.941	180.948	174.007	33.2211	72.6658	0.6	1.45	0.85	1.03	0.55155	12	101	89	61.5	53.6696	0	0	0	0	0	317.5	6000
0.98	2.58	1.6	1.80556	0.98428	2	4	2	2.66667	0.86603	1	16	15	9.11111	9.45604	1	2	1	1.56	0.6455	6.941	72.64	65.699	25.4071	26.595	1	1.45	0.45	1.23	0.27576	12	94	82	56.4444	51.7848	9.50E-08	1.00E+23	1.00E+23	4.44E+22	6.45E+22	0	0
1.31	2.02	0.71	1.54667	0.50205	3	6	3	4	2.12132	2	15	13	6.33333	9.19239	1	2	1	1.33	0.70711	24.305	208.98	184.675	85.8635	130.585	1.5	1.6	0.1	1.53	0.07071	73	87	14	77.6667	9.89949	4.40E-08	1.30E-06	1.26E-06	4.63E-07	8.88E-07	1790	4602
1.31	2	0.69	1.54	0.4879	3	6	3	4	2.12132	2	12	10	5.33333	7.07107	1	3	2	1.67	1.41421	24.305	200.59	176.285	83.0667	124.652	1.5	1.5	0	1.5	0	73	74	1	73.3333	0.70711	4.40E-08	9.60E-07	9.16E-07	3.49E-07	6.48E-07	1407	4602
1.31	1.96	0.65	1.635	0.45962	3	5	2	4	1.41421	2	14	12	8	8.48528	1	2	1	1.5	0.70711	24.305	118.71	94.405	71.5075	66.7544	1.45	1.5	0.05	1.47	0.03536	73	83	10	78	7.07107	4.40E-08	1.15E-07	7.10E-08	7.95E-08	5.02E-08	2500	4602
1.31	3.44	2.13	2.02	1.50614	2	3	1	2.66667	0.70711	2	16	14	6.66667	9.89949	1	2	1	1.33	0.70711	15.9994	24.305	8.3056	21.5365	5.87295	0.6	1.5	0.9	1.2	0.6364	73	101	28	82.3333	19.799	0	0	0	0	0	317.5	4602

1.31	1.65	0.34	1.42333	0.24042	3	4	1	3.33333	0.70711	2	12	10	5.33333	7.07107	1	3	2	1.67	1.41421	24.305	65.409	41.104 [illegible]
1.31	2	0.69	1.54	0.4879	3	6	3	4	2.12132	2	12	10	5.33333	7.07107	1	3	2	1.67	1.41421	24.305	200.59	176.285 [illegible]
1.31	2.2	0.89	1.60667	0.62933	3	5	2	3.66667	1.41421	2	10	8	4.66667	5.65685	1	3	2	1.67	1.41421	24.305	106.42	82.115 [illegible]
1.31	1.9	0.59	1.40833	0.41719	3	4	1	3.16667	0.70711	2	11	9	3.5	6.36396	1	3	2	1.33	1.41421	24.305	63.546	39.241 [illegible]
1.31	2	0.69	1.54	0.4879	3	6	3	4	2.12132	2	12	10	5.33333	7.07107	1	3	2	1.67	1.41421	24.305	200.59	176.285 [illegible]
1.31	2.28	0.97	1.63333	0.68589	3	5	2	3.66667	1.41421	2	9	7	4.33333	4.94975	1	3	2	1.67	1.41421	24.305	102.906	78.6005 [illegible]
1.31	2.33	1.02	1.65	0.72125	3	6	3	4	2.12132	2	14	12	6	8.48528	1	2	1	1.33	0.70711	24.305	207.2	182.895 [illegible]
1.31	2.28	0.97	1.47167	0.68589	3	5	2	3.33333	1.41421	2	9	7	3.16667	4.94975	1	3	2	1.33	1.41421	24.305	102.906	78.6005 [illegible]
1.31	1.96	0.65	1.635	0.45962	3	5	2	4	1.41421	2	14	12	8	8.48528	1	2	1	1.5	0.70711	24.305	118.71	94.405 [illegible]
1.31	1.91	0.6	1.46	0.42426	3	4	1	3.25	0.70711	2	10	8	4	5.65685	1	3	2	1.5	1.41421	24.305	58.6934	34.3884 [illegible]
0.98	3.44	2.46	2.06051	1.52507	2	4	2	2.20253	0.79092	1	16	15	8.29114	9.50354	1	2	1	1.51	0.65537	6.941	69.723	62.782 [illegible]
1.31	1.81	0.5	1.47667	0.35355	3	4	1	3.33333	0.70711	2	13	11	5.66667	7.77817	1	2	1	1.33	0.70711	24.305	69.723	45.418 [illegible]
0.98	3.44	2.46	2.06051	1.52507	2	4	2	2.20253	0.79092	1	16	15	8.29114	9.50354	1	2	1	1.51	0.65537	6.941	69.723	62.782 [illegible]
1.31	2.2	0.89	1.755	0.62933	3	5	2	4	1.41421	2	10	8	6	5.65685	1	3	2	2	1.41421	24.305	106.42	82.115 [illegible]
1.31	1.9	0.59	1.605	0.41719	3	4	1	3.5	0.70711	2	11	9	6.5	6.36396	1	3	2	2	1.41421	24.305	63.546	39.241 [illegible]
1.31	1.9	0.59	1.605	0.41719	3	4	1	3.5	0.70711	2	11	9	6.5	6.36396	1	3	2	2	1.41421	24.305	63.546	39.241 [illegible]

[illegible]

1.31	1.91	0.6	1.61	0.42426	3	4	1	3.5	0.70711	2	10	8	6	5.65685	1	3	[illegible]
1.31	1.81	0.5	1.56	0.35355	3	4	1	3.5	0.70711	2	13	11	7.5	7.77817	1	2	[illegible]
1	2.01	1.01	1.67333	0.71418	4	4	0	4	0	2	14	12	10	8.48528	1	2	[illegible]
1.31	2.02	0.71	1.665	0.50205	3	6	3	4.5	2.12132	2	15	13	8.5	9.19239	1	2	[illegible]
1.31	3.44	2.13	2.375	1.50614	2	3	1	2.5	0.70711	2	16	14	9	9.89949	1	2	[illegible]
0.98	2.2	1.22	2.03231	0.41575	1	2	1	1.53846	0.66144	1	13	12	6.53846	7.93725	1	2	[illegible]
0.98	3.44	2.46	2.144	1.56007	2	8	6	2.8	2.62342	1	16	15	9.33333	8.99346	1	3	[illegible]
0.98	3.04	2.06	1.7675	1.30069	2	3	1	2.08333	0.37553	1	15	14	6.83333	9.3781	1	2	[illegible]
1.13	1.31	0.18	1.22	0.12728	3	8	5	5.5	3.53553	2	5	3	3.5	2.12132	1	3	[illegible]
1	2.02	1.02	1.628	0.47232	4	6	2	4.8	1.22474	2	15	13	9.2	6.34429	1	3	[illegible]
0.98	3.44	2.46	2.275	1.52596	2	6	4	2.5	1.71679	1	16	15	9	9.2224	1	3	[illegible]
1.31	1.91	0.6	1.51	0.42426	3	4	1	3.33333	0.70711	2	10	8	4.66667	5.65685	1	3	[illegible]
1.31	1.91	0.6	1.61	0.42426	3	4	1	3.5	0.70711	2	10	8	6	5.65685	1	3	[illegible]
1.31	3.44	2.13	2.375	1.50614	2	3	1	2.5	0.70711	2	16	14	9	9.89949	1	2	[illegible]
0.89	3.04	2.15	1.45	1.1621	2	6	4	4	2.52982	1	15	14	5	7.3212	1	2	[illegible]
0.98	3.44	2.46	2.77333	1.38522	2	6	4	2.66667	2.10819	1	16	15	12	8.3666	1	3	[illegible]
[illegible]																	

1.22	2.2	0.98	1.81	0.51971	4	8	4	5.66667	2.08167	8	14	6	11.3333	3.05505	2	3	[illegible]
1.22	2.28	1.06	1.83667	0.55085	4	8	4	5.66667	2.08167	9	14	5	11.6667	2.51661	2	3	[illegible]
1.24	2.28	1.04	1.84333	0.53966	4	8	4	5.66667	2.08167	9	14	5	12.3333	2.88675	2	3	[illegible]
1.5	2.19	0.69	1.67	0.37041	3	6	3	5.16667	1.71594	5	15	10	7.16667	5.1908	2	3	[illegible]
0.79	3.44	2.65	2.26333	1.46845	2	6	4	3.16667	1.79118	1	16	15	9	8.66025	1	3	[illegible]
0.82	3.44	2.62	2.26833	1.46097	2	5	3	3	1.41421	1	16	15	9	8.66025	1	3	[illegible]
1.38	2.58	1.2	2.17	0.68432	3	9	6	5.33333	3.21455	6	16	10	12.6667	5.7735	2	3	[illegible]
0.95	3.98	3.03	3.03	1.90568	2	5	3	2.66667	1.56347	2	17	15	12	10	1	2	[illegible]
1.54	1.9	0.36	1.78	0.20785	3	4	1	3.66667	0.57735	4	14	10	9.66667	5.1316	2	3	[illegible]
1.3	1.9	0.6	1.7	0.34641	3	6	3	4.33333	1.52753	4	14	10	9.66667	5.1316	2	3	[illegible]
2.2	3.44	1.24	3.09	0.71698	2	5	3	2.66667	1.56347	10	16	6	15	3.16228	2	3	[illegible]
1.36	2.28	0.92	1.84667	0.46231	3	5	2	4	1	3	14	11	8.66667	5.50757	2	3	[illegible]
0.82	2.58	1.76	2.18917	0.84714	3	9	6	4.33333	3.0844	1	16	15	13	7.18106	1	3	[illegible]
1.1	1.9	0.8	1.51667	0.40104	3	8	5	5	2.64575	7	14	7	10.6667	3.51188	2	3	[illegible]
1.22	1.9	0.68	1.55667	0.34005	3	8	5	5	2.64575	7	14	7	11	3.60555	2	3	[illegible]
1.24	1.9	0.66	1.56333	0.3302	3	8	5	5	2.64575	7	14	7	11.6667	4.04145	2	3	[illegible]
1.22	2.2	0.98	1.77333	0.50213	3	5	2	4.33333	1.1547	3	14	11	8.33333	5.50757	2	3	[illegible]
1.1	2.2	1.1	1.73333	0.56862	3	8	5	5.33333	2.51661	8	14	6	11	3	2	3	[illegible]
2.02	3.44	1.42	2.96667	1.00409	2	6	4	3.33333	2.82843	15	16	1	15.6667	0.70711	2	2	[illegible]
0.89	3.44	2.55	2.9125	1.13377	2	6	4	2.75	1.81212	2	16	14	13.8333	6.26013	1	3	[illegible]
1.36	2.28	0.92	1.84667	0.46231	3	6	3	4.33333	1.52753	3	14	11	9	5.56776	2	3	[illegible]
1.22	1.91	0.69	1.67667	0.39552	3	5	2	4	1	3	14	11	9	5.56776	2	3	[illegible]
1.27	3.44	2.17	2.87	1.20028	2	8	6	3.16667	3.10018	15	17	2	16	0.8165	2	3	[illegible]
0.89	3.44	2.55	2.36333	1.39708	2	6	4	2.83333	1.79118	1	16	15	10.6667	8.05191	1	2	[illegible]
1.33	1.9	0.57	1.71	0.32909	3	5	2	4	1	4	14	10	9.66667	5.1316	2	3	[illegible]
1.24	2.2	0.96	1.78	0.49112	3	8	5	5.33333	2.51661	8	14	6	12	3.4641	2	3	[illegible]
1.9	2.55	0.65	2.225	0.41574	3	4	1	3.83333	0.47673	11	16	5	14	2.86039	2	3	[illegible]
[illegible]																	

1.69	2.96	1.27	2.15167	0.54375	3	5	2	3.83333	1.14812	12	17	5	14.3333	2.29624	2	3
1.69	3.16	1.47	2.185	0.62684	3	5	2	3.66667	1.20605	12	17	5	14.3333	2.29624	2	3
0.82	2.1	1.28	1.78667	0.56472	4	5	1	4.33333	0.60302	1	16	15	11	6.39602	1	3
1.69	3.16	1.47	2.185	0.62684	3	5	2	3.66667	1.20605	12	17	5	14.3333	2.29624	2	3
1.69	2.96	1.27	2.15167	0.54375	3	5	2	3.83333	1.14812	12	17	5	14.3333	2.29624	2	3
1.69	2.66	0.97	2.10167	0.42877	3	5	2	4	1.2792	12	17	5	14.3333	2.29624	2	3
1.23	1.91	0.68	1.65	0.36715	4	8	4	5.33333	2.3094	10	13	3	12	1.73205	2	3
1.5	2.2	0.7	1.85	0.49497	6	6	0	6	0	5	9	4	7	2.82843	3	3
1.12	2.28	1.16	1.67	0.58232	3	8	5	5.66667	2.51661	4	13	9	9	4.58258	2	3
0.95	1.96	1.01	1.40667	0.51189	3	5	2	4.33333	1.1547	2	14	12	6	6.9282	1	2
1.6	1.96	0.36	1.84	0.25456	5	5	0	5	0	5	14	9	11	6.36396	2	3
2.18	2.33	0.15	2.24	0.08191	4	6	2	5.16667	0.87905	10	15	5	12.1667	2.80422	2	3
1	2.54	1.54	1.78333	0.77035	4	6	2	4.66667	1.1547	2	13	11	8.66667	5.85947	1	3
0.89	2.28	1.39	1.72667	0.73704	4	6	2	5.33333	1.1547	2	14	12	8.66667	6.1101	1	3
1.57	1.83	0.26	1.65667	0.18385	2	4	2	2.66667	1.41421	2	8	6	4	4.24264	1	3
1.12	1.96	0.84	1.66333	0.4712	4	8	4	5.66667	2.08167	4	14	10	9.33333	5.03322	2	3

0.95	1.96	1.01	1.30333	0.56924	4	5	1	4.66667	0.57735	2	14	12	6	6.9282	1	2
0.95	2.33	1.38	1.42667	0.78271	4	6	2	5	1	2	14	12	6	6.9282	1	2
1.23	3.44	2.21	2.775	1.34154	2	8	6	3.33333	3.12694	6	16	10	13.8333	5.1908	2	3
0.79	2.1	1.31	1.66667	0.61886	5	8	3	5.66667	1.35401	1	16	15	12.1667	6.49679	1	3
0.79	2.1	1.31	1.64667	0.64248	5	8	3	5.66667	1.35401	1	16	15	10.6667	7.7352	1	3
0.79	2.1	1.31	1.675	0.61019	5	8	3	5.66667	1.35401	1	17	16	13	6.85565	1	3
0.79	2.1	1.31	1.65167	0.63622	5	8	3	5.66667	1.35401	1	16	15	11	7.28011	1	3
0.79	2.1	1.31	1.65833	0.62823	5	8	3	5.66667	1.35401	1	16	15	11.5	6.77311	1	3
0.89	2.2	1.31	1.70167	0.55728	5	6	1	5.33333	0.66667	2	13	11	10.5	5.75905	1	3
0.89	3.44	2.55	2.84333	1.26111	2	6	4	2.75	1.81212	2	16	14	13.6667	6.69812	1	3
1.66	3.44	1.78	2.88667	1.111	2	6	4	3	2.16025	6	16	10	12.8333	6.34648	2	3
1.9	2.28	0.38	2.07	0.2234	3	6	3	4.33333	1.7581	6	14	8	11.3333	3.81385	2	3
0.82	3.44	2.62	1.575	1.61385	2	6	4	3.66667	1.58565	1	16	15	5.91667	9.97926	1	2
0.95	3.44	2.49	2.55417	1.37334	2	8	6	3.41667	2.86722	2	16	14	13.1667	5.48571	1	3
0.89	3.98	3.09	3.02	1.92764	2	6	4	2.83333	2.06828	2	17	15	12	10	1	2
1.12	1.96	0.84	1.46333	0.44049	3	8	5	5.33333	2.51661	2	14	12	6.66667	6.4291	1	3

[illegible]

1.5	2.2	0.7	1.86667	0.35119	3	6	3	5	1.73205	5	14	9	9.33333	4.50925	2	3	1	2.67	0.57735	28.0855	192.217	164.132	133.75
1.31	2.01	0.7	1.66	0.49497	3	4	1	3.5	0.70711	2	14	12	8	8.48528	1	2	1	1.5	0.70711	24.305	72.64	48.335	48.4725
0.82	2.1	1.28	1.72667	0.75801	5	8	3	5.5	1.58114	1	16	15	11.8333	8.57969	1	3	2	2	0.8165	85.4678	144.242	58.7742	123.352
0.82	2.1	1.28	1.725	0.7606	5	8	3	5.5	1.58114	1	16	15	11.6667	8.81917	1	3	2	2	0.8165	85.4678	140.908	55.4398	122.796
1.31	1.93	0.62	1.62	0.43841	3	5	2	4	1.41421	2	11	9	6.5	6.36396	1	3	2	2	1.41421	24.305	107.868	83.5632	66.0866
1.31	2.05	0.74	1.68	0.52326	3	5	2	4	1.41421	2	15	13	8.5	9.19239	1	2	1	1.5	0.70711	24.305	121.76	97.455	73.0325
0.82	2.1	1.28	1.72667	0.75801	4	8	4	5.33333	1.76383	1	16	15	11.8333	8.57969	1	3	2	2	0.8165	39.0983	144.242	105.144	115.623
1.13	2.28	1.15	1.57333	0.61857	3	8	5	5.33333	2.51661	2	9	7	5.33333	3.51188	1	3	2	2.33	1.1547	24.305	140.908	116.603	89.3727
0.95	2.28	1.33	1.67	0.67179	5	6	1	5.33333	0.57735	2	13	11	8.33333	5.68624	1	3	2	2	1	87.62	195.084	107.464	132.507
1.6	2.2	0.6	1.9	0.3	3	5	2	4.33333	1.1547	5	14	9	9.66667	4.50925	2	3	1	2.67	0.57735	28.0855	106.42	78.3345	75.804
1.96	2.96	1	2.62667	0.70711	4	5	1	4.33333	0.70711	14	17	3	16	2.12132	2	2	0	2	0	79.904	118.71	38.806	92.8393
1.17	2.28	1.11	1.82	0.57888	4	8	4	6	2	8	14	6	10.6667	3.05505	2	3	1	2.67	0.57735	72.64	195.084	122.444	139.361
1.6	2.2	0.6	1.9	0.3	3	6	3	4.66667	1.52753	5	14	9	9.33333	4.50925	2	3	1	2.67	0.57735	28.0855	192.217	164.132	104.403
1.1	2.54	1.44	1.77667	0.7239	5	8	3	6.33333	1.52753	11	16	5	13	2.64575	3	3	0	3	0	112.411	196.967	84.5556	160.806
1.31	2.2	0.89	1.89167	0.38819	3	5	2	4.66667	1.05409	2	14	12	11	6.48074	1	3	2	2	0.8165	24.305	118.71	94.405	100.036
0.95	2.58	1.63	1.97667	0.81415	3	8	5	4.33333	2.19848	2	17	15	13	6.48074	1	3	2	2.17	0.84163	32.065	174.967	142.902	70.388
0.82	2.1	1.28	1.725	0.60489	5	6	1	5.16667	0.45644	1	16	15	10.6667	7.47217	1	3	2	2.17	0.84163	85.4678	178.49	93.0222	125.771
0.95	2.28	1.33	1.73	0.69419	5	6	1	5.33333	0.57735	2	14	12	8.66667	6.1101	1	3	2	2	1	87.62	195.084	107.464	133.805
1.27	2.2	0.93	1.82667	0.49136	4	8	4	6	2	9	17	8	13.3333	4.04145	2	3	1	2.67	0.57735	72.64	192.217	119.577	146.608
1.13	2.55	1.42	2.07667	1.00409	4	8	4	5.33333	2.82843	5	16	11	12.3333	7.77817	2	3	1	2.33	0.70711	78.96	140.908	61.9476	99.6092
1.24	2.2	0.96	1.78	0.49112	3	8	5	5.66667	2.51661	9	14	5	12.3333	2.88675	2	3	1	2.67	0.57735	28.0855	192.217	164.132	129.187
1.93	3.16	1.23	2.37333	0.54491	3	5	2	3.83333	1.19523	11	17	6	14	3.12984	2	3	1	2.42	0.59761	30.9738	107.868	76.8944	64.1234
1.23	2.2	0.97	1.77667	0.49662	3	8	5	5.66667	2.51661	9	14	5	12	2.64575	2	3	1	2.67	0.57735	28.0855	192.217	164.132	128.411

1.1	1.91	0.81	1.7075	0.57276	4	8	4	5	2.82843	10	16	6	11.5	4.24264	3	3	[illegible]
1.22	2.58	1.36	2.01333	0.96167	3	8	5	5.08333	3.53553	12	16	4	14.3333	2.82843	2	3	[illegible]
1.22	1.91	0.69	1.7375	0.4879	4	8	4	5	2.82843	10	12	2	10.5	1.41421	3	3	[illegible]
1.36	3.44	2.08	2.90167	1.09825	2	6	4	3	2.05334	3	16	13	13	6.61693	2	3	[illegible]
2.02	2.2	0.18	2.08	0.12728	6	6	0	6	0	9	15	6	13	4.24264	2	3	[illegible]
1.2	2.2	1	1.95	0.70711	6	8	2	6.5	1.41421	9	10	1	9.25	0.70711	3	3	[illegible]
1.2	1.91	0.71	1.7325	0.50205	4	8	4	5	2.82843	10	10	0	10	0	3	3	[illegible]
1.25	1.83	0.58	1.685	0.41012	4	8	4	5	2.82843	8	15	7	9.75	4.94975	3	3	[illegible]
1.1	1.96	0.86	1.65667	0.48274	4	8	4	5.66667	2.08167	3	14	11	9	5.56776	2	3	[illegible]
1.22	1.83	0.61	1.6775	0.43134	4	5	1	4.25	0.70711	3	8	5	6.75	3.53553	3	3	[illegible]
0.82	3.44	2.62	2.40667	1.36665	2	8	6	3.83333	2.68483	1	16	15	10.3333	7.70281	1	3	[illegible]
1.13	1.91	0.78	1.715	0.55154	4	8	4	5	2.82843	5	10	5	8.75	3.53553	3	3	[illegible]
1.54	1.81	0.27	1.72	0.19092	4	4	0	4	0	4	13	9	10	6.36396	2	3	[illegible]
1.27	1.27	0	1.27	0	8	8	0	8	0	17	17	0	17	0	3	3	[illegible]
0	0	0	0	0	2	2	0	2	0	18	18	0	18	0	2	2	[illegible]
1.13	1.13	0	1.13	0	8	8	0	8	0	5	5	0	5	0	3	3	[illegible]
1.63	1.63	0	1.63	0	4	4	0	4	0	5	5	0	5	0	3	3	[illegible]
0	0	0	0	0	2	2	0	2	0	18	18	0	18	0	2	2	[illegible]
2.28	2.28	0	2.28	0	5	5	0	5	0	9	9	0	9	0	3	3	[illegible]

[illegible]

1.1	1.96	0.86	1.53	0.60811	5	8	3	6.5	2.12132	14	16	2	15	1.41421	2	3	1	2.5	0.70711	118.71	173.04	54.33	145.875	38.4171	1.45	1.75	0.3	1.6	0.21213	17	83	66	50	46.669	1.15E-07	2.50E-07	1.35E-07	1.83E-07	9.55E-08	1590	2500	910	2045	643.467	39	67	28	53	19.799	505.08	1097	591.92	801.04	418.551	31	58	27	44.5	19.0919	2.20E-05	2.63E-05	4.30E-06	2.41E-05	3.04E-06	53.926	123	3.45319	3.45319	4.52227	1.5708	1.5708	1.5708	-0.7204
1.14	1.61	0.47	1.375	0.33234	3	8	5	5.5	3.53553	6	13	7	9.5	4.94975	2	3	1	2.5	0.70711	26.9815	144.242	117.26	85.6118	82.9157	1.25	1.85	0.6	1.55	0.42426	30	80	50	55	35.3553	2.70E-08	6.43E-07	6.16E-07	3.35E-07	4.36E-07	2330	5100	2770	3715	1958.69	17	235	218	126	154.149	933.47	1297	363.53	1115.24	257.055	32	76	44	54	31.1127	9.60E-06	2.31E-05	1.35E-05	1.63E-05	9.55E-06	51.7355	221	3.72617	3.72617	3.72617	1.5708	1.5708	1.5708	-0.3524
1.55	2.28	0.73	1.915	0.51619	4	5	1	4.5	0.70711	7	9	2	8	1.41421	3	3	0	3	0	54.938	102.906	47.9675	78.9218	33.9181	1.35	1.4	0.05	1.38	0.03536	60	65	5	62.5	3.53553	4.30E-08	1.44E-06	1.40E-06	7.42E-07	9.88E-07	4700	5150	450	4925	318.198	7.8	150	142.2	78.9	100.551	1519	2237	718	1878	507.703	120	380	260	250	183.848	8.20E-06	2.17E-05	1.35E-05	1.50E-05	9.55E-06	27.8137	221	3.02984	3.02984	3.02984	1.5708	1.5708	1.5708	-0.1849
1.83	3.44	1.61	2.635	1.13844	2	4	2	3	1.41421	8	16	8	12	5.65685	2	3	1	2.5	0.70711	15.9994	55.845	39.8456	35.9222	28.1751	0.6	1.4	0.8	1	0.56569	61	101	40	81	28.2843	0	0	0	0	0	317.5	4910	4592.5	2613.75	3247.39	0.02658	80	79.9734	40.0133	56.5497	54.8	1811	1756.2	932.9	1241.82	0	0	0	0	0	0	0	0	0	0	19.605	225	3.02664	3.02664	3.02664	1.5708	2.0944	2.0944	-1.2172
1.23	2.58	1.35	1.905	0.95459	3	8	5	5.5	3.53553	13	16	3	14.5	2.12132	2	3	1	2.5	0.70711	32.065	164.93	132.865	98.4977	93.95	1	1.75	0.75	1.38	0.53033	23	94	71	58.5	50.2046	8.14E-07	1.00E+23	1.00E+23	5.00E+22	7.07E+22	0	0	0	0	0	0.205	16	15.795	8.1025	11.1688	388.36	1734	1345.64	1061.18	951.511	7.7	40	32.3	23.85	22.8395	0	0	0	0	0	41.2746	225	3.87912	3.87912	3.87912	1.5708	2.0944	2.0944	-2.1187
2	2.1	0.1	2.05	0.07071	5	6	1	5.5	0.70711	12	16	4	14	2.82843	2	3	1	2.5	0.70711	127.6	200.59	72.99	164.095	51.6117	1.4	1.5	0.1	1.45	0.07071	74	92	18	83	12.7279	9.60E-07	0.0001	9.90E-05	5.05E-05	7.00E-05	1407	2610	1203	2008.5	850.649	3	8.3	5.3	5.65	3.74767	234.32	722.66	488.34	478.49	345.309	25	65	40	45	28.2843	0	0	0	0	0	71.763	216	4.6645	4.6645	4.6645	1.5708	2.0944	2.0944	-0.1373
1.17	1.62	0.45	1.395	0.3182	6	8	2	7	1.41421	8	13	5	10.5	3.53553	2	3	1	2.5	0.70711	150.36	204.383	54.0233	177.372	38.2002	1.85	1.9	0.05	1.88	0.03536	28	78	50	53	35.3553	1.50E-07	9.40E-07	7.90E-07	5.45E-07	5.59E-07	818	2130	1312	1474	927.724	13	46	33	29.5	23.3345	577	1345	768	961	543.058	38	43	5	40.5	3.53553	1.27E-05	2.99E-05	1.72E-05	2.13E-05	1.22E-05	56.2719	123	3.59792	3.59792	4.347	1.5708	1.5708	1.5708	-0.3803
1.61	2.02	0.41	1.815	0.28991	3	6	3	4.5	2.12132	13	15	2	14	1.41421	2	2	0	2	0	26.9815	208.98	181.999	117.981	128.693	1.25	1.6	0.35	1.43	0.24749	80	87	7	83.5	4.94975	2.70E-08	1.30E-06	1.27E-06	6.64E-07	9.00E-07	1790	5100	3310	3445	2340.52	8	235	227	121.5	160.513	544.4	933.47	389.07	738.935	275.114	31	76	45	53.5	31.8198	1.34E-05	2.31E-05	9.70E-06	1.83E-05	6.86E-06	65.9149	216	4.53419	4.53419	4.53419	1.5708	2.0944	2.0944	0.05924
1.1	1.9	0.8	1.5	0.56569	4	8	4	6	2.82843	11	11	0	11	0	3	3	0	3	0	63.546	158.925	95.3794	111.236	67.4434	1.35	1.75	0.4	1.55	0.28284	26	72	46	49	32.5269	1.72E-08	1.15E-06	1.13E-06	5.84E-07	8.01E-07	2620	3570	950	3095	671.751	11	400	389	205.5	275.065	1357.77	1629	271.23	1493.39	191.789	38.7	140	101.3	89.35	71.6299	1.03E-05	1.65E-05	6.20E-06	1.34E-05	4.38E-06	41.7658	221	3.46955	3.46955	3.46955	1.5708	1.5708	1.5708	0.09088
1.36	2.2	0.84	1.78	0.59397	4	5	1	4.5	0.70711	3	10	7	6.5	4.94975	3	3	0	3	0	44.9559	106.42	61.4641	75.688	43.4617	1.4	1.6	0.2	1.5	0.14142	19	69	50	44	35.3553	1.08E-07	5.50E-07	4.42E-07	3.29E-07	3.13E-07	0	0	0	0	0	16	72	56	44	39.598	1814	1828.05	14.05	1821.03	9.93485	57	180	123	118.5	86.9741	1.02E-05	1.18E-05	1.60E-06	1.10E-05	1.13E-06	35.9492	221	3.30037	3.30037	3.30037	1.5708	1.5708	1.5708	-0.9074
2.04	2.19	0.15	2.115	0.10607	2	3	1	2.5	0.70711	13	15	2	14	1.41421	2	2	0	2	0	10.811	30.9738	20.1628	20.8924	14.2572	0.85	1	0.15	0.93	0.10607	86	90	4	88	2.82843	1.00E-07	1E+12	1E+12	5E+11	7.1E+11	0	0	0	0	0	0.236	27	26.764	13.618	18.925	317.3	2349	2031.7	1333.15	1436.63	11	320	309	165.5	218.496	0	0	0	0	0	23.4126	216	3.21111	3.21111	3.21111	1.5708	2.0944	2.0944	-0.5351
0	0	0	0	0	2	2	0	2	0	18	18	0	18	0	2	2	0	2	0	20.1797	20.1797	0	20.1797	0	0	0	0	0	0	2	2	0	2	0	0	0	0	0	0	936	936	0	936	0	0.0491	0.0491	0	0.0491	0	24.56	24.56	0	24.56	0	0	0	0	0	0	0	0	0	0	0	10.3103	225	2.44303	2.44303	2.44303	1.5708	2.0944	2.0944	0.29902
2.2	3.04	0.84	2.62	0.59397	2	5	3	3.5	2.12132	10	15	5	12.5	3.53553	2	3	1	2.5	0.70711	14.0067	106.42	92.4133	60.2134	65.3461	0.65	1.4	0.75	1.03	0.53033	69	100	31	84.5	21.9203	0	0	0	0	0	333.6	3070	2736.4	1701.8	1934.93	0.02583	72	71.9742	36.0129	50.8934	63.05	1828.05	1765	945.55	1248.04	0	0	0	0	0	0	0	0	0	0	21.1154	221	2.76397	2.76397	2.76397	1.5708	1.5708	1.5708	1.4115
1.24	1.62	0.38	1.43	0.2687	6	8	2	7	1.41421	13	14	1	13.5	0.70711	2	3	1	2.5	0.70711	167.259	204.383	37.1243	185.821	26.2508	1.75	1.9	0.15	1.83	0.10607	22	78	56	50	39.598	1.50E-07	8.60E-07	7.10E-07	5.05E-07	5.02E-07	818	2830	2012	1824	1422.7	15	46	31	30.5	21.9203	577	1802	1225	1189.5	866.206	43	44	1	43.5	0.70711	1.22E-05	2.99E-05	1.77E-05	2.11E-05	1.25E-05	52.0381	123	3.51624	3.51624	4.20887	1.5708	1.5708	1.5708	-0.3062
1.91	2.28	0.37	2.095	0.26163	4	6	2	5	1.41421	10	10	0	10	0	3	3	0	3	0	58.6934	195.084	136.391	126.889	96.4427	1.35	1.35	0	1.35	0	67	68	1	67.5	0.70711	7.20E-08	1.06E-07	3.40E-08	8.90E-08	2.40E-08	2680	4970	2290	3825	1619.27	72	91	19	81.5	13.435	1728	2041.4	313.4	1884.7	221.607	180	230	50	205	35.3553	8.80E-06	1.34E-05	4.60E-06	1.11E-05	3.25E-06	26.7066	123	2.71924	2.71924	3.61181	1.5708	1.5708	1.5708	-0.1011
1.54	2	0.46	1.77	0.32527	4	6	2	5	1.41421	4	12	8	8	5.65685	3	3	0	3	0	47.867	200.59	152.723	124.229	107.991	1.4	1.5	0.1	1.45	0.07071	51	74	23	62.5	16.2635	4.00E-07	9.60E-07	5.60E-07	6.80E-07	3.96E-07	1407	4140	2733	2773.5	1932.52	8.3	22	13.7	15.15	9.68736	234.32	1941	1706.68	1087.66	1206.81	25	110	85	67.5	60.1041	0	0	0	0	0	37.1921	123	3.02502	3.02502	4.06439	1.5708	1.5708	1.5708	-0.0071
1.24	2.1	0.86	1.67	0.60811	5	8	3	6.5	2.12132	14	16	2	15	1.41421	2	3	1	2.5	0.70711	127.6	167.259	39.659	147.43	28.0431	1.4	1.75	0.35	1.58	0.24749	22	92	70	57	49.4975	8.60E-07	0.0001	9.91E-05	5.04E-05	7.01E-05	2610	2830	220	2720	155.563	3	15	12	9	8.48528	722.66	1802	1079.34	1262.33	763.209	44	65	21	54.5	14.8492	0	0	0	0	0	56.6847	225	4.31182	4.31182	4.31182	1.5708	2.0944	2.0944	-1.2082
1.54	1.61	0.07	1.575	0.0495	3	4	1	3.5	0.70711	4	13	9	8.5	6.36396	2	3	1	2.5	0.70711	26.9815	47.867	20.8855	37.4243	14.7683	1.25	1.4	0.15	1.33	0.10607	51	80	29	65.5	20.5061	2.70E-08	4.00E-07	3.73E-07	2.14E-07	2.64E-07	4140	5100	960	4620	678.823	22	235	213	128.5	150.614	933.47	1941	1007.53	1437.24	712.431	76	110	34	93	24.0416	8.60E-06	2.31E-05	1.45E-05	1.59E-05	1.03E-05	32.2486	123	2.81776	2.81776	4.06167	1.5708	1.5708	1.5708	-0.436
1.62	3.16	1.54	2.39	1.08894	3	6	3	4.5	2.12132	13	17	4	15	2.82843	2	2	0	2	0	35.453	204.383	168.93	119.918	119.452	1	1.9	0.9	1.45	0.6364	78	99	21	88.5	14.8492	1.50E-07	1E+10	1E+10	5E+09	7.1E+09	206	818	612	512	432.749	0.0089	46	45.9911	23.0045	32.5206	171.6	577	405.4	374.3	286.661	1.1	43	41.9	22.05	29.6278	0	0	0	0	0	57.9995	221	3.87087	3.87087	3.87087	1.5708	1.5708	1.5708	-1.1567
0.98	3.16	2.18	2.07	1.54149	2	3	1	2.5	0.70711	1	17	16	9	11.3137	1	2	1	1.5	0.70711	6.941	35.453	28.512	21.197	20.161	1	1.45	0.45	1.23	0.3182	12	99	87	55.5	61.5183	9.50E-08	1E+10	1E+10	5E+09	7.1E+09	206	6000	5794	3103	4096.98	0.0089	85	84.9911	42.5045	60.0978	171.6	453.69	282.09	312.645	199.468	1.1	11	9.9	6.05	7.00036	0	0	0	0	0	32.817	225	3.59367	3.59367	3.59367	1.5708	2.0944	2.0944	-1.9998
0.98	2.96	1.98	1.97	1.40007	2	4	2	3	1.41421	1	17	16	9	11.3137	1	2	1	1.5	0.70711	6.941	79.904	72.963	43.4225	51.5926	1.15	1.45	0.3	1.3	0.21213	12	98	86	55	60.8112	9.50E-08	1.00E+18	1.00E+18	5.00E+17	7.07E+17	0	0	0	0	0	0.12	85	84.88	42.56	60.0192	265.8	453.69	187.89	359.745	132.858	1.9	11	9.1	6.45	6.43467	0	0	0	0	0	40.4546	225	3.85325	3.85325	3.85325	1.5708	2.0944	2.0944	-1.7073
1.1	2.02	0.92	1.56	0.65054	6	8	2	7	1.41421	11	15	4	13	2.82843	2	3	1	2.5	0.70711	158.925	208.98	50.055	183.953	35.3943	1.6	1.75	0.15	1.68	0.10607	26	87	61	56.5	43.1335	1.15E-06	1.30E-06	1.50E-07	1.23E-06	1.06E-07	1790	2620	830	2205	586.899	8	11	3	9.5	2.12132	544.4	1629	1084.6	1086.7	766.928	31	38.7	7.7	34.85	5.44472	1.03E-05	1.34E-05	3.10E-06	1.19E-05	2.19E-06	63.1959	225	4.47097	4.47097	4.47097	1.5708	2.0944	2.0944	-0.5707
1	2.2	1.2	1.6	0.84853	4	5	1	4.5	0.70711	2	10	8	6	5.65685	1	3	2	2	1.41421	40.078	106.42	66.342	73.249	46.9109	1.4	1.8	0.4	1.6	0.28284	16	69	53	42.5	37.4767	3.40E-08	1.08E-07	7.40E-08	7.10E-08	5.23E-08	3070	3810	740	3440	523.259	72	200	128	136	90.5097	1115	1828.05	713.05	1471.53	504.202	17	180	163	98.5	115.258	1.18E-05	2.23E-05	1.05E-05	1.71E-05	7.42E-06	43.4657	221	3.516	3.516	3.516	1.5708	1.5708	1.5708	-0.6884
1.1	1.9	0.8	1.5	0.56569	4	8	4	6	2.82843	11	11	0	11	0	3	3	0	3	0	63.546	158.925	95.3794	111.236	67.4434	1.35	1.75	0.4	1.55	0.28284	26	72	46	49	32.5269	1.72E-08	1.15E-06	1.13E-06	5.84E-07	8.01E-07	2620	3570	950	3095	671.751	11	400	389	205.5	275.065	1357.77	1629	271.23	1493.39	191.789	38.7	140	101.3	89.35	71.6299	1.03E-05	1.65E-05	6.20E-06	1.34E-05	4.38E-06	42.2499	123	3.47702	3.47702	3.49472	1.5708	1.5708	1.5708	0.08978
1.12	1.69	0.57	1.405	0.40305	5	8	3	6.5	2.12132	4	12	8	8	5.65685	3	3	0	3	0	112.411	140.116	27.705	126.264	19.5904	1.55	1.85	0.3	1.7	0.21213	32	75	43	53.5	30.4056	7.00E-08	7.40E-07	6.70E-07	4.05E-07	4.74E-07	2100	2310	210	2205	148.492	11	97	86	54	60.8112	594.22	1068	473.78	831.11	335.013	22	42	20	32	14.1421	6.30E-06	3.08E-05	2.45E-05	1.86E-05	1.73E-05	58.704	221	3.88648	3.88648	3.88648	1.5708	1.5708	1.5708	-0.3249
1.25	1.9	0.65	1.575	0.45962	4	8	4	6	2.82843	11	15	4	13	2.82843	3	3	0	3	0	63.546	168.934	105.388	116.24	74.5207	1.35	1.75	0.4	1.55	0.28284	21	72	51	46.5	36.0624	1.72E-08	6.76E-07	6.59E-07	3.47E-07	4.66E-07	0	0	0	0	0	17	400	383	208.5	270.822	1357.77	1818	460.23	1587.89	325.432	45	140	95	92.5	67.1751	1.33E-05	1.65E-05	3.20E-06	1.49E-05	2.26E-06	39.867	221	3.41616	3.41616	3.41616	1.5708	1.5708	1.5708	-0.2909
1.61	1.91	0.3	1.76	0.21213	3	4	1	3.5	0.70711	10	13	3	11.5	2.12132	2	3	1	2.5	0.70711	26.9815	58.6934	31.7119	42.8375	22.4237	1.25	1.35	0.1	1.3	0.07071	67	80	13	73.5	9.19239	2.70E-08	7.20E-08	4.50E-08	4.95E-08	3.18E-08	4970	5100	130	5035	91.9239	91	235	144	163	101.823	933.47	1728	794.53	1330.74	561.818	76	180	104	128	73.5391	1.34E-05	2.31E-05	9.70E-06	1.83E-05	6.86E-06	24.0552	221	2.88671	2.88671	2.88671	1.5708	1.5708	1.5708	-0.6694
1.1	2	0.9	1.55	0.6364	6	8	2	7	1.41421	12	16	4	14	2.82843	3	3	0	3	0	173.04	200.59	27.55	186.815	19.4808	1.5	1.75	0.25	1.63	0.17678	17	74	57	45.5	40.3051	2.50E-07	9.60E-07	7.10E-07	6.05E-07	5.02E-07	1407	1590	183	1498.5	129.401	8.3	39	30.7	23.65	21.7082	234.32	1097	862.68	665.66	610.007	25	31	6	28	4.24264	0	0	0	0	0	51.578	221	3.72239	3.72239	3.72239	1.5708	1.5708	1.5708	-0.6198
1.63	2.55	0.92	2.09	0.65054	2	4	2	3	1.41421	5	14	9	9.5	6.36396	2	3	1	2.5	0.70711	12.0107	50.9415	38.9308	31.4761	27.5282	0.7	1.35	0.65	1.03	0.45962	54	95	41	74.5	28.9914	2.00E-07	1.00E-05	9.80E-06	5.10E-06	6.93E-06	4560	18350	13790	11455	9751	31	140	109	85.5	77.0746	2183	3800	1617	2991.5	1143.39	33	160	127	96.5	89.8026	7.10E-06	8.40E-06	1.30E-06	7.75E-06	9.19E-07	17.8703	225	2.9346	2.9346	2.9346	1.5708	2.0944	2.0944	-0.3924
1.66	2.1	0.44	1.88	0.31113	4	5	1	4.5	0.70711	6	16	10	11	7.07107	2	3	1	2.5	0.70711	51.9961	127.6	75.6039	89.798	53.46	1.4	1.4	0	1.4	0	57	92	35	74.5	24.7487	1.27E-07	0.0001	9.99E-05	5.01E-05	7.06E-05	2610	5940	3330	4275	2354.67	3	94	91	48.5	64.3467	722.66	2180	1457.34	1451.33	1030.49	65	160	95	112.5	67.1751	0	0	0	0	0	60.2157	216	4.39955	4.39955	4.39955	1.5708	2.0944	2.0944	0.11059
0.82	2.96	2.14	1.89	1.51321	4	4	0	4	0	1	17	16	9	11.3137	1	2	1	1.5	0.70711	39.0983	79.904	40.8057	59.5011	28.854	1.15	2.2	1.05	1.68	0.74246	10	98	88	54	62.2254	7.50E-08	1.00E+18	1.00E+18	5.00E+17	7.07E+17	0	0	0	0	0	0.12	100	99.88	50.06	70.6258	265.8	336.53	70.73	301.165	50.0137	1.9	3.1	1.2	2.5	0.84853	0	0	0	0	0	65.7632	221	4.0364	4.0364	4.0364	1.5708	1.5708	1.5708	-1.8555
1.61	3.44	1.83	2.525	1.29401	2	3	1	2.5	0.70711	13	16	3	14.5	2.12132	2	2	0	2	0	15.9994	26.9815	10.9821	21.4905	7.76554	0.6	1.25	0.65	0.93	0.45962	80	101	21	90.5	14.8492	0	0	0	0	0	317.5	5100	4782.5	2708.75	3381.74	0.02658	235	234.973	117.513	166.151	54.8	933.47	878.67	494.135	621.314	0	0	0	0	0	0	0	0	0	0	21.8193	225	3.13655	3.13655	3.13655	1.5708	2.0944	2.0944	-1.4156

1.2	2.33	1.13	1.765	0.79903	6	8	2	7	1.41421	9	14	5	11.5	3.53553	2	3	1	2.5	0.70711	151.964	207.2	55.236	179.582	39.0578	1.8	1.85	0.05	1.83	0.03536
1.6	2.2	0.6	1.9	0.42426	5	5	0	5	0	5	8	3	6.5	2.12132	3	3	0	3	0	92.9064	101.07	8.16362	96.9882	5.77255	1.3	1.45	0.15	1.38	0.10607
1.12	1.65	0.53	1.385	0.37477	4	8	4	6	2.82843	4	12	8	8	5.65685	3	3	0	3	0	65.409	140.116	74.707	102.763	52.8258	1.35	1.85	0.5	1.6	0.35355
1.14	2.58	1.44	1.86	1.01823	3	8	5	5.5	3.53553	6	16	10	11	7.07107	2	3	1	2.5	0.70711	32.065	144.242	112.177	88.1535	79.3211	1	1.85	0.85	1.43	0.60104
2.2	3.04	0.84	2.62	0.59397	2	5	3	3.5	2.12132	8	15	7	11.5	4.94975	2	3	1	2.5	0.70711	14.0067	101.07	87.0633	57.5383	61.563	0.65	1.3	0.65	0.98	0.45962
1.22	2.54	1.32	1.88	0.93338	5	6	1	5.5	0.70711	3	11	8	7	5.65685	3	3	0	3	0	88.9059	196.967	108.061	142.936	76.4105	1.35	1.8	0.45	1.58	0.3182
1.36	3.44	2.08	2.4	1.47078	2	9	7	5.5	4.94975	7	16	9	11.5	6.36396	2	3	1	2.5	0.70711	15.9994	237	221.001	126.5	156.271	0.6	1.75	1.15	1.18	0.81317
1.55	2.1	0.55	1.825	0.38891	4	5	1	4.5	0.70711	7	16	9	11.5	6.36396	2	3	1	2.5	0.70711	54.938	127.6	72.662	91.269	51.3798	1.4	1.4	0	1.4	0
0.93	3.98	3.05	2.455	2.15668	2	3	1	2.5	0.70711	1	17	16	9	11.3137	1	2	1	1.5	0.70711	18.9984	22.9898	3.99137	20.9941	2.82232	0.5	1.8	1.3	1.15	0.91924
1.2	2.33	1.13	1.765	0.79903	6	8	2	7	1.41421	9	14	5	11.5	3.53553	2	3	1	2.5	0.70711	151.964	207.2	55.236	179.582	39.0578	1.8	1.85	0.05	1.83	0.03536
1.66	2.55	0.89	2.105	0.62933	2	4	2	3	1.41421	6	14	8	10	5.65685	2	3	1	2.5	0.70711	12.0107	51.9961	39.9854	32.0034	28.2739	0.7	1.4	0.7	1.05	0.49497
1.69	2.58	0.89	2.135	0.62933	3	5	2	4	1.41421	12	16	4	14	2.82843	2	3	1	2.5	0.70711	32.065	112.411	80.346	72.238	56.8132	1	1.55	0.55	1.28	0.38891
1.9	3.16	1.26	2.53	0.89095	3	4	1	3.5	0.70711	11	17	6	14	4.24264	2	3	1	2.5	0.70711	35.453	63.546	28.093	49.4995	19.8648	1	1.35	0.35	1.18	0.24749
1.14	1.65	0.51	1.395	0.36062	4	8	4	6	2.82843	6	12	6	9	4.24264	3	3	0	3	0	65.409	144.242	78.833	104.826	55.7433	1.35	1.85	0.5	1.6	0.35355
2.54	3.04	0.5	2.79	0.35355	2	6	4	4	2.82843	11	15	4	13	2.82843	2	3	1	2.5	0.70711	14.0067	196.967	182.96	105.487	129.372	0.65	1.35	0.7	1	0.49497
1.55	2.2	0.65	1.875	0.45962	4	6	2	5	1.41421	7	9	2	8	1.41421	3	3	0	3	0	54.938	192.217	137.279	123.578	97.0709	1.35	1.4	0.05	1.38	0.03536
1.54	2.04	0.5	1.79	0.35355	2	4	2	3	1.41421	4	13	9	8.5	6.36396	2	3	1	2.5	0.70711	10.811	47.867	37.056	29.339	26.2025	0.85	1.4	0.55	1.13	0.38891
1.36	1.61	0.25	1.485	0.17678	3	4	1	3.5	0.70711	3	13	10	8	7.07107	2	3	1	2.5	0.70711	26.9815	44.9559	17.9744	35.9687	12.7098	1.25	1.6	0.35	1.43	0.24749
1.61	2.28	0.67	1.945	0.47376	3	5	2	4	1.41421	9	13	4	11	2.82843	2	3	1	2.5	0.70711	26.9815	102.906	75.924	64.9435	53.6863	1.25	1.35	0.1	1.3	0.07071
1.22	2.28	1.06	1.75	0.74953	5	8	3	6.5	2.12132	9	12	3	10.5	2.12132	3	3	0	3	0	102.906	162.5	59.5945	132.703	42.1397	1.35	1.75	0.4	1.55	0.28284
1.12	2.18	1.06	1.65	0.74953	4	8	4	6	2.82843	4	15	11	9.5	7.77817	2	3	1	2.5	0.70711	74.9216	140.116	65.1944	107.519	46.0994	1.15	1.85	0.7	1.5	0.49497
1.6	2.2	0.6	1.9	0.42426	5	6	1	5.5	0.70711	5	9	4	7	2.82843	3	3	0	3	0	92.9064	192.217	99.3106	142.562	70.2232	1.35	1.45	0.1	1.4	0.07071
1.9	2.96	1.06	2.43	0.74953	4	4	0	4	0	11	17	6	14	4.24264	2	3	1	2.5	0.70711	63.546	79.904	16.358	71.725	11.5669	1.15	1.35	0.2	1.25	0.14142
1.61	2.05	0.44	1.83	0.31113	3	5	2	4	1.41421	13	15	2	14	1.41421	2	2	0	2	0	26.9815	121.76	94.7785	74.3708	67.0185	1.25	1.45	0.2	1.35	0.14142
0.82	0.82	0	0.82	0	4	4	0	4	0	1	1	0	1	0	1	1	0	1	0	39.0983	39.0983	0	39.0983	0	2.2	2.2	0	2.2	0
1.25	2.02	0.77	1.635	0.54447	6	8	2	7	1.41421	15	15	0	15	0	2	3	1	2.5	0.70711	168.934	208.98	40.0462	188.957	28.3169	1.6	1.75	0.15	1.68	0.10607
1.22	2.02	0.8	1.62	0.56569	5	6	1	5.5	0.70711	3	15	12	9	8.48528	2	3	1	2.5	0.70711	88.9059	208.98	120.075	148.943	84.9055	1.6	1.8	0.2	1.7	0.14142
2.2	3.04	0.84	2.62	0.59397	2	5	3	3.5	2.12132	8	15	7	11.5	4.94975	2	3	1	2.5	0.70711	14.0067	101.07	87.0633	57.5383	61.563	0.65	1.3	0.65	0.98	0.45962
0.95	0.95	0	0.95	0	5	5	0	5	0	2	2	0	2	0	1	1	0	1	0	87.62	87.62	0	87.62	0	2	2	0	2	0
1.2	1.69	0.49	1.445	0.34648	5	8	3	6.5	2.12132	10	12	2	11	1.41421	3	3	0	3	0	112.411	157.25	44.839	134.831	31.706	1.55	1.8	0.25	1.68	0.17678
1.54	1.9	0.36	1.72	0.25456	4	6	2	5	1.41421	4	7	3	5.5	2.12132	3	3	0	3	0	47.867	186.207	138.34	117.037	97.8212	1.35	1.4	0.05	1.38	0.03536
1.9	2.2	0.3	2.05	0.21213	4	5	1	4.5	0.70711	10	11	1	10.5	0.70711	3	3	0	3	0	63.546	106.42	42.874	84.983	30.3165	1.35	1.4	0.05	1.38	0.03536
1.13	2.02	0.89	1.575	0.62933	6	8	2	7	1.41421	5	15	10	10	7.07107	2	3	1	2.5	0.70711	140.908	208.98	68.0728	174.944	48.1347	1.6	1.85	0.25	1.73	0.17678
2.01	2.1	0.09	2.055	0.06364	4	5	1	4.5	0.70711	14	16	2	15	1.41421	2	2	0	2	0	72.64	127.6	54.96	100.12	38.8626	1.25	1.4	0.15	1.33	0.10607
2.36	2.55	0.19	2.455	0.13435	2	6	4	4	2.82843	6	14	8	10	5.65685	2	3	1	2.5	0.70711	12.0107	183.84	171.829	97.9254	121.502	0.7	1.35	0.65	1.03	0.45962
2.16	2.55	0.39	2.355	0.27577	2	5	3	3.5	2.12132	6	14	8	10	5.65685	2	3	1	2.5	0.70711	12.0107	95.94	83.9293	53.9754	59.347	0.7	1.45	0.75	1.08	0.53033
1.36	2.05	0.69	1.705	0.4879	4	5	1	4.5	0.70711	3	15	12	9	8.48528	2	3	1	2.5	0.70711	44.9559	121.76	76.8041	83.358	54.3087	1.45	1.6	0.15	1.53	0.10607
1.2	2.55	1.35	1.875	0.95459	4	8	4	6	2.82843	9	16	7	12.5	4.94975	2	3	1	2.5	0.70711	78.96	151.964	73.004	115.462	51.6216	1.15	1.85	0.7	1.5	0.49497
1.36	2.55	1.19	1.955	0.84146	2	4	2	3	1.41421	3	14	11	8.5	7.77817	2	3	1	2.5	0.70711	12.0107	44.9559	32.9452	28.4833	23.2958	0.7	1.6	0.9	1.15	0.6364
1.93	2.55	0.62	2.24	0.43841	2	5	3	3.5	2.12132	11	14	3	12.5	2.12132	2	3	1	2.5	0.70711	12.0107	107.868	95.8575	59.9395	67.7815	0.7	1.6	0.9	1.15	0.6364
1.2	1.62	0.42	1.41	0.29698	6	8	2	7	1.41421	9	13	4	11	2.82843	2	3	1	2.5	0.70711	151.964	204.383	52.4193	178.174	37.066	1.85	1.9	0.05	1.88	0.03536
1.3	2.55	1.25	1.925	0.88388	2	9	7	5.5	4.94975	4	14	10	9	7.07107	2	3	1	2.5	0.70711	12.0107	232.038	220.027	122.024	155.583	0.7	1.8	1.1	1.25	0.77782
1.96	2.18	0.22	2.07	0.15556	4	5	1	4.5	0.70711	14	15	1	14.5	0.70711	2	2	0	2	0	74.9216	118.71	43.7884	96.8158	30.9631	1.15	1.45	0.3	1.3	0.21213

1.65	1.93	0.28	1.79	0.19799	4	5	1	4.5	0.70711	11	12	1	11.5	0.70711	3	3
1.13	3.04	1.91	2.085	1.35057	2	8	6	5	4.24264	5	15	10	10	7.07107	2	3
1.24	3.04	1.8	2.14	1.27279	2	8	6	5	4.24264	14	15	1	14.5	0.70711	2	3
2.1	2.18	0.08	2.14	0.05657	4	5	1	4.5	0.70711	15	16	1	15.5	0.70711	2	2
1.1	1.65	0.55	1.375	0.38891	4	8	4	6	2.82843	3	12	9	7.5	6.36396	3	3
1.9	2.66	0.76	2.28	0.5374	4	5	1	4.5	0.70711	11	17	6	14	4.24264	2	3
1.55	3.04	1.49	2.295	1.05359	2	4	2	3	1.41421	7	15	8	11	5.65685	2	3
1.78	2.05	0.27	1.915	0.19092	5	5	0	5	0	13	15	2	14	1.41421	2	2
2	2.1	0.1	2.05	0.07071	5	6	1	5.5	0.70711	12	16	4	14	2.82843	2	3
1.22	1.61	0.39	1.415	0.27577	3	5	2	4	1.41421	3	13	10	8	7.07107	2	3
0.98	2.28	1.3	1.63	0.91924	2	5	3	3.5	2.12132	1	9	8	5	5.65685	1	3
1.2	2.1	0.9	1.65	0.6364	5	8	3	6.5	2.12132	10	16	6	13	4.24264	2	3
1.91	2	0.09	1.955	0.06364	4	6	2	5	1.41421	10	12	2	11	1.41421	3	3
1.2	2.1	0.9	1.65	0.6364	5	8	3	6.5	2.12132	9	16	7	12.5	4.94975	2	3
1.55	2.58	1.03	2.065	0.72832	3	4	1	3.5	0.70711	7	16	9	11.5	6.36396	2	3
1.63	2.2	0.57	1.915	0.40305	4	5	1	4.5	0.70711	5	8	3	6.5	2.12132	3	3
0.93	3.16	2.23	2.045	1.57685	3	3	0	3	0	1	17	16	9	11.3137	1	2
1.28	2.55	1.27	1.915	0.89803	4	9	5	6.5	3.53553	8	16	8	12	5.65685	2	3
1.36	2.18	0.82	1.77	0.57983	4	9	5	6.5	3.53553	7	15	8	11	5.65685	2	3
1.57	2.28	0.71	1.925	0.50205	2	5	3	3.5	2.12132	2	9	7	5.5	4.94975	1	3
1.61	1.96	0.35	1.785	0.24749	3	5	2	4	1.41421	13	14	1	13.5	0.70711	2	2
1.33	2.1	0.77	1.715	0.54447	5	5	0	5	0	4	16	12	10	8.48528	2	3

1.78	3.04	1.26	2.41	0.89095	2	5	3	3.5	2.12132	13	15	2	14	1.41421	2	2	0	[illegible]
0.98	3.04	2.06	1.89	1.05076	2	4	2	2.66667	1.1547	1	15	14	9.33333	7.37111	1	3	2	[illegible]
1.1	2.28	1.18	1.85667	0.65684	3	8	5	5.66667	2.51661	10	15	5	12	2.64575	2	3	1	[illegible]
1.54	2.28	0.74	1.78667	0.52326	4	5	1	4.33333	0.70711	4	9	5	5.66667	3.53553	3	3	0	[illegible]
1.36	2.04	0.68	1.81333	0.48083	2	4	2	2.66667	1.41421	3	13	10	9.66667	7.07107	2	3	1	[illegible]
1.3	2.2	0.9	1.9	0.6364	1	9	8	3.66667	5.65685	1	4	3	2	2.12132	1	3	2	[illegible]
1.96	3.44	1.48	2.94667	1.04652	2	5	3	3	2.12132	14	16	2	15.3333	1.41421	2	2	0	[illegible]
1.66	1.9	0.24	1.82	0.16971	3	4	1	3.33333	0.70711	6	14	8	11.3333	5.65685	2	3	1	[illegible]
1.62	2.55	0.93	2.085	0.65761	2	6	4	4	2.82843	13	14	1	13.5	0.70711	2	2	0	[illegible]
1.61	2.04	0.43	1.89667	0.30406	2	3	1	2.33333	0.70711	13	13	0	13	0	2	2	0	[illegible]
1.1	1.93	0.83	1.515	0.5869	5	8	3	6.5	2.12132	3	11	8	7	5.65685	3	3	0	[illegible]
1.3	2	0.7	1.53333	0.49497	6	6	0	6	0	4	12	8	6.66667	5.65685	3	3	0	[illegible]
1.62	2.02	0.4	1.88667	0.28284	6	6	0	6	0	13	15	2	14.3333	1.41421	2	2	0	[illegible]
0.98	3.44	2.46	3.0387	1.10459	2	8	6	2.26087	1.70015	1	16	15	14	6.37399	1	3	2	[illegible]
1.78	2.04	0.26	1.92793	0.1007	2	5	3	3.65517	1.34784	10	13	3	10.8276	2.04939	2	3	1	[illegible]
0.93	2.58	1.65	1.73944	0.93309	2	4	2	2.94444	0.63317	1	16	15	8	8.7116	1	3	2	[illegible]
[illegible]	[illegible]	[illegible]	[illegible]	[illegible]	[illegible]	[illegible]	[illegible]	[illegible]	[illegible]	[illegible]	[illegible]	[illegible]	[illegible]	[illegible]	[illegible]	[illegible]	[illegible]	[illegible]

1.3	2.2	0.9	1.82	0.46605	5	6	1	5.33333	0.57735	4	14	10	[illegible]
0.93	2	1.07	1.64333	0.7566	3	6	3	5	2.12132	1	12	11	[illegible]
1.9	2.2	0.3	2	0.21213	3	5	2	3.66667	1.41421	8	14	6	[illegible]
1.22	2.02	0.8	1.62	0.56569	6	8	2	7	1.41421	12	15	3	[illegible]
1.81	2.54	0.73	2.05333	0.51619	4	6	2	4.66667	1.41421	11	13	2	[illegible]
1.1	1.93	0.83	1.65333	0.5869	5	8	3	6	2.12132	11	11	0	[illegible]
1.36	2.28	0.92	1.86667	0.46705	4	6	2	5	1	3	14	11	[illegible]
1.3	1.9	0.6	1.7	0.42426	3	9	6	5	4.24264	4	14	10	[illegible]
1.33	1.93	0.6	1.53	0.42426	5	5	0	5	0	4	11	7	[illegible]
1.24	2.02	0.78	1.63	0.55154	6	8	2	7	1.41421	14	15	1	[illegible]
1.1	1.31	0.21	1.17	0.14849	3	8	5	6.33333	3.53553	2	11	9	[illegible]
1.9	2.36	0.46	2.05333	0.32527	3	6	3	4	2.12132	6	14	8	[illegible]
1.22	2.54	1.32	2.03	0.7093	6	8	2	6.66667	1.1547	11	14	3	[illegible]
1.36	1.36	0	1.36	0	4	4	0	4	0	3	3	0	[illegible]
1.36	2.02	0.66	1.76333	0.3536	4	6	2	4.66667	1.1547	3	15	12	[illegible]
0.89	1.9	1.01	1.56333	0.71418	3	6	3	4	2.12132	2	14	12	[illegible]
[illegible]													

1.22	2.55	1.33	2.10667	0.94045	2	5	3	3	2.12132	3	14	11	10.3333	7.77817	2	3	1	2.33	0.70711	12.0107	88.9059	76.8952	37.6424
1.22	2.05	0.83	1.77333	0.5869	5	8	3	6	2.12132	12	15	3	14	2.12132	2	3	1	2.33	0.70711	121.76	162.5	40.74	135.34
0.82	2.55	1.73	1.39667	1.22329	4	5	1	4.66667	0.70711	1	16	15	6	10.6066	1	2	1	1.33	0.70711	78.96	85.4678	6.5078	83.2985
1.9	2.55	0.65	2.11667	0.45962	4	4	0	4	0	11	16	5	12.6667	3.53553	2	3	1	2.67	0.70711	63.546	78.96	15.414	68.684
1.33	2.2	0.87	1.62	0.61518	5	5	0	5	0	4	10	6	6	4.24264	3	3	0	3	0	91.224	106.42	15.196	96.2893
1.2	2.54	1.34	1.9	0.67201	5	8	3	6.33333	1.52753	10	14	4	11.6667	2.08167	2	3	1	2.67	0.57735	118.71	196.967	78.2566	157.642
0.89	3.44	2.55	2.59	1.80312	2	6	4	3.33333	2.82843	2	16	14	11.3333	9.89949	1	2	1	1.67	0.70711	15.9994	137.327	121.328	56.4419
1.1	1.81	0.71	1.57333	0.50205	4	8	4	5.33333	2.82843	3	13	10	9.66667	7.07107	2	3	1	2.33	0.70711	69.723	138.905	69.1825	92.7838
1.1	2.55	1.45	2.06667	1.0253	2	8	6	4	4.24264	14	16	2	14.6667	1.41421	2	3	1	2.33	0.70711	12.0107	173.04	161.029	65.6871
0.89	1.9	1.01	1.56333	0.71418	3	6	3	4	2.12132	2	14	12	10	8.48528	1	2	1	1.67	0.70711	28.0855	137.327	109.242	64.4993
1.54	1.93	0.39	1.6375	0.27577	4	5	1	4.25	0.70711	4	11	7	5.75	4.94975	3	3	0	3	0	47.867	107.868	60.0012	62.8673
2.2	3.04	0.84	2.59667	0.42194	1	2	1	1.66667	0.57735	1	15	14	10	7.81025	1	2	1	1.67	0.57735	1.00794	14.0067	12.9988	9.00845
1.36	2.05	0.69	1.77333	0.36474	4	5	1	4.33333	0.57735	3	15	12	9.33333	6.02771	2	3	1	2.67	0.57735	44.9559	121.76	76.8041	75.1364
2.36	2.58	0.22	2.50667	0.15556	3	6	3	4	2.12132	6	16	10	12.6667	7.07107	2	3	1	2.33	0.70711	32.065	183.84	151.775	82.6567
1.23	2.04	0.81	1.77	0.57276	2	8	6	4	4.24264	13	13	0	13	0	2	3	1	2.33	0.70711	10.811	164.93	154.119	62.1841
1.3	1.81	0.51	1.64	0.36062	4	9	5	5.66667	3.53553	4	13	9	10	6.36396	2	3	1	2.33	0.70711	69.723	232.038	162.315	123.828
1.2	2.55	1.35	2.1	0.95459	2	8	6	4	4.24264	10	14	4	12.6667	2.82843	2	3	1	2.33	0.70711	12.0107	157.25	145.239	60.4238
1.83	2.01	0.18	1.89	0.12728	4	4	0	4	0	8	14	6	10	4.24264	2	3	1	2.67	0.70711	55.845	72.64	16.795	61.4433
1.31	1.96	0.65	1.72333	0.35921	3	5	2	4	1	2	14	12	9	6.245	1	3	2	2	1	24.305	118.71	94.405	68.8537
1.3	2.28	0.98	1.84667	0.49973	5	6	1	5.66667	0.57735	4	14	10	9.33333	5.03322	2	3	1	2.67	0.57735	118.71	195.084	76.374	164.095
1.17	2.54	1.37	1.89	0.68768	5	8	3	6.33333	1.52753	8	14	6	11	3	2	3	1	2.67	0.57735	118.71	196.967	78.2566	155.346
1.54	2.2	0.66	1.98	0.46669	4	5	1	4.66667	0.70711	4	10	6	8	4.24264	3	3	0	3	0	47.867	106.42	58.553	86.9023
1.24	1.81	0.57	1.62	0.40305	4	8	4	5.33333	2.82843	13	14	1	13.3333	0.70711	2	3	1	2.33	0.70711	69.723	167.259	97.536	102.235
0.98	2.2	1.22	1.485	0.66582	2	5	3	3.5	1.89737	1	13	12	6.25	6.77495	1	3	2	1.75	1.04881	6.941	114.818	107.877	58.78
1.22	2.05	0.83	1.72667	0.44433	4	5	1	4.66667	0.57735	3	15	12	9.33333	6.02771	2	3	1	2.67	0.57735	58.6934	121.76	63.0666	89.7864
0.79	3.44	2.65	1.67333	1.87383	2	6	4	4.66667	2.82843	1	16	15	6	10.6066	1	2	1	1.33	0.70711	15.9994	132.905	116.906	93.9368
1.36	3.44	2.08	2.74667	1.47078	2	9	7	4.33333	4.94975	7	16	9	13	6.36396	2	3	1	2.33	0.70711	15.9994	237	221.001	89.6663
1.31	3.04	1.73	2.485	0.89463	2	3	1	2.25	0.54772	2	15	13	11.5	6.95701	1	2	1	1.75	0.54772	12.0107	24.305	12.2943	16.0823
[illegible]																							

1.6	1.69	0.09	1.6675	0.06364	5	5	0	5	0	5	12	7	10.25	4.94975	3	3	0	3	0	92.9064	112.411	19.5046	107.535	13.7918	1.45	1.55	0.1	1.53
1.1	1.78	0.68	1.61	0.48083	5	8	3	5.75	2.12132	11	13	2	12.5	1.41421	2	3	1	2.25	0.70711	114.818	158.925	44.1074	125.845	31.1886	1.55	1.75	0.2	1.6
1.36	2.2	0.84	1.99	0.59397	4	6	2	5.5	1.41421	3	9	6	7.5	4.24264	3	3	0	3	0	44.9559	192.217	147.261	155.402	104.129	1.35	1.6	0.25	1.41
0.82	3.04	2.22	2.485	1.56978	2	5	3	2.75	2.12132	1	15	14	11.5	9.89949	1	2	1	1.75	0.70711	14.0067	85.4678	71.4611	31.872	50.5306	0.65	2.35	1.7	1.08
1.78	2.02	0.24	1.9	0.16971	5	6	1	5.5	0.70711	13	15	2	14	1.41421	2	2	0	2	0	114.818	208.98	94.1624	161.899	66.5829	1.55	1.6	0.05	1.58
2.2	3.16	0.96	2.68	0.67882	1	3	2	2	1.41421	1	17	16	9	11.3137	1	2	1	1.5	0.70711	1.00794	35.453	34.4451	18.2305	24.3563	0.25	1	0.75	0.63
2.16	3.04	0.88	2.6	0.62225	2	5	3	3.5	2.12132	6	15	9	10.5	6.36396	2	3	1	2.5	0.70711	14.0067	95.94	81.9333	54.9733	57.9356	0.65	1.45	0.8	1.05
0.89	2.2	1.31	1.65	0.56101	1	6	5	3.25	2.06155	1	14	13	7.5	6.95222	1	2	1	1.5	0.57735	1.00794	137.327	136.319	48.3505	60.623	0.25	2.15	1.9	1.19
1.61	2.28	0.67	2.1125	0.47376	3	6	3	5.25	2.12132	10	13	3	10.75	2.12132	2	3	1	2.75	0.70711	26.9815	195.084	168.102	153.058	118.866	1.25	1.35	0.1	1.33
1.78	1.9	0.12	1.84	0.08485	4	5	1	4.5	0.70711	11	13	2	12	1.41421	2	3	1	2.5	0.70711	63.546	114.818	51.272	89.182	36.2548	1.35	1.55	0.2	1.45
2.36	3.04	0.68	2.81333	0.48083	2	6	4	3.33333	2.82843	6	15	9	12	6.36396	2	3	1	2.33	0.70711	14.0067	183.84	169.833	70.6178	120.09	0.65	1.35	0.7	0.88
1.61	3.16	1.55	2.7725	1.09602	3	3	0	3	0	13	17	4	16	2.82843	2	2	0	2	0	26.9815	35.453	8.47146	33.3351	5.99023	1	1.25	0.25	1.06
1.66	2.58	0.92	2.12	0.65054	3	4	1	3.5	0.70711	6	16	10	11	7.07107	2	3	1	2.5	0.70711	32.065	51.9961	19.9311	42.0306	14.0934	1	1.4	0.4	1.2
1.55	1.63	0.08	1.585	0.04517	3	4	1	3.75	0.54772	5	13	8	8	3.79473	2	3	1	2.75	0.54772	26.9815	54.938	27.9565	46.9498	14.7281	1.25	1.4	0.15	1.35
1.55	1.63	0.08	1.585	0.04517	3	4	1	3.75	0.54772	5	13	8	8	3.79473	2	3	1	2.75	0.54772	26.9815	54.938	27.9565	46.9498	14.7281	1.25	1.4	0.15	1.35
0.98	3.98	3	2.785	1.60702	1	2	1	1.75	0.54772	1	17	16	9	10.1193	1	2	1	1.5	0.63246	1.00794	18.9984	17.9905	11.4864	9.86548	0.25	1.45	1.2	0.68
1.69	3.44	1.75	2.6425	1.01825	2	6	4	3.75	2.25832	12	16	4	14	2.52982	2	3	1	2.5	0.63246	15.9994	200.59	184.591	86.25	97.218	0.6	1.55	0.95	1.06
0.98	2.2	1.22	1.6125	0.56494	2	5	3	3.75	1.5	1	14	13	6.75	6.29153	1	3	2	1.75	0.95743	6.941	118.71	111.769	64.094	56.6393	1.4	1.5	0.1	1.45
1.38	2.28	0.9	2.055	0.6364	5	9	4	6	2.82843	6	9	3	8.25	2.12132	3	3	0	3	0	102.906	238.029	135.123	136.686	95.5467	1.35	1.75	0.4	1.45
0.89	3.44	2.55	2.18	1.59401	2	6	4	3.75	2.25832	2	16	14	9	8.85438	1	2	1	1.5	0.63246	15.9994	137.327	121.328	64.2364	64.9389	0.6	2.15	1.55	1.34
0.89	3.44	2.55	2.1925	1.57874	2	6	4	3.5	2.09762	2	16	14	9	8.85438	1	2	1	1.5	0.63246	15.9994	137.327	121.328	52.351	63.2912	0.6	2.15	1.55	1.29
1.33	2.28	0.95	2.0425	0.67175	5	5	0	5	0	4	9	5	7.75	3.53553	3	3	0	3	0	91.224	102.906	11.6815	99.9851	8.26007	1.35	1.55	0.2	1.4
2.05	2.2	0.15	2.125	0.10607	5	5	0	5	0	10	15	5	12.5	3.53553	2	3	1	2.5	0.70711	106.42	121.76	15.34	114.09	10.847	1.4	1.45	0.05	1.42
1.12	1.62	0.5	1.495	0.35355	6	8	2	6.5	1.41421	4	13	9	10.75	6.36396	2	3	1	2.25	0.70711	140.116	204.383	64.2673	188.316	45.4438	1.85	1.9	0.05	1.89
1.24	1.81	0.57	1.6675	0.40305	4	8	4	5	2.82843	13	14	1	13.25	0.70711	2	3	1	2.25	0.70711	69.723	167.259	97.536	94.107	68.9684	1.3	1.75	0.45	1.41
0.93	3.04	2.11	1.4825	1.13773	2	3	1	2.25	0.54772	1	15	14	4.5	7.66812	1	2	1	1.25	0.54772	6.941	22.9898	16.0488	12.7196	8.34068	0.65	1.8	1.15	1.34
0.93	3.04	2.11	1.4825	1.13773	2	3	1	2.25	0.54772	1	15	14	4.5	7.66812	1	2	1	1.25	0.54772	6.941	22.9898	16.0488	12.7196	8.34068	0.65	1.8	1.15	1.34
1.93	2.1	0.17	2.045	0.08786	5	5	0	5	0	11	16	5	14.5	2.60768	2	3	1	2.25	0.54772	107.868	127.6	19.7318	121.207	10.1975	1.4	1.6	0.2	1.46
1.25	1.78	0.53	1.6475	0.37477	5	8	3	5.75	2.12132	13	15	2	13.5	1.41421	2	3	1	2.25	0.70711	114.818	168.934	54.1162	128.347	38.2659	1.55	1.75	0.2	1.6
1.83	2.2	0.37	2.1075	0.26163	4	5	1	4.75	0.70711	8	10	2	9.5	1.41421	3	3	0	3	0	55.845	106.42	50.575	93.7763	35.7619	1.4	1.4	0	1.4
1.1	1.78	0.68	1.61	0.48083	5	8	3	5.75	2.12132	13	16	3	13.75	2.12132	2	3	1	2.25	0.70711	114.818	173.04	58.222	129.374	41.1692	1.55	1.75	0.2	1.6
1.38	1.9	0.52	1.77	0.3677	3	9	6	4.5	4.24264	6	14	8	12	5.65685	2	3	1	2.25	0.70711	28.0855	238.029	209.943	80.5714	148.452	1.1	1.75	0.65	1.26

1.6	1.9	0.3	1.675	0.21213	3	5	2	4.5	1.41421	5	14	9	7.25	6.36396	2	3	1	2.75	0.70711	28.0855	92.9064	64.8209	76.7012	45.8353	1.1	1.45	0.35	1.36	0.24749
2.04	2.28	0.24	2.16	0.16971	2	5	3	3.5	2.12132	9	13	4	11	2.82843	2	3	1	2.5	0.70711	10.811	102.906	92.0945	56.8583	65.1206	0.85	1.35	0.5	1.1	0.35355
0.79	3.44	2.65	2.754	1.37294	2	6	4	3.4	2.32993	1	17	16	13.2	8.16788	1	2	1	1.8	0.53452	15.9994	132.905	116.906	61.5616	74.6118	0.6	2.6	2	1.16	1.04745
0.93	3.44	2.51	2.55	1.47639	2	6	4	3	2.0702	1	16	15	10.8	8.67673	1	3	2	2	0.84515	15.9994	180.948	164.948	50.3872	87.3095	0.6	1.8	1.2	1.01	0.68713
1.83	1.96	0.13	1.8975	0.05822	4	5	1	4.25	0.54772	8	14	6	11	2.68328	2	3	1	2.75	0.54772	55.845	118.71	62.865	75.4118	31.8697	1.35	1.45	0.1	1.39	0.05244
1.63	2.2	0.57	1.915	0.40305	4	6	2	5	1.41421	5	9	4	7	2.82843	3	3	0	3	0	50.9415	192.217	141.276	121.579	99.8969	1.35	1.35	0	1.35	0
0.82	2.55	1.73	1.955	0.89427	2	5	3	3.25	1.64317	1	14	13	10	6.75278	1	3	2	2	0.89443	12.0107	85.4678	73.4571	43.2588	40.7237	0.7	2.35	1.65	1.28	0.85381
1.9	2.96	1.06	2.43	0.74953	4	4	0	4	0	11	17	6	14	4.24264	2	3	1	2.5	0.70711	63.546	79.904	16.358	71.725	11.5669	1.15	1.35	0.2	1.25	0.14142
1.9	2.55	0.65	2.225	0.45962	4	4	0	4	0	11	16	5	13.5	3.53553	2	3	1	2.5	0.70711	63.546	78.96	15.414	71.253	10.8993	1.15	1.35	0.2	1.25	0.14142
1.14	1.31	0.17	1.2675	0.12021	3	8	5	4.25	3.53553	2	6	4	3	2.82843	1	3	2	1.5	1.41421	24.305	144.242	119.937	54.2892	84.8083	1.5	1.85	0.35	1.59	0.24749
1.5	1.91	0.41	1.8075	0.28991	4	6	2	4.5	1.41421	5	10	5	8.75	3.53553	3	3	0	3	0	58.6934	180.948	122.254	89.257	86.447	1.35	1.45	0.1	1.38	0.07071
0.89	2.2	1.31	1.6775	0.57973	1	6	5	3.5	2.08167	1	14	13	7.5	6.95222	1	2	1	1.5	0.57735	1.00794	137.327	136.319	59.4891	59.7452	0.25	2.15	1.9	1.23	0.77621
1.9	2.1	0.2	2	0.14142	4	5	1	4.5	0.70711	11	16	5	13.5	3.53553	2	3	1	2.5	0.70711	63.546	127.6	64.054	95.573	45.293	1.35	1.4	0.05	1.38	0.03536
1.93	2.1	0.17	2.045	0.08786	5	5	0	5	0	11	16	5	14.5	2.60768	2	3	1	2.25	0.54772	107.868	127.6	19.7318	121.207	10.1975	1.4	1.6	0.2	1.46	0.10368
0.98	2.2	1.22	1.7	0.56306	2	5	3	3.75	1.3784	1	13	12	9.25	6.22093	1	3	2	2	0.89443	6.941	106.42	99.479	63.2018	45.2466	1.3	1.45	0.15	1.36	0.08216
0.93	3.44	2.51	2.3575	1.40361	2	6	4	3.25	2.07364	1	16	15	11.5	7.82304	1	2	1	1.75	0.54772	15.9994	204.383	188.384	64.843	101.97	0.6	1.9	1.3	1.23	0.79183
1.36	1.81	0.45	1.6975	0.3182	4	9	5	5.25	3.53553	7	13	6	11.5	4.24264	2	3	1	2.25	0.70711	69.723	237	167.277	111.542	118.283	1.3	1.75	0.45	1.41	0.3182
1.54	2.05	0.51	1.795	0.36062	4	5	1	4.5	0.70711	4	15	11	9.5	7.77817	2	3	1	2.5	0.70711	47.867	121.76	73.893	84.8135	52.2502	1.4	1.45	0.05	1.42	0.03536
0.79	2.66	1.87	2.22	0.9706	5	6	1	5.4	0.65465	1	17	16	13.2	8.29802	1	2	1	1.8	0.53452	126.904	207.2	80.2955	144.164	42.2322	1.4	2.6	1.2	1.72	0.62335
1.9	1.96	0.06	1.9175	0.03146	4	5	1	4.25	0.54772	10	14	4	11.5	1.89737	2	3	1	2.75	0.54772	58.6934	118.71	60.0166	76.1238	31.2013	1.35	1.45	0.1	1.38	0.05477
1.54	2.2	0.66	1.87	0.46669	4	5	1	4.5	0.70711	4	10	6	7	4.24264	3	3	0	3	0	47.867	106.42	58.553	77.1435	41.4032	1.4	1.4	0	1.4	0
1.62	2.58	0.96	2.1175	0.58586	3	6	3	4.25	1.64317	12	16	4	14.25	2.25832	2	3	1	2.25	0.54772	32.065	204.383	172.318	95.2311	89.8649	1	1.9	0.9	1.36	0.48451
0.93	3.44	2.51	2.36	1.40151	2	4	2	2.75	1.04881	1	16	15	9.5	8.41427	1	3	2	2	0.89443	15.9994	50.9415	34.9421	26.4825	18.2234	0.6	1.8	1.2	1.09	0.64865
0.98	2.58	1.6	1.92	0.8716	2	4	2	3	0.89443	1	16	15	9.25	8.64292	1	3	2	2	0.89443	6.941	47.867	40.926	29.7345	18.5385	1	1.45	0.45	1.21	0.26972
1.3	2.54	1.24	1.92	0.87681	6	6	0	6	0	4	11	7	7.5	4.94975	3	3	0	3	0	178.49	196.967	18.4766	187.728	13.0649	1.35	1.55	0.2	1.45	0.14142
1.93	2.28	0.35	2.1925	0.24749	5	6	1	5.75	0.70711	10	11	1	10.25	0.70711	3	3	0	3	0	107.868	195.084	87.2158	173.28	61.6709	1.35	1.6	0.25	1.41	0.17678
1.9	3.44	1.54	2.92667	1.08894	2	4	2	2.66667	1.41421	11	16	5	14.3333	3.53553	2	3	1	2.33	0.70711	15.9994	63.546	47.5466	31.8483	33.6205	0.6	1.35	0.75	0.85	0.53033
1.61	2.55	0.94	2.1525	0.51926	3	4	1	3.75	0.54772	11	16	5	14	2.68328	2	3	1	2.25	0.54772	26.9815	78.96	51.9785	62.1119	26.862	1.15	1.35	0.2	1.23	0.10488
1.9	3.04	1.14	2.185	0.8061	2	6	4	5	2.82843	7	15	8	9	5.65685	2	3	1	2.75	0.70711	14.0067	186.207	172.2	143.157	121.764	0.65	1.35	0.7	1.18	0.49497
1.12	2.33	1.21	1.4225	0.8556	6	8	2	7.5	1.41421	4	14	10	6.5	7.07107	2	3	1	2.75	0.70711	140.116	207.2	67.084	156.887	47.4356	1.8	1.85	0.05	1.84	0.03536
1.54	1.91	0.37	1.725	0.26163	4	4	0	4	0	4	10	6	7	4.24264	3	3	0	3	0	47.867	58.6934	10.8264	53.2802	7.65542	1.35	1.4	0.05	1.38	0.03536
2.2	2.36	0.16	2.28	0.11314	6	6	0	6	0	6	9	3	7.5	2.12132	3	3	0	3	0	183.84	192.217	8.377	188.029	5.92343	1.35	1.35	0	1.35	0
1.63	2.55	0.92	2.09	0.65054	4	4	0	4	0	5	16	11	10.5	7.77817	2	3	1	2.5	0.70711	50.9415	78.96	28.0185	64.9508	19.8121	1.15	1.35	0.2	1.25	0.14142
1.83	2.28	0.45	2.075	0.26176	4	6	2	5	1.26491	8	10	2	9.5	1.09545	3	3	0	3	0	55.845	195.084	139.239	126.177	87.171	1.35	1.4	0.05	1.36	0.02739
0.98	2.33	1.35	1.9275	0.69521	2	6	4	4.5	1.89737	1	14	13	8.75	6.02495	1	3	2	2.25	1.04881	6.941	207.2	200.259	106.745	89.5595	1.4	1.8	0.4	1.51	0.21154
0.82	2.05	1.23	1.1825	0.63607	3	5	2	3.75	1.04881	1	15	14	4.5	7.66812	1	2	1	1.25	0.54772	22.9898	121.76	98.7702	51.7095	51.8296	1.45	2.2	0.75	1.81	0.33578
1.36	3.44	2.08	2.74	1.1791	2	6	4	3.2	2.13809	3	16	13	13.2	6.83478	2	3	1	2.2	0.53452	15.9994	208.98	192.981	60.3869	100.408	0.6	1.6	1	1	0.65465
1.23	2.28	1.05	2.022	0.54356	2	8	6	5	2.53546	9	13	4	10.6	2.61861	2	3	1	2.8	0.53452	10.811	164.93	154.119	96.8916	65.8669	0.85	1.75	0.9	1.33	0.38173
1.33	2.54	1.21	2.0475	0.65467	5	6	1	5.5	0.63246	4	13	9	9.75	4.32435	2	3	1	2.75	0.54772	91.224	196.967	105.743	149.994	60.346	1.35	1.55	0.2	1.45	0.12649
1.38	1.9	0.52	1.51	0.3677	3	9	6	7.5	4.24264	6	14	8	8	5.65685	2	3	1	2.75	0.70711	28.0855	238.029	209.943	185.543	148.452	1.1	1.75	0.65	1.59	0.45962
2.16	2.2	0.04	2.18	0.02828	1	5	4	3	2.82843	1	6	5	3.5	3.53553	1	3	2	2	1.41421	1.00794	95.94	94.9321	48.474	67.1271	0.25	1.45	1.2	0.85	0.84853
1.33	1.69	0.36	1.6	0.25456	5	5	0	5	0	4	12	8	10	5.65685	3	3	0	3	0	91.224	112.411	21.187	107.114	14.9815	1.55	1.55	0	1.55	0
0.98	2.28	1.3	1.5125	0.7056	2	5	3	3.25	1.64317	1	13	12	6	6.57267	1	3	2	1.75	1.04881	6.941	102.906	95.9645	46.6276	52.3475	1.3	1.45	0.15	1.39	0.08216
0.79	3.16	2.37	2.562	1.22668	3	6	3	4.2	1.96396	1	17	16	12.6	8.34951	1	3	2	2	0.84515	35.453	196.967	161.514	87.2462	88.9842	1	2.6	1.6	1.39	0.82851
1.55	2.55	1	1.77	0.52372	2	4	2	3.6	1.06904	7	14	7	9.4	4.01782	2	3	1	2.8	0.53452	12.0107	65.409	53.3983	48.4468	24.9407	0.7	1.4	0.7	1.25	0.36839
1.1	2.05	0.95	1.564	0.5052	3	8	5	4.8	2.29129	2	16	14	10	8.17771	1	3	2	1.8	0.93541	24.305	173.04	148.735	93.034	73.9482	1.45	1.75	0.3	1.53	0.14031
0.79	3.98	3.19	2.946	1.76784	2	6	4	3.6	2.61861	1	17	16	12.8	8.29802	1	3	2	2	0.84515	18.9984	200.59	181.592	78.0981	100.865	0.5	2.6	2.1	1.12	1.11612
1.12	1.78	0.66	1.285	0.46669	5	8	3	7.25	2.12132	4	13	9	6.25	6.36396	2	3	1	2.75	0.70711	114.818	140.116	25.298	133.792	17.8884	1.55	1.85	0.3	1.78	0.21213
0.89	3.44	2.55	2.682	1.35848	2	6	4	3.6	2.61861	2	16	14	11.8	7.48331	1	3	2	2	0.84515	15.9994	192.217	176.218	75.5084	100.119	0.6	2.15	1.55	1.06	0.82527
0.82	3.98	3.16	2.952	1.7548	2	6	4	3.4	2.32993	1	17	16	12.8	8.29802	1	3	2	2	0.84515	18.9984	200.59	181.592	68.6106	94.6551	0.5	2.35	1.85	1.07	0.99964
1.55	2.05	0.5	1.855	0.23418	4	5	1	4.25	0.54772	7	15	8	10.5	3.63318	2	3	1	2.75	0.54772	54.938	121.76	66.822	73.5212	35.282	1.35	1.45	0.1	1.39	0.05244
1.78	2.28	0.5	2.155	0.35355	5	6	1	5.75	0.70711	10	13	3	10.75	2.12132	2	3	1	2.75	0.70711	114.818	195.084	80.266	175.017	56.7566	1.35	1.55	0.2	1.4	0.14142
1.54	2.55	1.01	2.045	0.71418	4	4	0	4	0	4	16	12	10	8.48528	2	3	1	2.5	0.70711	47.867	78.96	31.093	63.4135	21.9861	1.15	1.4	0.25	1.28	0.17678
1.54	2.54	1	2.0575	0.61112	3	6	3	4.75	1.64317	4	13	9	9.75	4.32435	2	3	1	2.75	0.54772	26.9815	196.967	169.985	117.195	101.335	1.25	1.4	0.15	1.34	0.06892
0.98	1.96	0.98	1.7	0.52604	2	5	3	4.25	1.64317	1	14	13	9.25	6.22093	1	3	2	2.25	1.04881	6.941	118.71	111.769	85.3469	57.5326	1.45	1.6	0.15	1.53	0.09487
1.6	2.18	0.58	1.89	0.41012	4	5	1	4.5	0.70711	5	15	10	10	7.07107	2	3	1	2.5	0.70711	74.9216	92.9064	17.9848	83.914	12.7172	1.15	1.45	0.3	1.3	0.21213
0.82	3.44	2.62	2.34	1.44122	2	4	2	3	1.26491	1	16	15	9.75	8.21584	1	3	2	2	0.89443	15.9994	51.9961	35.9967	30.7733	19.5576	0.6	2.2	1.6	1.2	0.83905
1.1	2.55	1.45	1.955	0.78773	4	8	4	5.5	2.09762	13	16	3	15.25	1.64317	2	3	1	2.25	0.54772	78.96	204.383	125.423	133.836	70.8142	1.15	1.9	0.75	1.49	0.43215
1.1	2.66	1.56	1.88	1.10309	5	8	3	6.5	2.12132	3	17	14	10	9.89949	2	3	1	2.5	0.70711	126.904	138.905	12.001	132.905	8.48599	1.4	1.95	0.55	1.67	0.38891
1.55	2.54	0.99	2.06	0.60775	3	6	3	4.75	1.64317	7	13	6	10.5	2.75681	2	3	1	2.75	0.54772	26.9815	196.967	169.985	118.963	99.4563	1.25	1.4	0.15	1.34	0.06892
1.12	1.62	0.5	1.245	0.35355	6	8	2	7.5	1.41421	4	13	9	6.25	6.36396	2	3	1	2.75	0.70711	140.116	204.383	64.2673	156.183	45.4438	1.85	1.9	0.05	1.86	0.03536
0.93	3.04	2.11	1.4825	1.13773	2	3	1	2.25	0.54772	1	15	14	4.5	7.66812	1	2	1	1.25	0.54772	6.941	22.9898	16.0488	12.7196	8.34068	0.65	1.8	1.15	1.34	0.53362
1.55	2.05	0.5	1.675	0.35355	4	5	1	4.25	0.70711	7	15	8	9	5.65685	2	3	1	2.75	0.70711	54.938	121.76	66.822	71.6435	47.2503	1.4	1.45	0.05	1.41	0.03536
0.95	3.44	2.49	2.58	1.43654	2	5	3	3	1.69031	2	16	14	11	8.28079	1	3	2	2	0.84515	15.9994	87.62	71.6206	37.3119	38.1694	0.6	2	1.4	1.03	0.75546
1.36	1.36	0	1.36	0	4	4	0	4	0	3	3	0	3	0	3	3	0	3	0	44.9559	44.9559	0	44.9559	0	1.6	1.6	0	1.6	0
1.66	2.55	0.89	2.105	0.62933	4	4	0	4	0	6	16	10	11	7.07107	2	3	1	2.5	0.70711	51.9961	78.96	26.9639	65.4781	19.0664	1.15	1.4	0.25	1.28	0.17678
1.91	2.1	0.19	2.005	0.13435	4	5	1	4.5	0.70711	10	16	6	13	4.24264	2	3	1	2.5	0.70711	58.6934	127.6	68.9066	93.1467	48.7243	1.35	1.4	0.05	1.38	0.03536
1.12	1.96	0.84	1.33	0.59397	5	8	3	7.25	2.12132	4	14	10	6.5	7.07107	2	3	1	2.75	0.70711	118.71	140.116	21.406	134.765	15.1363	1.45	1.85	0.4	1.75	0.28284
1.27	2.28	1.01	2.03	0.52277	2	8	6	5	2.53546	9	17	8	11.4	4.27618	2	3	1	2.8	0.53452	10.811	174.967	164.156	98.8989	69.6778	0.85	1.75	0.9	1.33	0.38173
1.36	1.91	0.55	1.7475	0.28766	4	4	0	4	0	3	13	10	9	4.64758	2	3	1	2.75	0.54772	44.9559	69.723	24.7671	58.0164	11.1092	1.3	1.6	0.3	1.4	0.14832
1.54	1.91	0.37	1.7925	0.19149	4	4	0	4	0	4	13	9	9.25	4.13521	2	3	1	2.75	0.54772	47.867	69.723	21.856	58.7442	9.77451	1.3	1.4	0.1	1.35	0.04472
0.82	2.55	1.73	1.78	0.9887	4	8	4	5.25	2.07364	1	16	15	10.75	7.76531	1	3	2	2	0.89443	78.96	157.25	78.29	100.159	41.8283	1.15	2.35	1.2	1.61	0.63463
1.63	1.91	0.28	1.815	0.14464	4	4	0	4	0	5	13	8	9.5	3.63318	2	3	1	2.75	0.54772	50.9415	69.723	18.7815	59.5128	8.46305	1.3	1.35	0.05	1.34	0.02739
1.81	2.55	0.74	2.18	0.52326	4	4	0	4	0	13	16	3	14.5	2.12132	2	2	0	2	0	69.723	78.96	9.237	74.3415	6.53155	1.15	1.3	0.15	1.23	0.10607
1.25	2.2	0.95	1.8575	0.49383	5	8	3	5.75	1.64317	10	15	5	12	2.68328	2	3	1	2.75	0.54772	106.42	168.934	62.5142	124.148	32.9934	1.4	1.75	0.35	1.53	0.18166
0.98	2.28	1.3	1.5125	0.7056	2	6	4	3.5	2.09762	1	13	12	6.25	6.77495	1	3	2	1.75	1.04881	6.941	195.084	188.143	69.6723	97.1566	1.3	1.45	0.15	1.39	0.08216
1.1	3.44	2.34	2.606	1.38186	2	8	6	3.4	3.11677	3	16	13	12.8	6.72947	2	3	1	2.2	0.53452	15.9994	138.905	122.906	42.777	64.4795	0.6	1.95	1.35	1	0.71838
1.61	1.9	0.29	1.726	0.20506	3	4	1	3.4	0.70711	11	13	2	12.2	1.41421	2	3	1	2.4	0.70711	26.9815	63.546	36.5645	41.6073	25.855	1.25	1.35	0.1	1.29	0.07071
1.91	2.58	0.67	2.178	0.47376	3	4	1	3.6	0.70711	10	16	6	12.4	4.24264	2	3	1	2.6	0.70711	32.065	58.6934	26.6284	48.042	18.8291	1	1.35	0.35	1.21	0.24749
2	2.28	0.28	2.056	0.19799	6	6	0	6	0	10	12	2	11.6	1.41421	3	3	0	3	0	195.084	200.59	5.506	199.489	3.89333	1.35	1.5	0.15	1.47	0.10607
1.14	3.44	2.3	2.614	1.36637	2	8	6	3.4	3.11677	6	16	10	13.4	5.18239	2	3	1	2.2	0.53452	15.9994	144.242	128.243	43.8443	67.3214	0.6	1.85	1.25	0.98	0.67162
1.63	3.44	1.81	2.8475	0.90082	2	4	2	3	0.89443	5	17	12	13.75	6.41093	2	3	1	2.25	0.54772	15.9994	50.9415	34.9421	34.4617	15.6768	0.6	1.35	0.75	0.99	0.33578
2	2.1	0.1	2.05	0.07071	5	6	1	5.5	0.70711	12	16	4	14	2.82843	2	3	1	2.5	0.70711	127.6	200.59	72.99	164.095	51.6117	1.4	1.5	0.1	1.45	0.07071
1.14	1.81	0.67	1.3075	0.47376	4	8	4	7	2.82843	6	13	7	7.75	4.94975	2	3	1	2.75	0.70711	69.723	144.242	74.519	125.612	52.6929	1.3	1.85	0.55	1.71	0.38891
1.22	1.61	0.39	1.415	0.27577	3	5	2	4	1.41421	3	13	10	8	7.07107	2	3	1	2.5	0.70711	26.9815	88.9059	61.9243	57.9437	43.7871	1.25	1.8	0.55	1.53	0.38891
1.54	1.81	0.27	1.7425	0.19092	4	4	0	4	0	4	13	9	10.75	6.36396	2	3	1	2.25	0.70711	47.867	69.723	21.856	64.259	15.4545	1.3	1.4	0.1	1.33	0.07071
0.98	1.96	0.98	1.4625	0.61047	2	5	3	3.5	1.89737	1	14	13	6.75	7.39594	1	3	2	1.75	1.04881	6.941	118.71	111.769	60.1151	67.435	1.45	1.6	0.15	1.49	0.08216
1.36	2.2	0.84	2	0.43556	2	6	4	4.8	2.13809	3	13	10	8.6	4.27618	2	3	1	2.8	0.53452	10.811	192.217	181.406	126.484	108.545	0.85	1.6	0.75	1.3	0.32733
1.33	3.04	1.71	2.185	1.20915	2	5	3	3.5	2.12132	4	15	11	9.5	7.77817	2	3	1	2.5	0.70711	14.0067	91.224	77.2173	52.6154	54.6009	0.65	1.55	0.9	1.1	0.6364
2.1	2.28	0.18	2.19	0.12728	5	6	1	5.5	0.70711	10	16	6	13	4.24264	2	3	1	2.5	0.70711	127.6	195.084	67.484	161.342	47.7184	1.35	1.4	0.05	1.38	0.03536
1.78	2.05	0.27	1.915	0.19092	5	5	0	5	0	13	15	2	14	1.41421	2	2	0	2	0	114.818	121.76	6.942	118.289	4.90874	1.45	1.55	0.1	1.5	0.07071
1.55	2.55	1	2.05	0.70711	4	4	0	4	0	7	16	9	11.5	6.36396	2	3	1	2.5	0.70711	54.938	78.96	24.022	66.949	16.9861	1.15	1.4	0.25	1.28	0.17678
0.79	2.1	1.31	1.5325	0.7347	5	8	3	6	1.54919	1	16	15	9.75	8.21584	1	3	2	2	0.89443	127.6	144.242	16.642	133.087	8.59491	1.4	2.6	1.2	1.81	0.62028
1.81	1.83	0.02	1.825	0.01414	4	4	0	4	0	8	13	5	9.25	3.53553	2	3	1	2.75	0.70711	55.845	69.723	13.878	59.3145	9.81323	1.3	1.4	0.1	1.37	0.07071
0.98	2.28	1.3	1.6325	0.59337	2	6	4	4	1.82574	1	14	13	6.75	6.29153	1	3	2	1.75	0.95743	6.941	195.084	188.143	86.26	87.6082	1.35	1.5	0.15	1.44	0.06292
0.98	2.33	1.35	1.3175	0.95459	2	6	4	3	2.82843	1	14	13	4.25	9.19239	1	2	1	1.25	0.70711	6.941	207.2	200.259	57.0058	141.604	1.45	1.8	0.35	1.54	0.24749
0.93	3.44	2.51	1.878	1.55603	2	3	1	2.8	0.53452	1	17	16	7.2	10.1559	1	2	1	1.4	0.65465	15.9994	35.453	19.4536	24.0843	8.41357	0.6	1.8	1.2	1.4	0.67612
0.93	3.44	2.51	2.36	1.40151	2	4	2	2.75	1.04881	1	16	15	9.5	8.41427	1	3	2	2	0.89443	15.9994	50.9415	34.9421	26.4825	18.2234	0.6	1.8	1.2	1.09	0.64865
1.55	2.55	1	2.05	0.70711	4	4	0	4	0	7	16	9	11.5	6.36396	2	3	1	2.5	0.70711	54.938	78.96	24.022	66.949	16.9861	1.15	1.4	0.25	1.28	0.17678
1.96	2.54	0.58	2.25	0.41012	5	6	1	5.5	0.70711	11	14	3	12.5	2.12132	2	3	1	2.5	0.70711	118.71	196.967	78.2566	157.838	55.3358	1.35	1.45	0.1	1.4	0.07071
1.3	2.54	1.24	1.9975	0.70008	3	6	3	5.25	1.64317	4	13	9	9.75	4.32435	2	3	1	2.75	0.54772	26.9815	196.967	169.985	149.851	90.2371	1.25	1.55	0.3	1.38	0.13784
1.36	1.93	0.57	1.75	0.29516	4	5	1	4.75	0.54772	3	13	10	9.5	4.85798	2	3	1	2.75	0.54772	44.9559	114.818	69.8621	93.8776	35.9071	1.55	1.6	0.05	1.59	0.02739
1.54	1.69	0.15	1.615	0.10607	4	5	1	4.5	0.70711	4	12	8	8	5.65685	3	3	0	3	0	47.867	112.411	64.544	80.139	45.6395	1.4	1.55	0.15	1.48	0.10607
0.98	2.28	1.3	1.72	0.59242	2	6	4	4	1.78885	1	13	12	9.25	6.22093	1	3	2	2	0.89443	6.941	195.084	188.143	85.3678	86.4359	1.3	1.45	0.15	1.35	0.07746
1.1	2.28	1.18	1.996	0.61142	2	8	6	5	2.53546	3	13	10	8.6	4.27618	2	3	1	2.8	0.53452	10.811	138.905	128.094	91.6866	57.1592	0.85	1.95	1.1	1.37	0.46599
1.61	1.91	0.3	1.765	0.18363	3	4	1	3.75	0.54772	5	13	8	9.5	3.63318	2	3	1	2.75	0.54772	26.9815	58.6934	31.7119	48.8275	16.4485	1.25	1.35	0.1	1.33	0.05477
1.65	2.55	0.9	2.1	0.6364	4	4	0	4	0	12	16	4	14	2.82843	2	3	1	2.5	0.70711	65.409	78.96	13.551	72.1845	9.582	1.15	1.35	0.2	1.25	0.14142
1.17	1.78	0.61	1.6275	0.43134	5	8	3	5.75	2.12132	8	13	5	11.75	3.53553	2	3	1	2.25	0.70711	114.818	150.36	35.542	123.704	25.132	1.55	1.85	0.3	1.63	0.21213
1.54	2.55	1.01	1.756	0.53175	2	4	2	3.4	1.06904	4	14	10	7.8	6.23355	2	3	1	2.6	0.65465	12.0107	47.867	35.8563	36.5186	19.621	0.7	1.4	0.7	1.23	0.36253
1.62	3.16	1.54	2.39	1.08894	3	6	3	4.5	2.12132	13	17	4	15	2.82843	2	2	0	2	0	35.453	204.383	168.93	119.918	119.452	1	1.9	0.9	1.45	0.6364
1.1	2.55	1.45	2.0275	0.75368	2	8	6	4	3.09839	10	16	6	13.5	2.75681	2	3	1	2.5	0.63246	12.0107	173.04	161.029	63.9387	83.2434	0.7	1.75	1.05	1.13	0.56657
1.63	2.28	0.65	2.0375	0.3404	4	5	1	4.75	0.54772	5	14	9	9.25	4.03733	2	3	1	2.75	0.54772	50.9415	118.71	67.7685	93.8656	32.3924	1.35	1.45	0.1	1.38	0.05477
0.98	2.01	1.03	1.4825	0.63601	2	5	3	3.25	1.64317	1	14	13	7.5	8.22192	1	2	1	1.5	0.63246	6.941	118.71	111.769	51.308	59.7828	1.25	1.45	0.2	1.4	0.10954
0.82	3.44	2.62	2.33	1.44892	2	6	4	3.75	2.25832	1	16	15	11.5	7.82304	1	2	1	1.75	0.54772	15.9994	204.383	188.384	80.4625	97.3497	0.6	2.35	1.75	1.36	0.98527
1.96	2.28	0.32	2.12	0.22627	5	6	1	5.5	0.70711	10	14	4	12	2.82843	2	3	1	2.5	0.70711	118.71	195.084	76.374	156.897	54.0046	1.35	1.45	0.1	1.4	0.07071
0.93	3.44	2.51	2.55	1.47639	2	6	4	3	2.0702	1	16	15	10.8	8.67673	1	3	2	2	0.84515	15.9994	180.948	164.948	50.3872	87.3095	0.6	1.8	1.2	1.01	0.68713
0.98	3.44	2.46	1.868	1.46742	2	4	2	2.4	1.06904	1	17	16	7.2	10.1559	1	2	1	1.4	0.65465	6.941	79.904	72.963	23.3453	38.0796	0.6	1.45	0.85	1.22	0.4424
1.36	2.28	0.92	1.925	0.48674	4	6	2	5.25	1.04881	3	13	10	9	4.64758	2	3	1	2.75	0.54772	44.9559	195.084	150.128	137.485	79.2735	1.35	1.6	0.25	1.46	0.14405
1.1	1.96	0.86	1.315	0.60811	5	8	3	7.25	2.12132	3	14	11	5.75	7.77817	2	3	1	2.75	0.70711	118.71	138.905	20.1955	133.857	14.2804	1.45	1.95	0.5	1.83	0.35355
0.89	3.44	2.55	2.542	1.49213	2	9	7	4.2	3.81725	2	16	14	11	8.28079	1	3	2	2	0.84515	15.9994	231.036	215.036	83.2722	117.006	0.6	2.15	1.55	1.15	0.91222
1.13	3.44	2.31	2.612	1.37023	2	8	6	3.4	3.11677	5	16	11	13.2	5.69461	2	3	1	2.2	0.53452	15.9994	140.908	124.908	43.1775	65.5456	0.6	1.85	1.25	0.98	0.67162
1.83	3.04	1.21	2.162	0.63118	2	5	3	3.8	1.30931	8	15	7	9.6	3.64496	2	3	1	2.8	0.53452	14.0067	102.906	88.8988	56.8894	37.6055	0.65	1.4	0.75	1.24	0.39506
1	1.61	0.61	1.488	0.43134	3	4	1	3.2	0.70711	2	13	11	10.8	7.77817	1	2	1	1.8	0.70711	26.9815	40.078	13.0965	29.6008	9.2606	1.25	1.8	0.55	1.36	0.38891
0.82	3.98	3.16	2.862	1.85558	2	4	2	2.8	1.30931	1	17	16	11.8	8.88015	1	3	2	2	0.84515	18.9984	54.938	35.9396	30.2063	19.5263	0.5	2.2	1.7	1.02	0.91574
0.82	3.98	3.16	2.934	1.7728	2	4	2	2.8	1.30931	1	17	16	12.4	8.43462	1	3	2	2	0.84515	18.9984	58.6934	39.695	30.9574	21.252	0.5	2.2	1.7	1.01	0.90869
0.82	3.44	2.62	2.528	1.52002	2	6	4	3.2	2.13809	1	16	15	10.8	8.67673	1	3	2	2	0.84515	15.9994	180.948	164.948	53.6089	85.9177	0.6	2.2	1.6	1.09	0.86231
1.23	2.2	0.97	1.82667	0.52214	5	8	3	6	1.73205	10	15	5	12.6667	2.51661	2	3	1	2.67	0.57735	106.42	164.93	58.5103	131.037	30.3382	1.4	1.75	0.35	1.53	0.1893
1.63	1.91	0.28	1.81667	0.19799	4	4	0	4	0	5	10	5	8.33333	3.53553	3	3	0	3	0	50.9415	58.6934	7.7519	56.1094	5.48142	1.35	1.35	0	1.35	3.33E-16
1.24	2.28	1.04	1.84667	0.54123	6	8	2	6.66667	1.1547	10	15	5	13	2.64575	2	3	1	2.67	0.57735	167.259	208.98	41.7214	190.441	21.2447	1.35	1.75	0.4	1.57	0.20207
1.5	3.04	1.54	2.37	0.86616	2	6	4	3.5	2.09762	5	15	10	11.5	5.17687	2	3	1	2.5	0.63246	14.0067	180.948	166.941	68.1268	86.2729	0.65	1.45	0.8	1.03	0.47645
0.95	1.96	1.01	1.455	0.71418	5	5	0	5	0	2	14	12	8	8.48528	1	2	1	1.5	0.70711	87.62	118.71	31.09	103.165	21.9839	1.45	2	0.55	1.73	0.38891
0.98	1.96	0.98	1.3925	0.53988	2	5	3	3.25	1.64317	1	14	13	7	7.64199	1	3	2	1.75	1.04881	6.941	118.71	111.769	49.5002	58.875	1.35	1.45	0.1	1.42	0.05477
0.93	3.04	2.11	2.1525	1.16191	2	5	3	3	1.54919	1	15	14	9	7.79744	1	3	2	2	0.89443	14.0067	92.9064	78.8997	35.9774	41.8331	0.65	1.8	1.15	1.14	0.6362
1.3	2.2	0.9	1.75	0.6364	6	9	3	7.5	2.12132	4	9	5	6.5	3.53553	3	3	0	3	0	192.217	232.038	39.8211	212.128	28.1577	1.35	1.8	0.45	1.58	0.3182
0.98	2.2	1.22	1.685	0.55868	2	5	3	4.25	1.64317	1	13	12	9.25	6.22093	1	3	2	2	0.89443	6.941	114.818	107.877	85.7493	57.7166	1.4	1.55	0.15	1.49	0.08216
1.27	1.96	0.69	1.7625	0.3606	4	8	4	5.25	2.07364	10	17	7	12.75	3.72827	2	3	1	2.75	0.54772	58.6934	174.967	116.274	102.766	61.162	1.35	1.75	0.4	1.48	0.20736
0.82	3.44	2.62	2.21	1.56241	2	8	6	4.25	3.14643	1	16	15	9.75	8.21584	1	3	2	2	0.89443	15.9994	144.242	128.243	65.4272	67.8222	0.6	2.35	1.75	1.35	0.97468
1.81	3.44	1.63	2.6475	1.00325	2	4	2	3	1.26491	11	16	5	14	2.68328	2	3	1	2.25	0.54772	15.9994	69.723	53.7236	41.317	32.1434	0.6	1.35	0.75	0.96	0.45908
1.24	2.55	1.31	2.0625	0.68559	2	8	6	4	3.09839	10	14	4	13	2.19089	2	3	1	2.5	0.63246	12.0107	167.259	155.248	62.4934	80.2179	0.7	1.75	1.05	1.13	0.56657
1.55	1.9	0.35	1.74	0.20416	3	4	1	3.75	0.54772	7	13	6	10.5	2.75681	2	3	1	2.75	0.54772	26.9815	63.546	36.5645	52.2529	18.9834	1.25	1.4	0.15	1.34	0.06892
1.83	3.44	1.61	2.6525	0.99661	2	4	2	3	1.26491	8	16	8	12.75	4.32435	2	3	1	2.5	0.63246	15.9994	63.546	47.5466	37.8474	27.8496	0.6	1.4	0.8	0.99	0.49066
1.33	1.93	0.6	1.48	0.42426	5	5	0	5	0	4	11	7	5.75	4.94975	3	3	0	3	0	91.224	107.868	16.6442	95.3851	11.7692	1.55	1.6	0.05	1.56	0.03536
1.1	3.44	2.34	2.4775	1.2728	2	8	6	4.25	3.14643	11	16	5	14.75	2.73861	2	3	1	2.5	0.63246	15.9994	173.04	157.041	78.2268	83.9349	0.6	1.75	1.15	1.14	0.68319
1.27	2.2	0.93	1.9075	0.48178	5	8	3	5.75	1.64317	10	17	7	12.75	3.72827	2	3	1	2.75	0.54772	106.42	174.967	68.547	126.629	35.8669	1.4	1.75	0.35	1.5	0.18439
0.93	2.55	1.62	1.9225	0.85825	3	4	1	3.75	0.54772	1	16	15	9.75	8.21584	1	3	2	2	0.89443	22.9898	78.96	55.9702	58.2265	29.2589	1.15	1.8	0.65	1.38	0.33615
0.93	3.44	2.51	2.43	1.35064	2	4	2	2.75	1.04881	1	16	15	10.75	7.76531	1	3	2	2	0.89443	15.9994	58.6934	42.694	28.4205	22.401	0.6	1.8	1.2	1.09	0.64865
1.22	2.2	0.98	1.91	0.51241	5	6	1	5.25	0.54772	3	15	12	9.5	5.4037	2	3	1	2.75	0.54772	88.9059	208.98	120.075	127.682	60.0572	1.4	1.8	0.4	1.55	0.20976
0.98	2.28	1.3	1.62	0.58152	2	5	3	3.25	1.3784	1	13	12	9	6.19677	1	3	2	2	0.89443	6.941	102.906	95.9645	40.9524	46.4126	1.25	1.45	0.2	1.33	0.10488
1.54	2.54	1	2.04	0.70711	4	6	2	5	1.41421	4	11	7	7.5	4.94975	3	3	0	3	0	47.867	196.967	149.1	122.417	105.429	1.35	1.4	0.05	1.38	0.03536
0.93	2.58	1.65	1.93	0.87977	3	4	1	3.25	0.54772	1	16	15	9.5	8.41427	1	3	2	2	0.89443	22.9898	50.9415	27.9517	34.5153	12.8789	1	1.8	0.8	1.29	0.41563
0.93	3.44	2.51	2.41	1.36361	2	4	2	2.75	1.04881	1	16	15	10.25	7.91833	1	3	2	2	0.89443	15.9994	55.845	39.8456	27.7084	20.8627	0.6	1.8	1.2	1.1	0.65727
0.93	2.58	1.65	1.8275	0.96064	3	5	2	3.5	1.09545	1	16	15	9	8.89944	1	3	2	2	0.89443	22.9898	88.9059	65.9161	44.0064	33.1231	1	1.8	0.8	1.4	0.50596
1.6	2.2	0.6	1.9025	0.37634	3	5	2	4.5	1.09545	5	13	8	8.5	3.63318	2	3	1	2.75	0.54772	26.9815	101.07	74.0885	80.507	39.3161	1.25	1.45	0.2	1.33	0.09487
1.9	2.28	0.38	1.995	0.2687	4	6	2	4.5	1.41421	10	11	1	10.75	0.70711	3	3	0	3	0	63.546	195.084	131.538	96.4305	93.0114	1.35	1.35	0	1.35	0
1.3	1.96	0.66	1.77	0.34421	4	6	2	4.75	1.04881	4	14	10	9.5	4.51664	2	3	1	2.75	0.54772	58.6934	178.49	119.797	103.647	62.8332	1.35	1.55	0.2	1.43	0.10488
1.31	1.96	0.65	1.7725	0.33875	3	5	2	4	0.89443	2	14	12	9	5.51362	1	3	2	2.25	1.04881	24.305	118.71	94.405	65.1005	42.99	1.35	1.5	0.15	1.41	0.08216
0.93	3.44	2.51	2.4275	1.35219	2	4	2	2.75	1.04881	1	16	15	11	7.74597	1	3	2	2	0.89443	15.9994	63.546	47.5466	29.6336	25.0278	0.6	1.8	1.2	1.09	0.64865
0.98	2.28	1.3	1.8875	0.67725	2	5	3	4	1.54919	1	14	13	8.25	5.89067	1	3	2	2.25	1.04881	6.941	102.906	95.9645	71.348	49.5648	1.25	1.45	0.2	1.35	0.08944
1.12	2.54	1.42	1.995	0.74991	5	8	3	6.25	1.3784	4	13	9	9.75	4.32435	2	3	1	2.75	0.54772	114.818	196.967	82.1486	162.217	45.388	1.35	1.85	0.5	1.53	0.25884
0.82	3.44	2.62	2.2075	1.56515	2	8	6	4	3.09839	1	16	15	9.5	8.41427	1	3	2	2	0.89443	15.9994	140.908	124.908	53.0012	65.2966	0.6	2.2	1.6	1.31	0.91474
1.6	3.04	1.44	2.32	1.01823	2	5	3	3.5	2.12132	5	15	10	10	7.07107	2	3	1	2.5	0.70711	14.0067	92.9064	78.8997	53.4565	55.7905	0.65	1.45	0.8	1.05	0.56569
1.9	1.9	0	1.9	0	3	3	0	3	0	14	14	0	14	0	2	2	0	2	0	28.0855	28.0855	0	28.0855	0	1.1	1.1	0	1.1	0

0.82	3.44	2.62	2.2325	1.53835	2	8	6	4.25	3.14643	1	16	15	11.5	7.82304	1	3	2	2	0.89443	15.9994	164.93	148.931	70.5992	77.6704	0.6	2.35	1.75	1.33
1.65	2.54	0.89	1.935	0.4603	4	6	2	4.5	1.09545	11	12	1	11.5	0.63246	3	3	0	3	0	63.546	196.967	133.421	97.8326	72.4036	1.35	1.35	0	1.35
1.24	2.28	1.04	2.02	0.73539	6	8	2	6.5	1.41421	10	14	4	11	2.82843	3	3	0	3	0	167.259	195.084	27.825	188.128	19.6752	1.35	1.75	0.4	1.45
1.24	2.2	0.96	1.9	0.49767	5	8	3	5.75	1.64317	10	14	4	12	2.52982	2	3	1	2.75	0.54772	106.42	167.259	60.839	124.702	31.7204	1.4	1.75	0.35	1.5
1.3	2.28	0.98	2.035	0.69296	5	6	1	5.25	0.70711	4	9	5	7.75	3.53553	3	3	0	3	0	102.906	178.49	75.5845	121.802	53.4463	1.35	1.55	0.2	1.4
1.17	2.54	1.37	1.855	0.96874	6	8	2	7	1.41421	8	11	3	9.5	2.12132	3	3	0	3	0	150.36	196.967	46.6066	173.663	32.9558	1.35	1.85	0.5	1.6
1.12	1.61	0.49	1.2425	0.34648	3	8	5	6.75	3.53553	4	13	9	6.25	6.36396	2	3	1	2.75	0.70711	26.9815	140.116	113.134	111.832	79.9981	1.25	1.85	0.6	1.7
0.98	1.61	0.63	1.295	0.44548	2	3	1	2.5	0.70711	1	13	12	7	8.48528	1	2	1	1.5	0.70711	6.941	26.9815	20.0405	16.9613	14.1708	1.25	1.45	0.2	1.35
1.23	1.61	0.38	1.515	0.2687	3	8	5	4.25	3.53553	13	13	0	13	0	2	3	1	2.25	0.70711	26.9815	164.93	137.949	61.4687	97.5445	1.25	1.75	0.5	1.38
1.9	2.2	0.3	1.975	0.21213	4	5	1	4.25	0.70711	10	11	1	10.75	0.70711	3	3	0	3	0	63.546	106.42	42.874	74.2645	30.3165	1.35	1.4	0.05	1.36
1	1.96	0.96	1.72	0.67882	4	5	1	4.75	0.70711	2	14	12	11	8.48528	1	2	1	1.75	0.70711	40.078	118.71	78.632	99.052	55.6012	1.45	1.8	0.35	1.54
1.91	2.58	0.67	2.35667	0.47376	3	4	1	3.33333	0.70711	10	16	6	14	4.24264	2	3	1	2.33	0.70711	32.065	58.6934	26.6284	40.9411	18.8291	1	1.35	0.35	1.12
1.61	1.83	0.22	1.7325	0.12534	3	4	1	3.75	0.54772	6	13	7	8.75	3.27109	2	3	1	2.75	0.54772	26.9815	55.845	28.8635	47.6669	15.2367	1.25	1.4	0.15	1.36
0.82	3.44	2.62	2.225	1.54623	2	8	6	4.25	3.14643	1	16	15	10.5	7.82304	1	3	2	2	0.89443	15.9994	151.964	135.965	67.3576	71.4466	0.6	2.35	1.75	1.35
0.93	2.58	1.65	1.93	0.87977	3	4	1	3.25	0.54772	1	16	15	9.5	8.41427	1	3	2	2	0.89443	22.9898	50.9415	27.9517	34.5153	12.8789	1	1.8	0.8	1.29
1.93	2.1	0.17	2.0575	0.12021	5	5	0	5	0	11	16	5	14.75	3.53553	2	3	1	2.25	0.70711	107.868	127.6	19.7318	122.667	13.9525	1.4	1.6	0.2	1.45
1.66	2.19	0.53	1.9625	0.29548	3	4	1	3.5	0.63246	6	15	9	12.25	4.67974	2	3	1	2.25	0.54772	30.9738	69.723	38.7492	45.9167	20.4967	1	1.4	0.4	1.18
1.3	1.62	0.32	1.54	0.22627	6	9	3	6.75	2.12132	4	13	9	10.75	6.36396	2	3	1	2.25	0.70711	204.383	232.038	27.6548	211.297	19.5549	1.8	1.9	0.1	1.87
1.5	2.28	0.78	2.085	0.55154	5	9	4	6	2.82843	5	9	4	8	2.82843	3	3	0	3	0	102.906	231.036	128.13	134.938	90.6019	1.35	1.8	0.45	1.46
1.38	3.44	2.06	2.925	1.45664	2	9	7	3.75	4.94975	6	16	10	13.5	7.07107	2	3	1	2.25	0.70711	15.9994	238.029	222.03	71.5068	156.999	0.6	1.75	1.15	0.89
1.23	1.81	0.58	1.665	0.41012	4	8	4	5	2.82843	13	13	0	13	0	2	3	1	2.25	0.70711	69.723	164.93	95.2073	93.5248	67.3217	1.3	1.75	0.45	1.41
1.13	2.33	1.2	2.03	0.84853	6	8	2	6.5	1.41421	5	14	9	11.75	6.36396	2	3	1	2.25	0.70711	140.908	207.2	66.2924	190.627	46.8758	1.8	1.85	0.05	1.81
1.25	1.81	0.56	1.67	0.39598	4	8	4	5	2.82843	13	15	2	13.5	1.41421	2	3	1	2.25	0.70711	69.723	168.934	99.2112	94.5258	70.1529	1.3	1.75	0.45	1.41
1.25	1.62	0.37	1.5275	0.26163	6	8	2	6.5	1.41421	13	15	2	13.5	1.41421	2	3	1	2.25	0.70711	168.934	204.383	35.4491	195.521	25.0663	1.75	1.9	0.15	1.86
1	2.2	1.2	1.7275	0.51286	1	4	3	3	1.41421	1	14	13	7.5	6.95222	1	2	1	1.5	0.57735	1.00794	69.723	68.7151	34.7236	28.4865	0.25	1.8	1.55	1.11
2.28	2.33	0.05	2.2925	0.03536	6	6	0	6	0	10	14	4	11	2.82843	2	3	1	2.75	0.70711	195.084	207.2	12.116	198.113	8.56731	1.35	1.8	0.45	1.46
1.38	1.62	0.24	1.56	0.16971	6	9	3	6.75	2.12132	6	13	7	11.25	4.94975	2	3	1	2.25	0.70711	204.383	238.029	33.6456	212.795	23.791	1.75	1.9	0.15	1.86
1.63	2.28	0.65	1.955	0.45962	4	5	1	4.5	0.70711	5	9	4	7	2.82843	3	3	0	3	0	50.9415	102.906	51.964	76.9235	36.7441	1.35	1.35	0	1.35
0.82	3.44	2.62	1.772	1.57122	2	4	2	3.6	1.06904	1	17	16	7.2	10.1559	1	2	1	1.4	0.65465	15.9994	79.904	63.9046	42.6397	27.6196	0.6	2.2	1.6	1.67
0.82	2.55	1.73	1.7625	1.00572	4	8	4	5.25	2.07364	1	16	15	9.5	8.41427	1	3	2	2	0.89443	78.96	140.908	61.9476	96.0739	32.914	1.15	2.35	1.2	1.63
1.36	1.91	0.55	1.74	0.28552	4	5	1	4.25	0.54772	3	13	10	9	4.64758	2	3	1	2.75	0.54772	44.9559	114.818	69.8621	69.2902	33.9972	1.35	1.6	0.25	1.46
0.98	2.2	1.22	1.7	0.56306	2	5	3	3.75	1.3784	1	13	12	8.75	6.22093	1	3	2	2	0.89443	6.941	101.07	94.129	61.8643	43.2536	1.3	1.45	0.15	1.34
2.19	2.28	0.09	2.244	0.06364	3	5	2	4.2	1.41421	9	15	6	11.4	4.24264	2	3	1	2.6	0.70711	30.9738	102.906	71.9317	74.1328	50.8634	1	1.35	0.35	1.21
1.17	2.55	1.38	2.045	0.71942	2	8	6	4	3.09839	8	14	6	11.5	3.28634	2	3	1	2.5	0.63246	12.0107	150.36	138.349	58.2687	71.4439	0.7	1.85	1.15	1.15
1.91	2.28	0.37	2.1075	0.21934	4	5	1	4.75	0.54772	9	14	5	10.5	2.60768	2	3	1	2.75	0.54772	58.6934	118.71	60.0166	95.8036	28.3037	1.35	1.45	0.1	1.38
1	3.44	2.44	2.572	1.43881	2	4	2	2.8	1.30931	2	16	14	10.8	8.55236	1	3	2	2	0.84515	15.9994	47.867	31.8676	27.1886	18.6061	0.6	1.8	1.2	1
0.95	3.44	2.49	2.574	1.4437	2	5	3	3.2	1.96396	2	16	14	11	8.28079	1	3	2	2	0.84515	15.9994	92.9064	76.907	45.7049	48.6684	0.6	2	1.4	1.05
1.36	2.2	0.84	1.885	0.4405	4	5	1	4.75	0.54772	3	13	10	9	4.64758	2	3	1	2.75	0.54772	44.9559	114.818	69.8621	93.1535	35.4647	1.4	1.6	0.2	1.49
0.98	2.58	1.6	2.025	0.8276	2	5	3	3.25	1.3784	1	16	15	11.75	7.91833	1	2	1	1.75	0.54772	6.941	118.71	111.769	47.4453	53.6372	1	1.45	0.45	1.23
1.69	3.04	1.35	2.58	0.69748	2	5	3	2.75	1.64317	12	15	3	14	1.54919	2	3	1	2.25	0.54772	12.0107	112.411	100.4	38.1088	54.2725	0.65	1.55	0.9	0.89
1.61	2.1	0.49	1.9275	0.25383	3	5	2	4.25	1.04881	11	16	5	14	2.68328	2	3	1	2.25	0.54772	26.9815	127.6	100.618	86.4319	54.5811	1.25	1.4	0.15	1.35
1.36	1.36	0	1.36	0	4	4	0	4	0	3	3	0	3	0	3	3	0	3	0	44.9559	44.9559	0	44.9559	0	1.6	1.6	0	1.6
2.1	2.1	0	2.1	0	5	5	0	5	0	16	16	0	16	0	2	2	0	2	0	127.6	127.6	0	127.6	0	1.4	1.4	0	1.4
1.55	1.9	0.35	1.7825	0.18075	4	5	1	4.25	0.54772	7	13	6	10.5	2.75681	2	3	1	2.75	0.54772	54.938	114.818	59.88	74.212	29.9857	1.35	1.55	0.2	1.41
1.63	1.91	0.28	1.84	0.19799	4	4	0	4	0	5	10	5	8.75	3.53553	3	3	0	3	0	50.9415	58.6934	7.7519	56.7554	5.48142	1.35	1.35	0	1.35
1.23	2.2	0.97	1.974	0.50396	2	8	6	5	2.53546	10	13	3	11.2	1.96396	2	3	1	2.8	0.53452	10.811	164.93	154.119	99.0003	66.2497	0.85	1.75	0.9	1.36
0.82	3.98	3.16	2.952	1.7548	2	6	4	3.2	2.13809	1	17	16	12.8	8.29802	1	3	2	2	0.84515	18.9984	200.59	181.592	59.3367	94.9504	0.5	2.2	1.7	1.04
0.95	3.44	2.49	2.586	1.42953	2	5	3	3	1.69031	2	16	14	11.2	8.03563	1	3	2	2	0.84515	15.9994	87.62	71.6206	37.5229	38.3079	0.6	2	1.4	1.04
1.14	1.78	0.64	1.3	0.45255	5	8	3	7.25	2.12132	6	13	7	7.75	4.94975	2	3	1	2.75	0.70711	114.818	144.242	29.424	136.886	20.8059	1.55	1.85	0.3	1.78
0.98	2.01	1.03	1.7125	0.53654	2	5	3	4	1.54919	1	14	13	9.25	6.22093	1	3	2	2.25	1.04881	6.941	107.868	100.927	73.8293	52.1258	1.25	1.6	0.35	1.48
1.5	1.81	0.31	1.7325	0.2192	4	6	2	4.5	1.41421	5	13	8	11	5.65685	2	3	1	2.25	0.70711	69.723	180.948	111.225	97.5292	78.6479	1.3	1.45	0.15	1.34
1.91	2.55	0.64	2.23	0.45255	4	4	0	4	0	10	16	6	13	4.24264	2	3	1	2.5	0.70711	58.6934	78.96	20.2666	68.8267	14.3307	1.15	1.35	0.2	1.25
0.89	1.9	1.01	1.582	0.46188	3	6	3	3.6	1.5	2	14	12	11.2	5.77711	1	2	1	1.8	0.5	26.9815	137.327	110.345	49.4922	54.9002	1.1	2.15	1.05	1.37
1.36	1.96	0.6	1.785	0.31145	4	5	1	4.25	0.54772	3	14	11	9.25	5.00999	2	3	1	2.75	0.54772	44.9559	118.71	73.7541	70.2632	36.0847	1.35	1.6	0.25	1.44
0.95	1.78	0.83	1.52	0.4519	3	5	2	4.6	1.06904	2	13	11	8.6	7.20119	1	2	1	1.6	0.65465	24.305	114.818	90.513	91.2758	46.9086	1.5	2	0.5	1.63
1	3.04	2.04	1.532	1.05744	2	6	4	4	1.69031	2	15	13	6.8	7.90118	1	2	1	1.4	0.65465	14.0067	204.383	190.377	67.7248	92.3004	0.65	1.9	1.25	1.59
1.3	3.44	2.14	2.79	1.14941	2	6	4	3.6	2.61861	4	16	12	13.2	6.23355	2	3	1	2.2	0.53452	15.9994	207.2	191.201	86.7376	116.407	0.6	1.8	1.2	1.03
0.98	2.19	1.21	1.832	0.55663	2	4	2	3.2	0.93541	1	15	14	10.6	6.40312	1	3	2	2.2	0.93541	6.941	63.546	56.605	39.1961	27.1652	1	1.45	0.45	1.23
1	3.04	2.04	1.716	1.19073	2	6	4	4	1.69031	2	15	13	6.4	7.39691	1	3	2	1.6	1.06904	14.0067	196.967	182.96	66.2415	88.3805	0.65	1.8	1.15	1.48
1.17	1.81	0.64	1.33	0.45255	4	8	4	7	2.82843	8	13	5	9.25	3.53553	2	3	1	2.75	0.70711	69.723	150.36	80.637	130.201	57.019	1.3	1.85	0.55	1.71
0.98	2.28	1.3	1.72	0.59242	2	5	3	3.75	1.3784	1	13	12	9	6.19677	1	3	2	2	0.89443	6.941	102.906	95.9645	62.3231	43.9255	1.3	1.45	0.15	1.35
0.89	3.44	2.55	2.55	1.48228	2	6	4	3.2	2.13809	2	16	14	10.8	8.55236	1	3	2	2	0.84515	15.9994	137.327	121.328	46.6384	62.7984	0.6	2.15	1.55	1.07
1	1.9	0.9	1.62	0.41193	3	4	1	3.6	0.61237	2	14	12	10.8	5.61249	1	3	2	2.2	0.93541	28.0855	65.409	37.3235	45.4134	21.1293	1.1	1.8	0.7	1.34
1.1	3.44	2.34	2.606	1.38186	2	8	6	3.4	3.11677	3	16	13	12.8	6.72947	2	3	1	2.2	0.53452	15.9994	138.905	122.906	42.777	64.4795	0.6	1.95	1.35	1
0.82	3.44	2.62	2.504	1.55006	2	9	7	4	3.68394	1	16	15	11	8.45154	1	3	2	2	0.84515	15.9994	238.029	222.03	74.299	115.153	0.6	2.35	1.75	1.18
1.54	2.55	1.01	1.758	0.53079	2	6	4	4	1.69031	4	14	10	7.8	6.23355	2	3	1	2.6	0.65465	12.0107	204.383	192.373	71.999	90.3787	0.7	1.9	1.2	1.36
1.54	3.04	1.5	1.856	0.79218	2	6	4	4	1.69031	4	15	11	8	6.60087	2	3	1	2.6	0.65465	14.0067	204.383	190.377	72.3982	89.9106	0.65	1.9	1.25	1.35
1.1	3.44	2.34	2.616	1.36919	2	8	6	3.6	3.11677	3	16	13	11.4	7.63451	2	3	1	2.4	0.65465	15.9994	138.905	122.906	47.78	63.6723	0.6	1.95	1.35	1.03
1.1	2.28	1.18	1.996	0.61142	2	8	6	5	2.53546	9	13	4	10.2	2.13809	2	3	1	2.8	0.53452	10.811	158.925	148.114	95.6906	63.6939	0.85	1.75	0.9	1.33
1.1	3.98	2.88	3.26	2.03647	2	8	6	3.5	4.24264	3	17	14	13.5	9.89949	2	3	1	2.25	0.70711	18.9984	138.905	119.907	48.9752	84.7871	0.5	1.95	1.45	0.86
1.22	2.55	1.33	2.0575	0.69521	2	5	3	3.25	1.64317	3	14	11	10.25	5.68331	2	3	1	2.5	0.63246	12.0107	88.9059	76.8952	42.9052	41.3486	0.7	1.8	1.1	1.14
1.54	1.96	0.42	1.83	0.21335	4	5	1	4.25	0.54772	4	14	10	9.5	4.51664	2	3	1	2.75	0.54772	47.867	118.71	70.843	70.991	35.2947	1.35	1.45	0.1	1.39
1.1	1.96	0.86	1.72	0.45352	4	8	4	5.25	2.07364	10	16	6	12.5	3.28634	2	3	1	2.75	0.54772	58.6934	173.04	114.347	102.284	60.2545	1.35	1.75	0.4	1.48
1.78	3.16	1.38	2.47	0.97581	3	5	2	4	1.41421	13	17	4	15	2.82843	2	2	0	2	0	35.453	114.818	79.365	75.1355	56.1195	1	1.55	0.55	1.28
2.16	2.28	0.12	2.22	0.08485	5	5	0	5	0	6	9	3	7.5	2.12132	3	3	0	3	0	95.94	102.906	6.9655	99.4228	4.92535	1.35	1.45	0.1	1.4
1.93	2.55	0.62	2.2625	0.36588	4	6	2	4.75	1.04881	11	16	5	14.5	2.60768	2	3	1	2.25	0.54772	78.96	208.98	130.02	118.692	67.606	1.15	1.6	0.45	1.38
1.22	2.55	1.33	1.566	0.68929	2	6	4	4.6	1.81265	3	14	11	7.2	6.88684	2	3	1	2.6	0.65465	12.0107	204.383	192.373	96.6223	82.2674	0.7	1.9	1.2	1.6
1.36	2.28	0.92	2.048	0.47618	2	5	3	4.2	1.55839	3	13	10	8.6	4.27618	2	3	1	2.8	0.53452	10.811	102.906	92.0945	72.8967	51.1891	0.85	1.6	0.75	1.3
1.1	2.28	1.18	1.996	0.61142	2	8	6	5	2.53546	9	16	7	11.2	3.81725	2	3	1	2.8	0.53452	10.811	173.04	162.229	98.5135	68.9301	0.85	1.75	0.9	1.33
1.14	3.44	2.3	2.618	1.36115	2	8	6	3.6	3.11677	5	16	11	11.8	6.88684	2	3	1	2.4	0.65465	15.9994	144.242	128.243	48.6363	66.3897	0.6	1.85	1.25	1
1.65	3.98	2.33	3.104	1.43857	2	5	3	3	1.69031	11	17	6	14.8	3.62531	2	3	1	2.4	0.65465	18.9984	107.868	88.8698	46.0545	47.7779	0.5	1.6	1.1	0.89
0.89	1.96	1.07	1.425	0.7566	5	6	1	5.5	0.70711	2	14	12	8	8.48528	1	2	1	1.5	0.70711	118.71	137.327	18.617	128.019	13.1642	1.45	2.15	0.7	1.8
1.62	2.55	0.93	2.185	0.49514	4	6	2	5	1.26491	13	16	3	15	1.54919	2	2	0	2	0	78.96	208.98	130.02	142.821	80.8045	1.15	1.9	0.75	1.45
0.95	3.44	2.49	2.4575	1.32853	2	6	4	3.75	2.25832	2	16	14	11.5	7.23878	1	3	2	2	0.89443	15.9994	200.59	184.591	80.0522	95.4823	0.6	2	1.4	1.18
1.2	1.9	0.7	1.664	0.31953	3	8	5	4.4	2.3184	6	14	8	10	4.47214	2	3	1	2.6	0.61237	28.0855	157.25	129.165	63.4826	60.1096	1.1	1.8	0.7	1.36
0.82	3.44	2.62	2.548	1.49662	2	5	3	3	1.69031	1	16	15	10.8	8.67673	1	3	2	2	0.84515	15.9994	92.9064	76.907	36.0006	39.8569	0.6	2.2	1.6	1.09
1.14	2.28	1.14	2.004	0.5905	2	8	6	5	2.53546	6	13	7	9.2	2.9761	2	3	1	2.8	0.53452	10.811	144.242	133.431	92.7539	58.7818	0.85	1.85	1	1.35
1.65	2.55	0.9	1.938	0.41939	2	4	2	3.6	1.06904	8	14	6	10	3.38062	2	3	1	2.8	0.53452	12.0107	65.409	53.3983	48.9909	25.1994	0.7	1.4	0.7	1.25
2	2.28	0.28	2.056	0.19799	6	6	0	6	0	10	12	2	11.6	1.41421	3	3	0	3	0	195.084	200.59	5.506	199.489	3.89333	1.35	1.5	0.15	1.47
1.54	2	0.46	1.86	0.23933	4	6	2	5	1.26491	4	12	8	9.75	4.23084	3	3	0	3	0	47.867	200.59	152.723	128.148	91.9003	1.35	1.5	0.15	1.44
1.38	1.91	0.53	1.745	0.27488	4	9	5	5.5	2.60768	6	13	7	9.75	3.14643	2	3	1	2.75	0.54772	58.6934	238.029	179.336	117.558	92.6301	1.35	1.75	0.4	1.5
1.63	2.28	0.65	2.1175	0.45962	4	6	2	5.5	1.41421	5	10	5	8.75	3.53553	3	3	0	3	0	50.9415	195.084	144.143	159.048	101.924	1.35	1.35	0	1.35
0.89	3.44	2.55	2.529	1.48558	2	8	6	3.7	2.87977	2	17	15	12.2	7.98274	1	3	2	2	0.83045	15.9994	174.967	158.968	63.8524	80.6515	0.6	2.15	1.55	1.11
0.89	3.44	2.55	2.615	1.38729	2	6	4	3.4	2.36352	2	16	14	11.5	7.59424	1	3	2	2	0.83045	15.9994	186.207	170.208	61.2702	82.5296	0.6	2.15	1.55	1.07
1.17	2.55	1.38	2.062	0.54982	2	8	6	4.6	2.4267	8	15	7	10.6	3.77124	2	3	1	2.6	0.57735	12.0107	150.36	138.349	79.0969	59.7691	0.7	1.85	1.15	1.23
1.1	3.44	2.34	2.606	1.38186	2	8	6	3.4	3.11677	3	16	13	12.8	6.72947	2	3	1	2.2	0.53452	15.9994	138.905	122.906	42.777	64.4795	0.6	1.95	1.35	1
2.2	2.55	0.35	2.48	0.24749	2	6	4	2.8	2.82843	9	14	5	13	3.53553	2	3	1	2.2	0.70711	12.0107	192.217	180.206	48.052	127.425	0.7	1.35	0.65	0.83
1.1	3.44	2.34	2.686	1.24229	2	8	6	3.4	2.63923	7	16	9	13.6	4.7416	2	3	1	2.3	0.60172	15.9994	208.98	192.981	57.3778	86.6823	0.6	1.75	1.15	0.98
0.79	2.96	2.17	2.272	1.1885	4	6	2	4.6	1.06904	1	17	16	12.8	8.29802	1	3	2	2	0.84515	79.904	132.905	53.0015	97.0057	29.2985	1.15	2.6	1.45	1.52
1.14	2.55	1.41	2.012	0.88222	4	8	4	5.6	2.61861	6	17	11	14.2	5.50325	2	3	1	2.4	0.65465	78.96	174.967	96.007	111.218	54.3673	1.15	1.85	0.7	1.41
0.95	2.28	1.33	1.648	0.74806	5	6	1	5.4	0.61237	2	13	11	7.4	5.67891	1	3	2	2	1.11803	87.62	195.084	107.464	136.045	61.5217	1.35	2	0.65	1.65
0.89	3.44	2.55	2.547	1.49863	2	6	4	3.4	2.28941	2	16	14	11.8	8.4241	1	2	1	1.7	0.60172	15.9994	137.327	121.328	58.587	70.5739	0.6	2.15	1.55	1.13
1	3.44	2.44	2.572	1.43881	2	4	2	2.8	1.30931	2	16	14	10.8	8.55236	1	3	2	2	0.84515	15.9994	47.867	31.8676	27.1886	18.6061	0.6	1.8	1.2	1
0.95	2.54	1.59	1.93	0.73599	4	6	2	5	1.11803	2	13	11	10	5.12348	1	3	2	2.2	0.93541	69.723	196.967	127.244	124.2	74.7151	1.3	2	0.7	1.46
0.89	2.54	1.65	2.014	0.75401	5	6	1	5.6	0.61237	2	15	13	10.8	5.93717	1	3	2	2.2	0.93541	121.76	196.967	75.2066	154.956	43.4616	1.35	2.15	0.8	1.55
1.93	3.04	1.11	2.633	0.48242	2	5	3	2.3	1.10782	11	15	4	14	1.45644	2	3	1	2.1	0.36927	10.811	107.868	97.0572	22.2749	35.1458	0.65	1.6	0.95	0.78
0.89	3.44	2.55	2.514	1.50415	2	6	4	3.5	2.43891	2	16	14	10.8	8.4241	1	3	2	2	0.83045	15.9994	180.948	164.948	64.0504	81.8695	0.6	2.15	1.55	1.12
0.89	3.44	2.55	2.576	1.46026	2	8	6	3.8	2.98271	2	16	14	11.5	7.63951	1	3	2	2	0.83045	15.9994	192.217	176.218	70.7109	90.025	0.6	2.15	1.55	1.11
0.89	3.44	2.55	2.641	1.36783	2	6	4	3.3	2.2011	2	16	14	11.4	7.7058	1	3	2	2	0.83045	15.9994	137.327	121.328	52.2435	64.7043	0.6	2.15	1.55	1.08
1.9	3.04	1.14	2.63	0.48912	2	4	2	2.2	0.73855	11	15	4	14	1.45644	2	3	1	2.1	0.36927	10.811	63.546	52.735	17.8427	18.8018	0.65	1.35	0.7	0.76
2.02	3.44	1.42	2.878	0.9367	2	6	4	2.4	1.63299	13	16	3	15	1.82574	2	2	0	2	0	10.811	208.98	198.169	33.741	79.5529	0.6	1.6	1	0.78
0.79	2.96	2.17	2.3	1.16616	4	6	2	4.4	1.06904	1	17	16	12	8.7014	1	3	2	2	0.84515	55.845	132.905	77.0605	85.6925	33.9141	1.15	2.6	1.45	1.49
0.82	2.19	1.37	1.586	0.79217	3	4	1	3.6	0.61237	1	15	14	8.4	7.88987	1	3	2	1.8	0.93541	30.9738	58.6934	27.7196	39.7675	12.6706	1	2.2	1.2	1.55
1.66	3.44	1.78	2.8	1.05844	2	6	4	3.2	2.13809	6	16	10	13.8	5.23723	2	3	1	2.2	0.53452	15.9994	208.98	192.981	61.7949	100.092	0.6	1.6	1	0.96
1.22	1.9	0.68	1.51	0.40016	3	5	2	4.2	0.93541	2	11	9	6	5.12348	1	3	2	2.6	1	24.305	88.9059	64.6009	65.8417	29.5791	1.35	1.8	0.45	1.56
1	3.04	2.04	2.044	1.0493	2	4	2	2.4	1	2	15	13	7.2	7.96084	1	2	1	1.4	0.61237	9.01218	40.078	31.0658	17.2232	14.5546	0.65	1.8	1.15	1.04
1.14	1.91	0.77	1.558	0.43828	4	8	4	5.8	2.29129	6	12	6	8.8	3	3	3	0	3	0	58.6934	144.242	85.5486	103.656	48.1351	1.35	1.85	0.5	1.59
1.12	2.01	0.89	1.762	0.46563	4	8	4	4.8	2.13809	4	14	10	10.4	5.95219	2	3	1	2.4	0.65465	51.9961	140.116	88.1199	82.0064	40.2693	1.25	1.85	0.6	1.4
1.93	3.16	1.23	2.57	0.62548	3	6	3	4	1.49071	11	17	6	15.2	2.62467	2	3	1	2.2	0.4714	32.065	208.98	176.915	83.9639	80.9675	1	1.6	0.6	1.24
1.14	1.9	0.76	1.554	0.43327	4	8	4	5.8	2.29129	6	12	6	9.2	3.29773	3	3	0	3	0	63.546	144.242	80.696	105.597	45.311	1.35	1.85	0.5	1.59
1.2	1.9	0.7	1.578	0.39752	4	8	4	5.8	2.29129	10	12	2	10.8	0.93541	3	3	0	3	0	63.546	157.25	93.704	110.801	52.3918	1.35	1.8	0.45	1.57
0.98	3.04	2.06	2.538	0.74711	2	2	0	2	0	1	15	14	13	4.98483	1	2	1	1.9	0.36927	6.941	14.0067	7.0657	12.1822	2.54604	0.65	1.45	0.8	0.77
0.93	3.44	2.51	2.128	1.41035	2	4	2	2.8	0.93541	1	16	15	9	8.47791	1	3	2	1.8	0.93541	15.9994	63.546	47.5466	28.3049	22.3697	0.6	1.8	1.2	1.23
0.82	2.58	1.76	2.15	0.91128	3	5	2	3.4	1.06904	1	16	15	12.8	7.90118	1	2	1	1.8	0.53452	30.9738	85.4678	54.494	42.5273	28.6964	1	2.35	1.35	1.27
1.1	3.44	2.34	2.692	1.28703	2	8	6	3.2	3.20713	13	16	3	15.4	1.60357	2	3	1	2.2	0.53452	10.811	173.04	162.229	46.3698	84.6776	0.6	1.75	1.15	0.88
1.24	2.55	1.31	2.084	0.59961	2	8	6	3.2	3	13	14	1	13.6	0.61237	2	3	1	2.2	0.5	10.811	167.259	156.448	42.5805	77.927	0.7	1.75	1.05	0.97
1.12	2.2	1.08	1.666	0.60392	5	8	3	6.2	1.83712	4	12	8	8	4.1833	3	3	0	3	0	106.42	140.116	33.696	121.097	19.6033	1.4	1.85	0.45	1.61
1.1	1.61	0.51	1.508	0.36062	3	8	5	4	3.53553	11	13	2	12.6	1.41421	2	3	1	2.2	0.70711	26.9815	158.925	131.944	53.3703	93.2984	1.25	1.75	0.5	1.35
1.14	2.2	1.06	1.924	0.4981	2	8	6	4.8	3	6	13	7	10	3.3541	2	3	1	2.6	0.61237	10.811	192.217	181.406	110.06	103.635	0.85	1.85	1	1.25
0.89	2.05	1.16	1.506	0.65468	4	6	2	5.2	0.93541	2	15	13	9.2	7.47496	1	3	2	1.8	0.93541	65.409	137.327	71.918	116.717	33.2271	1.35	2.15	0.8	1.71
0.89	3.44	2.55	2.512	1.50713	2	8	6	3.8	2.98271	2	16	14	11.4	7.77263	1	3	2	2	0.83045	15.9994	180.948	164.948	70.3562	88.6571	0.6	2.15	1.55	1.12
1.55	2.66	1.11	2.272	0.4932	4	5	1	4.4	0.57735	7	17	10	14.2	4.30762	2	3	1	2.2	0.4714	54.938	126.904	71.9664	92.3045	32.5627	1.15	1.45	0.3	1.31
2.04	2.16	0.12	2.08	0.08485	2	5	3	3	2.12132	6	13	7	10.6667	4.94975	2	3	1	2.33	0.70711	10.811	95.94	85.129	39.1873	60.1953	0.85	1.45	0.6	1.05
1.3	2.54	1.24	1.91333	0.62011	3	9	6	6	3	4	14	10	9.66667	5.1316	2	3	1	2.67	0.57735	28.0855	232.038	203.953	152.363	109.047	1.1	1.8	0.7	1.42
1.1	2.04	0.94	1.72667	0.66468	2	8	6	4	4.24264	11	13	2	12.3333	1.41421	2	3	1	2.33	0.70711	10.811	158.925	148.114	60.1824	104.733	0.85	1.75	0.9	1.15
1.31	2	0.69	1.77	0.4879	3	6	3	5	2.12132	2	12	10	8.66667	7.07107	1	3	2	2.33	1.41421	24.305	200.59	176.285	141.828	124.652	1.5	1.5	0	1.5
1.22	2.04	0.82	1.76667	0.57983	2	5	3	3	2.12132	3	13	10	9.66667	7.07107	2	3	1	2.33	0.70711	10.811	88.9059	78.0949	36.8426	55.2214	0.85	1.8	0.95	1.17

1	1.62	0.62	1.465	0.43841	4	6	2	5.5	1.41421	2	13	11	10.25	7.77817	1	2	[illegible]
1.61	2.28	0.67	2.0175	0.35647	3	6	3	4.75	1.64317	10	13	3	11	1.54919	2	3	[illegible]
1.55	2.1	0.55	1.825	0.38891	4	5	1	4.5	0.70711	7	16	9	11.5	6.36396	2	3	[illegible]
1.66	2.2	0.54	2.065	0.38184	4	6	2	5.5	1.41421	6	9	3	8.25	2.12132	3	3	[illegible]
0.98	2.2	1.22	1.8475	0.64109	2	5	3	4	1.54919	1	14	13	8.75	6.02495	1	3	[illegible]
1.54	2	0.46	1.655	0.32527	4	6	2	4.5	1.41421	4	12	8	6	5.65685	3	3	[illegible]
1.96	2.2	0.24	2.14	0.16971	5	5	0	5	0	10	14	4	11	2.82843	2	3	[illegible]
1.54	1.61	0.07	1.5925	0.0495	3	4	1	3.25	0.70711	4	13	9	10.75	6.36396	2	3	[illegible]
1.38	2.02	0.64	1.7	0.45255	6	9	3	7.5	2.12132	6	15	9	10.5	6.36396	2	3	[illegible]
1.22	2.55	1.33	1.66333	0.94045	2	5	3	4	2.12132	3	14	11	6.66667	7.77817	2	3	[illegible]
0.82	3.44	2.62	2.2	1.57348	2	8	6	4.25	3.14643	1	16	15	9	8.89944	1	3	[illegible]
1.93	2.96	1.03	2.445	0.72832	4	5	1	4.5	0.70711	11	17	6	14	4.24264	2	3	[illegible]
1.22	1.61	0.39	1.5125	0.27577	3	8	5	4.25	3.53553	12	13	1	12.75	0.70711	2	3	[illegible]
0.98	1.69	0.71	1.335	0.50205	2	5	3	3.5	2.12132	1	12	11	6.5	7.77817	1	3	[illegible]
1.31	2.28	0.97	2.0375	0.68589	3	6	3	5.25	2.12132	2	10	8	8	5.65685	1	3	[illegible]
1.17	1.31	0.14	1.275	0.09899	3	8	5	4.25	3.53553	2	8	6	3.5	4.24264	1	3	[illegible]
[illegible]	[illegible]	[illegible]	[illegible]	[illegible]	[illegible]	[illegible]	[illegible]	[illegible]	[illegible]	[illegible]	[illegible]	[illegible]	[illegible]	[illegible]	[illegible]	[illegible]	[illegible]

1.36	2.04	0.68	1.81333	0.48083	2	9	7	4.33333	4.94975	7	13	6	11	4.24264	2	3	1	2.33	0.70711	10.811	237	226.189	86.2073	159.94	0.85	1.75	0.9	1.15
1.3	2.2	0.9	1.6	0.6364	5	6	1	5.66667	0.70711	4	10	6	6	4.24264	3	3	0	3	0	106.42	178.49	72.07	154.467	50.9612	1.4	1.55	0.15	1.5
0.89	1.9	1.01	1.56333	0.71418	3	6	3	4	2.12132	2	14	12	10	8.48528	1	2	1	1.67	0.70711	28.0855	137.327	109.242	64.4993	77.2454	1.1	2.15	1.05	1.45
1.9	3.16	1.26	2.74	0.89095	3	4	1	3.33333	0.70711	11	17	6	15	4.24264	2	3	1	2.33	0.70711	35.453	63.546	28.093	44.8173	19.8648	1	1.35	0.35	1.12
1.63	2.96	1.33	2.51667	0.94045	4	4	0	4	0	5	17	12	13	8.48528	2	3	1	2.33	0.70711	50.9415	79.904	28.9625	70.2498	20.4796	1.15	1.35	0.2	1.22
1.25	2.55	1.3	2.11667	0.91924	2	8	6	4	4.24264	14	15	1	14.3333	0.70711	2	3	1	2.33	0.70711	12.0107	168.934	156.924	64.3185	110.962	0.7	1.75	1.05	1.05
1.54	2.2	0.66	1.98	0.46669	1	4	3	2	2.12132	1	4	3	2	2.12132	1	3	2	1.67	1.41421	1.00794	47.867	46.8591	16.6276	33.1344	0.25	1.4	1.15	0.63
1.54	2.1	0.56	1.91333	0.39598	4	5	1	4.66667	0.70711	4	16	12	12	8.48528	2	3	1	2.33	0.70711	47.867	127.6	79.733	101.022	56.3797	1.4	1.4	0	1.4
0.98	2.02	1.04	1.43667	0.53144	2	6	4	3.66667	2.08167	1	15	14	6	7.81025	1	2	1	1.33	0.57735	6.941	208.98	202.039	80.0755	111.972	1.45	1.6	0.15	1.52
1.36	2.54	1.18	1.95333	0.59003	4	6	2	5	1	3	14	11	9.33333	5.68624	2	3	1	2.67	0.57735	44.9559	196.967	152.011	120.211	76.0164	1.35	1.6	0.25	1.47
0.82	0.82	0	0.82	0	5	5	0	5	0	1	1	0	1	0	1	1	0	1	0	85.4678	85.4678	0	85.4678	0	2.35	2.35	0	2.35
1.38	3.44	2.06	2.75333	1.45664	2	9	7	4.33333	4.94975	6	16	10	12.6667	7.07107	2	3	1	2.33	0.70711	15.9994	238.029	222.03	90.0092	156.999	0.6	1.75	1.15	0.98
1.63	2.2	0.57	2.01	0.40305	4	5	1	4.66667	0.70711	5	10	5	8.33333	3.53553	3	3	0	3	0	50.9415	106.42	55.4785	87.9272	39.2292	1.35	1.4	0.05	1.38
1.25	2.54	1.29	1.91667	0.64609	5	8	3	6.33333	1.52753	11	15	4	13.3333	2.08167	2	3	1	2.67	0.57735	118.71	196.967	78.2566	161.537	39.6492	1.35	1.75	0.4	1.52
1.63	2.66	1.03	2.31667	0.72832	4	5	1	4.66667	0.70711	5	17	12	13	8.48528	2	3	1	2.33	0.70711	50.9415	126.904	75.963	101.583	53.7139	1.35	1.4	0.05	1.38
1.1	2.54	1.44	2.06	1.01823	6	8	2	6.66667	1.41421	11	11	0	11	0	3	3	0	3	0	158.925	196.967	38.0412	184.286	26.8992	1.35	1.75	0.4	1.48
1.2	2.05	0.85	1.72	0.45574	4	8	4	5.66667	2.08167	10	15	5	11.6667	2.88675	2	3	1	2.67	0.57735	58.6934	157.25	98.5566	112.568	49.9172	1.35	1.8	0.45	1.53
1.2	2.01	0.81	1.62	0.40583	4	8	4	5.33333	2.3094	9	14	5	11.6667	2.51661	2	3	1	2.67	0.57735	65.409	151.964	86.555	96.671	48.0214	1.25	1.85	0.6	1.48
2.2	2.55	0.35	2.43333	0.24749	2	6	4	3.33333	2.82843	8	14	6	12	4.24264	2	3	1	2.33	0.70711	12.0107	190.23	178.219	71.4171	126.02	0.7	1.3	0.6	0.9
1.31	2.2	0.89	1.90333	0.62933	1	3	2	1.66667	1.41421	1	2	1	1.33333	0.70711	1	1	0	1	0	1.00794	24.305	23.2971	8.77363	16.4735	0.25	1.5	1.25	0.67
1.23	2.05	0.82	1.77667	0.57983	5	8	3	6	2.12132	13	15	2	14.3333	1.41421	2	3	1	2.33	0.70711	121.76	164.93	43.1703	136.15	30.526	1.45	1.75	0.3	1.55
1.61	2.2	0.59	1.80667	0.41719	3	5	2	3.66667	1.41421	10	13	3	12	2.12132	2	3	1	2.33	0.70711	26.9815	106.42	79.4385	53.461	56.1715	1.25	1.4	0.15	1.3
1.54	2.2	0.66	1.98	0.46669	1	4	3	2	2.12132	1	4	3	2	2.12132	1	3	2	1.67	1.41421	1.00794	47.867	46.8591	16.6276	33.1344	0.25	1.4	1.15	0.63
0.95	3.16	2.21	2.42333	1.56271	3	5	2	3.66667	1.41421	2	17	15	12	10.6066	1	2	1	1.67	0.70711	35.453	87.62	52.167	52.842	36.8876	1	2	1	1.33
1.24	2.33	1.09	2.0575	0.77075	6	8	2	6.5	1.41421	14	14	0	14	0	2	3	1	2.25	0.70711	167.259	207.2	39.941	197.215	28.2426	1.75	1.8	0.05	1.79
1.36	1.81	0.45	1.6975	0.3182	4	4	0	4	0	3	13	10	10.5	7.07107	2	3	1	2.25	0.70711	44.9559	69.723	24.7671	63.5312	17.513	1.3	1.6	0.3	1.38
1.55	1.65	0.1	1.625	0.07071	4	4	0	4	0	7	12	5	10.75	3.53553	3	3	0	3	0	54.938	65.409	10.471	62.7913	7.40408	1.35	1.4	0.05	1.36
1.33	2	0.67	1.8325	0.47376	5	6	1	5.75	0.70711	4	12	8	10	5.65685	3	3	0	3	0	91.224	200.59	109.366	173.249	77.3334	1.5	1.55	0.05	1.51
2.04	3.04	1	2.545	0.44726	2	2	0	2	0	13	15	2	14	0.89443	2	2	0	2	0	10.811	14.0067	3.1957	12.2098	1.45118	0.65	0.85	0.2	0.73
1.31	1.93	0.62	1.715	0.32069	3	5	2	4.5	1.09545	2	12	10	9	5.13809	1	3	2	2.5	1.09545	24.305	112.411	88.106	88.1131	46.6579	1.5	1.6	0.1	1.56
1.1	2.2	1.1	1.925	0.77782	5	8	3	5.75	2.12132	10	11	1	10.25	0.70711	3	3	0	3	0	106.42	158.925	52.5054	119.546	37.1269	1.4	1.75	0.35	1.49
0.98	3.04	2.06	1.495	1.45664	2	2	0	2	0	1	15	14	4.5	9.89949	1	2	1	1.25	0.70711	6.941	14.0067	7.0657	8.70743	4.9962	0.65	1.45	0.8	1.25

[illegible]

1.6	2.28	0.68	2.05333	0.48083	5	6	1	5.66667	0.70711	5	10	5	8.33333	3.53553	3	3	[illegible]
1.63	2.2	0.57	2.01	0.40305	1	4	3	2	2.12132	1	5	4	2.33333	2.82843	1	3	[illegible]
1.57	2.54	0.97	2.21667	0.68589	2	6	4	4.66667	2.82843	2	11	9	8	6.36396	1	3	[illegible]
1.6	2.58	0.98	2.25333	0.69296	3	5	2	3.66667	1.41421	5	16	11	12.3333	7.77817	2	3	[illegible]
0.98	1.96	0.98	1.35333	0.53003	2	8	6	5	3	1	14	13	6.33333	6.80686	1	3	[illegible]
1.61	2.54	0.93	1.92	0.65761	3	6	3	4	2.12132	11	13	2	12.3333	1.41421	2	3	[illegible]
1.24	2.2	0.96	1.83	0.51643	5	8	3	6	1.73205	10	15	5	13	2.64575	2	3	[illegible]
2.02	2.2	0.18	2.08	0.12728	5	6	1	5.66667	0.70711	10	15	5	13.3333	3.53553	2	3	[illegible]
1.25	2.05	0.8	1.73667	0.42724	4	8	4	5.66667	2.08167	10	15	5	13.3333	2.88675	2	3	[illegible]
1.33	1.57	0.24	1.49	0.16971	2	5	3	3	2.12132	2	4	2	2.66667	1.41421	1	3	[illegible]
1.36	2.05	0.69	1.705	0.4879	5	9	4	7	2.82843	7	15	8	11	5.65685	2	3	[illegible]
1.9	2.96	1.06	2.60667	0.74953	4	4	0	4	0	11	17	6	15	4.24264	2	3	[illegible]
1.14	2.2	1.06	1.78667	0.56722	5	8	3	6.33333	1.52753	6	15	9	10.3333	4.50925	2	3	[illegible]
1.61	2.54	0.93	2.23	0.65761	3	6	3	5	2.12132	11	13	2	11.6667	1.41421	2	3	[illegible]
1.36	2.05	0.69	1.705	0.4879	5	9	4	7	2.82843	7	15	8	11	5.65685	2	3	[illegible]
1.9	3.04	1.14	2.49667	0.57187	2	3	1	2.33333	0.57735	14	15	1	14.3333	0.57735	2	2	[illegible]
1.13	2.2	1.07	1.84333	0.7566	1	8	7	3.33333	4.94975	1	5	4	2.33333	2.82843	1	3	[illegible]
1.54	2.58	1.04	2.23333	0.73539	3	4	1	3.33333	0.70711	4	16	12	12	8.48528	2	3	[illegible]
1.23	2.28	1.05	1.85333	0.55194	5	8	3	6.33333	1.52753	10	15	5	12.6667	2.51661	2	3	[illegible]
1.23	2.55	1.32	2.11	0.93338	2	8	6	4	4.24264	13	14	1	13.6667	0.70711	2	3	[illegible]
1.22	2.2	0.98	1.81333	0.52166	5	6	1	5.33333	0.57735	3	15	12	9.33333	6.02771	2	3	[illegible]
2.2	3.04	0.84	2.76	0.59397	2	6	4	3.33333	2.82843	8	15	7	12.6667	4.94975	2	3	[illegible]
[illegible]	[illegible]	[illegible]	[illegible]	[illegible]	[illegible]	[illegible]	[illegible]	[illegible]	[illegible]	[illegible]	[illegible]	[illegible]	[illegible]	[illegible]	[illegible]	[illegible]	[illegible]

0.82	3.98	3.16	2.992	1.71894	2	5	3	3	1.69031	1	17	16	12.4	8.43462	1	3	2	2	0.84515	18.9984	106.42	87.4216	40.5027
1.22	2.2	0.98	1.895	0.50829	5	8	3	5.75	1.64317	10	14	4	11.5	2.09762	2	3	1	2.75	0.54772	106.42	162.5	56.08	123.513
1.33	2.28	0.95	1.805	0.67175	5	6	1	5.5	0.70711	4	10	6	7	4.24264	3	3	0	3	0	91.224	195.084	103.86	143.154
0.79	2.55	1.76	2.1075	0.96218	2	6	4	4	2.52982	1	14	13	10	6.75278	1	3	2	2	0.89443	12.0107	196.967	184.956	88.4734
0.95	2.54	1.59	1.966	0.72885	3	6	3	4.6	1.69558	2	14	12	10.4	5.51135	1	3	2	2.2	0.93541	28.0855	196.967	168.881	107.545
1.23	1.9	0.67	1.626	0.3155	3	8	5	4.4	2.3184	7	14	7	11	4.10792	2	3	1	2.6	0.61237	28.0855	164.93	136.845	66.1955
1.12	2.28	1.16	2	0.60095	2	8	6	5.6	2.61861	4	13	9	9.4	3.92792	2	3	1	2.8	0.53452	10.811	195.084	184.273	147.236
1.22	2.19	0.97	1.884	0.44354	3	8	5	4.4	2.3184	10	15	5	12.4	2.80624	2	3	1	2.6	0.61237	30.9738	162.5	131.526	68.3669
1.24	2.19	0.95	1.888	0.43419	3	8	5	4.4	2.3184	10	15	5	12.8	2.89396	2	3	1	2.6	0.61237	30.9738	167.259	136.285	69.3187
1.2	2.2	1	1.992	0.49513	4	8	4	5.2	1.83712	8	15	7	11	4.10792	2	3	1	2.6	0.61237	74.9216	151.964	77.0424	100.789
0.79	3.16	2.37	2.562	1.22668	3	6	3	4.2	1.96396	1	17	16	12.6	8.34951	1	3	2	2	0.84515	35.453	196.967	161.514	87.2462
0.79	2.96	2.17	2.326	1.1494	4	6	2	4.6	1.06904	1	17	16	13.2	8.29802	1	2	1	1.8	0.53452	79.904	132.905	53.0015	98.2655
1.38	1.78	0.4	1.68	0.28284	5	9	4	6	2.82843	6	13	7	11.25	4.94975	2	3	1	2.25	0.70711	114.818	238.029	123.211	145.621
1.63	3.04	1.41	2.476	0.99702	2	4	2	2.8	1.41421	5	15	10	11	7.07107	2	3	1	2.4	0.70711	14.0067	50.9415	36.9348	28.7806
1.23	2.55	1.32	1.57	0.68369	2	8	6	5.8	3.62531	13	14	1	13.2	0.53452	2	3	1	2.6	0.65465	12.0107	164.93	152.92	106.757
1.55	3.04	1.49	1.978	0.78528	2	6	4	4	1.69031	7	15	8	9	4.14039	2	3	1	2.8	0.53452	14.0067	192.217	178.21	74.2076
1	3.04	2.04	1.646	1.11661	2	4	2	3.4	1.06904	2	15	13	7.2	8.5105	1	2	1	1.4	0.65465	14.0067	40.078	26.0713	33.0429
1.2	2.58	1.38	1.995	0.76344	3	8	5	5	2.68328	10	16	6	13.75	3.14643	2	3	1	2.25	0.54772	32.065	204.383	172.318	106.441
1.13	1.93	0.8	1.6925	0.41803	5	8	3	5.75	1.64317	5	13	8	10	3.79473	2	3	1	2.75	0.54772	107.868	140.908	33.0394	117.866
1.1	2.01	0.91	1.784	0.4319	4	8	4	4.8	2	11	16	5	13.2	2.42384	2	3	1	2.6	0.61237	63.546	173.04	109.494	89.0824
1.12	2	0.88	1.70667	0.62225	6	8	2	6.66667	1.41421	4	12	8	9.33333	5.65685	3	3	0	3	0	140.116	200.59	60.474	180.432
1.36	1.96	0.6	1.51	0.42426	5	9	4	8	2.82843	7	14	7	8.75	4.94975	2	3	1	2.75	0.70711	118.71	237	118.29	207.428
0.98	1.9	0.92	1.67	0.5039	2	4	2	3.25	1.04881	1	14	13	9.25	6.22093	1	3	2	2.25	1.04881	6.941	63.546	56.605	40.5296
2.28	3.44	1.16	3.05333	0.82024	2	6	4	3.33333	2.82843	10	16	6	14	4.24264	2	3	1	2.33	0.70711	15.9994	195.084	179.085	75.6943
1.12	2.54	1.42	1.89	0.71757	4	8	4	6	2	4	14	10	9.66667	5.1316	2	3	1	2.67	0.57735	72.64	196.967	124.327	136.574
1.27	2.54	1.27	2.11667	0.89803	6	8	2	6.66667	1.41421	11	17	6	13	4.24264	3	3	0	3	0	174.967	196.967	21.9996	189.633
1.61	1.9	0.29	1.7675	0.16909	3	4	1	3.75	0.54772	6	13	7	10.25	3.27109	2	3	1	2.75	0.54772	26.9815	63.546	36.5645	51.5174
0.98	2.01	1.03	1.6975	0.52706	2	4	2	3.5	1.09545	1	14	13	9.25	6.22093	1	3	2	2.25	1.04881	6.941	72.64	65.699	51.6683
1.31	2.2	0.89	1.9775	0.62933	3	5	2	4.5	1.41421	2	10	8	8	5.65685	1	3	2	2.5	1.41421	24.305	106.42	82.115	85.8913
2.16	2.28	0.12	2.24	0.08485	5	6	1	5.66667	0.70711	6	10	4	8.66667	2.82843	3	3	0	3	0	95.94	195.084	99.144	162.036
0.98	2.28	1.3	1.4625	0.6799	2	6	4	3.25	2.07364	1	13	12	6.25	6.77495	1	3	2	1.75	1.04881	6.941	195.084	188.143	58.9869
1.22	1.93	0.71	1.69333	0.50205	5	5	0	5	0	3	11	8	8.33333	5.65685	3	3	0	3	0	88.9059	107.868	18.9624	101.547
1.33	1.65	0.32	1.49	0.22627	4	5	1	4.5	0.70711	4	12	8	8	5.65685	3	3	0	3	0	65.409	91.224	25.815	78.3165
1.12	2.28	1.16	1.80667	0.60871	6	8	2	6.66667	1.1547	4	15	11	9.66667	5.50757	2	3	1	2.67	0.57735	140.116	208.98	68.8644	181.393
1.22	2.02	0.8	1.71667	0.43363	4	6	2	5	1	3	15	12	9.33333	6.02771	2	3	1	2.67	0.57735	58.6934	208.98	150.287	118.86
2.6	3.98	1.38	3.52	0.97581	2	5	3	3	2.12132	17	18	1	17.3333	0.70711	2	2	0	2	0	18.9984	131.293	112.295	56.4299
1.22	1.69	0.47	1.53333	0.33234	5	5	0	5	0	3	12	9	9	6.36396	3	3	0	3	0	88.9059	112.411	23.5052	104.576
1.2	2.55	1.35	2.1	0.95459	2	8	6	4	4.24264	9	14	5	12.3333	3.53553	2	3	1	2.33	0.70711	12.0107	151.964	139.953	58.6618
1	3.04	2.04	1.68	1.4425	2	4	2	3.33333	1.41421	2	15	13	6.33333	9.19239	1	2	1	1.33	0.70711	14.0067	40.078	26.0713	31.3876
1.66	2.66	1	2.32667	0.70711	4	5	1	4.66667	0.70711	6	17	11	13.3333	7.77817	2	3	1	2.33	0.70711	51.9961	126.904	74.9084	101.935
1.5	2.2	0.7	1.73333	0.49497	1	6	5	4.33333	3.53553	1	5	4	3.66667	2.82843	1	3	2	2.33	1.41421	1.00794	180.948	179.94	120.968

1.1	2.66	1.56	2.14	1.10309	5	8	3	6	2.12132	3	17	14	12.3333	9.89949	2	3	1	2.33	0.70711	126.904	138.905	12.001	130.905	8.48599	1.4	1.95	0.55	1.58	0.38891	33	97	64	75.6667	45.2548	6.15E-07	1E+15	1E+15	6.7E+14	7.1E+14	0	0	0	0	0	0.449	13	12.551	4.63267	8.8749	386.85	1193	806.15	655.567	570.034	7.7	28	20.3	14.4667	14.3543	0	0	0	0	0	116.831	139	3.94974	3.94974	7.99275	1.82047	1.82047	1.5708	-1.5424
2.2	2.55	0.35	2.375	0.24749	2	6	4	4	2.82843	8	14	6	11	4.24264	2	3	1	2.5	0.70711	12.0107	190.23	178.219	101.12	126.02	0.7	1.3	0.6	1	0.42426	63	95	32	79	22.6274	8.10E-08	1.00E-05	9.92E-06	5.04E-06	7.01E-06	4940	18350	13410	11645	9482.3	88	140	52	114	36.7696	3306	3800	494	3553	349.311	0	0	0	0	0	5.10E-06	7.10E-06	2.00E-06	6.10E-06	1.41E-06	40.7826	194	2.92027	2.92029	5.52197	1.5708	1.5708	2.09439	0.92447
1.91	2	0.09	1.982	0.06364	4	6	2	5.6	1.41421	10	12	2	11.6	1.41421	3	3	0	3	0	58.6934	200.59	141.897	172.211	100.336	1.35	1.5	0.15	1.47	0.10607	67	74	7	72.6	4.94975	7.20E-08	9.60E-07	8.88E-07	7.82E-07	6.28E-07	1407	4970	3563	2119.6	2519.42	8.3	91	82.7	24.84	58.4777	234.32	1728	1493.68	533.056	1056.19	25	180	155	56	109.602	0	0	0	0	0	118.23	229	5.35529	5.35529	5.35529	1.91063	1.91063	1.91063	0.06135
1.62	2.1	0.48	1.924	0.22045	4	6	2	4.8	0.93541	11	16	5	13.4	2.80624	2	3	1	2.4	0.61237	63.546	204.383	140.837	117.335	65.1313	1.35	1.9	0.55	1.48	0.26398	72	92	20	81.2	11.3578	1.72E-08	0.0001	1.00E-04	4.00E-05	6.12E-05	818	3570	2752	2635.6	1256.38	3	400	397	170.4	235.155	577	1357.77	780.77	947.572	423.903	43	140	97	90.6	51.4089	0	0	0	0	0	123.736	139	4.01168	4.01168	8.19517	1.81807	1.81807	1.5708	-0.1597
0.82	3.98	3.16	2.932	1.77487	2	4	2	2.8	1.30931	1	17	16	12.6	8.34951	1	3	2	2	0.84515	18.9984	63.546	44.5476	31.9279	23.5481	0.5	2.2	1.7	1.01	0.90869	10	102	92	77.6	47.7613	0	0	0	0	0	0	0	0	0	0	0.0277	400	399.972	100.017	207.005	53.53	1357.77	1304.24	370.978	675.401	0	0	0	0	0	0	0	0	0	0	62.2139	123	3.97279	3.97279	3.94181	1.5708	1.5708	1.5708	-2.3233
0.82	2.55	1.73	1.755	1.01337	4	8	4	5	2.19089	1	16	15	12.25	8.21584	1	3	2	2	0.89443	39.0983	173.04	133.942	92.5146	62.306	1.15	2.2	1.05	1.56	0.55924	10	93	83	53.25	50.3776	1.00E-08	2.50E-07	2.40E-07	8.63E-08	1.24E-07	1590	3350	1760	2572.5	1000.41	0.52	100	99.48	35.01	51.4538	336.53	1097	760.47	605.382	368.12	3.1	31	27.9	12.675	13.6494	0	0	0	0	0	122.479	166	4.30047	4.30047	8.04009	1.84153	1.5708	1.0472	-1.3138
0.93	2.58	1.65	1.84	0.9483	3	8	5	4.25	2.73861	1	17	16	12.5	8.41427	1	3	2	2	0.89443	22.9898	174.967	151.977	65.5217	80.0648	1	1.8	0.8	1.39	0.49066	11	94	83	54.75	49.8106	4.90E-08	1.00E+23	1.00E+23	5.00E+22	6.32E+22	0	0	0	0	0	0.205	140	139.795	39.1025	74.1352	370.87	1925	1554.13	768.148	844.894	6.3	48	41.7	17.425	22.3405	0	0	0	0	0	86.494	166	3.89132	3.89132	6.96786	1.85379	1.5708	1.0472	-2.0244
1.22	2.2	0.98	1.884	0.4476	3	5	2	4.2	1.22474	3	14	11	9.4	5.22015	2	3	1	2.6	0.61237	28.0855	101.07	72.9845	69.4434	42.5743	1.1	1.8	0.7	1.32	0.32016	25	85	60	63.8	27.4477	7.10E-08	0.001	0.001	0.0004	0.00061	2200	5970	3770	3928	2143.73	17	150	133	111.4	61.3372	1687	2607	920	2077.4	542.933	41	220	179	136.2	89.6674	2.60E-06	1.06E-05	8.00E-06	5.72E-06	3.72E-06	84.0138	139	4.18537	4.18537	5.63566	1.95124	1.95124	1.5708	-0.9045
1.25	1.9	0.65	1.582	0.36378	3	8	5	5	3.06186	13	15	2	14.2	0.93541	2	3	1	2.4	0.61237	26.9815	168.934	141.953	84.2042	86.4787	1.1	1.75	0.65	1.39	0.37375	21	85	64	58.4	38.2394	2.70E-08	0.001	0.001	0.0004	0.00061	0	0	0	0	0	17	235	218	113.8	106.141	933.47	1818	884.53	1588.69	416.011	45	100	55	73.2	30.7957	2.60E-06	2.31E-05	2.05E-05	1.10E-05	9.65E-06	98.2642	71	4.02973	5.52046	5.52046	1.94441	1.77225	1.94441	-0.7065
1.1	1.9	0.8	1.644	0.36551	3	8	5	4.4	2.3184	6	14	8	10.2	4.5	2	3	1	2.6	0.61237	28.0855	158.925	130.84	63.8177	60.9266	1.1	1.75	0.65	1.35	0.30104	26	85	59	62	27.4089	1.27E-07	0.001	0.001	0.0004	0.00061	2200	5940	3740	3780	2212.86	11	150	139	99.8	63.7201	1629	2180	551	1872.6	314.854	38.7	160	121.3	111.74	56.6474	2.60E-06	1.03E-05	7.70E-06	5.06E-06	3.52E-06	79.6441	139	3.8604	3.8604	6.00106	1.89826	1.89826	1.5708	-0.2873
1.1	1.9	0.8	1.74	0.4	3	8	5	4.4	2.3184	11	16	5	13.2	2.42384	2	3	1	2.6	0.61237	28.0855	173.04	144.955	71.2606	66.6292	1.1	1.75	0.65	1.33	0.29738	17	85	68	66.2	31.5971	1.72E-08	0.001	0.001	0.0004	0.00061	1590	3570	1980	2626	1002.89	39	400	361	227.8	182.908	1097	1687	590	1437.31	281.266	31	140	109	102.2	49.8021	2.60E-06	2.63E-05	2.37E-05	1.29E-05	1.14E-05	79.4915	139	4.01205	4.01205	5.69528	1.93075	1.93075	1.5708	-0.4618
1.24	2.55	1.31	1.576	0.67837	2	8	6	5.8	3.62531	13	14	1	13.8	0.53452	2	3	1	2.6	0.65465	12.0107	167.259	155.248	108.154	96.9402	0.7	1.75	1.05	1.44	0.55806	22	95	73	48.2	43.3458	2.70E-08	1.00E-05	9.97E-06	2.52E-06	5.02E-06	2830	18350	15520	6388	8078.33	15	235	220	84	119.851	933.47	3800	2866.53	2027.89	1266.49	33	76	43	48.2	19.4275	7.10E-06	2.31E-05	1.60E-05	1.34E-05	7.02E-06	112.228	221	4.82356	4.82356	4.82356	1.5708	1.5708	1.5708	-0.4337
1.2	2.55	1.35	2.118	0.62908	2	8	6	5	2.53546	9	14	5	10.2	2.59119	2	3	1	2.8	0.53452	12.0107	157.25	145.239	95.5954	62.53	0.7	1.8	1.1	1.31	0.46942	27	95	68	63.4	28.8543	4.30E-08	1.00E-05	9.96E-06	2.29E-06	5.19E-06	2680	18350	15670	7026	7638.05	11	150	139	120.2	73.1456	1585	3800	2215	2419.2	982.36	33	380	347	242.2	225.538	7.10E-06	9.40E-06	2.30E-06	8.22E-06	9.72E-07	73.7384	221	4.19338	4.19338	4.19338	1.5708	1.5708	1.5708	-0.4894
1.24	1.9	0.66	1.672	0.3015	3	8	5	4.4	2.3184	6	14	8	10.8	4.89898	2	3	1	2.6	0.61237	28.0855	167.259	139.174	65.4844	64.9983	1.1	1.75	0.65	1.35	0.30104	22	85	63	61.2	29.0732	1.27E-07	0.001	0.001	0.0004	0.00061	2200	5940	3740	3822	2180.72	15	150	135	100.6	61.9859	1687	2180	493	1907.2	283.33	44	160	116	112.8	54.5344	2.60E-06	1.22E-05	9.60E-06	5.44E-06	4.42E-06	78.0192	139	3.82553	3.82553	5.97814	1.89648	1.89648	1.5708	-0.4266
1.31	2.02	0.71	1.594	0.50205	3	6	3	4.2	2.12132	2	15	13	7.2	9.19239	1	2	1	1.4	0.70711	24.305	208.98	184.675	98.1752	130.585	1.5	1.6	0.1	1.54	0.07071	73	87	14	78.6	9.89949	4.40E-08	1.30E-06	1.26E-06	5.46E-07	8.88E-07	1790	4602	2812	3477.2	1988.38	8	160	152	99.2	107.48	544.4	923	378.6	771.56	267.711	31	45	14	39.4	9.89949	8.20E-06	1.34E-05	5.20E-06	1.03E-05	3.68E-06	140.787	164	4.68922	4.68928	7.39311	1.57078	1.5708	2.0944	-0.2238
1.22	2.55	1.33	1.564	0.68903	2	5	3	4	1.69031	3	14	11	7.2	6.88684	2	3	1	2.6	0.65465	12.0107	88.9059	76.8952	61.142	45.8776	0.7	1.8	1.1	1.47	0.58797	25	95	70	50	41.4039	2.70E-08	1.00E-05	9.97E-06	2.37E-06	5.11E-06	3300	18350	15050	6670	7859.43	17	235	218	85.2	118.618	933.47	3800	2866.53	2026.09	1267.07	33	76	43	46.4	20.2061	7.10E-06	2.31E-05	1.60E-05	1.24E-05	7.38E-06	116.908	221	4.88968	4.88968	4.88968	1.5708	1.5708	1.5708	-0.424

1.22	2.55	1.33	1.985	0.73672	4	8	4	5.5	2.09762	12	16	4	14.25	2.25832	2	3
1.6	3.98	2.38	3.385	1.68291	2	5	3	2.75	2.12132	5	17	12	14	8.48528	2	3
1.23	2.55	1.32	1.9875	0.73258	4	8	4	5.5	2.09762	13	16	3	14.5	1.89737	2	3
1.2	2.55	1.35	2.0325	0.71265	2	8	6	4	3.09839	8	14	6	11.5	3.28634	2	3
1.62	2.55	0.93	2.095	0.57581	4	6	2	4.5	1.09545	6	16	10	12.75	5.1672	2	3
1.24	2.55	1.31	1.99	0.72845	4	8	4	5.5	2.09762	13	16	3	14.75	1.64317	2	3
1.23	2.55	1.32	2.04	0.69868	2	8	6	4	3.09839	8	14	6	12.25	3.14643	2	3
0.79	3.98	3.19	2.6725	1.47223	2	6	4	4	1.78885	1	17	16	13	8.76356	1	2
1.93	3.44	1.51	2.7725	0.85871	2	5	3	3.5	1.89737	9	16	7	13	3.89872	2	3
1.33	1.91	0.58	1.69	0.30516	3	5	2	4	0.89443	4	13	9	9.25	4.13521	2	3
1.62	2.2	0.58	2.055	0.41012	5	6	1	5.25	0.70711	10	13	3	10.75	2.12132	2	3
1.27	2.55	1.28	2.05	0.68024	2	8	6	4	3.09839	8	17	9	13.25	4.13521	2	3
1.12	1.81	0.69	1.465	0.4879	4	8	4	6	2.82843	4	13	9	8.5	6.36396	2	3
1.1	1.78	0.68	1.27	0.48083	5	8	3	7.25	2.12132	3	13	10	5.5	7.07107	2	3
2.19	2.19	0	2.19	0	3	3	0	3	0	15	15	0	15	0	2	2
0.79	2.58	1.79	1.685	1.26572	3	6	3	4.5	2.12132	1	16	15	8.5	10.6066	1	2
1.23	2.2	0.97	1.8975	0.50298	5	8	3	5.75	1.64317	10	14	4	11.75	2.25832	2	3
0.98	2.2	1.22	1.6	0.54575	2	6	4	3.5	1.89737	1	13	12	9	6.19677	1	3
1.17	2.55	1.38	2.025	0.72672	2	8	6	4	3.09839	8	14	6	11	3.79473	2	3
2.33	3.44	1.11	2.885	0.78489	2	6	4	4	2.82843	14	16	2	15	1.41421	2	2
1.17	1.61	0.44	1.28	0.31113	3	8	5	6.75	3.53553	8	13	5	9.25	3.53553	2	3
1.63	3.04	1.41	2.335	0.99702	2	4	2	3	1.41421	5	15	10	10	7.07107	2	3
2	2.66	0.66	2.33	0.46669	5	6	1	5.5	0.70711	12	17	5	14.5	3.53553	2	3
1	3.44	2.44	2.445	1.32138	2	4	2	3	1.26491	2	16	14	11.25	7.23187	1	3

0.95	3.44	2.49	2.52	1.51424	2	5	3	3.2	1.96396	2	16	14	10.8	8.55236	1	3	2	2	0.84515	15.9994	91.224	75.2246	45.3684	48.0905	0.6	2	1.4	1.07	0.79237	15	101	86	73.4	47.401	0	0	0	0	0	0	0
0.89	3.44	2.55	2.508	1.53663	2	6	4	3.4	2.32993	2	16	14	10.8	8.55236	1	3	2	2	0.84515	15.9994	137.327	121.328	55.3098	67.2218	0.6	2.15	1.55	1.1	0.8567	14	101	87	73.2	47.842	0	0	0	0	0	317.5	3800
0.89	2.01	1.12	1.658	0.51225	4	6	2	4.8	0.93541	2	14	12	10.8	5.61249	1	3	2	2.2	0.93541	72.64	137.327	64.687	101.486	31.5608	1.25	2.15	0.9	1.55	0.41079	14	84	70	66.4	33.1342	7.00E-08	0.0005	0.0005	0.0002	0.00031	1620	5400
1.12	2.2	1.08	1.864	0.49432	3	8	5	4.8	2.29129	4	14	10	10.4	4.58258	2	3	1	2.6	0.61237	28.0855	140.116	112.031	81.8254	56.9636	1.1	1.85	0.75	1.37	0.34369	32	85	53	68	24.2126	1.08E-07	0.001	0.001	0.0004	0.00061	2100	3070
1.25	2.55	1.3	1.582	0.67307	2	8	6	5.8	3.62531	13	15	2	14.4	1.06904	2	3	1	2.6	0.65465	12.0107	168.934	156.924	109.159	98.0345	0.7	1.75	1.05	1.44	0.55806	21	95	74	47.6	43.9935	2.70E-08	1.00E-05	9.97E-06	2.41E-06	5.08E-06	0	0
0.89	2.2	1.31	1.824	0.63175	4	6	2	4.6	1.06904	2	14	12	10.8	6.23355	1	3	2	2	0.84515	72.64	137.327	64.687	92.3334	34.7766	1.25	2.15	0.9	1.46	0.46752	14	84	70	67	36.2531	1.08E-07	0.0005	0.0005	0.0003	0.00033	1620	5400
1.91	2.16	0.25	2.062	0.1163	2	5	3	3.6	1.69558	6	13	7	9.6	3.9211	2	3	1	2.6	0.61237	10.811	95.94	85.129	54.4391	47.6628	0.85	1.45	0.6	1.19	0.35	56	86	30	70.2	16.8893	5.50E-08	1E+12	1E+12	4E+11	6.1E+11	4970	16200
0.95	2.18	1.23	1.822	0.56703	4	5	1	4.2	0.5	2	15	13	10.8	5.93717	1	3	2	2.2	0.93541	63.546	87.62	24.074	72.911	11.1782	1.15	2	0.85	1.4	0.39131	15	89	74	67.4	34.101	1.72E-08	3.30E-07	3.13E-07	1.66E-07	1.76E-07	0	0
1.14	2.2	1.06	1.868	0.48492	3	8	5	5.2	2.42384	6	14	8	10.4	3.9211	2	3	1	2.6	0.61237	28.0855	192.217	164.132	116.969	93.3222	1.1	1.85	0.75	1.35	0.34233	30	85	55	66.4	25.1073	4.70E-08	0.001	0.001	0.0004	0.00061	2200	4825
1.13	2.2	1.07	1.866	0.48962	3	8	5	4.8	2.29129	5	14	9	9.8	4.5	2	3	1	2.6	0.61237	28.0855	140.908	112.822	79.8437	55.8674	1.1	1.85	0.75	1.33	0.34369	31	85	54	65	24.837	7.10E-08	0.001	0.001	0.0004	0.00061	2200	5970
1.55	3.04	1.49	1.92	0.77118	2	4	2	3.6	1.06904	7	15	8	9.2	4.17475	2	3	1	2.8	0.53452	14.0067	58.6934	44.6867	47.5028	22.4648	0.65	1.4	0.75	1.24	0.39506	60	100	40	69.4	20.764	0	0	0	0	0	333.6	5150
0.89	2.2	1.31	1.93	0.6501	4	6	2	4.8	0.93541	2	15	13	10.4	5.94769	1	3	2	2.2	0.93541	74.9216	137.327	62.4054	100.002	29.2275	1.15	2.15	1	1.45	0.45928	14	89	75	66	34.3693	1.08E-07	3.40E-07	2.32E-07	2.43E-07	1.38E-07	0	0
1.14	2.19	1.05	1.868	0.48117	3	8	5	4.4	2.3184	6	15	9	11.2	4.28661	2	3	1	2.6	0.61237	30.9738	144.242	113.268	64.7153	52.0637	1	1.85	0.85	1.31	0.39011	30	90	60	68.8	27.4477	7.20E-08	6.43E-07	5.71E-07	1.97E-07	2.79E-07	0	0
2.16	3.04	0.88	2.6	0.62225	2	5	3	3.5	2.12132	6	15	9	10.5	6.36396	2	3	1	2.5	0.70711	14.0067	95.94	81.9333	54.9733	57.9356	0.65	1.45	0.8	1.05	0.56569	56	100	44	78	31.1127	0	0	0	0	0	333.6	6190
0.89	3.04	2.15	1.945	1.33496	2	6	4	4.5	2.09762	2	17	15	9	8.89944	1	2	1	1.5	0.63246	14.0067	137.327	123.32	92.1412	64.3063	0.65	2.15	1.5	1.53	0.82158	14	100	86	56.5	53.7662	0	0	0	0	0	0	0
1.5	2.19	0.69	1.845	0.4879	3	6	3	4.5	2.12132	5	15	10	10	7.07107	2	3	1	2.5	0.70711	30.9738	180.948	149.974	105.961	106.048	1	1.45	0.45	1.23	0.3182	52	90	38	71	26.8701	1.00E-07	1.35E-07	3.50E-08	1.18E-07	2.47E-08	0	0
0.82	3.44	2.62	2.104	1.46607	2	6	4	3.6	1.87083	1	16	15	9.2	8.56592	1	3	2	1.8	0.93541	15.9994	200.59	184.591	62.1571	87.4788	0.6	2.2	1.6	1.42	0.89582	10	101	91	59.2	51.7047	0	0	0	0	0	317.5	2000
1.38	2.28	0.9	1.948	0.4137	3	9	6	5	2.73861	6	14	8	10.4	3.9211	2	3	1	2.6	0.61237	28.0855	238.029	209.943	100.002	95.8714	1.1	1.75	0.65	1.33	0.29738	45	85	40	69	18.7083	4.30E-08	0.001	0.001	0.0004	0.00061	2200	4700
1.14	2.28	1.14	2.004	0.5905	2	8	6	5.6	2.61861	6	13	7	9.8	2.9761	2	3	1	2.8	0.53452	10.811	195.084	184.273	148.061	95.4045	0.85	1.85	1	1.35	0.42258	30	86	56	64	24.5531	1.06E-07	1E+12	1E+12	2E+11	5.3E+11	2330	16200
1.2	2.18	0.98	1.876	0.44865	4	8	4	4.8	2	10	15	5	12	3.06186	2	3	1	2.6	0.61237	58.6934	157.25	98.5566	84.896	46.1222	1.15	1.8	0.65	1.36	0.29686	27	89	62	67.8	28.3108	7.20E-08	1.31E-06	1.24E-06	4.23E-07	5.73E-07	0	0
1.2	2.28	1.08	1.912	0.4931	3	8	5	5.2	2.42384	9	14	5	11.4	2.69258	2	3	1	2.6	0.61237	28.0855	195.084	166.999	119.661	95.5132	1.1	1.85	0.75	1.35	0.34233	18	85	67	64.8	30.7551	1.06E-07	0.001	0.001	0.0004	0.00061	0	0
1.55	3.04	1.49	1.994	0.79447	2	5	3	3.8	1.30931	7	15	8	9	4.14039	2	3	1	2.8	0.53452	14.0067	102.906	88.8988	56.3453	37.6371	0.65	1.4	0.75	1.24	0.39506	60	100	40	69	20.8738	0	0	0	0	0	333.6	5150
1.23	2.28	1.05	1.918	0.47961	3	8	5	5.2	2.42384	10	14	4	12.2	2.29129	2	3	1	2.6	0.61237	28.0855	195.084	166.999	122.254	97.0906	1.1	1.75	0.65	1.33	0.29738	23	85	62	65.8	28.3879	1.06E-07	0.001	0.001	0.0004	0.00061	2200	2760

[illegible]

1.54	2.18	0.64	1.86	0.45255	4	4	0	4	0	4	15	11	9.5	7.77817	2	3
1.78	2.2	0.42	2.095	0.29698	5	5	0	5	0	10	13	3	10.75	2.12132	2	3
1.33	2.2	0.87	1.765	0.61518	1	5	4	3	2.82843	1	4	3	2.5	2.12132	1	3
1.13	1.96	0.83	1.3375	0.5869	5	8	3	7.25	2.12132	5	14	9	7.25	6.36396	2	3
1.2	1.96	0.76	1.77	0.5374	5	8	3	5.75	2.12132	9	14	5	12.75	3.53553	2	3
1.78	2.28	0.5	2.155	0.35355	5	6	1	5.75	0.70711	10	13	3	10.75	2.12132	2	3
1.1	1.81	0.71	1.6325	0.50205	4	8	4	5	2.82843	11	13	2	12.5	1.41421	2	3
1.22	1.78	0.56	1.64	0.39598	5	8	3	5.75	2.12132	12	13	1	12.75	0.70711	2	3
1.14	2.2	1.06	1.935	0.74953	6	8	2	6.5	1.41421	6	9	3	8.25	2.12132	3	3
1.14	2.28	1.14	1.995	0.8061	6	8	2	6.5	1.41421	6	10	4	9	2.82843	3	3
1.91	2.18	0.27	2.045	0.19092	4	4	0	4	0	10	15	5	12.5	3.53553	2	3
0.98	2.2	1.22	1.835	0.63658	2	5	3	4.25	1.64317	1	14	13	8.75	6.02495	1	3
1.81	2.28	0.47	2.1625	0.33234	4	6	2	5.5	1.41421	10	13	3	10.75	2.12132	2	3
1.78	2.2	0.42	2.095	0.29698	5	5	0	5	0	10	13	3	10.75	2.12132	2	3
1.54	2.28	0.74	2.095	0.52326	4	5	1	4.75	0.70711	4	9	5	7.75	3.53553	3	3
1.83	2.2	0.37	1.9225	0.26163	4	5	1	4.25	0.70711	8	10	2	8.5	1.41421	3	3
2.28	2.58	0.3	2.43	0.21213	3	6	3	4.5	2.12132	10	16	6	13	4.24264	2	3
1.38	2.2	0.82	1.995	0.57983	6	9	3	6.75	2.12132	6	9	3	8.25	2.12132	3	3
1.93	2.05	0.12	1.96	0.08485	5	5	0	5	0	11	15	4	12	2.82843	2	3
1.3	2.28	0.98	2.035	0.69296	5	9	4	6	2.82843	4	9	5	7.75	3.53553	3	3
1.83	2.2	0.37	2.1075	0.26163	4	6	2	5.5	1.41421	8	9	1	8.75	0.70711	3	3
1.78	1.93	0.15	1.8925	0.10607	5	5	0	5	0	11	13	2	11.5	1.41421	2	3
0.98	3.04	2.06	2.525	1.45664	2	2	0	2	0	1	15	14	11.5	9.89949	1	2

0.98	1.78	0.8	1.3	0.56569	2	5	3	3.2	2.12132	1	13	12	5.8	8.48528	1	2	1	1.4	0.70711	6.941	114.818	107.877	50.0918	76.2806	1.45	1.55	0.1	1.49
0.89	2.01	1.12	1.626	0.51147	3	6	3	4	1.36931	2	14	12	11.2	5.77711	1	2	1	1.8	0.5	26.9815	137.327	110.345	67.314	50.6581	1.25	2.15	0.9	1.43
1	3.44	2.44	2.656	1.34572	2	5	3	3	1.69031	2	16	14	12.8	7.28991	1	2	1	1.8	0.53452	15.9994	118.71	102.711	41.3572	53.1647	0.6	1.8	1.2	1.01
0.98	3.04	2.06	1.874	1.20072	2	5	3	2.6	1.5	1	15	14	7.2	8.07775	1	3	2	1.8	0.93541	6.941	91.224	84.283	26.6239	40.5678	0.65	1.55	0.9	1.15
1.1	2.02	0.92	1.552	0.48717	3	8	5	5.2	2.42384	2	16	14	10	8.17771	1	3	2	1.8	0.93541	24.305	208.98	184.675	127.922	107.019	1.5	1.75	0.25	1.59
1.27	1.91	0.64	1.778	0.31755	3	8	5	4.4	2.3184	10	17	7	13	3.3541	2	3	1	2.6	0.61237	28.0855	174.967	146.882	69.705	67.9774	1.1	1.75	0.65	1.33
1.91	2.55	0.64	2.166	0.45255	4	4	0	4	0	10	16	6	12.4	4.24264	2	3	1	2.6	0.70711	58.6934	78.96	20.2666	66.8	14.3307	1.15	1.35	0.2	1.27
1.1	2.55	1.45	1.562	0.79617	2	8	6	6.2	3.20713	14	16	2	15.2	1.30931	2	3	1	2.6	0.65465	12.0107	173.04	161.029	129.968	83.6794	0.7	1.75	1.05	1.48
1.22	2.2	0.98	1.972	0.50923	2	8	6	5	2.53546	10	13	3	11	1.69031	2	3	1	2.8	0.53452	10.811	162.5	151.689	98.5142	65.3931	0.85	1.75	0.9	1.36
0.79	2.96	2.17	2.336	1.14401	4	6	2	4.4	1.06904	1	17	16	13.2	8.29802	1	2	1	1.8	0.53452	72.64	132.905	60.2655	89.0515	29.5414	1.15	2.6	1.45	1.46
1.2	2.28	1.08	2.016	0.55918	2	8	6	5	2.53546	9	13	4	9.8	2.13809	2	3	1	2.8	0.53452	10.811	151.964	141.153	94.2983	61.2889	0.85	1.85	1	1.35
1.12	2.2	1.08	1.908	0.50382	4	8	4	5.2	1.83712	4	14	10	9.6	4.84768	2	3	1	2.6	0.61237	72.64	140.116	67.476	97.5072	31.0124	1.25	1.85	0.6	1.39
2.02	2.1	0.08	2.068	0.05657	5	6	1	5.4	0.70711	15	16	1	15.6	0.70711	2	2	0	2	0	127.6	208.98	81.3804	160.152	57.5446	1.4	1.6	0.2	1.48
1.83	3.44	1.61	2.834	0.99504	2	6	4	3.2	2.13809	8	16	8	14.2	4.17475	2	3	1	2.2	0.53452	15.9994	208.98	192.981	62.5647	99.978	0.6	1.6	1	0.96
1.12	2.2	1.08	1.66	0.76368	5	8	3	6.5	2.12132	4	10	6	7	4.24264	3	3	0	3	0	106.42	140.116	33.696	123.268	23.8267	1.4	1.85	0.45	1.63
1	2.2	1.2	1.952	0.59511	4	5	1	4.4	0.61237	2	15	13	10.4	5.94769	1	3	2	2.2	0.93541	40.078	106.42	66.342	80.5522	30.8213	1.15	1.8	0.65	1.38
1	3.44	2.44	1.754	1.32016	2	6	4	4	1.69031	2	16	14	7.2	8.55236	1	2	1	1.4	0.65465	15.9994	207.2	191.201	68.6867	93.3833	0.6	1.8	1.2	1.56
1.25	2.04	0.79	1.798	0.36206	2	8	6	4	2.73861	8	15	7	11.4	3.58818	2	3	1	2.6	0.61237	10.811	168.934	158.123	60.4492	72.3259	0.85	1.75	0.9	1.25
0.89	2.2	1.31	1.694	0.82901	1	6	5	2.2	2.59119	1	2	1	1.2	0.53452	1	1	0	1	0	1.00794	137.327	136.319	29.4584	72.1381	0.25	2.15	1.9	0.87
0.95	2.2	1.25	1.706	0.8086	1	5	4	2	2.0702	1	2	1	1.2	0.53452	1	1	0	1	0	1.00794	87.62	86.6121	19.517	45.6067	0.25	2	1.75	0.84
0.79	2.66	1.87	2.156	0.97272	4	6	2	5	0.84515	1	17	16	13.2	8.29802	1	2	1	1.8	0.53452	72.64	132.905	60.2655	117.252	29.9689	1.25	2.6	1.35	1.61
1	3.44	2.44	2.644	1.35714	2	4	2	2.6	1.06904	2	16	14	12.8	7.28991	1	2	1	1.8	0.53452	15.9994	40.078	24.0786	23.2323	12.8768	0.6	1.8	1.2	0.94
1.31	2.18	0.87	1.658	0.61518	3	4	1	3.4	0.70711	2	15	13	7.2	9.19239	1	2	1	1.4	0.70711	24.305	74.9216	50.6166	44.5516	35.7913	1.15	1.5	0.35	1.36
1.17	1.9	0.73	1.614	0.33954	3	8	5	4.4	2.3184	7	14	7	10	4.10792	2	3	1	2.6	0.61237	28.0855	150.36	122.275	63.2814	56.4563	1.1	1.85	0.75	1.37
1.33	1.96	0.63	1.7775	0.32783	4	5	1	4.5	0.63246	4	14	10	9.5	4.51664	2	3	1	2.75	0.54772	58.6934	118.71	60.0166	81.8302	31.7426	1.35	1.55	0.2	1.43
1.33	3.44	2.11	2.796	1.13555	2	6	4	3.4	2.32993	4	16	12	13.2	6.23355	2	3	1	2.2	0.53452	15.9994	207.2	191.201	69.2844	100.036	0.6	1.8	1.2	1.03
0.82	3.44	2.62	1.688	1.47063	2	6	4	4	1.69031	1	16	15	6	8.45154	1	3	2	1.6	1.06904	15.9994	196.967	180.967	66.0522	88.284	0.6	2.2	1.6	1.71
1.2	2.28	1.08	2.016	0.55918	2	8	6	5	2.53546	9	13	4	10	2.0702	2	3	1	2.8	0.53452	10.811	157.25	146.439	95.3555	63.1034	0.85	1.8	0.95	1.34
1.12	2.2	1.08	1.87	0.51067	4	8	4	5.2	2.13809	4	14	10	11	5.34522	2	3	1	2.4	0.65465	72.64	192.217	119.577	110.051	65.0656	1.25	1.85	0.6	1.39
1.28	2.2	0.92	1.896	0.41994	3	9	6	5	2.73861	8	14	6	11.2	3	2	3	1	2.6	0.61237	28.0855	244	215.915	102.602	98.628	1.1	1.75	0.65	1.35
1.13	2.2	1.07	1.866	0.48962	3	8	5	5.2	2.42384	5	14	9	9.8	4.5	2	3	1	2.6	0.61237	28.0855	190.23	162.145	115.508	92.0212	1.1	1.85	0.75	1.33
1.23	2.01	0.78	1.67	0.3765	4	8	4	4.8	2	7	14	7	11	4.10792	2	3	1	2.6	0.61237	54.938	164.93	109.992	84.0173	51.5298	1.25	1.75	0.5	1.41
1.1	1.91	0.81	1.744	0.40254	3	8	5	4.4	2.3184	10	16	6	12.8	3	2	3	1	2.6	0.61237	28.0855	173.04	144.955	69.3196	67.0454	1.1	1.75	0.65	1.33
1.2	1.9	0.7	1.76	0.35	3	8	5	4.4	2.3184	9	14	5	11.8	2.42384	2	3	1	2.6	0.61237	28.0855	151.964	123.879	67.0454	56.6552	1.1	1.85	0.75	1.35
1.36	2.55	1.19	1.792	0.71311	2	6	4	4	1.69031	3	14	11	7.4	7.20119	2	3	1	2.6	0.65465	12.0107	207.2	195.189	70.8157	92.7071	0.7	1.8	1.1	1.46
0.89	2.2	1.31	1.794	0.61665	5	6	1	5.2	0.53452	2	14	12	10.8	6.23355	1	3	2	2	0.84515	106.42	137.327	30.907	119.975	13.2238	1.4	2.15	0.75	1.58
1.62	2.58	0.96	2.116	0.49051	3	6	3	4	1.36931	11	16	5	13.4	2.80624	2	3	1	2.4	0.61237	32.065	204.383	172.318	79.1211	80.2425	1	1.9	0.9	1.32
1.3	3.04	1.74	2.384	1.07695	2	9	7	4.2	3.81725	4	15	11	10.8	6.88684	2	3	1	2.4	0.65465	14.0067	232.038	218.031	91.0012	127.848	0.65	1.8	1.15	1.04
0.79	3.16	2.37	2.456	1.26227	3	6	3	3.8	1.55839	1	17	16	13.2	8.29802	1	2	1	1.8	0.53452	35.453	132.905	97.4525	62.3809	50.9001	1	2.6	1.6	1.37
2.1	2.18	0.08	2.132	0.05657	4	5	1	4.6	0.70711	15	16	1	15.6	0.70711	2	2	0	2	0	74.9216	127.6	52.6784	106.529	37.2493	1.15	1.4	0.25	1.3
1.22	2.55	1.33	1.604	0.70197	2	5	3	4.2	1.55839	3	14	11	7.2	6.88684	2	3	1	2.6	0.65465	12.0107	88.9059	76.8952	69.6903	39.797	0.7	1.8	1.1	1.48
1.33	2.02	0.69	1.75333	0.37072	4	6	2	5	1	4	15	11	9.66667	5.50757	2	3	1	2.67	0.57735	58.6934	208.98	150.287	119.633	79.0685	1.35	1.6	0.25	1.5
1.24	2.58	1.34	2.005	0.74691	3	8	5	5	2.68328	13	16	3	14.75	1.64317	2	3	1	2.25	0.54772	32.065	204.383	172.318	108.943	98.651	1	1.9	0.9	1.41
1.93	3.44	1.51	3.058	0.78159	2	5	3	2.6	1.60357	11	16	5	14.8	2.59119	2	3	1	2.2	0.53452	14.0067	107.868	93.8615	33.9746	49.383	0.6	1.6	1	0.81
0.98	2.28	1.3	1.728	0.7643	1	6	5	2.4	2.3184	1	10	9	2.8	4.5	1	3	2	1.4	1	1.00794	195.084	194.076	42.1964	95.6123	0.25	1.45	1.2	0.95
1.2	2.19	0.99	1.88	0.45291	3	8	5	4.4	2.3184	10	15	5	12	3.06186	2	3	1	2.6	0.61237	30.9738	157.25	126.276	67.3169	58.3051	1	1.8	0.8	1.3
0.89	2.18	1.29	1.782	0.59084	4	6	2	4.4	1	2	15	13	9.6	6.15427	1	3	2	2.2	0.93541	55.845	137.327	81.482	79.772	37.5193	1.15	2.15	1	1.45
1.62	2.58	0.96	2.2	0.51287	3	6	3	4.5	1.89737	13	16	3	15	1.54919	2	2	0	2	0	32.065	208.98	176.915	119.373	110.457	1	1.9	0.9	1.38
1.1	1.9	0.8	1.6	0.3687	3	8	5	4.4	2.3184	3	14	11	9	5.41987	2	3	1	2.6	0.61237	28.0855	138.905	110.82	60.9905	50.958	1.1	1.95	0.85	1.39
0.89	3.44	2.55	2.514	1.52848	2	9	7	4.2	3.81725	2	16	14	11.4	7.81939	1	3	2	2	0.84515	15.9994	237	221.001	84.465	119.708	0.6	2.15	1.55	1.14
1.1	2.01	0.91	1.792	0.47165	4	8	4	4.8	2.13809	3	14	11	10.6	5.95219	2	3	1	2.4	0.65465	55.845	138.905	83.0605	82.5341	38.6547	1.25	1.95	0.7	1.42
1.14	2.01	0.87	1.764	0.40277	4	8	4	4.8	2	6	14	8	10	4.1833	2	3	1	2.6	0.61237	55.845	144.242	88.397	80.2424	41.0868	1.25	1.85	0.6	1.43
